# Cloning of the *Lycopene β-cyclase* Gene in *Nicotiana tabacum* and Its Overexpression Confers Salt and Drought Tolerance

**DOI:** 10.3390/ijms161226243

**Published:** 2015-12-21

**Authors:** Yanmei Shi, Jinggong Guo, Wei Zhang, Lifeng Jin, Pingping Liu, Xia Chen, Feng Li, Pan Wei, Zefeng Li, Wenzheng Li, Chunyang Wei, Qingxia Zheng, Qiansi Chen, Jianfeng Zhang, Fucheng Lin, Lingbo Qu, John Hugh Snyder, Ran Wang

**Affiliations:** 1 College of Chemistry and Molecular Engineering, Zhengzhou University, Zhengzhou 450001, Henan, China; symzgh@163.com (Y.S.); qulingbo@zzu.edu.cn (L.Q.); 2 National Tobacco Gene Research Center, Zhengzhou Tobacco Research Institute, Zhengzhou 450001, Henan, China; jin_lf@126.com (L.J.); Liu_pingping2012@163.com (P.L.); chenxia0372@163.com (X.C.); likite2002@163.com (F.L.); weipan83@126.com (P.W.); ibi.zefeng@gmail.com (Z.L.); zhengqingxia916@126.com (Q.Z.); chen_qiansi@163.com (Q.C.); Jianfengzhang068@gmail.com (J.Z.); fuchenglin@zju.edu.cn (F.L.); johnhughsnyder@gmail.com (J.H.S.); 3 Institute of Plant Stress Biology, State Key Laboratory of Cotton Biology, School of Life Sciences, Henan University, Kaifeng 475004, Henan, China; jgguo@henu.edu.cn; 4 China National Tobacco Quality Supervision & Test Centre, Zhengzhou 450001, Henan, China; zhangw_jh@163.com; 5 Yunnan Academy of Tobacco Agricultural Sciences, Kunming 650031, Yunnan, China; lwz67@163.com; 6 Staff Training Academy of CNTC, Zhengzhou 450008, Henan, China; ztrisam@126.com

**Keywords:** lycopene β-cyclase, carotenoid biosynthesis, salt and drought tolerance, reactive oxygen species, abscisic acid, *Nicotiana tabacum*

## Abstract

Carotenoids are important pigments in plants that play crucial roles in plant growth and in plant responses to environmental stress. Lycopene β cyclase (β-LCY) functions at the branch point of the carotenoid biosynthesis pathway, catalyzing the cyclization of lycopene. Here, a β-LCY gene from *Nicotiana tabacum*, designated as *Ntβ-LCY1*, was cloned and functionally characterized. Robust expression of *Ntβ-LCY1* was found in leaves, and *Ntβ-LCY1* expression was obviously induced by salt, drought, and exogenous abscisic acid treatments. Strong accumulation of carotenoids and expression of carotenoid biosynthesis genes resulted from *Ntβ-LCY1* overexpression. Additionally, compared to wild-type plants, transgenic plants with overexpression showed enhanced tolerance to salt and drought stress with higher abscisic acid levels and lower levels of malondialdehyde and reactive oxygen species. Conversely, transgenic RNA interference plants had a clear albino phenotype in leaves, and some plants did not survive beyond the early developmental stages. The suppression of *Ntβ-LCY1* expression led to lower expression levels of genes in the carotenoid biosynthesis pathway and to reduced accumulation of carotenoids, chlorophyll, and abscisic acid. These results indicate that *Ntβ-LCY1* is not only a likely cyclization enzyme involved in carotenoid accumulation but also confers salt and drought stress tolerance in *Nicotiana tabacum*.

## 1. Introduction

Due to the ever increasing severity of environmental deterioration, both water scarcity and soil salinization have become major problems in agriculture that limit plant growth and cause serious economic losses [1]. Accordingly, the development of plants with high stress-tolerance traits is needed urgently [2,3]. An increasing number of studies have reinforced the assertion that the molecular manipulation of genes, such as those encoding antioxidant enzymes [4,5,6], transcription factors [7,8,9,10], and ion transporters [11,12], has the potential to overcome multiple limitations to agricultural productivity by creating stress-tolerant transgenic plants.

Carotenoids are terpenoids with a number of conjugated double bonds that contribute to their characteristic colors in the yellow to red range [13]. In plants, carotenoids play a critical role in the light absorption processes and protect the photosynthetic machinery from photo-oxidative damage by quenching triplet chlorophyll and singlet oxygen derived from excess light energy [14,15]. Additionally, carotenoids are precursors for the synthesis of the hormone abscisic acid (ABA), which functions in plants as an important signal in a variety of developmental processes and in adaptive stress responses to environmental stimuli [16,17]. Abiotic stresses can generate oxidative stress by increasing reactive oxygen species (ROS) production and/or by altering antioxidant defenses in plants [18]. Recently, many reports have demonstrated that increased carotenoid content in plants can improve tolerance to abiotic stresses such as high light conditions, UV irradiation, and salt stress, by scavenging ROS [19,20,21]. Moreover, carotenoid accumulation contributes to product quality and nutritional value for some crops, such as wheat [22], maize [23], tomatoes [24], potatoes [25], and watermelons [26].

The carotenoid biosynthetic pathway has been studied extensively in recent years [27]. Cyclization of lycopene by lycopene ε-cyclase (ε-LCY) and lycopene β-cyclase (β-LCY) is regarded as a key branching point in carotenogenesis in plants, as this is where the fate of lycopene shifts to the α-branch or the β-branch of the pathway, thereby determining the composition of the global carotenoid content. Over-expressing endogenous *β-LCY* in tomatoes caused a strong accumulation of β-carotene in the fruit that resulted from the near complete cyclization of lycopene [28]. Bang *et al.* found that a critical mutation in the red watermelon *β-LCY* allele might reduce β-LCY activity and thus result in the accumulation of lycopene [26]. Lutein accumulation is reduced or completely absent in the *lut1* and *lut2* mutants of Arabidopsis, owing to the lack of functional copies of the *ε-carotene hydroxylase* (*ε-OHase*) and *lycopene ε-cyclase* (*ε-LCY*) genes, respectively. These mutants also had increased accumulation of β-branch carotenoid compounds [29,30]. Transgenic tomatoes expressing *β-LCY* from *citrus* had increased β-carotene and total carotenoid content [31]. Chen *et al.* found that overexpression of the *β-LCY* gene in transgenic *Arabidopsis* enhanced plant tolerance to oxidative stress and salt stress [32]; these findings motivated us to investigate the function of *Ntβ-LCY* in carotenoid accumulation and in plant responses to drought and salt stress in tobacco.

Tobacco, a tetraploid plant species, has played a pioneering role in plant research, laying part of the groundwork for modern agricultural biotechnology. Modern tobacco cultivars have been developed to produce high carotenoid content, given that carotenoids are aromatic precursors for tobacco quality. CuiBi One (CB1, *Nicotiana tabacum*) is a famous tobacco cultivar in China, known for the high levels of carotenoids in its mature leaves. In this study, a *lycopene β-cyclase* gene named *Ntβ-LCY1* from tobacco was chosen for cloning and function characterization. The transcript expression levels of the *Ntβ-LCY* gene were analyzed in different developmental stages and in response to salt, drought, and ABA treatment, using both RNA sequencing and quantitative real-time PCR (qRT-PCR). The function of the *Ntβ-LCY1* gene in salt and drought stress tolerance was investigated with overexpression (OE) and RNA interference (RNAi) plants. Transgenic OE plants had significantly improved salt and drought tolerance compared to wild-type (WT) plants. Our results suggest that *Ntβ-LCY1* plays an important role in carotenoid accumulation and tolerance to abiotic stress, and indicate that *Ntβ-LCY1* may prove useful in potential applications for molecular breeding and/or biotechnology in plants.

## 2. Results

### 2.1. RNA Sequencing Analysis of Genes in the Carotenoid Biosynthetic Pathway and the Characterization of Ntβ-LCY Genes

**Figure 1:**
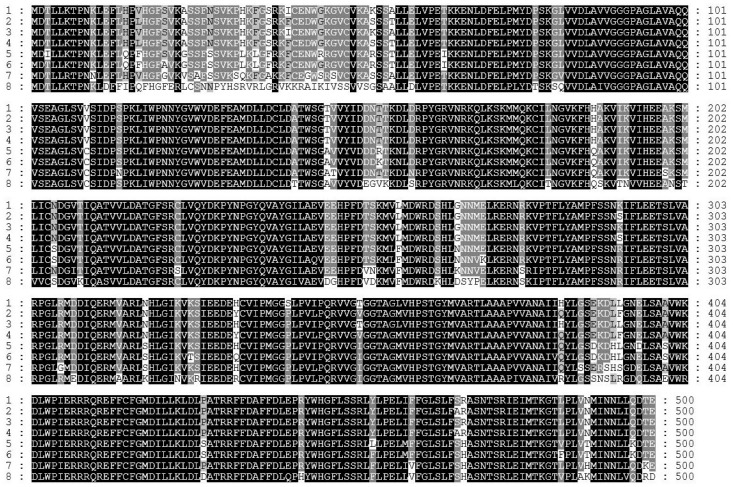
Comparison of β-LCY amino acid sequences by Genedoc software from *Nicotiana tabacum* (1: Ntβ-LCY1; 2: Ntβ-LCY2; this work), *Nicotiana tomentosiformis* (3: Ntomε-LCY, XM_009618113.1), *Nicotiana sylvestris* (4: Nsyε-LCY, XM_009795141.1), *Solanum tuberosum* (5: Stε-LCY, XM_006351204.1), *Solanum lycopersicum* (6: Slε-LCY, XM_010313794.1), *Capsicum annuum* (7: Caβ-LCY, GU085267.1), and *Arabidopsis* *thaliana* (8: Atε-LCY, AF117256.1). Blank back ground, completely conserved region; Grey back ground, partly conserved region; White back ground, non-conserved region.

**Figure 2:**
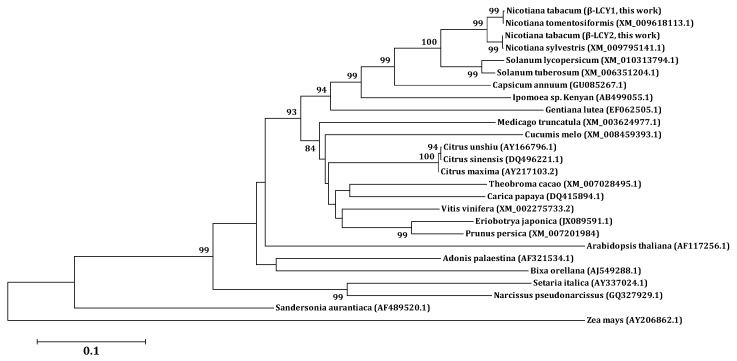
Phylogenetic analysis of the *β-LCY* genes in higher plants based on an alignment of the nucleotide sequences using MEGA5 software by the neighbor joining method. The bootstrap values were each estimated using 1000 replications.

To analyze the function of carotenoid biosynthetic genes in the CB1 cultivar, leaf samples were collected at the fast growing stage (FGS), the flowering stage (FS), the topping stage (TS), and the lower leaf maturity stage (LLMS). RNA sequencing was used to analyze the differential expression of various RNA transcripts. The transcript levels of the genes in the carotenoid biosynthetic pathway are displayed in Table 1. Interestingly, we found that two copies of *β-LCY* showed the highest transcript levels in the lower leaf maturity stage, although the transcript levels of most of the genes in the carotenoid biosynthetic pathway showed a declining trend from the flowering stage to lower leaf maturity stage, which suggested that the *β-LCY* gene might play an important role in the accumulation of carotenoids in mature tobacco leaves. There are two transcribed copies of *β-LCY* genes in the tobacco genome (China tobacco database V2.0). We found two *β-LCY* genes in the RNA sequencing results; these were designated as *Ntβ-LCY1* and *Ntβ-LCY2*. It can be seen from the results of the RNA sequencing that *Ntβ-LCY1* was more strongly expressed than *Ntβ-LCY2* in all four of the tested developmental stages of tobacco, which suggests that *Ntβ-LCY1* may be relatively more important for biological functions than *Ntβ-LCY2*. We designed primers to clone the coding region of *Ntβ-LCY1* from a CB1 leaf cDNA library. The cloned gene was 1503 bp in length and was predicted to encode a 500 amino acid protein with a calculated MW (molecular weight) of 56.05 kDa and a predicted pI of 6.68. Subsequently, we used a similar approach to clone the full-length *Ntβ-LCY1* gene from genomic DNA of CB1, and found that the complete gene sequence was 1503 bp in length, indicating that the *Ntβ-LCY1* gene had no introns. Sequence alignment revealed that the putative protein encoded by Ntβ-LCY1 likely shared high sequence identity with Ntβ-LCY2 and six other known β-LCY protein sequences from *Nicotiana tomentosiformis*, *Nicotiana sylvestris*, *Solanum tuberosum*, *Solanum lycopersicum*, *Capsicum annuum*, and *Arabidopsis* (Figure 1, Table 2). The coding regions of the *Ntβ-LCY1* and *Ntβ-LCY2* genes were highly similar to each other, with 97.1% identity between the two nucleotide sequences and 96.8% identity between the two amino acid sequences. In addition, the sequence identity between *Ntβ-LCY1* and *β-LCY* in *Nicotiana tomentosiformis* was 99.8%, while the sequence identity between *Ntβ-LCY2* and *β-LCY* in *Nicotiana sylvestris* was 100%*.* The results were validated by phylogenetic analysis done in MEGA5 using the UPGMA method [33]. According to the phylogenetic tree, *Ntβ-LCY1* was grouped with the *β-LCY* gene from *Nicotiana tomentosiformis*, while *Ntβ-LCY2* was grouped with the *β-LCY* gene from *Nicotiana sylvestris* (Figure 2). The *β-LCY*s genes of Solanaceae plants were clustered into a separate branch.

**Table 1:** Transcript levels of genes in the carotenoid biosynthetic pathway based on RNA sequencing analysis of samples from leaves of four different growth stages in tobacco, including the fast growing stage (FGS), the flowering stage (FS), the topping stage (TS), and the lower leaf maturity stage (LLMS). The full-length and coding sequences of gene in the carotenoid biosynthetic pathway are listed in Supplementary File 1.

Gene	Transcript ID	Gene ID	Sample_FGS	Sample_FS	Sample_TS	Sample_LLMS
*PSY1*	mRNA_108630_cds	Ntab0523090	50.00	20.26	26.26	26.84
*PSY2*	mRNA_24759_cds	Ntab0141080	5.72	6.43	5.15	4.42
*PSY3*	mRNA_28820_cds	Ntab0582610	90.19	31.8	35.02	48.05
*PSY4*	mRNA_3350_cds	Ntab0470140	46.29	35.54	24.17	26.29
*PDS1*	mRNA_13725_cds	Ntab0746310	79.50	42.74	56.13	65.04
*PDS2*	mRNA_73042_cds	Ntab0595110	103.66	63.22	80.42	64.72
*ZDS*	mRNA_101234_cds	Ntab0653840	82.89	62.56	70.39	62.62
*CRTISO1*	mRNA_114973_cds	Ntab0634540	44.61	26.42	33.46	23.74
*CRTISO2*	mRNA_122944_cds	Ntab0027300	24.79	14.27	19.2	22.76
*CRTISO3*	mRNA_78351_cds	Ntab0736080	14.34	15.26	15.03	18.19
*β-LCY1*	mRNA_46713_cds	Ntab0268950	54.02	37.97	37.51	55.58
*β-LCY2*	mRNA_18729_cds	Ntab0383390	43.56	34.1	29.14	44.63
*ε-LCY1*	mRNA_60735_cds	Ntab0006110	48.65	22.31	29.74	32.46
*ε-LCY2*	mRNA_99724_cds	Ntab0455950	26.79	14.13	11.97	13.38
*β-OHase1*	mRNA_106915_cds	Ntab0677920	55.63	26.45	25.08	30.60
*β-OHase2*	mRNA_120276_cds	Ntab0861090	4.22	1.65	2.48	0.16
*β-OHase3*	mRNA_121754_cds	Ntab0486180	77.56	70.69	73.00	96.59
*ε-OHase1*	mRNA_131608_cds	Ntab0299130	28.18	10.21	23.92	20.13
*ε-OHase2*	mRNA_140553_cds	Ntab0895820	27.02	15.45	19.14	26.27
*VDE1*	mRNA_114230_cds	Ntab0858420	53.14	39.84	69.32	21.80
*VDE2*	mRNA_119637_cds	Ntab0230700	107.99	84.86	95.45	41.86
*VDE3*	mRNA_130498_cds	Ntab0721110	8.82	5.59	10.51	5.70
*VDE4*	mRNA_86361_cds	Ntab0189070	8.52	4.45	5.66	5.53
*VDE5*	mRNA_95599_cds	Ntab0607170	1.03	0.00	0.46	0.84
*ZE1*	mRNA_119539_cds	Ntab0136170	316.73	279.76	267.74	297.37
*ZE2*	mRNA_42563_cds	Ntab0384590	381.6	339.56	352.04	353.57

**Table 2:** Sequence identities of the deduced Ntβ-LCY proteins with those of other plants.

Amino Acid		Ntomβ-LCY	Nsyβ-LCY	Stβ-LCY	Slβ-LCY	Caβ-LCY	Atβ-LCY
Identity (%)	Ntβ-LCY1	99.8	96.8	91.4	90.6	89.2	77.0
	Ntβ-LCY2	97.0	100	91.0	90.4	89.2	77.0

### 2.2. Examination of Ntβ-LCY Transcript Levels in Tobacco Organs and in Response to Stress Treatment

**Figure 3:**
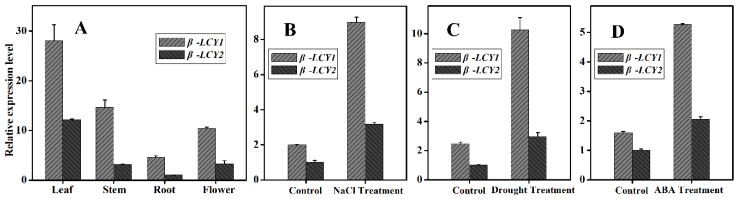
*Ntβ-LCY* expression. (**A**) Spatiotemporal expression of *Ntβ-LCY* in tobacco leaf, stem, root, and flower; (**B**,**C**) Relative expression levels of *Ntβ-LCY* following salt and drought stress, as compared with untreated control plants (control); (**D**) Relative expression level of *Ntβ-LCY* following ABA treatment. Error bars represent standard deviation (*n* = 3). The data presented here are representative of three independent experiments.

The spatial expression patterns of the *Ntβ-LCY* genes were investigated with qRT-PCR analysis of samples from different tissues (leaf, stem, root, and flower) at the flowering stage of *Nicotiana tabacum* grown under normal growth conditions. *Ntβ-LCY1* and *Ntβ-LCY2* were both strongly expressed in the leaves, had lower expression in stems and flowers, and were weakly expressed in roots (Figure 3A). The expression level of *Ntβ-LCY1* in all organs was higher than those of *Ntβ-LCY2*, a result consistent with the results of the CB1 RNA sequencing analysis.

*Ntβ-LCY* expression was evaluated after salt stress, drought stress, and treatment with the plant hormone ABA with qRT-PCR (Figure 3B–D). There were two important trends in the *Ntβ-LCY* gene expression patterns following these stress treatments. First, the transcript levels of the *Ntβ-LCY* genes in tobacco leaves were upregulated in response to both salt and drought stress treatment (Figure 3B,C), and the expression of the *Ntβ-LCY* genes was strongly induced by ABA treatment (Figure 3D). Second, the expression of the *Ntβ-LCY1* gene had a more pronounced response to stress treatment than the expression of *Ntβ-LCY2*. These results demonstrated that the *Ntβ-LCY* genes may take part in anti-stress processes in tobacco. Based on the differential degree of expression induction between the two genes, we speculate that *Ntβ-LCY1* may play a relatively more significant role in the stress resistance processes of tobacco.

### 2.3. Characterization of Ntβ-LCY1 OE and RNAi Transgenic Tobacco Plants

**Figure 4:**
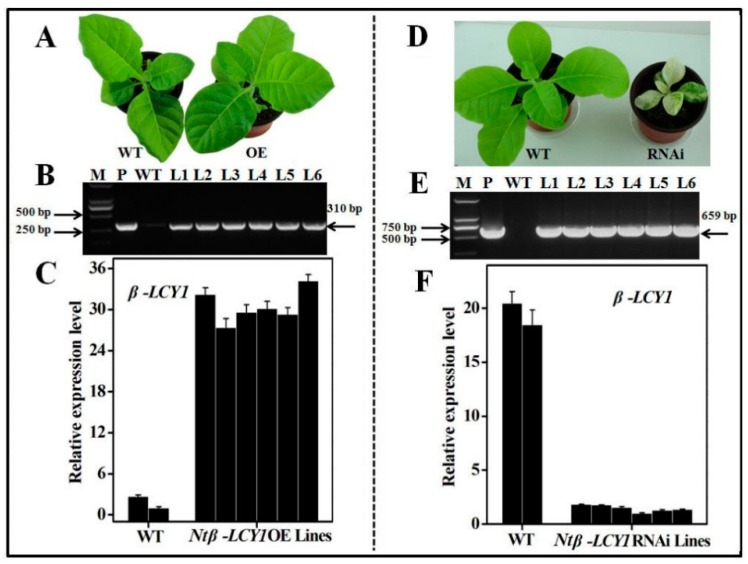
Identification and characterization of expression of the transgenic *Ntβ-LCY1* overexpression (OE) and RNAi plants by PCR and qRT-PCR. (**A**) Phenotypes of WT and *Ntβ-LCY1* OE transgenic tobacco; (**B**) Confirmation of the presence of the *Ntβ-LCY1* transgene construct in the OE transgenic plants based on PCR screening using primers flanking the *Ntβ-LCY1* gene. Lane 1: Marker DL2000; Lane 2: positive control; Lane 3: negative control. Lanes 4–9 are six independently *Ntβ-LCY1* OE transgenic lines; (**C**) Relative expression levels of *Ntβ-LCY1* in OE transgenic plants; (**D**) Phenotypes of WT and *Ntβ-LCY1* RNAi transgenic tobaccos; (**E**) Confirmation of the presence of the *Ntβ-LCY1* transgene construct in the RNAi transgenic lines based on PCR screening using primers of the kanamycin gene, Lane 1: Marker DL2000; Lane 2: positive control; Lane 3: negative control; Lanes 4–9: six independently *Ntβ-LCY1* RNAi transgenic lines; (**F**) Relative expression levels of *Ntβ-LCY1* in RNAi transgenic tobaccos. M, Marker DL2000; P, positive control (using plasmid as the PCR template); WT, negative control (DNA from WT lines used as the PCR template); L1–L6, DNA from L1–L6 transgenic lines used as the PCR template. Error bars represent standard deviation (*n* = 3). The data presented here are representative of three independent experiments.

Overexpressing and knockdown (RNAi) transgenic tobacco plants were generated to investigate the biological function of *Ntβ-LCY1* in abiotic stress responses. A total of 26 *Ntβ-LCY1* OE transgenic lines were obtained by hygromycin screening and PCR screening to amplify the inserted fragments spanning *Ntβ-LCY1* and the *Flag* gene in the vector. There were no obvious phenotypic difference between the *Ntβ-LCY1* OE and the WT lines (Figure 4A). Figure 4B shows that the PCR products of the six *Ntβ-LCY1* OE lines have a 310 bp band corresponding to the size of Ntβ-LCY1-Flag product. Comparatively, there was no 310 bp band in the PCR products of WT lines. Based on qRT-PCR analysis of the *Ntβ-LCY1* gene expression level (Figure 4C), six confirmed *Ntβ-LCY1* OE transgenic T1 lines were chosen for further analysis.

Twenty-three independent *Ntβ-LCY1* RNAi transgenic tobacco lines were verified by PCR and qRT-PCR to measure the *Ntβ-LCY1* gene transcript level. Transgenic *Ntβ-LCY1* RNAi lines exhibited abnormal phenotypes, including albino leaves and dwarfism, as compared to WT plants (Figure 4D), and some RNAi plants died during early developmental stages. Six T1 generation transgenic lines own with obvious phenotypes were selected for further analysis. Products of PCR using kanamycin-gene-specific primers from all six of the *Ntβ-LCY1* RNAi lines had a 659 bp band (Figure 4E), and the transcript levels of *Ntβ-LCY1* mRNA were significantly reduced in these RNAi plants (Figure 4F). Owing to the high degree of homology (97%), it was difficult to specifically silence only one copy. The expression of *Ntβ-LCY2* was also inhibited in the RNAi plants, which had silencing efficiencies ranging from 51%–67% in the L1–L6 RNAi transgenic lines.

**Figure 5:**
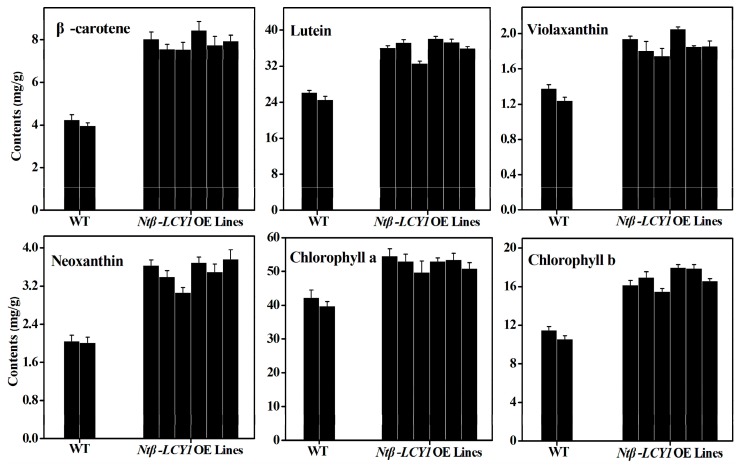
Carotenoid and chlorophyll content in WT and *Ntβ-LCY1* OE transgenic plants. Error bars represent standard deviation (*n* = 6). The data presented here are representative of three independent experiments.

**Figure 6:**
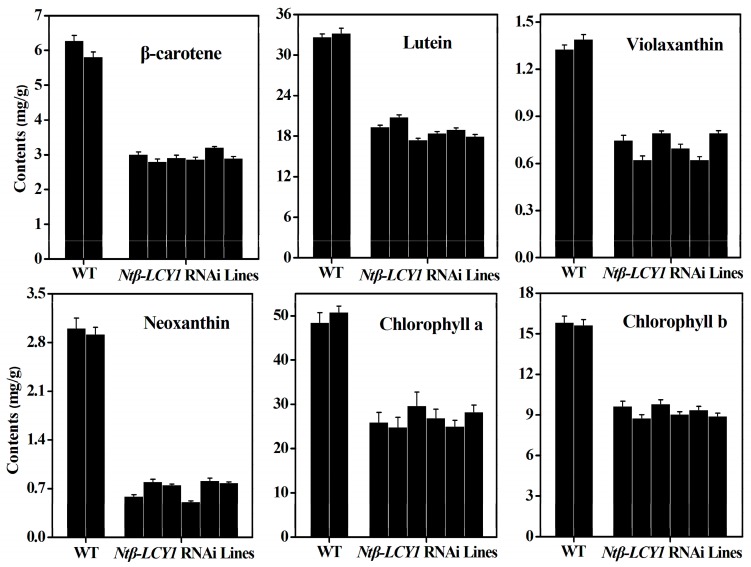
Carotenoid and chlorophyll content in control and *Ntβ-LCY1* RNAi transgenic plants. Error bars represent standard deviation (*n* = 6). The data presented here are representative of three independent experiments.

To further ascertain whether the *Ntβ-LCY1* expression levels in tobacco were correlated with carotenoid accumulation levels, the mRNA expression level of various carotenoid biosynthetic pathway genes and the carotenoid content were measured in *Ntβ-LCY1* OE and RNAi transgenic lines. The genes both up- and downstream of the *Ntβ-LCY* branch point in the carotenoid biosynthetic pathway, including *phytoene synthase* (*PSY*), *phytoene desaturase* (*PDS*), *ζ-carotene desaturase* (*ZDS*), *carotenoid isomerase* (*CRTISO*), *lycopene ε-cyclase* (*ε-LCY*), *β-carotene hydroxylase* (*β-OHase*), *zeaxanthin epoxidase* (*ZE*), *violaxanthin deepoxidase* (*VDE*), *neoxanthin synthase* (*NXS*), were all expressed at significantly elevated levels (*p* < 0.05) in the leaves of the *Ntβ-LCY1* OE lines as compared to the WT plants (Figure S1). Higher accumulation levels of carotenoids, including β-carotene, violaxanthin, neoxanthin, and lutein, as well as chlorophyll, were observed in the *Ntβ-LCY1* OE lines as compared to the WT plants (Figure 5). Consistently, in the RNAi transgenic lines, all of the genes of the carotenoid biosynthetic pathway showed dramatically (*p* < 0.05) reduced transcript levels as compared with the WT line (Figures S2 and S3). As expected, the carotenoid and chlorophyll content was markedly decreased in the RNAi transgenic lines (Figure 6).

### 2.4. Functional Analysis of Ntβ-LCY1 under Salt Stress

**Figure 7:**
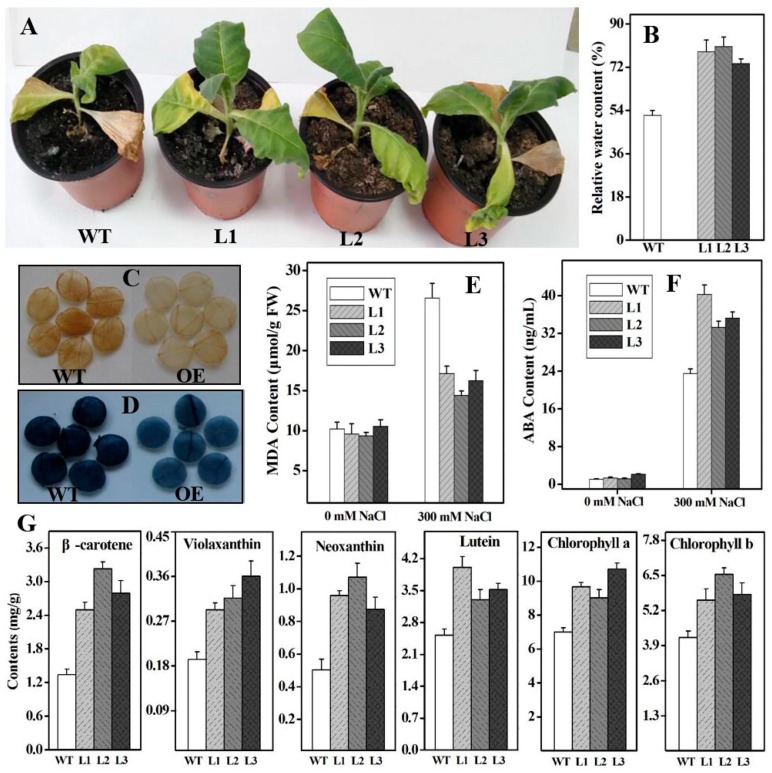
Effects of salt treatment on the T_1_ generation of *Ntβ-LCY1* OE transgenic plants. (**A**) Phenotypes of WT and *Ntβ-LCY1* OE plants after three weeks of treatment with 300 mM NaCl; (**B**) Relative water content in leaves of WT and OE plants after salt stress treatment; (**C**,**D**) 3,3,-diaminobenzidine (DAB) (**C**) and nitro blue tetrazolium (NBT) staining; (**D**) for evaluation the accumulation of H_2_O_2_ and O_2_^−^ in WT and OE plants after salt stress treatment; (**E**,**F**) Malondialdehyde (MDA) and abscisic acid (ABA) content in the leaves of WT and OE plants, with or without salt stress treatment; (**G**) Carotenoid and chlorophyll content in WT and *Ntβ-LCY1* OE transgenic lines after three weeks of salt stress treatment. L1–L3, three lines of *Ntβ-LCY1* OE transgenic plants. Error bars represent standard deviation (*n* = 6). The data presented here are representative of three independent experiments.

To test whether overexpressing *Ntβ-LCY1* in tobacco could enhance salt tolerance, six-week-old seedlings of WT and OE transgenic lines were treated with 300 mM NaCl. Under normal (no treatment) conditions, the increased *Ntβ-LCY1* expression in the OE plants did not lead to any major observable effects in plant architecture or growth habit. When subjected to salt stress for three weeks, the three OE lines grew well, with slightly yellow leaves, whereas the leaves of the WT plants were severely wilted and chlorotic (Figure 7A). Analysis of the relative water content (RWC) showed that the RWC of the leaves from the OE plants was higher than that of WT leaves (Figure 7B). The carotenoid and chlorophyll content were also investigated in these plants treated with salt stress. Although there were significant reductions in carotenoid and chlorophyll content in both OE and WT lines following three weeks of salt treatment, the carotenoid content (β-carotene, violaxanthin, neoxanthin, and lutein) and the chlorophyll content was obviously higher in the leaves of the OE transgenic plants (Figure 7G).

Abiotic stress often results in the substantial accumulation of ROS, causing membrane damage in plants. Therefore, the accumulation of H_2_O_2_ and of superoxide radical anions (O_2_^−^) was evaluated in *Ntβ-LCY1* OE transgenic plants grown under salt stress, using histochemical staining with 3,3,-diaminobenzidine (DAB, for H_2_O_2_) and nitro blue tetrazolium (NBT, for O_2_^−^). It can be seen from Figure 7C,D that leaves from OE lines exhibited less intense staining for both DAB and NBT than that of WT plants, indicating that OE plants accumulated lower levels of ROS under salt stress. Malondialdehyde (MDA) content is often assessed and used to represent the extent of lipid peroxidation and membrane injury in living cells [34,35]. Before salt treatment, there was no difference in the MDA content between the WT and the OE lines. However, following salt stress, the MDA content was significantly lower in the leaves of OE plants than in WT plants (Figure 7E). These results highlight the excellent salt stress resistance properties of the *Ntβ-LCY1* OE transgenic plants. Given its known role as an important hormone in plant abiotic stress resistance, we also measured the ABA content in transgenic and WT plants. Under normal growth conditions, there were no significant differences in ABA content (*p* > 0.05) between the WT and OE lines. By contrast, following the salt stress treatment, ABA content was higher (*p* < 0.05) in the leaves of OE transgenic lines than in the leaves of the WT lines (Figure 7F).

**Figure 8:**
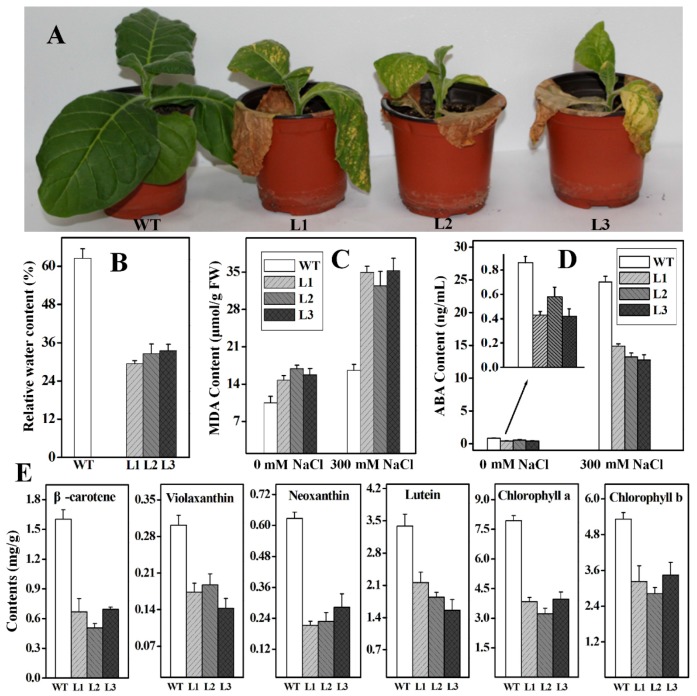
Effects of salt stress on *Ntβ-LCY1* RNAi transgenic plants. (**A**) Phenotypes of WT and *Ntβ-LCY1* RNAi plants under salt stress for eight days; (**B**) Relative water content in WT and RNAi plant leaves after salt stress for two weeks; (**C**,**D**) Malondialdehyde (MDA) and abscisic acid (ABA) content in leaves of WT and RNAi plants with or without salt stress; (**E**) Carotenoid and chlorophyll content in WT and *Ntβ-LCY1* RNAi transgenic plants after two weeks of salt treatment. Error bars represent standard deviation (*n* = 6). The data presented here are representative of three independent experiments.

The *Ntβ-LCY1* RNAi transgenic lines were also used to study plant responses to salt stress. Six week-old WT and RNAi transgenic plants were subjected to 300 mM NaCl stress treatment. It can be seen from Figure 8A that leaf wilting was more evident in the RNAi transgenic plants than in the WT plants after eight days of salt treatment. The RWC values of the RNAi plants were obviously lower than those of the WT plants after two weeks of salt stress treatment (Figure 8B). In addition, the MDA and the ABA content were also examined. As shown in Figure 8C,D, as compared to WT plants, the *Ntβ-LCY1* RNAi transgenic plants had higher MDA content and lower ABA content before and after the salt treatment. The carotenoid and the chlorophyll content decreased dramatically in the RNAi transgenic plants as compared to the WT plants, following salt stress treatment (Figure 8E). These results indicated that the attenuated expression of the *Ntβ-LCY1* gene in tobacco reduced plant tolerance to salt stress.

### 2.5. Functional Analysis of Ntβ-LCY1 under Drought Stress

**Figure 9:**
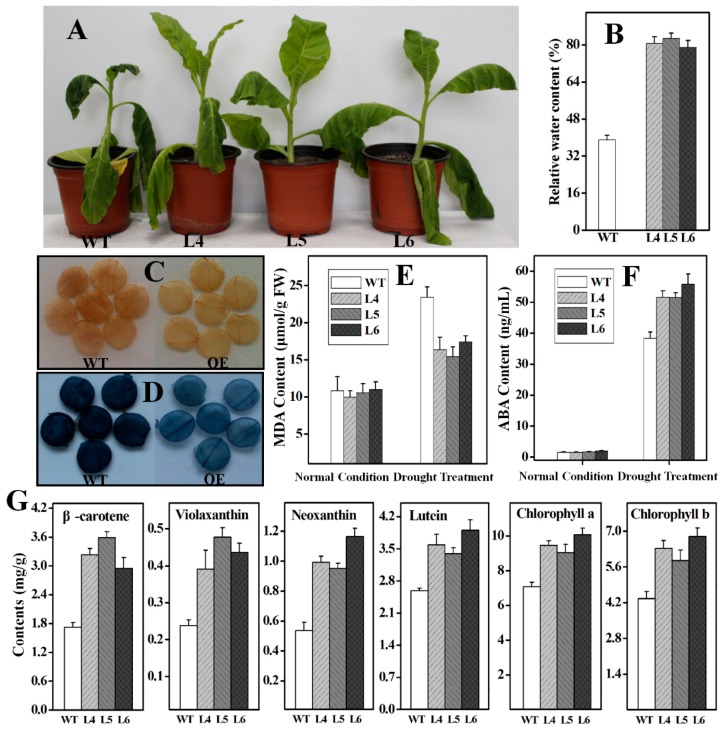
Effects of drought treatment on T_1_ generation *Ntβ-LCY1* OE transgenic plants. (**A**) Phenotypes of WT and *Ntβ-LCY1* OE plants after three weeks of drought stress; (**B**) Relative water content in leaves of WT and OE plants after drought stress; (**C**,**D**) 3,3,-diaminobenzidine (DAB) (**C**) and nitro blue tetrazolium (NBT) (**D**) staining for evaluation the accumulation of H_2_O_2_ and O_2_^−^ in WT and OE plants after drought stress; (**E**,**F**) Malondialdehyde (MDA) and abscisic acid (ABA) content in leaves of WT and OE plants treated with or without drought stress; (**G**) Carotenoid and chlorophyll content in WT and *Ntβ-LCY1* OE transgenic plants after three weeks of drought treatment. L4–L6, three lines of *Ntβ-LCY1* OE transgenic plants. Error bars represent standard deviation (*n* = 6). The data presented here are representative of three independent experiments.

In order to investigate whether *Ntβ-LCY1* is involved in the drought stress resistance of tobacco plants, seven-week-old WT and *Ntβ-LCY1* OE transgenic plants from lines 4, 5, and 6 were used for drought stress assays. Under normal growth conditions, plants of the three OE transgenic lines showed no obvious abnormal morphological phenotypes as compared with WT plants. However, after three weeks of water deprivation, the *Ntβ-LCY1* OE transgenic plants showed a reduced rate of leaf wilting (Figure 9A) and exhibited higher RWC values than did the WT plants (Figure 9B). The carotenoid content and chlorophyll content of the OE transgenic plants were higher than those of the WT plants following three weeks of drought treatment (Figure 9G). The histochemical staining assays indicated that the ROS content (H_2_O_2_ and O_2_^−^) (Figure 9C,D) and the MDA (Figure 9E) were lower in the *Ntβ-LCY1* OE plants than in the WT plants. Additionally, following drought stress, the ABA content was significantly higher in the leaves of the OE plants than in the WT plants (*p* < 0.05) (Figure 9F).

**Figure 10:**
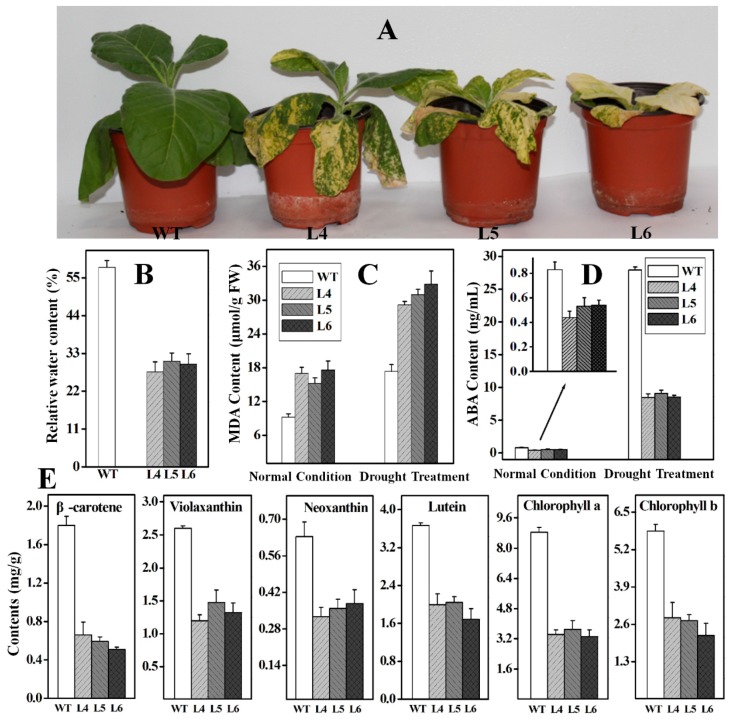
Effects of drought stress on *Ntβ-LCY1* RNAi transgenic plants. (**A**) Phenotypes of WT and *Ntβ-LCY1* RNAi plants under drought stress for eight days; (**B**) Relative water content in WT and RNAi plant leaves after drought treatment for two weeks; (**C**,**D**) Malondialdehyde (MDA) and abscisic acid (ABA) content in leaves of WT and RNAi plants with or without drought stress; (**E**) Carotenoids and chlorophyll content in WT and *Ntβ-LCY1* RNAi transgenic plants after two weeks of drought treatment. Error bars represent standard deviation (*n* = 6). The data presented here are representative of three independent experiments.

Following drought treatment for eight days, the leaves of *Ntβ-LCY1* RNAi plants exhibited a more serious wilting phenotype than did the WT plants (Figure 10A). The RWC values (Figure 10B) and the ABA content (Figure 10D) decreased, while the MDA content (Figure 10C) increased in RNAi plants compared to the WT plants after two weeks of drought treatment. In addition, the carotenoid content and the chlorophyll content were significantly lower in the *Ntβ-LCY1* RNAi transgenic plants than in the WT plants after drought treatment (Figure 10E).

## 3. Discussion

We used RNA sequencing data for the CB1 tobacco cultivar and the China tobacco database V2.0 to identify and investigate tobacco *β-LCY* genes expected to function in tobacco carotenoid biosynthesis. The function of the *Ntβ-LCY1* gene was characterized in detail. As an allotraploid plant, tobacco has homologous copies of functional genes; there are two copies of *Ntβ-LCY* in tobacco, *Ntβ-LCY1* and *Ntβ-LCY2*. Sequence analysis results showed that the *Ntβ-LCY* genes were highly conserved with homologous genes in other higher plants (Figure 1), suggesting that the tobacco *Ntβ-LCY* genes likely have similar biological functions to the *β-LCY* genes in other plants. Spatial-temporal expression analysis showed that *Ntβ-LCY* expression was, relatively, significantly higher in leaves than in other organs (Figure 3A), indicating that its functions were mainly in leaves. In all organs, *Ntβ-LCY1* was expressed more strongly than *Ntβ-LCY2*. Characterization of *Ntβ-LCY* expression following treatment with salt and drought stresses indicated that *Ntβ-LCY1* expression was more responsive to stress than was the expression of *Ntβ-LCY2* (Figure 3B,C), implying that *Ntβ-LCY1* likely has a relatively more important role in plant stress resistance than *Ntβ-LCY2*. ABA is known to act as an important signaling molecule in plant abiotic stress responses [36]. We found that the transcription levels of the *Ntβ-LCY* genes, especially that of *Ntβ-LCY1*, were dramatically upregulated in response to the exogenous application of ABA (Figure 3D), suggesting that these genes may possibly be involved in ABA-mediated stress responses.

Transgenic orchids with silenced *PSY* expression had lower carotenoid content than did WT plants, and had semi-dwarf and photo-bleaching phenotypes in plants. These changes were likely the result of unusual thylakoid membrane assembly or lipid phase changes of the membrane structure of the mutant plants [37]. Kim *et al.* [38] silenced the *β-LCY* gene in RNAi transgenic sweet potato calli; silencing significantly increased the total carotenoid content and led to a change in the color of transgenic calli from yellow to orange. The transgenic calli also enhanced the antioxidant activity compared to the nontransgenic (NT) calli. In the present study, OE and RNAi transgenic tobacco lines were generated for the *Ntβ-LCY1* gene. Compared to WT plants, the *Ntβ-LCY1* OE transgenic lines showed no morphological differences under normal conditions (Figure 4A). The *Ntβ-LCY1* RNAi transgenic lines had obvious phenotypes, including bleached leaves and retarded growth (Figure 4D). Most of the T_0_ *Ntβ-LCY1* RNAi transgenic seedlings died during the early growth stages, which indicated that the *Ntβ-LCY1* gene plays a vital role in plant growth. Similar results were obtained by Pogson *et al.* [39]; genetic lesions in *β-LCY* were lethal in *Arabidopsis*. The carotenoid and chlorophyll content was dramatically decreased in the *Ntβ-LCY1* RNAi transgenic lines that we generated (Figure 6), while the *Ntβ-LCY1* OE transgenic lines had increased accumulation of carotenoids and chlorophyll (Figure 5). qRT-PCR results for the OE and RNAi lines helped to explain the variation in pigment composition that we observed in the transgenic plants. The fact that the regulation of *Ntβ-LCY1* expression in tobacco had a strong impact on carotenoid content and on the expression levels of genes both up- and downstream of the *Ntβ-LCY* branch point of the pathway suggested that there might be a feedback mechanism in the regulation of the carotenoid pathway.

Salinity and drought are major abiotic environmental stressors, and plants can produce and accumulate numerous active oxygen species under stress conditions. The membrane stability of cells can be affected by lipid peroxidation caused by ROS [40]. Carotenoids provide protection for plants against oxidative stress as non-enzymatic antioxidants, by scavenging ROS generated due to excess excitation energy from chlorophyll during photosynthesis [41,42,43,44], and thus helping to maintain the redox state of the cell and facilitate proper functioning of the cell under stress. Over-expression of the *PSY* gene in transgenic *Arabidopsis* enhanced plant tolerance to reactive oxygen species under salt stress [21]. Silencing of the *β-OHase* genes in transgenic sweet potato resulted in elevated β-carotene and total carotenoid levels, as well as enhanced salt stress tolerance [45]. In a previous study, we found that silencing of the *ε-LCY* in *Nicotiana benthamiana* resulted in an increase in the accumulation of β-branch carotenoids and alleviated photoinhibition of Photosystem II in plants grown in low temperatures and under low light stress [46]. We further manipulated the *Ntε-LCY* expression levels in *Nicotiana tabacum* with transgenic technology and observed that strong accumulation of β-branch carotenoids and enhanced salt and drought tolerance resulted from the suppression of *Ntε-LCY* expression [47]. These results suggested that knocking down *ε-LCY* expression led to increased β-branch carotenoid biosynthesis and enhanced plant tolerance to environmental stresses. In the present study, we found that overexpressing the *Ntβ-LCY1* gene in tobacco dramatically improved plant tolerance to salinity and drought, while silencing of the *Ntβ-LCY1* gene decreased the ability of plants to tolerate salinity and drought stress. The ROS and MDA content were apparently lower in the *Ntβ-LCY1* OE plants than in the WT plants (Figure 7C–E and Figure 9C–E), indicating that the extent of cellular membrane injury due to salt and drought stress was less severe in the transgenic plants than in the WT plants. We also observed from MDA analysis that more lipid peroxidation occurred in the RNAi plants than in the WT plants, not only under stress conditions but also under normal conditions (Figure 8C and Figure 10C), suggesting that the silencing of the *Ntβ-LCY1* gene triggered severe oxidative damage and directly affected the normal growth of tobacco plants. The elevated accumulation of carotenoids improved the antioxidant activity of OE plants, while the lower carotenoid levels in the RNAi plants reduced the antioxidant capacity of plants. Transgenic sweet potato calli with silenced *ε-LCY* gene expression inhibited the production of H_2_O_2_ by increasing the carotenoid content in plants [48]. Transgenic *Arabidopsis* overexpressing *β-LCY* exhibited lower lipid peroxidation than WT plants, likely due to lower levels of MDA under abiotic stress conditions [32]. RWC is a typical phenotypic and physiological parameter used for evaluating plant vitality under stress conditions. Generally, water loss from plants largely depends upon stomatal aperture, which is closely regulated by ABA content [49,50]. The high RWC values in the *Ntβ-LCY1* OE plants is likely related to increased ABA content in the leaves of these plants. ABA synthesis was reduced, as a downstream effect of the inhibition of the carotenoid metabolic pathway, by silencing of *PSY* in transgenic orchids [37]. Logically, it followed that the ABA content was lower in the *Ntβ-LCY1* RNAi transgenic plants than in the WT plants in our study (Figure 8D and Figure 10D), implying that the sharp decline in carotenoid content obviously affected the downstream ABA biosynthesis. Downregulation of *β-OHase* and *ε-LCY* expression in sweet potato calli enhanced the accumulation of carotenoids and ABA, and further improved the tolerance to salt-mediated oxidative stress conditions [45,48]. Therefore, the increase in ABA content was more pronounced in the OE lines than in the WT plants under stress conditions (Figure 7F and Figure 9F), which might offer a reasonable explanation for the enhanced tolerance capacity to environmental stresses observed for the OE transgenic lines.

## 4. Materials and Methods

### 4.1. Plant Materials

Seeds of L. cv. Petit Havana SR1 (*Nicotiana tabacum*) were obtained from the stocks maintained in our laboratory. The L. cv. Petit Havana SR1 tobacco plants grew in a greenhouse maintaining day/night temperature at 28/23 °C and 16 h light photoperiod, at the National Tobacco Gene Research Center, Zhengzhou, China. Foliar discs (1.0 cm diameter) of L. cv. Petit Havana SR1 were excised from healthy and fully expanded tobacco leaves from six-week-old WT plants and used for plant transformation. Seeds of transgenic tobacco were planted on MS medium with 150 mg/L kanamycin (for RNAi plants) or 5 mg/L of hygromycin (for OE plants). Three weeks later, T_1_ tobacco seedlings with kanamycin (or hygromycin)-resistance were transferred into soil. The *Nicotiana tabacum* cultivar CB-1 plants used for cloning and for expression analysis were cultivated at the experimental farm in Yunnan Province, China. The tobacco leaves, stems, roots, and flowers at flowering stages used for the expression profiling were collected and stored at −80 °C. The effect of ABA (10 μM) on expression of *Ntβ-LCY* was tested by spraying a 10 μM ABA solution on six-week-old seedlings’ leaves and sampling the treated leaves 10 h later.

### 4.2. RNA Isolation and cDNA Preparation

An RNeasy Plant Mini Kit (Gene Answer, Beijing, China) was used to isolate total RNA of tobacco. For gene cloning and qRT-PCR analysis, first-strand cDNA was synthesized from total RNA using the Super Script First-Strand Synthesis System according to the manufacturer’s instructions (Takara, Japan).

### 4.3. cDNA Library Construction and Sequencing

For the synthesis of cDNA and Solexa sequencing, about 50 μg total RNA samples were prepared at concentrations of approximately 1000 ng/μL from tobacco leaves at four different growth stages: the fast growing stage, the flowering stage, the topping stage, and the lower leaf maturity stage. The cDNA library construction, sequencing, and bioinformatics analyses referred to the methods of Pang *et al.* [51].

### 4.4. Cloning of Ntβ-LCY1 and Vector Construction

The coding sequence of *Ntβ-LCY1* gene was amplified by PCR using high-fidelity DNA polymerase (PrimeSTAR^®^ HS DNA Polymerase, Takara, Otsu, Japan) from CB-1 leaves using the primers of β-LCY1-F & β-LCY1-R, and then cloned into the T vector (Takara). Clones containing the *Ntβ-LCY1* gene were further sequenced to confirm their sequences.

To construct the vector for gene overexpression in transgenic tobacco, the coding sequence of *Ntβ-LCY1* was amplified using the forward primer (β-LCY1-OE-F, including *SpeI* site) and the reverse primer (β-LCY1-OE-R, including *KpnI* site). The purified amplified gene fragment was then digested with *SpeI* and *KpnI* and ligated into the Sp1300-Flag plant vector (kindly provided by Professor Weiqiang Qian’s Lab at Peking University, Beijing, China).

For the construction of the *Ntβ-LCY1* RNAi vector, primers were designed from the sequence of a partial CDS of *Ntβ-LCY1* with attB sites using primers β-LCY1-RNAi-attB-F and β-LCY1-RNAi-attB-R. The partial *Ntβ-LCY1* fragment used in RNAi study was amplified from the cloned *Ntβ-LCY1* plasmid detailed above. Then the obtained PCR products were integrated into the RNAi expression vector (pHellsgate2, provided by Professor Weiqiang Qian of Peking University) by BP site-specific recombination (Invitrogen, Carlsbad, CA, USA). Primer sequences used in this study are listed in Table S1.

### 4.5. Plant Transformation and Confirmation

The construct of Ntβ-LCY1-pHellsgate2 and Ntβ-LCY1-Sp1300-Flag were introduced into *Agrobacterium tumefaciens* strain *GV3101*. *Agrobacterium*-mediated leaf disc transformation was performed to generate transgenic tobacco [52]. WT plants were used as controls in the experiments. The transformed plants were screened on MS medium with cephalosporin (250 mg/L) and either kanamycin (150 mg/L, for Ntβ-LCY1-RNAi plants) or hygromycin (5 mg/L, for Ntβ-LCY1-OE plants) and the surviving seedlings were grown in a greenhouse to produce seeds following self-pollination. The transgenic T_0_ line seeds were screened by germination on MS media with kanamycin (150 mg/L, for Ntβ-LCY1-RNAi plants) or hygromycin (5 mg/L, for Ntβ-LCY1-OE plants). The obtained resistant plants were transplanted to a close greenhouse for use in further analyses. The transgenic plants were confirmed through PCR and qRT-PCR analyses. The genomic DNA was isolated from leaves of tobacco for the PCR experiments. The integration of *Ntβ-LCY1* RNAi lines was confirmed by PCR using primers of *nptII* gene (nptII-F and nptII-R), while OE plants was confirmed with hygromycin gene primers (Hyg-F and Hyg-R) and *Ntβ-LCY1* gene flanking primers (β-LCY1-flanking-F and Flag-R). Primer sequences used in this study are listed in Table S1.

### 4.6. Gene Expression Analysis

qRT-PCR analysis was used to analyze the relative expression levels of the *Ntβ-LCY1* and other genes in the carotenoid biosynthetic pathway in transgenic and control plants. Briefly, the analysis was done with a Fluorescent Quantitative PCR Detector (Bio-Rad, Carlsbad, CA, USA) using SYBR Green fluorescence probe (Gene Answer, Beijing, China) and *26s* RNA (or *L25*) internal reference gene. qRT-PCR amplification products were assessed by melting curve analysis and gel electrophoresis to ensure amplification specificity. Three technical replicates were evaluated for each biological sample. The thermal cycling program for qRT-PCR cycling was 95 °C for 3 min, and then 40 cycles of 95 °C for 20 s and 60 °C for 20 s. The relative expression level of each gene was calculated using the 2^−ΔΔ*C*t^ method [53]. The qRT-PCR primers of *β-LCY1* and *β-LCY2* were designed based on the 3’-untranslated region (UTR) sequences of *Ntβ-LCY1* and *Ntβ-LCY2*, respectively*.* Primers used in the qRT-PCR analysis are listed in Table S2 [46].

### 4.7. Carotenoid and Chlorophyll Extraction and Quantification

Two hundred milligrams of freeze-dried leaf samples were used to extract carotenoids and chlorophyll with 25 mL of acetone. The samples were sonicated for 20 min and then centrifuged at 4 °C for 10 min at 6000 rpm. The obtained extract was filtered through a Millipore filter (0.22 μm, Shanghai Chuding Analytical Instruments Ltd., Shanghai, China) and analyzed by high performance liquid chromatography (HPLC, Agilent, Palo Alto, CA, USA).

For HPLC analysis, the carotenoids and chlorophyll were separated on an Agilent 1100 HPLC system with a C_18_ column (3.9 mm × 150 mm, 3 μm; Waters Corporation, Bedford, MA, USA) and analyzed with a diode array detector (DAD) at 448 and 428 nm. Solvent A was isopropanol. Solvent B was 80% acetonitrile-water.

### 4.8. Salt and Drought Stress Treatments

Six-week-old seedlings of *Nicotiana tabacum* cultivar CB-1 were subjected to salt and drought stress treatments. The mRNA expression of levels of the *Ntβ-LCY* genes in response to salt and drought stresses was examined. For the salt stress treatment, the seedlings were irrigated with 300 mM NaCl for 24 h. For the drought treatment, water was withheld from the plants for eight days.

Two-week-old transgenic and WT plants grown on MS medium were transplanted to pots filled with potting soil. The seedlings were cultivated for 4 weeks before salt and drought treatments. For the salt stress, plants were treated with 300 mM NaCl for three weeks for the OE lines or two weeks for the RNAi lines. For the drought treatment, water was withheld from plants for three weeks for the OE lines or two weeks for the RNAi lines. Phenotypic changes in the treated plants were carefully observed and photographed when obvious phenotypic appeared. The carotenoid and chlorophyll content, RWC, MDA, ABA, H_2_O_2_, and O_2_^−^ were all measured (methods detailed below) following the stress treatments. Each treatment was repeated three times.

### 4.9. Relative Water Content (RWC)

The RWC of leaves in transgenic (OE and RNAi) and WT plants were analyzed after the salt and drought treatments. First, the fresh weight of equally-sized discs leaves from treated tobaccos was measured after excision (FW). The leaf discs were then soaked in water overnight and the weight of inflated leaves was measured (IW) after careful drying of excess water. The dry weight (DW) of completely drying discs was also weighted after drying at 80 °C for 48 h in an incubator. The following formula was used to calculate the RWC: RWC (%) = (FW − DW)/(IW − DW) ×100 [54].

### 4.10. Detection of H_2_O_2_ and O_2_^−^

Following the salt and drought stress treatments, H_2_O_2_ accumulation was analyzed with the 3, 3,-diaminobenzidine (DAB, Sigma, St. Louis, MO, USA) method, and O_2_^−^ content was analyzed using nitroblue tetrazolium (NBT, Sigma) staining methods [55]. The leaf discs were excised from tobacco using a cork borer. These discs were then immersed in a solution containing 5 mg/mL DAB (pH 3.8) for 20 h or containing 0.5 mg/mL NBT for 20 h in the dark to analyze H_2_O_2_ and O_2_^−^, respectively. Stained samples were then depigmented in 75% (*v*/*v*) ethanol and 5% (*v*/*v*) glycerol at 80 °C for 10 min. After cooling to room temperature, the samples were transferred into fresh ethanol and finally photographed with a digital camera.

### 4.11. Determination of MDA Content

A thiobarbituric acid (TBA) method was employed to measure the content of Malondialdehyde (MDA) [56]. Three hundred milligram samples of tobacco leaves were ground in 5 mL of 5% (*w*/*v*) trichloroaceticic acid (TCA) and subsequently centrifuged for 10 min at 10,000 rpm. Then 2 mL of the resulting supernatant was transferred into a new tube and immediately mixed with 2 mL of TBA. The reaction mixture was heated at 98 °C for 30 min, cooled quickly on ice, and then centrifuged at 10,000 rpm for 20 min at 4 °C. Absorbance of the supernatant was measured at 450, 532, and 600 nm by an ultraviolet spectrophotometer (Cary100/300, Agilent). The MDA content, expressed as μmol/g, was calculated according to the following formula: MDA content (μmol/g) = C (μmol/L) ×V (L)/fresh weight (g) × 1000, where C (μmol/L) = 6.45× (A_532_ − A_600_) − 0.56A_450_ and V refers to the volume (L) of the extracting solution.

### 4.12. ABA Extraction and Quantification

Samples were prepared using a modification of the method reported by Liu *et al.* [57]. Tobacco leaves were ground to a powder in liquid nitrogen. One hundred milligrams of powder were dissolved into 1.0 mL of extraction solvent (CH_3_OH/H_2_O, 80/20, *v*/*v*, with internal standards ABA-*d*_6_ at 20 ng/mL). The mixture was sonicated for 30 min and then centrifuged at 4 °C for 5 min at 8000 rpm. The supernatant was then filtered through a 0.22-μm filter and evaluated with HPLC-MS/MS analysis.

A 1290 Infinity LC system coupled to a 6490 Triple Quad mass spectrometer (Agilent) was employed in the ABA determination. The chromatographic separation was completed using an Agilent SB-C_18_ column (2.1 mm × 100 mm, 1.8 μm) held at 50 °C, with a sample injection volume of 5 μL. Mobile phase A (MA) was 0.001% formic acid in water and mobile phase B (MB) was acetonitrile. The flow rate was 200 μL/min. The gradient elution method and mass spectrometer instrumental parameters were the same as in [47].

### 4.13. Statistical Analysis

All data were expressed as the mean ± SD of three independent replicates. One-way ANOVA tests were performed with SPSS for Windows Version 16.0 (SPSS Inc., Chicago, IL, USA). Values of *p* <0.05 were considered to be statistically significant.

## 5. Conclusions

In conclusion, we functionally characterized a stress-responsive *Ntβ-LCY1* gene from tobacco and confirmed its essential role in the survival of plants owing to its important role in carotenoid biosynthesis. Overexpressing of the *Ntβ-LCY1* gene can improve drought and salt tolerance by enhancing the ROS scavenging capacity of tobacco, making it an important candidate for modulating responses to abiotic stress in tobacco and in other plants.

## Supplementary Materials

Supplementary materials can be found at http://www.mdpi.com/1422-0067/16/12/26243/s1.

## References

[1] Mahajan S., Tuteja N. (2005). Cold, salinity and drought stresses: An overview. Arch. Biochem. Biophys..

[2] Bromham L., Saslis-Lagoudakis C.H., Bennett T.H., Flowers T.J. (2013). Soil alkalinity and salt tolerance: adapting to multiple stresses. Biol. Lett..

[3] Evers D., Lefevre I., Legay S., Lamoureux D., Hausman J.F., Rosales R.O.G., Marca L.R.T., Hoffmann L., Bonierbale M., Schafleitner R. (2010). Identification of drought-responsive compounds in potato through a combined transcriptomic and targeted metabolite approach. J. Exp. Bot..

[4] Natwar S., Avinash M., Bhavanath J. (2014). Over-expression of the Peroxisomal Ascorbate Peroxidase (*SbpAPX*) gene cloned from Halophyte Salicornia brachiata confers salt and drought stress tolerance in transgenic Tobacco. Mar. Biotechnol..

[5] Wu G., Wang G., Ji J., Gao H., Guan W., Wu J., Guan C., Wang Y. (2014). Cloning of a cytosolic ascorbate peroxidase gene from Lycium chinense Mill. and enhanced salt tolerance by overexpressing in tobacco. Gene.

[6] Zhang D.Y., Yang H.L., Li X.S., Li H.Y., Wang Y.C. (2014). Overexpression of Tamarix albiflonum TaMnSOD increases drought tolerance in transgenic cotton. Mol. Breed..

[7] Tang L.L., Cai H., Zhai H., Luo X., Wang Z.Y., Cui L., Bai X. (2014). Overexpression of Glycine soja WRKY20 enhances both drought and salt tolerance in transgenic alfalfa (*Medicago sativa* L.). Plant Cell Tissue Organ Cult..

[8] Xu Q., He Q., Li S., Tian Z. (2014). Molecular characterization of *StNAC2* in potato and its overexpression confers drought and salt tolerance. Acta Physiol. Plant.

[9] Min D.H., Zhao Y., Huo D.Y., Li L.C., Chen M., Xu Z.S., Ma Y.Z. (2013). Isolation and identification of a wheat gene encoding a zinc finger protein (TaZnFP) responsive to abiotic stresses. Acta Physiol. Plant.

[10] Sun P.P., Zhu X.F., Huang X.S., Liu J.H. (2014). Overexpression of a stress-responsive MYB transcription factor of Poncirus trifoliata confers enhanced dehydration tolerance and increases polyamine biosynthesis. Plant Physiol. Biochem..

[11] Muhammad R., Khurram S., Rashid A., Moddassir A., Imran H., Shahid M., Gerald A.B., Nasir A.S. (2014). Cloning and characterization of Na^+^/H^+^ antiporter (*LfNHX1*) gene from a halophyte grass *Leptochloa fusca* for drought and salt tolerance. Mol. Biol. Rep..

[12] Yarra R., He S.J., Abbagani S., Ma B., Bulle M., Zhang W.K. (2012). Overexpression of a wheat Na^+^/H^+^ antiporter gene (*TaNHX2*) enhances tolerance to salt stress in transgenic tomato plants (*Solanum lycopersicum* L.). Plant Cell Tissue Organ Cult..

[13] Bramley P.M. (1997). The regulation and genetic manipulation of carotenoid biosynthesis in tomato fruit. Pure Appl. Chem..

[14] Dall’Osto L., Fiore A., Cazzaniga S., Giuliano G., Bassi R. (2007). Different roles of α- and β-branch xanthophylls in photosystem assembly and photoprotection. J. Biol. Chem..

[15] Andrade-Souza V., Costa M.G.C., Chen C.X., Gmitter F.G., Costa M.A. (2011). Physical location of the carotenoid biosynthesis genes *Psy* and *β-Lcy*
*in Capsicum annuum* (Solanaceae) using heterologous probes *from Citrus sinensis* (Rutaceae). Genet. Mol. Res..

[16] Nambara E., Marion-Poll A. (2005). Abscisic acid biosynthesis and catabolism. Annu. Rev. Plant Biol..

[17] Taylor I.B., Sonneveld T., Bugg T.D.H., Thompson A.J. (2005). Regulation and manipulation of the biosynthesis of abscisic acid, including the supply of xanthophyll precursors. J. Plant Growth Regul..

[18] Miller G., Suzuki N., Ciftci-Yilmaz S., Mittler R. (2010). Reactive oxygen species homeostasis and signaling during drought and salinity stresses. Plant Cell Environ..

[19] Götz T., Sandmann G., Römer S. (2002). Expression of bacterial carotene hydroxylase gene (*crtZ*) enhances UV tolerance in tobacco. Plant Mol. Biol..

[20] Davison P.A., Hunter C.N., Horton P. (2002). Overexpression of β-carotene hydroxylase enhances stress tolerance in Arabidopsis. Nature.

[21] Han H., Li Y., Zhou S. (2008). Overexpression of phytoene synthase gene from Salicornia europaea alters response to reactive oxygen species under salt stress in transgenic Arabidopsis. Biotechnol. Lett..

[22] Howitt C.A., Cavanagh C.R., Bowerman A.F., Cazzonelli C., Rampling L., Mimica J.L., Pogson B.J. (2009). Alternative splicing, activation of cryptic exons and amino acid substitutions in carotenoid biosynthetic genes are associated with lutein accumulation in wheat endosperm. Funct. Integr. Genom..

[23] Harjes C.E., Rocheford T.R., Bai L., Brutnell T.P., Kandianis C.B., Sowinski S.G., Stapleton A.E., Vallabhaneni R., Williams M., Wurtzel E.T. (2008). Natural genetic variation in lycopene epsilon cyclase tapped for maize biofortification. Science.

[24] Apel W., Bock R. (2009). Enhancement of carotenoid biosynthesis in transplastomic tomatoes by induced lycopene-to-provitamin A conversion. Plant Physiol..

[25] Diretto G., Welsch R., Tavazza R., Mourgues F., Pizzichini D., Beyer P., Giuliano G. (2007). Silencing of beta-carotene hydroxylase increases total carotenoid and beta-carotene levels in potato tubers. BMC Plant Biol..

[26] Bang H., Kim S., Leskovar D., King S. (2007). Development of a codominant CAPS marker for allelic selection between canary yellow and red watermelon based on SNP in lycopene β-cyclase (*LCYB*) gene. Mol. Breed..

[27] Cazzonelli C.I., Pogson B.J. (2010). Source to sink: regulation of carotenoid biosynthesis in plants. Trends Plant Sci..

[28] D’Ambrosio C., Giorio G., Marino I., Merendino A., Petrozza A., Salfi L., Stigliani A., Cellini F. (2004). Virtually complete conversion of lycopene into β-carotene in fruits of tomato plants transformed with the tomato lycopene β-cyclase (tlcy-β) cDNA. Plant Sci..

[29] Cunningham F.X., Pogson B., Sun Z., McDonald K.A., DellaPenna D., Gantt E. (1996). Functional analysis of the beta and epsilon lycopene cyclase enzymes of Arabidopsis reveals a mechanism for control of cyclic carotenoid formation. Plant Cell.

[30] Tian L., Musetti V., Kim J., Magallanes-Lundback M., DellaPenna D. (2004). The Arabidopsis *LUT1* locus encodes a member of the cytochrome P450 family that is required for carotenoid epsilon-ring hydroxylation activity. Proc. Natl. Acad. Sci. USA.

[31] Guo F., Zhou W., Zhang J., Xu Q., Deng X. (2012). Effect of the Citrus Lycopene β-Cyclase Transgene on Carotenoid Metabolism in Transgenic Tomato Fruits. PLoS ONE.

[32] Chen X.Y., Han H.P., Jiang P., Nie L.L., Bao H.X., Fan P.X., Lv S., Feng J., Li Y. (2011). Transformation of β-lycopene cyclase genes from *Salicornia europaea* and Arabidopsis conferred salt tolerance in Arabidopsis and tobacco. Plant Cell Physiol..

[33] Tamura K., Peterson D., Peterson N., Stecher G., Nei M., Kumar S. (2011). MEGA5: Molecular evolutionary genetics analysis using maximum likelihood, evolutionary distance, and maximum parsimony methods. Mol. Biol. Evol..

[34] Robles P., Micol J.L., Quesada V. (2012). Arabidopsis MDA1, a nuclearencoded protein, functions in chloroplast development and abiotic stress responses. PLoS ONE.

[35] Zhou M.L., Ma J.T., Zhao Y.M., Wei Y.H., Tang Y.X., Wu Y.M. (2012). Improvement of drought and salt tolerance in *Arabidopsis* and *Lotus corniculatus* by overexpression of a novel DREB transcription factor from *Populus euphratica*. Gene.

[36] Danquah A., de Zelicourt A., Colcombet J., Hirt H. (2014). The role of ABA and MAPK signaling pathways in plant abiotic stress responses. Biotechnol. Adv..

[37] Liu J.X., Chiou C.Y., Shen C.H., Chen P.J., Liu Y.C., Jian C.D., Shen X.L., Shen F.Q., Yeh K.W. (2014). RNA interference-based gene silencing of phytoene synthase impairs growth, carotenoids, and plastid phenotype in Oncidium hybrid orchid. SpringerPlus.

[38] Kim S.H., Jeong J.C., Park S., Bae J.Y., Ahn M.J., Lee H.S., Kwak S.S. (2014). Down-regulation of sweetpotato lycopene β-cyclase gene enhances tolerance to abiotic stress in transgenic calli. Mol. Biol. Rep..

[39] Pogson B.J., Rissler H.M. (2000). Genetic manipulation of carotenoid biosynthesis and photoprotection. Philos. Trans. R. Soc. Lond. B.

[40] De Azevedo Neto A.D., Prisco J.T., Enéas-Filho J., Braga de Abreu C.E., Gomes-Filho E. (2006). Effect of salt stress on antioxidative enzymes and lipid peroxidation in leaves and roots of salt-tolerant and salt-sensitive maize genotypes. Environ. Exp. Bot..

[41] Bode S., Quentmeier C.C., Liao P.N., Barrosc T., Walla P.J. (2008). Xanthophyll-cycle dependence of the energy transfer between carotenoid dark states and chlorophylls in NPQ mutants of living plants and in LHC II. Chem. Phys. Lett..

[42] Demmig-Adams B., Adams W.W. (2002). Antioxidants in photosynthesis and human nutrition. Science.

[43] Parvaiz A., Satyawati S. (2008). Salt stress and phyto-biochemical responses of plants-a review. Plant Soil Environ..

[44] Jithesh M.N., Prashanth S.R., Sivaprakash K.R., Parida A.K. (2006). Antioxidative response mechanisms in halophytes: Their role in stress defense. J. Genet..

[45] Kim S.H., Ahn Y.O., Ahn M.J., Lee H.S., Kwak S.S. (2012). Down-regulation of β-carotene hydroxylase increases β-carotene and total carotenoids enhancing salt stress tolerance in transgenic cultured cells of sweet potato. Phytochemistry.

[46] Shi Y.M., Wang R., Luo Z.P., Jin L.F., Liu P.P., Chen Q.S., Li Z.F., Li F., Wei C.Y., Wu M.Z. (2014). Molecular cloning and functional characterization of the lycopene ε-Cyclase gene via virus-induced gene silencing and its expression pattern in *Nicotiana tabacum*. Int. J. Mol. Sci..

[47] Shi Y.M., Liu P.P., Xia Y.Z., Wei P., Li W.Z., Zhang W., Chen X., Cao P.J., Xu Y.L., Jin L.F. (2015). Downregulation of the *lycopene ε-cyclase* gene confers tolerance to salt and drought stress in *Nicotiana tabacum*. Acta Physiol. Plant.

[48] Kim S.H., Kim Y.H., Ahn Y.O., Ahn M.J., Jeong J.C., Lee H.S., Kwak S.S. (2012). Down-regulation of the *lycopene-ε-cyclase* gene increases carotenoid synthesis via the β-branch-specific pathway and enhances salt-stress tolerance in sweetpotato transgenic calli. Physiol. Plant.

[49] Zhu J.K. (2002). Salt and drought stress signal transduction in plants. Annu. Rev. Plant Biol..

[50] Shinozaki K., Yamaguchi-Shinozaki K. (1997). Gene expression and signal transduction in water-stress response. Plant Physiol..

[51] Pang T., Ye C.Y., Xia X.L., Yin W.L. (2013). De novo sequencing and transcriptome analysis of the desert shrub, *Ammopiptanthus mongolicus*, during cold acclimation using Illumina/Solexa. BMC Genom..

[52] Horsch R.B., Fry J.E., Hoffmann N.L., Eicholtz D., Rogers S.G., Fraley R.T. (1985). A simple method for transferring genes into plants. Science.

[53] Livak K.J., Schmittgen T.D. (2001). Analysis of relative gene expression data using real-time quantitative PCR and the 2^−ΔΔ*C*t^ Method. Methods.

[54] Boyer J.S. (1969). Measurement of the water status of plants. Annu. Rev. Plant Physiol..

[55] Zhang X., Wang L., Meng H., Wen H.T., Fan Y.L., Zhao J. (2011). Maize ABP9 enhances tolerance to multiple stresses in transgenic Arabidopsis by modulating ABA signaling and cellular levels of reactive oxygen species. Plant Mol. Biol..

[56] Wang J., Sun P.P., Chen C.L., Wang Y., Fu X.Z., Liu J.H. (2011). An arginine decarboxylase gene PtADC from Poncirus trifoliata confers abiotic stress tolerance and promotes primary root growth in Arabidopsis. J. Exp. Bot..

[57] Liu H.B., Li X.H., Xiao J.H., Wang S.P. (2012). A convenient method for simultaneous quantification of multiple phytohormones and metabolites: Application in study of rice-bacterium interaction. Plant Methods.

