# Supplementary material for: Cloning of the Lycopene β-cyclase Gene in Nicotiana tabacum and Its Overexpression Confers Salt and Drought Tolerance
Source: Int J Mol Sci. 2015 Dec 21;16(12):30438–57. doi: 10.3390/ijms161226243 (PMC4691183; doi:10.3390/ijms161226243)
Supplement: Supplementary file 1 [file ijms-16-26243-s001.pdf]

Supplementary Materials: Cloning of the *Lycopene β-cyclase* Gene in *Nicotiana tabacum* and Its Overexpression Confers Salt and Drought Tolerance

Yanmei Shi, Jinggong Guo, Wei Zhang, Lifeng Jin, Pingping Liu, Xia Chen, Feng Li, Pan Wei, Zefeng Li, Wenzheng Li, Chunyang Wei, Qingxia Zheng, Qiansi Chen, Jianfeng Zhang, Fucheng Lin, Lingbo Qu, John Hugh Snyder and Ran Wang

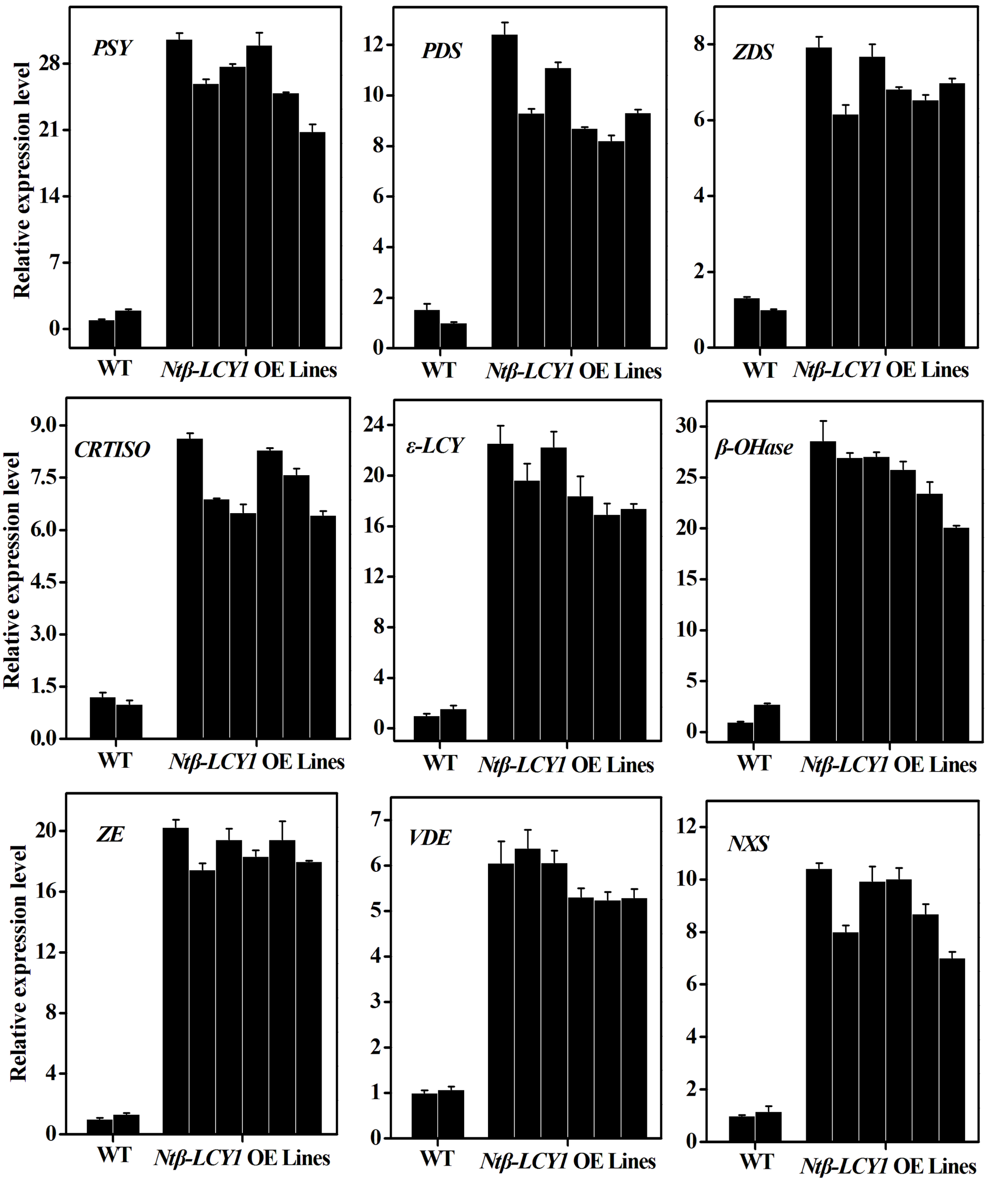

Figure S1. Gene relative expression levels of genes both up- and down-stream of the *Ntβ-LCY* branch point in the carotenoid biosynthetic pathway in WT and *Ntβ-LCY1* OE transgenic lines. *PSY* (*phytoene synthase*), *PDS* (*phytoene desaturase*), *ZDS* (*ζ-carotene desaturase*), *CRTISO* (*carotenoid isomerase*), *β-LCY* (*β-lycopene cyclase*), *β-OHase* (*β-carotene hydroxylase*), *ZE* (*zeaxanthin epoxidase*), *VDE* (*violaxanthin deepoxidase*), and *NXS* (*neoxanthin synthase*). Error bars represent standard deviation (n = 3). The data presented here are representative of three independent experiments.

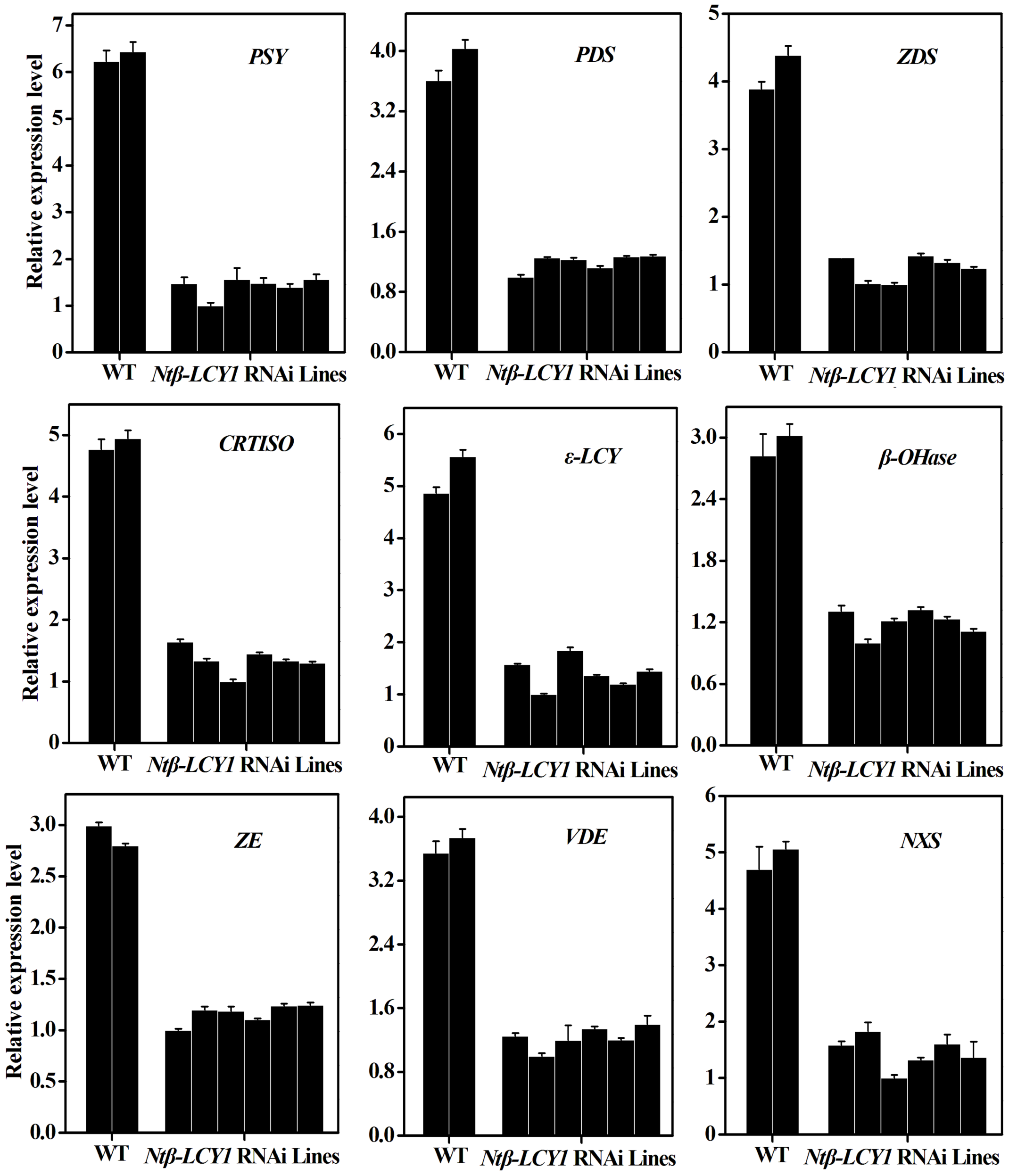

Figure S2. Gene relative expression levels of genes both up- and down-stream of the *Ntβ-LCY* branch point in the carotenoid biosynthetic pathway in WT and *Ntβ-LCY1*RNAi transgenic lines (*26S RNA* as the internal reference gene). Error bars represent standard deviation (*n* = 3). The data presented here are representative of three independent experiments.

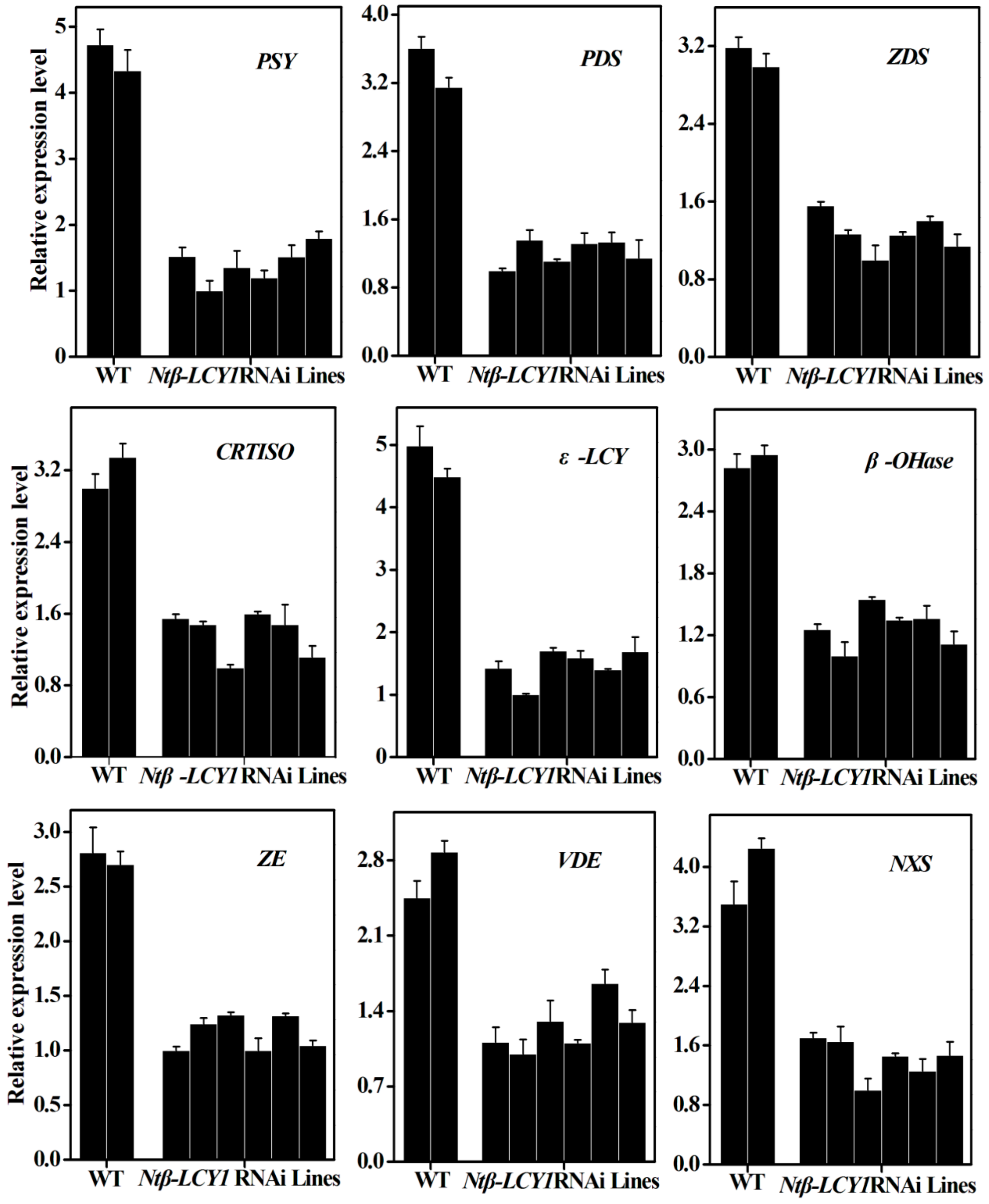

Figure S3. Gene relative expression levels of genes both up- and down-stream of the *Ntβ-LCY* branch point in the carotenoid biosynthetic pathway in WT and *Ntβ-LCY1*RNAi transgenic lines (*L25* as the internal reference gene). Error bars represent standard deviation (n = 3). The data presented here are representative of three independent experiments.

Table S1. Gene primers used for cloning and transgenic confirmation of plant.

Primer Name	Primer Sequence
β-LCY1-F	ATGGATACATTGTTGAAAACCCCAAATAAG
β-LCY1-R	TTCTGTATCCTGTAACAAATTGTTGATCAT
β-LCY1-OE-F	GCACTAGTATGGATACATTGTTGAAAACCCCAAATAAG
β-LCY1-OE-R	TAGGTACCTTCTGTATCCTGTAACAAATTGTTGATCAT
β-LCY1-RNAi-attB-F	GGGGACAAGTTTGTACAAAAAAGCAGGCTTCACGAGGAAGCTAAATCTATGC
β-LCY1-RNAi-attB-R	GGGGACCACTTTGTACAAGAAAGCTGGGTATACGTAATCCAGGACGAGCAAC
nptII-F	TCGCCGCCAAGCTCTTCAGCAAT
nptII-R	GTGGAGAGGCTATTCGGCTATGACT
Hyg-F	GTGCTTTCAGCTTCGATG
Hyg-R	AACCAAGCTCTGATAGAG
β-LCY1-flanking-F	CGGTATGGATATTCTTCTGAAGCTTG
Flag-R	CTTATCGTCATCGTCCTTGTAATCG

Table S2. Gene-specific primers used for qRT-PCR analysis.

Gene	Primer	Primer Sequence	Amplification Efficiency (%)	Primer Origin
PSY	PSY-Q-F<br>PSY-Q-R	TGTTGGAGAAGATGCCAGAAGAG<br>ATAAGCAATAGGTAAGGAAATTAGCTTC	95.4	[46]
PDS	PDS-Q-F<br>PDS-Q-R	ATAAACCCTGACGAGCTTTC<br>AATATGTTCAACAATCGGCAT	97.5	
ZDS	ZDS-Q-R<br>ZDS-Q-F	TGAAATAGGGGAGCTTGATTTCCGC<br>GAGCATATGCGACAGGATCCCAC	102.3	
CRTISO	CRTISO-Q-F<br>CRTISO-Q-R	CGTGTACACCGAGAATATGATG<br>GTAGGCGAGAGTCAAGCACTC	94.7	
ε-*LCY*	ε-LCY-Q-F<br>ε-LCY-Q-R	CAGGAGTCTTTTTCGAGGAAACTTG<br>GTGTTCCAAGCTTGAGTTGAGAT	96.5	
β-*OHase*	β-OHase-Q-F<br>β-OHase-Q-R	ATGGCCGCCAGCAGAATTTC<br>CTCAATTTTCATTTCAATCTCCTCTGTC	95.2	
VDE	VDE-Q-F<br>VDE-Q-R	ATGATGCATGGGATGGATATG<br>CGTTGGAGCTCTTTAAAACCTTC	97.1	
ZE	ZE-Q-F<br>ZE-Q-R	GTGGTGGGATTGGAGGGTTAGTG<br>AGGATCTGCTGCAAAGTCATGC	92.3	
NXS	NXS-Q-F<br>NXS-Q-R	GCCGGGCTCTATTCGACGTGAT<br>ACTGACTCTACCATATGGTCTTCCCAAAT	93.8	
26S-RNA	26S-RNA-Q-F<br>26S-RNA-Q-R	GAAGAAGGTCCCAAGGGTTC<br>TCTCCCTTTAACACCAACGG	99.7	
β-*LCY1*	β-LCY1-Q-F<br>β-LCY1-Q-R	GCCTGTATCTTCCTGAGCTTATATTTTTC<br>TCAGAAAATGGAAATAGTTACTATTGCAAT	97.3	This work
β-*LCY2*	β-LCY2-Q-F<br>β-LCY2-Q-R	GCTGTATCTTCCCGAGCTTATATTTTTG<br>CAGAAATAGAAAATCAAAAAATGGAAATAG	95.6	
L25	L25-Q-F<br>L25-Q-R	CCCCTCACCACAGAGTCTGC<br>AAGGGTGTTGTTGTCCTCAATCTT	96.6	

The full-length and coding sequences of gene in the carotenoid biosynthetic pathway.

>PSY1_Ntab0523090

ATGTCTGTTGCTTTGTTGTGGGTTGTTTCTCCCACTTCCGAGGTCTCGAATGGGACAGG
ATTGTTGGATTCAGTCCGAGAAGGAAACCGCGTCTTTGTATCATCCAGGTTCCTAGCT
CGAGATAGGAATTTGATGTGGAATGGGAGAATCAAGAAAGGTGGGAGACAAAGGTG
GAATTTTGGCTCTTTAATTGCTGATCCAAGATATTCATGCTTGGGTGGATCAAGAACT
GAAAAGGGAAGCAGTTTCTCTGTACAGTCCAGTTTGGTGGCTAGCCCAGCTGGAGAA
ATGACAGTGTCATCAGAGAAAAAGGTCTATGATGTGGTATTGAAGCAAGCAGCTTTA
GTGAAGAGGCAGCTGAGATCTACCGATGAATTAGAAGTGAAACCTGATATAGTTGTT
CCAGGGAATTTGGGCTTGTTGAGTGAAGCATATGATCGTTGTGGCGAAGTATGTGCAG
AGTATGCAAAGACATTTTACTTAGGTCAGTCTCAGCCTCTTTTATCTTTAGTTTACAAA
TTCTTGATTAAGGGACCAATTGATGAAAACATAATCTGATCTTTATTTGGTTCTTATTC
AGGAACAAAGCTAATGACTCCAGAGAGAAGAAGAGCTATCTGGGCAATATATGGTG
TGTATATCTGTGCTTAACTGGGGACATAAAAGGGAATATTATAGGTGCATGTGAGCTC
TACTAGTTTATGAGGCATTATTATATGCATAAAGAGAATGATCTAATTTCTTATACCAT
ATACAACAGTCAAAATGAGTGGAAGAAGTGCAAAAATTATAAGACTGGATCCAGGG
AAGAGTAACATGCTTTTACTGTTAGTTTTATCAAACTTTCTTGGGAATCTTTGACATAA
TTTCTACTTTTGGCTGGCCGGAGATTACGCTTTGTGTAAAAAAATCTTATGTTCTTTAT
ACAATCCATAAGGGTAAAGAGAGTAGAATTCAGATCATTTAAAACTGGTGATCTGAT
TGTTTCTCTTTCCCATTTTGTTTGGCAATCTTCAAAATGTGGTCACACTTTGTGAATAGT
CATTTATGGTAAACTCTTGATGAGATTGACAGCAATTTTGTATAAAGAAATTTTCACC
TGAAGAAAGCTTCTTGTTGGGTTGTTTTTAAACAGTGTGGTGCAGGAGAACGGATGAG
CTAGTCGATGGCCCTAACGCATCACACATAACTCCACAAGCTTTAGACAGGTGGGAG
GCCAGGCTGGAAGATATTTTCAGTGGGCGGCCATTTGATATGCTTGATGCTGCTTTAT
CCGATACTGTCTCCAGATTTCCTGTTGATATTCAGGTTTAGTATTTCTTTTGATCATCTT
TATCTTCTCCATCTAATTTGATTATTAGGAACTAGTTTACCTTTACTGTCCTTGGCATAT
ATGTGAGGATTCAGATTGTGTTCTTATTCTGTATTCATCAGTCTTCGATAACCCCTCTA
TTCTATGTGTGTTGAAAGCCTAAATACTATGCTAGTTTTGTTTCAATAACCTCTTTTATA
TCCTTCAGAAGGGTTCATATTGCATTTGAAGTCATAAAAAACTTGTTTCTTTAGCCCAC
ATCTTTAGGTTACTGTATTGGGAAATATGGTCTTCATTCACATAATTTAAGGAAGTTGT
TTGCTCGCTATCTAATTCTTCATCTACCTTGGTGCAGCCATTCAGAGATATGATAGAA
GGAATGCGTATGGACTTGTGGAAATCCAGATATAACAACTTCGATGAGCTATATCTCT
ATTGTTATTATGTTGCTGGTACAGTAGGACTGATGAGTGTTCCAGTTATGGGTATTGCA
CCTGAATCAAAGGCAACAACAGAGAGTGTATATAATGCTGCTTTGGCTTTAGGGCTT
GCAAATCAACTAACCAATATACTCAGAGATGTAGGAGAAGAGTAAGCATGACGCTA
CATTTAATGCACATTGAACATGCCCAAAAAAGGTCAAAAGAGTTAGGAAAAACTCA
GTGGCTAATCATCTCTTAGTATAGCTACATTAAGTACTTAAAACTTAGTTCTATTCTTA
CATATTTCCTTTTGTTATACAGTGCCAGAAGAGGACGAGTATACTTACCTCAAGATGA
ATTAGCACAGGCAGGGCTTTCTGATGAAGATATATTTGCTGGAAGAGTGACCGATAA
GTGGAGGAACTTTATGAAGAAACAAATTCAGAGGGCGAGGAAATTCTTTGATGAGTC
AGAGAAAGGTGTCACAGAACTGGACTCTGCTAGTAGATGGCCTGTAAGTAGTCTTAA
GATTACAATTGATTGCTTGTGTCCAGTAGTTTTTCTTCCTATTGTTTTACCTGCTAGGAG
ATATGAACCAAGTTTTCTATCGAAAAACCATTAACTGGTAGGCATTGATAGGTATCAT

AGTATCCAATTTTCAACAGCATTCTCTTTTGGTGATGTATCATGCTTCTTTAAATCCTTT
AATCGTGCTCAAATCTTCTCTATTATGTTCTGCTTATATTCCTACACCACAGAGGAAAG
AAAGCTTGATCGGCGTACTTATGCATTGATGGTTTAATGTTGCATTGAGCTTACATTCT
GAATCTATATTCTACTACTTAGTATGGCAATGAGCTGATTTTTTTGTCGTTGCAGGTGT
TAGCAGCGCTGCTGTTGTATCGCAAGATATTGGACGAGATTGAAGCCAATGACTACA
ATAACTTCACAAGGAGGGCTTATGTTAGCAAGCCAAAGAAGCTTCTCACCTTGCCCA
TTGCTTATGCAAAATCTCTTGTGCCCCCTAATAGAACTTCCTCTCCACTAGCAAAAAC
ATGA

>mRNA_108630_cds

ATGAGCATGTCTGTTGCTTTGTTGTGGGTTGTTTCTCCCACTTCCGAGGTCTCGAATGG
GACAGGATTGTTGGATTCAGTCCGAGAAGGAAACCGCGTCTTTGTATCATCCAGGTTC
CTAGCTCGAGATAGGAATTTGATGTGGAATGGGAGAATCAAGAAAGGTGGGAGACA
AAGGTGGAATTTTGGCTCTTTAATTGCTGATCCAAGATATTCATGCTTGGGTGGATCA
AGAACTGAAAAGGGAAGCAGTTTCTCTGTACAGTCCAGTTTGGTGGCTAGCCCAGCT
GGAGAAATGACAGTGTCATCAGAGAAAAAGGTCTATGATGTGGTATTGAAGCAAGC
AGCTTTAGTGAAGAGGCAGCTGAGATCTACCGATGAATTAGAAGTGAAACCTGATAT
AGTTGTTCCAGGGAATTTGGGCTTGTTGAGTGAAGCATATGATCGTTGTGGCGAAGTA
TGTGCAGAGTATGCAAAGACATTTTACTTAGGAACAAAGCTAATGACTCCAGAGAGA
AGAAGAGCTATCTGGGCAATATATGTGTGGTGCAGGAGAACGGATGAGCTAGTCGAT
GGCCCTAACGCATCACACATAACTCCACAAGCTTTAGACAGGTGGGAGGCCAGGCTG
GAAGATATTTTCAGTGGGCGGCCATTTGATATGCTTGATGCTGCTTTATCCGATACTGT
CTCCAGATTTCCTGTTGATATTCAGCCATTCAGAGATATGATAGAAGGAATGCGTATG
GACTTGTGGAAATCCAGATATAACAACTTCGATGAGCTATATCTCTATTGTTATTATGT
TGCTGGTACAGTAGGACTGATGAGTGTTCCAGTTATGGGTATTGCACCTGAATCAAAG
GCAACAACAGAGAGTGTATATAATGCTGCTTTGGCTTTAGGGCTTGCAAATCAACTA
ACCAATATACTCAGAGATGTAGGAGAAGATGCCAGAAGAGGACGAGTATACTTACC
TCAAGATGAATTAGCACAGGCAGGGCTTTCTGATGAAGATATATTTGCTGGAAGAGT
GACCGATAAGTGGAGGAACTTTATGAAGAAACAAATTCAGAGGGCGAGGAAATTCT
TTGATGAGTCAGAGAAAGGTGTCACAGAACTGGACTCTGCTAGTAGATGGCCTGTGT
TAGCAGCGCTGCTGTTGTATCGCAAGATATTGGACGAGATTGAAGCCAATGACTACA
ATAACTTCACAAGGAGGGCTTATGTTAGCAAGCCAAAGAAGCTTCTCACCTTGCCCA
TTGCTTATGCAAAATCTCTTGTGCCCCCTAATAGAACTTCCTCTCCACTAGCAAAAAC
ATGA

>PSY2_Ntab0141080

ATGTCTGTTGCCTTGTTATGGGTTGTTTCACCTTGTGAAGTCTCAAATGGGACAGGATT
CTTGGATTCAGTCCGGGAGGGAAACCGGGTTTTTGATTCGTCGAGGCATAGGAATTTA
GTGTGCAATGAGAGAAACAAGAGAGGTGTGAAACAAAGGTGGAATTTTGGTTCTGTA
AGGTCTGCTATGGTGGCTACACCGGCGGGAGAAATGGCGACGATGACATCAGAACA
GATGGTTTATGATGTGGTTTTAAAACAAGCAGCTTTAGTGAAGAGGCAGTTGAGATCT
GCTGATGATTTAGAAGTGAAGCCGGAGATCCCTCTCCCCGGGAATTTGAGCTTGTTGA
GTGAAGCATATGATAGGTGTAGTGAAGTATGTGCAGAGTATGCAAAGACATTTTACT
TAGGTTTGCATCTTTAATCTATTTATTATTGGTTTACCAAAATTTTGGTTTTAAGTACTA

ATTCAGGCTATACGCTTGTTTATGTAATCGATGAATGCAAAAATTGATCTTTGTTTGGT
TGCTATTCAGGAACCATGCTAATGACTCCAGAGAGAAGAAGGGCTATTTGGGCAATA
TATGGTGAGGTTTCTAGCCATTTAGTATCACTTATGCACACACACACTCAAAAACATG
ATTAATTGGAGACGAAGAGAACTCAAGTTGTTGCCATGTGACCAGGAGGAGGTCACA
GGTTCGAGGCGTGGAAACAGTCTCTTGCAGAAATGCACTGTAAGGATGTGTACAATA
GATCCTTGTGGTTCGGCCTTTCCTCCGACCCGTGCATAGCGACAACTTAGTGTATCGG
GCTGCCCTTTTTAAAGAGGAGAACTCAAATTTGAGTTTGAGGGGTACGTGTAATAGGT
AAAACTTGAGCTTCACTAGTTTGAAAGGCAATTAAAAAATCTTATTTGTTATATTATTC
CTAATACTAGCCACAAAATTGGAAGGACTTGCTTAATAGTTAGACTCGAGCCATAGA
TGTAGCATGCTTTAACTTCTGGTCATTTCTGTTAGATTTTTAGAGGTCAGATTTGATCTC
TGAATCTTTTTCATAAGAGAGTAATACTGGCGTTATTGTATGATTGTTTTGCAGTGCAA
GTGTGCAACAAGTCTTTCTCTCCAAATTATCAGCTAGTCATGCATGGTCAAGACCACT
TTGAAAAAACACATGATTAATATACATGAAAGTGTTGATTTTGATCTATTATACATCG
TCTTCAATGTTAATAACATATCACTTCTGATCCTCTTTCTTTCTCTTTAACTGTAATCTA
ATGGTAATTGTTGGAGCTTGGGTTGCTGCATATATAAACGAATTTCTGAGTTGAAAAG
TTTCTCATTAAGTTCTTTTAAACAGTGTGGTGCAGGAGAACAGATGAACTTGTTGATG
GCCCAAACGCATCACATATTACACCCCAAGCCTTAGATAGGTGGGAAGACCGGCTTG
AAGATGTTTTCAGCGGGCGACCATTTGATATGCTCGATGCTGCTTTGTCCGATACTGTT
TCCAAGTTTCCAGTTGATATTCAGGTTAGTCTTCCAATTCTATCATTTTTATCTTTCTCT
GGTTCAGAGTTCTATGTCGCTTTCTTCATTCACATAAGTTGATTCACTTACGTCAATCA
TGTAAACTGTCTCATTGTTCAATATATCTTGCAGCCGTTCAGAGATATGATTGAAGGA
ATGCGTATGGACTTGAGGAAGTCAAGATATAGAAACTTTGATGAGCTTTACCTCTATT
GTTATTACGTTGCTGGTACGGTTGGGTTGATGAGTGTTCCAATTATGGGTATTGCACCT
GATTCAAAGGCAACAACAGAGAGCGTATATAATGCAGCTTTGGCTTTAGGAATCGCA
AATCAACTAACGAACATACTCAGAGATGTTGGAGAAGAGTAAGTACAAGCCTACATT
TTTACGCACATTCAGTTTGCTACTATGACATACCGATAATGTTAGTTGAGCTGTTTAGT
TATCAGGTTTATGTTGTGGTTATGTCAATATAATACTAGAATAATTCGGAAAACTGCA
GATAGGGAANNNNNNNNNNNNNNAGGGAAAGAAAACTCTTCATAAGTGAAGTGC
GATATTTTTATTGGTTAAGGCTTCCCTTGTTCTTATATATTGCTTTCCCCGCAGTGCCAG
AAGAGGAAGAGTCTACTTACCTCAAGATGAATTAGCACAGGCAGGTCTCTTCGACGA
TGACATATTTGCTGGAAAAGTGACTGATAAGTGGAGAAGCTTTATGAAGAAGCAAAT
CCAGAGGGCAAGAAAGTTCTTCGATGAGGCAGAGGAAGGAGTTACACAACTGAGCT
CAGCTAGCAGATGGCCTGTAAGCAGTCGTAAACTCTTAGCTTAGCTATATGAATATGA
TTTCTGGTCTGAGTATAATCTTTTTTCACCTTTTTTGGCTGTATGGAATATGGTAGGGTT
AAAAAAAAGAAGGAAATATATCAAAATATTCGGACATTAATGATCCATTCAGTTGTA
TTTCTTGGTCAATGAAATATCCGGATTTGGATAATTTTAGTGGTTCCATCAGCCAGTTT
ATCTGCTACCTAAACTTAGATGGCTGAGACAACATTGTTCCCAAATTTACACCAGTAT
TCTCTTCGCCAGATGTAAGATTCGCCTTAAATACTGTTGTGCCAAAATCTTCTCTATGA
TCCTCGACTTGCTTCACTTCCTAGGAAAAGAACAGCTTTGTCTTTGTATGTCTTGATAA
TTTCATTGCATAGTTTAGGTAGAGGCACCAGAAACAGCCTTTATACCTTCTCAAGATG
GGGGTAAGGACTGCGTACACACTACCCTCCCCATACCTCACTTGTAGGACTACGTTA
GGTTGTTTGTTGTTGTCGTTGTTATTGTAGTTTAGGTAGAGGCAAAGGCCTATACTTTT
ATAGGACACTGAGCGAGAAACATCTCTTTGTTCCTTTTGGTTTATTCACTGATCTGAAA
CTTAGCACTAGTTTTCTTATTGATTCCTGAGGTGCTGACATTCCTGAATGAAGTCTATC

TGAACCATATTAGCACTTCTCATGATCTCGGCTTTTGCAGGTATGGGCATCTTTGCTGT
TGTACCGCCAAATACTGGACGAGATTGAAGCCAATGACTACAACAACTTCACAAAG
AGAGCTTATGTGAGCAAACCAAAGAAGCTAATTTCCTTACCTATTGCTTATGCAAAAT
CTCTTGTGCCCCCTACAAGAACTCTTGTCACCTCTAGCTAA

>mRNA_24759_cds

ATGCTCGATGCTGCTTTGTCCGATACTGTTTCCAAGTTTCCAGTTGATATTCAGCCGTT
CAGAGATATGATTGAAGGAATGCGTATGGACTTGAGGAAGTCAAGATATAGAAACTT
TGATGAGCTTTACCTCTATTGTTATTACGTTGCTGGTACGGTTGGGTTGATGAGTGTTC
CAATTATGGGTATTGCACCTGATTCAAAGGCAACAACAGAGAGCGTATATAATGCAG
CTTTGGCTTTAGGAATCGCAAATCAACTAACGAACATACTCAGAGATGTTGGAGAAG
ATGCCAGAAGAGGAAGAGTCTACTTACCTCAAGATGAATTAGCACAGGCAGGTCTCT
TCGACGATGACATATTTGCTGGAAAAGTGACTGATAAGTGGAGAAGCTTTATGAAGA
AGCAAATCCAGAGGGCAAGAAAGTTCTTCGATGAGGCAGAGGAAGGAGTTACACAA
CTGAGCTCAGCTAGCAGATGGCCTGTATGGGCATCTTTGCTGTTGTACCGCCAAATAC
TGGACGAGATTGAAGCCAATGACTACAACAACTTCACAAAGAGAGCTTATGTGAGC
AAACCAAAGAAGCTAATTTCCTTACCTATTGCTTATGCAAAATCTCTTGTGCCCCCTA
CAAGAACTCTTGTCACCTCTAGCTAA

>PSY3_Ntab0582610

ATGTCTGTTGCCTTGTTATGGGTTGTTTCACCTTGTGAGGTCTCAAATGGGACAGGATT
CTTGGATTCAGTAAGGGAGGGAAACCGGGTTTTTGACTCGTCGAGGCATAGGAATTT
AGTGTGCAATGAGAGAATCAAAAGAGGTGTGAAACAAAGGTGGAATTTTGGTTCTGT
ACGGTCTGCGATGGTGGCTACACCAACGGGAGAAATGGCGACAATGACATCAGAAC
AGAAGGTTTATGATGTGGTATTGAAACAAGCAGCTTTAGTGAAAAGGCAGCTGAGAT
CTACTGATGATTTAGAAGTGAAGCCGGAGATCCCTCTCCCCGGGAATTTGAGCTTGTT
AAGTGAAGCATATGATAGGTGTAGTGAAGTATGCGCAGAGTATGCAAAGACATTTTA
CTTAGGTTACCTTTTCATCTATTTATTGGTTTACCAAAATATTGGTTTAAGTACTAATTA
AGACTATATGCCTGTTTATGTAATCGATGAATTCAAAAATTGATCTTGTTTTTGTTTGTT
ATTCAGGAACTATGCTAATGACTCCAGAGAGAAGAAGGGCTATTTGGGCAATATATG
GTGAGGTTTCTAGCCATTTAGTATCAGATACACAGACACACACACGCGCGATTAATT
GGAGACAAAGAGAACTCAAATTTGAGTTTGAGGGGTACCTGTAATATGTAAAACTTG
AGCTTTGAAAGGCAATATAAGAATTCAAATCTGTAGTATCAGATGTAGTATGCTTTAA
CTTTTTGACGGAATATTTATCATAAGAGACTTCTGGTCGTTTCTGTAAAATTTTTAGAA
GTTAGATTTGAATCTCTGAATCTTTTTCATAAGAGAGTAATACTGGCGTTACTGTGAG
ATGATTTTAAAGTGAGACAAATCTTTCTCTCCAAATTATTAGCTAGTCTTGCATTGTCA
AGACCACTTTGAAAAAGCACATGTTACAGATATTAATGAAAGTGTTGATATTGCATAT
ATAAACGAACTTCTTAGTTGAAAAGTTTCTCATTATGTTCTTTTAAACAGTATGGTGCA
GGAGAACAGATGAACTTGTTGATGGCCCGAATGCATCACATATTACTCCACAAGCCT
TAGATAGGTGGGAAGACCGGCTGGAAGATGTTTTCAGTGGGCGGCCATTTGATATGC
TCGATGCTGCTTTGTCCGATACTGTTTCCCAGTTTCCAGTTGATATTCAGGTTTGTCTTC
CAATTCTATCATTTTTATCTTTCTCTGATTCAGAGTTCTATGTCGCTTTCTTCATTCACAT
AAGTTGATTCACTTGGTTCAATCATGTAAACCGTCTCATTGTTCAATCGACGTTGCAG
CCGTTCAGAGATATGATTGAAGGAATGCGTATGGACTTGAGGAAGTCAAGATACAGA

AACTTTGATGAGCTATACCTATATTGTTATTACGTTGCTGGTACGGTTGGGTTGATGAG
TGTTCCAATTATGGGTATTGCACCTGATTCAAAGGCAACAACAGAGAGTGTATATAAT
GCAGCTTTGGCTTTAGGGATCGCAAATCAACTAACCAACATACTCAGAGATGTCGGA
GAAGAGTAAGTACAAACCTACAATTTTACGCACGTCCAATTTGCTACTATGACATAC
CAAAAGATGTTAGTTGAGCTGTTTATCAGGTTTGTTGTTGCTATGTCAACATAATATTG
GGGAAAGAAAGCTCTTCGTAAGTGAAGTGCGATATCTTTTTGGTTAAGGCTTCCCTTG
TTCTTATATATTGCTTTCGTTGCAGTGCCAGAAGAGGAAGAGTCTACTTACCTCAAGA
TGAGTTAGCACAGGCAGGTCTCTCCGACAATGACATTTTTGCTGGAAAAGTGACTGAT
AAATGGAGAAGCTTTATGAAGAAGCAAATCCAGAGGGCAAGAAAATTCTTCGACGA
GGCAGAGGAAGGAGTGACACAACTGAGCTCAGCTAGTAGATGGCCTGTAAGCAGGC
ATAAACTCTTAGTTATATGAATATGATTTCTTGTTTGAGTAGATTCTTTTTTCAACTTTT
TTGGCTGCATGGAATATGGTAGGGTTAAAAAAAAGAAGGCAATATATCAAAATATTT
GGACATTAACGATCCATTCCGTTGTATTTCTTGGTCAATGAAATATCCGGATTTGATTG
AAGTCATTCCATCAGCCATGTTATCTGCTACCGAAACTTAGATGATTGAGAGACCTGA
TATTATTTTAGTTTGAATTAGAAGTAGTTGTACTAGTATGCTTGGAAAGACAACATTGT
TCCCAAATTTACACCAGTATTCTCTTCGCCTCTTCGCCAGAAGTAAGATTCGCCTTGG
ATACTGTTGCGCCAAAATCTTCTCTATGATCCTCTATTTGCTTTTCACTTCCTAGGAAA
AGAACAGCTTTATCTTCGTATGTCTTGATAATTTCATTGCATAGTTTAGGTAGAGGCAC
TGGAAACAACCACTCTACCTTCTTAAGGTAGGGGTAAGGATTGCGTACACACTACCC
TCCCCAGATCTCACTTGTGGGACTACACTTGGTTTATAGTTGTTGTCGTTGTTGTTGTA
GTTTAGGTAGAGGCAAAGACCTATACTTTTATTGGGCAATGAGCGAGAAACGTCTCTT
CTTTCCTTTTAGTTTATTCAGTGATCTGAAACTTAGCACTTGTTTTCTTATTGATTCCCG
GGGTGGTGACATACTTGAATGAAGTCTATCCGAACCAAATTAACACTTCTCATGATCT
CGGCTTTTGCAGGTATGGGCATCTTTGCTGTTGTACCGCCAGATACTCGACGAGATTG
AAGCCAATGACTACAACAACTTCACAAAGAGAGCTTATGTGAGCAAACCAAAGAAG
CTAATTTCCTTACCTATTGCTTATGCAAAATCTCTTGTGCCCCCTACAAGAACTCTTGT
CACCTCTAGCTAA

>mRNA_28820_cds

ATGTCTGTTGCCTTGTTATGGGTTGTTTCACCTTGTGAGGTCTCAAATGGGACAGGATT
CTTGGATTCAGTAAGGGAGGGAAACCGGGTTTTTGACTCGTCGAGGCATAGGAATTT
AGTGTGCAATGAGAGAATCAAAAGAGGTGTGAAACAAAGGTGGAATTTTGGTTCTGT
ACGGTCTGCGATGGTGGCTACACCAACGGGAGAAATGGCGACAATGACATCAGAAC
AGAAGGTTTATGATGTGGTATTGAAACAAGCAGCTTTAGTGAAAAGGCAGCTGAGAT
CTACTGATGATTTAGAAGTGAAGCCGGAGATCCCTCTCCCCGGGAATTTGAGCTTGTT
AAGTGAAGCATATGATAGGTGTAGTGAAGTATGCGCAGAGTATGCAAAGACATTTTA
CTTAGGAACTATGCTAATGACTCCAGAGAGAAGAAGGGCTATTTGGGCAATATATGT
ATGGTGCAGGAGAACAGATGAACTTGTTGATGGCCCGAATGCATCACATATTACTCC
ACAAGCCTTAGATAGGTGGGAAGACCGGCTGGAAGATGTTTTCAGTGGGCGGCCATT
TGATATGCTCGATGCTGCTTTGTCCGATACTGTTTCCCAGTTTCCAGTTGATATTCAGC
CGTTCAGAGATATGATTGAAGGAATGCGTATGGACTTGAGGAAGTCAAGATACAGAA
ACTTTGATGAGCTATACCTATATTGTTATTACGTTGCTGGTACGGTTGGGTTGATGAGT
GTTCCAATTATGGGTATTGCACCTGATTCAAAGGCAACAACAGAGAGTGTATATAAT
GCAGCTTTGGCTTTAGGGATCGCAAATCAACTAACCAACATACTCAGAGATGTCGGA

GAAGATGCCAGAAGAGGAAGAGTCTACTTACCTCAAGATGAGTTAGCACAGGCAGG
TCTCTCCGACAATGACATTTTTGCTGGAAAAGTGACTGATAAATGGAGAAGCTTTATG
AAGAAGCAAATCCAGAGGGCAAGAAAATTCTTCGACGAGGCAGAGGAAGGAGTGA
CACAACTGAGCTCAGCTAGTAGATGGCCTGTATGGGCATCTTTGCTGTTGTACCGCCA
GATACTCGACGAGATTGAAGCCAATGACTACAACAACTTCACAAGGAGAGCTTATGT
GAGCAAACCAAAGAAGCTAATTTCCTTACCTATTGCTTATGCAAAATCTCTTGTGCCC
CCTACAAGAACTCTTGTCACCTCTAGCTAA

>PSY4_Ntab0470140

ATGTCTGTTGCTTTGTTGTGGATTGTTTCTCCCAATTCCGAGGTCTCAAATGGGACAGG
ATTCTTGGATTCAGTCCGAGATGGAAACCGCGTCTTTGTATCATCCAGGTTCCTAGCT
CGAGGTAGGAATTTGATGTGGAATGGGAGAATCAAGAAAGGCGGGAGACGACAAA
GGTGGAATTTTGGCTCTTTAATTGCAGATTCAAGATATGCATGCTTGGGAGGATCAAG
AACTGAAAATGGAAGCACTTTCTCTGTACAGTCCAGTTTGGTGGCTAGCCCAGCTGGA
GAAATGACTGTGTCATCAGAGAAAAAGGTGTATGATGTGGTATTAAAGCAGGCAGCT
TTAGTGAAGAGGCAGCTGAGATCTACCGATGATTTAGAAGTGAAGCCGGATATTGTT
GTTCCAGGGAATTTGGGCTTGTTGAGTGAAGCATATGATCGTTGTGGCGAAGTATGTG
CAGAGTATGCAAAGACATTTTACTTAGGTCAGTCTCAGCCTCTTTTATCTTTAGTTTAC
AAATTCTTGGTTAAGGGACCAATTGATGAAAACAAAATTTGATCTTTATTTTGGTACT
TATTCAGGAACCAAGCTAATGACCCCAGAGAGAAGAAGAGCTATCTGGGCAATATA
TGGTGTGTATATCTGTGCTTAACTGGGGACATAAAAAGGAATATTATAGGTGCATGTG
CGCTCTACTAGTTTCTGAGGCATTATTATATGCATAAAGAGAAGAGGTCTAATTTCTC
ATACCATATACAAGAGTCAAAATGAGTGGAAGAAGTGCAAAAATTATTAGACAGGA
TTCAGGGAAGTGTAACATGCTTTTACTGTTAGTTTTATCAAACTTTCTTGGGAATCTTT
GACATAATTTCTACTTTTGGCTGGCCGGAGGTTACACTCTTTGTGTAAAAACAATCTTA
TGTTCTTTATACAGTCCAAAAGGGTAAAGAGAGTAGACTTCAGATAATTTAAAACTG
GTGATCTGATTGTTTCTCTTTCCCATTTTGTTTGGCAGTCTTCAAAATATAGTGTCACAC
TTTGTGATATATCTGGTACTTGTAATACTTTCAAATATTCTGCTTCCAAGAAGTACTAA
ATTGACACTTTGTGAATAGTCAATTATGGCAAACTCTTGGTGAATTGACAGCAATTTT
GTATTAGTGCATGTATAAAGAAATTTTCATCTGAAGAAAGCTTCTTGTTGTGTTGTTTT
TAAACAGTGTGGTGCAGGAGAACGGATGAGCTTGTTGATGGCCCTAATGCATCCCAC
ATAACTCCGCAAGCTTTAGATAGGTGGGAGACCAGGCTGGAAGATATTTTCAGTGGG
CGGCCATTTGATATGCTTGATGCTGCTTTATCCGATACTGTCTCCAGATTTCCTGTTGA
TATTCAGGTTACTATTTCTTTTGATCATCTGTATCCTCTCCTGTGTCAGAATTCTACTCT
TTTCTGCAACTACTCCGTCTTTTAAGCATATTTTTCTAGTAATTCCATCAAATTTGATTA
TTAGGAATTCGTTTACCTTTACTGTCATTGGCATATATGTGAGGATTCAGGTTGTGTTC
TTATTCTGTATTCATCAGTCCTCGATAACCCCTCTATCCTATGTGTGTTTTGTGTTCAAT
AACCTCTCTTATATCCTATTTGTGGTTTTAAGATTCAGAAAGGGTTCATGTTGCATTTG
AAGTAATTTACACTTCAAAATAAAACACTTGTCATGATAGCTTCTCTTAGGTTACTGT
ATTCGGAATATGGTCTTCATTCACATAATTCAAGGAAGTCGTTTGCTTGCAGCCATTC
AGAGATATGATTGAAGGAATGCGTATGGACTTGTGGAAATCCAGATACAAAACTTTC
GATGAGCTATATCTCTATTGTTACTATGTTGCTGGTACTGTAGGATTGATGAGTGTTCC
AGTTATGGGTATTGCACCTGAATCAAAGGCAACAACAGAGAGTGTATATAATGCTGC
TTTGGCTTTAGGGCTTGCAAATCAACTAACCAATATACTCAGAGATGTAGGAGAAGA

GTAAGCATGAAACTACATTTAATGCACATTGAACATGCCAAAAAAGGTCAAAAGAG
TTGGGAAAAGCTCTGTGGCTAATCATCTCTTAGTATAGCTACACTAAGTGCTTAAAAT
TTGGTTCTATTCTTACATATTTCCTTTTGTTATACAGTGCCAGAAGAGGAAGAGTATAC
TTGCCTCAAGATGAATTAGCACAGGCAGGGCTCTCCGACGAAGACATATTTGCTGGA
AGAGTGACTGATAAGTGGAGGAACTTTATGAAGAAACAAATTCAGAGGGCGAGGAA
ATTCTTTGATGAGTCAGAGAAAGGTGTCACAGAACTGGACTCTGCTAGTAGATGGCC
TGTAAGTAGTCTTAAGATTACAATTGATCGCTTGTATCCAGTAGTTTTTCTTCCTATTTT
TTTACCTGCTAGCTAGGAGATATGGTTGTGAGAAAAAATCAGTATGAACTCAGTTTTC
TATCGAAAAACCATACACTGATAGGCATTGATAGGTATCATAGTTTCCAATTTTCAAC
AGCATTCTCTTGTGGTGTTGTATCATGCTTGTTAAATCCTCTAGTTGTGCTCATATCTTC
TCTATTATGTTCTGCTTATATTCCTACACCACAGAGGAAAGAGAGCTTGATCGACGTA
CCTATGCATTGATGGTCTAAATTTGCATTGAGCTTATATTCTGAATCTATATTCTACTG
TACTTAGTATGGCATTGAGCTGTTTTTTTTGTCGTTGCAGGTGTTAACAGCGCTGCTGTT
GTATCGCAAGATATTGGACGAGATTGAAGCCAACGACTACAACAACTTCACAAGGA
GGGCTTATGTTAGCAAGCCAAAGAAGCTTCTCACCTTGCCCATTGCTTATGCAAAATC
TCTTGTGCCCCCTAATAGAACTTCCTCTCCACTAGCAAAGACATGA

>mRNA_3350_cds

ATGAGCATGTCTGTTGCTTTGTTGTGGATTGTTTCTCCCAATTCCGAGGTCTCAAATGG
GACAGGATTCTTGGATTCAGTCCGAGATGGAAACCGCGTCTTTGTATCATCCAGGTTC
CTAGCTCGAGGTAGGAATTTGATGTGGAATGGGAGAATCAAGAAAGGCGGGAGACG
ACAAAGGTGGAATTTTGGCTCTTTAATTGCAGATTCAAGATATGCATGCTTGGGAGGA
TCAAGAACTGAAAATGGAAGCACTTTCTCTGTACAGTCCAGTTTGGTGGCTAGCCCA
GCTGGAGAAATGACTGTGTCATCAGAGAAAAAGGTGTATGATGTGGTATTAAAGCAG
GCAGCTTTAGTGAAGAGGCAGCTGAGATCTACCGATGATTTAGAAGTGAAGCCGGAT
ATTGTTGTTCCAGGGAATTTGGGCTTGTTGAGTGAAGCATATGATCGTTGTGGCGAAG
TATGTGCAGAGTATGCAAAGACATTTTACTTAGGAACCAAGCTAATGACCCCAGAGA
GAAGAAGAGCTATCTGGGCAATATATGTGTGGTGCAGGAGAACGGATGAGCTTGTTG
ATGGCCCTAATGCATCCCACATAACTCCGCAAGCTTTAGATAGGTGGGAGACCAGGC
TGGAAGATATTTTCAGTGGGCGGCCATTTGATATGCTTGATGCTGCTTTATCCGATACT
GTCTCCAGATTTCCTGTTGATATTCAGCCATTCAGAGATATGATTGAAGGAATGCGTA
TGGACTTGTGGAAATCCAGATACAAAACTTTCGATGAGCTATATCTCTATTGTTACTA
TGTTGCTGGTACTGTAGGATTGATGAGTGTTCCAGTTATGGGTATTGCACCTGAATCA
AAGGCAACAACAGAGAGTGTATATAATGCTGCTTTGGCTTTAGGGCTTGCAAATCAA
CTAACCAATATACTCAGAGATGTAGGAGAAGATGCCAGAAGAGGAAGAGTATACTT
GCCTCAAGATGAATTAGCACAGGCAGGGCTCTCCGACGAAGACATATTTGCTGGAAG
AGTGACTGATAAGTGGAGGAACTTTATGAAGAAACAAATTCAGAGGGCGAGGAAAT
TCTTTGATGAGTCAGAGAAAGGTGTCACAGAACTGGACTCTGCTAGTAGATGGCCTG
TGTTAACAGCGCTGCTGTTGTATCGCAAGATATTGGACGAGATTGAAGCCAACGACT
ACAACAACTTCACAAGGAGGGCTTATGTTAGCAAGCCAAAGAAGCTTCTCACCTTGC
CCATTGCTTATGCAAAATCTCTTGTGCCCCCTAATAGAACTTCCTCTCCACTAGCAAA
GACATGA

>PDS1_Ntab0746310.1

ATGCCCCAAATTGGACTTGTTTCTGCCGTTAATTTGAGAGTCCAAGGTAATTCAGCTT
ATCTTTGGAGCTCGAGGTCTTCTTTGGGAACTGAAAGTCAAGATGGTCACTTGCAAAG
GAATTTGTTATGTTTTGGTAGTAGCGACTCCATGGGGCATAAGTTAAGGATTCGTACT
CCCAGTGCCATGACCAGAAGATTGACAAAGGACTTTAATCCTTTAAAGGTTTGTTTTG
AATGCGGTGTGATTCTGAATTTATGATCTTGGGCATATATTCTCTAAAATAAGAGAAG
TATATCTTGCCATTCAGGTAGTCTGCATTGATTATCCAAGACCAGAGCTAGACAATAC
AGTTAACTATTTGGAGGCGGCGTTATTATCATCATCATTTCGTACTTCCTCACGCCCAA
CTAAACCATTGGAGATTGTTATTGCTGGTGCAGGTGATTTTTTCCAGTCATCTATATTT
GTAGTCTTCATTTTTCTTTTTTCGGAGGGAAGATCATTCTATTAGTTGTATTATCACTAG
AATATTTACCTGTGCATTCTTTTCTGATTAACTGTTTGTACCGCAAAATTTTAGGTTCT
TAGTTCTTCGCCATTTTGCAACTAATCAGCAAGTCCCAACTGATTAGATTAGGAGCGA
TTTGAAAATTAGTTTGTTTTGAACTATTTTTGCCGTCACTCTTATACTGTTGAGTTGTCC
CACATCGGTGGGATTTGAGGTCCTTGGTCTCACCTCATAAGCTAGCTTTTGAGGTTGA
GTTAGACCCAATGTCCATTTATCATGGCATCCGAGCCAGGCCCATCCCATTTATTGTT
ACCGATATGGGGCTCCCATTTATGTTGTCCACGCTCCAGTTTGCAAGCCTGGGCGTGG
GGGAGGGGGGGTGGGGTGTTGAGTTGTCCCATATCGGTGGGATTTGAGGTCCTTGGTC
TCCTTATATGGTCTTGGACAATCCTCGCCTCATAAGCTAGCTTTTGGGGTTGAGTTAGG
CCCAAGATCCATTTATCATATATATGTCTATTCTCTCTTATCATCTGAGCCATGATAAG
CGGGTGAACGTGCTGTCTATTGGGTGGCATGTCCAATGGATCATTCTGAAATATTGGA
GGCAAATGAACCAATACCTTGTGCAAGATTGATATCACAATACCTATAATCAGAGTA
CTTAGAGTTCCAAAAATTTCAAAACCCATTGAAAAGTCAAACGAGTTACATATAGGG
GTTGCACTCTTCTAAGGCTTGCAATCTGTGAGAAAAAGATGAGAAGGAGATCTTCAT
ATTTCATCTTTACTAGGCTGGACCATTGACCGGTTAGCAGTTTTGAACTTGTTCTTCAA
CTTGGCTTGCATAGTACTGTGCCGATCATTTCTTTTGTATTGTCATCAACTGGTTGATTA
TCTGAGTACCTAAAGAAAGAATGTTATATGCATGATACATTCTGCTGTACTATAAAAG
ATATAATAAAGAATGCTAGCCGAGGTACTACATGGCCTTTTCAGACAAATAGAAGCT
GTAGCATGATTCTAATTCAATTTTTTTTTTTTTTTTTTGGAATATCAGGTTTGGGTGGTTT
GTCTACAGCAAAATATCTGGCTGATGCTGGTCACAAACCGATATTGCTGGAGGCAAG
AGATGTCCTAGGTGGAAAGGTGAAGAATATCCATGCTTTCCTTTAATTTTATTCCTTTT
TCTTTTGTGTCCTTCCCTATTGATAGTCCCTTTTCAGGAAGGCTTCTATTTGTTTTGTTTA
AAATCATTTTTCATACTCTTTAAACATTCAGTTGCTCAAACAATTGCAAGGGTGTTCAC
TATTCCTATTTTTGACTGTCTTCCTTTCTCTCAGTTTAGTTTTATTCCTCTCTCTCTCTCTC
TCTCTATTTTTGGAGGAAATAGATCTGTCCTAAAAATTTCCAGCTTTACTACTAATAGT
GTTAATTGTCGAGAAAATAGTACAGCATATTAGGTAAAAGATATGGAAAGTATATTA
TTATTATTATTATTATTATTATTATTATTATTATTATTATTATTATTATTATTCTCTATTAT
TTTAAGATTGAGTCAATTTTACCTGTCCTGTTGGTTGCATTTCTCATATAAACATTCTTT
TCTGTGAGATGCTATGTGAATTAGCTGATGTTTTTGGTATAGAGCACTATGTTAGTCAG
TTTTATCTTACTGAAGCAGTCACCAAGAATCTAGTTGTATAGGCTAAAAGATTGAATT
AGCATTAATCTTTATGTGTTCTGCACCTGAATACTTATACCTACCTTTTAGGTAGCTGC
ATGGAAAGATGATGATGGAGATTGGTATGAGACTGGGTTGCACATATTCTGTAAGTTT
GACTCCTCAAGAATGCATACTTTAATCTTCTAGTACAACAGTTTCTTTCAAGATCTCTT
TTGTCCATTAATCAGATAGCTATCCCTGTTTGTCTTTTGCAAATAGCCAATATGTCAGT
CGATCTGTATTCTGCCTTGCCTATCTTTTTTTATCTGTTAATTTCGTATGGTGACTCATA
CAAGTTGGTGCATCTCCTTTAAGTTGGGGCTTACCCAAATATGCAGAACTTGTTTGGA

GAACTAGGGATAAACGATCGGTTGCAGTGGAAGGAACATTCAATGATATTTGCGATG
CCTAACAAGCCAGGGGAGTTCAGCCGCTTTGATTTTCCTGAAGCTCTTCCTGCGCCAT
TAAATGGTAAGTACTTAATCATGAGTAAATTTCTCCCTTCTGCATTGATTATGTAAACT
TCCCCAATAAGGCATGAAATTGATTAGTCCATTAATACTCTGGCACATTGCTAACATC
AAAAGAACATAAAGGTTCATTACGTCTTGATCAGAATTTCTGCATGTAGCTAAAGTG
ATTGAGTGTCTGTATAGGTTTTTACACATTGCAAGCATAAGCCAGTTATGTTATCTCTT
ATTTTCATTTCTCTACCTGTATCTCTTATTCTCATTTCTCTATCTATGCGTTATTACTTCT
ACAGGAATTTTGGCCATACTAAAGAACAACGAAATGCTTACGTGGCCCGAAAAAGTC
AAATTTGCTATTGGACTCTTGCCAGCAATGCTTGGAGGGCAATCTTATGTTGAAGCTC
AAGACGGTTTAAGTGTTAAGGACTGGATGAGAAAGCAAGTGCGTGATCGTTTTATCTT
ACTCTTTAAAGTTCATAACCTTGAGGACATAGTTGACTTGCATGTTGTTGATTTCACAT
GCTAGAATTGTCTACCTGCCTTTCTTTTTCTAACAACATACATCTTACAAATCTCAGCA
GCAGCTATTTGCTTAATTGCTTTTCAGGGTGTGCCTGATAGGGTGACAGATGAGGTGT
TCATTGCCATGTCAAAGGCACTTAACTTCATAAACCCTGACGAGCTTTCGATGCAGTG
CATTTTGATTGCTTTGAACAGATTTCTTCAGGTTAGAATCCTGATCCACCCTCAAAACA
AAAAGAGAGAAAGGATATAATCCTACCAAAGCTGTAAATCATGTTAGGGACCTGA
CATATTGGTGCAGGAAACTTATTTCTGAACTTTTTCACTCTGTTTAACTTTTCTGATATA
TTTGAATTATTAATCTGCAGGAGAAACATGGTTCAAAAATGGCCTTTTTAGATGGTAA
CCCTCCTGAGAGACTTTGCATGCCGATTGTTGAACATATTGAGTCAAAAGGTGGCCA
AGTCAGACTAAACTCACGAATAAAAAAGATTGAGCTGAATGAGGATGGAAGTGTCA
AATGTTTTATACTGAATAATGGCAGTACAATTAAAGGAGATGCTTTTGTGTTTGCCAC
TCCAGGTATAATATCCATTATACTAGCATCGATGCTTCCAGTTTTCACATTTTTAATAT
GAATTTATAATTTTTTGCTGACTTTTGATTATCCGATTAGTGGATATCTTCAAGCTTCTT
TTGCCTGAAGAGTGGAAAGAGATCCCATATTTCCAAAAGTTGGAGAAGCTAGTGGGA
GTTCCTGTGATAAATGTCCATATATGGTTAGTGATGAAAATTTTACTTTTCAGTGTTTG
TTCTTCCTCTAGCATATCTATGTATGTGCCTGTTAATGTCTATACGTACATGTTTATGTG
GTCCTCCGGTATTCTGTTAACTTCCCTTGAATGAGGAACTTATGGATGTAAGCTTTTCC
CAAACTTTGATTGGACACATTGCAATTGTCTGTTCAACTTTGATGAGCAGAACTACCA
TTGTTTAGCTATTAGTGGCTGAGATTCCTACTGAAAAGATTTGTATAAATTTAATTTGC
AGGTTTGACAGAAAACTGAAGAACACATCTGATAATCTGCTCTTCAGCAGGTTCATTT
TTGATCAATTTTATTGTTCCAGAGCAGTTTCTGCGTGTCCATGACTACATTCTCACATT
AGCCCCCCCTCCCCGCCCGCCCTCCTTCCCCGGTCTCTTATTAAATACAACATCGTA
AATTGTGGCTCTTCATCTTCTCTCGTGAACATTTGAAAGAATTTTTTTGCCAATTCTGG
TAGGTAGTTTTCAAAATCTTTTATGAGCCCTTACAATTTGTTTAGTACTCTACCATAGT
GTTTTTAATCAATAAGCCAAAGGGGAAAACCTAATAAAAGTTTATAAAATTTCCTCCT
GTATTGGTCCAATTCTTTTGCAACTTATATTATTAATATTATTTATCTTTTGGATTGAAA
TGGATTTTGTATATATCTAATATAAACAAATATGTCTCTTCCGCTTATATGATTTTTCAC
CATAGAAAAATGCTCCCATAAGGTCAGTTATTCTGTCTAAAATATCACACACTTCAAC
CATTGAGATATTTTCTTCTTTGCATCCAGCAATACATTTGGCATCAATAGATAGGAAT
CCAATGAAGATATATTATCAATTTCCTGCAAGTTTCCTTGGCACTAGAAACATTAGAT
CCATATCATGTAAATTGCCTTTGTTAAATTGAAGGTCTATGAAATTTGGGTTGGTTCGA
AAACCTTTTGTTTTTGCCCCCCCACATCCCTAATCGTTTGTTCAGTCAAGGACAGACCT
GACATGTTATGATGACCATTTCTCCAAGGCATTTATAATGGACTGGAGTATCCATGCC
ACGTTTCATCAGCTACATGTTGACTATGTTCCCCTACTTTTTTAATGGCACCATTGTTG

GTGGAGCAAGATTATAGATGTTCCTGATACTTGTATGGTTCCCTTGCTCAATCTCTCTT
TTACTTCATGCAGAAGCCCATTGCTCAGTGTGTATGCTGACATGTCTGTTACATGTAA
GGTATTGACTCGTCTGTACCATTCATACTGGTCTAATCTGTTGGGTATGAGTTGCTGGT
AAATTGCATAATGCTTGTTGGATATGTGTGTGAGTTGCTGCTAGATCTGTGTCCTGCTA
TATTTATGTATGAGTTGCTGCTATTGTAATCTTCATTTAGGATGCATAATGATATAGGT
TCTGTATGTACGGAATAGTCAGGACAATGCTCCTGTCTGTGCACAGGGGCTCTACAGG
AAGCAACTTTCGAAGGAGAAGTAAAGAAAGAGTGATGAAACTTAAGGCAGGGAAA
GTAGTTTCTTTTAGCTAAATTTTGAAATAATTTGAAGGAGGGGAAAACGCTCTCTCAG
TCTGTGGTTGCATTTCATACTCGCACCAAACGGACCCTTGGGGGGGGGGGGGGGGA
GGGGGGCTTAATTCAGTTTTACCTGCAGTGTTCAGTGTTTGGAGAGAGGTGATAAGCC
TACTTCTTAATTTTGTTAGAAAATGTGTACAAAATATAAAGCAGTGGTTACTAAAAAG
TTGGAGAAGTAGTGGGATCTTCTTGCTATTTTTAACCCAGAATAAGACAGCTATGCCA
TATAGCTTTGATTATCTGTTAACGTTCTACATATAAATAGATAATTAATAATGATGTCG
TAATACTAAAGCCTGGAGATCAGACTGCTCTAACTATCCTGAGATGATTACTTTTACT
CTCGGATTAGCTTAGGCGAGCCGCAAGACTACATTGAATCTTTAGAAATGAGAACAT
AAAAAAGGTGCAGAGGTGGGGAAGTGGCTGAACGATATGCATATGGGAGTGAGTGG
GGAGAAAAATTATTTCCTTTACTTGGGTACAATCAAGAATGGATGACAACTTAGCCC
ACTATATCCGTTCATGTGTTCTTTAGGGTCCTCTGATATAACTGGTCTCTCTGCAGGAA
TATTACAACCCCAATCAGTCTATGTTGGAATTGGTATTTGCACCTGCAGAAGAGTGGA
TAAATCGTAGTGACTCAGAAATTATTGATGCTACAATGAAGGAACTAGCAAAGCTTT
TCCCTGACGAAATTTCGGCAGATCAGAGCAAAGCAAAAATATTGAAGTATCACATTG
TCAAAACTCCAAGGTCAGTAATCATTTTGCTTTCATAGTTGTGTAATATGTGAGAATT
ACAGTCCACATGGAATATATTCCTATTCTGAATCCTGATTAATCTGCTTTTTTTCTCTCA
GGTCTGTTTATAAAACTGTGCCAGGTTGTGAACCCTGTCGGCCCTTGCAAAGATCTCC
TATTGAGGGGTTTTATTTAGCTGGTGACTACACAAAACAGAAATACTTGGCTTCAATG
GAAGGTGCTGTCTTATCAGGAAAGCTTTGTGCCCAAGCTATTGTACAGGTCAGTTCTC
ACAGTTGTTTTTGTCCACTGATAGTATATTTGATCAAATTTTGTCATCTTTGCTGCGGTA
GAGAATTTTAGAAGCATGGACGTCAAGCATGCCTCTTACTTATAATTGCTAATTCTGC
GTCAGCTTATAGTTCTCCAACCAATATAGTGTTAAACCAAAAAAAAACAAAATTGTGC
ACACAGATCACAGAGTTACTCAGGATATCTGCACTTTTGGAGCCTCAGTAGTAGCAT
GATAAAATGCAGAAGGTTATGTTTTTTCATTCCTTATTAAACTTATATCTCTATATTTTG
CAGGATTACGAGTTACTTCTTGGCCGGAGCCAGAAGAAGTTGGCAGAAGCAAGCGTA
GTTTAG

>mRNA_13725_cds

ATGCCCCAAATTGGACTTGTTTCTGCCGTTAATTTGAGAGTCCAAGGTAATTCAGCTT
ATCTTTGGAGCTCGAGGTCTTCTTTGGGAACTGAAAGTCAAGATGGTCACTTGCAAAG
GAATTTGTTATGTTTTGGTAGTAGCGACTCCATGGGGCATAAGTTAAGGATTCGTACT
CCCAGTGCCATGACCAGAAGATTGACAAAGGACTTTAATCCTTTAAAGGTAGTCTGC
ATTGATTATCCAAGACCAGAGCTAGACAATACAGTTAACTATTTGGAGGCGGCGTTA
TTATCATCATCATTTCGTACTTCCTCACGCCCAACTAAACCATTGGAGATTGTTATTGC
TGGTGCAGGTTTGGGTGGTTTGTCTACAGCAAAATATCTGGCTGATGCTGGTCACAAA
CCGATATTGCTGGAGGCAAGAGATGTCCTAGGTGGAAAGGTAGCTGCATGGAAAGAT
GATGATGGAGATTGGTATGAGACTGGGTTGCACATATTCTTTGGGGCTTACCCAAATA

TGCAGAACTTGTTTGGAGAACTAGGGATAAACGATCGGTTGCAGTGGAAGGAACATT
CAATGATATTTGCGATGCCTAACAAGCCAGGGGAGTTCAGCCGCTTTGATTTTCCTGA
AGCTCTTCCTGCGCCATTAAATGGAATTTTGGCCATACTAAAGAACAACGAAATGCTT
ACGTGGCCCGAAAAAGTCAAATTTGCTATTGGACTCTTGCCAGCAATGCTTGGAGGG
CAATCTTATGTTGAAGCTCAAGACGGTTTAAGTGTTAAGGACTGGATGAGAAAGCAA
GGTGTGCCTGATAGGGTGACAGATGAGGTGTTCATTGCCATGTCAAAGGCACTTAACT
TCATAAACCCTGACGAGCTTTCGATGCAGTGCATTTTGATTGCTTTGAACAGATTTCTT
CAGGAGAAACATGGTTCAAAAATGGCCTTTTTAGATGGTAACCCTCCTGAGAGACTTT
GCATGCCGATTGTTGAACATATTGAGTCAAAAGGTGGCCAAGTCAGACTAAACTCAC
GAATAAAAAAGATTGAGCTGAATGAGGATGGAAGTGTCAAATGTTTTATACTGAATA
ATGGCAGTACAATTAAAGGAGATGCTTTTGTGTTTGCCACTCCAGTGGATATCTTCAA
GCTTCTTTTGCCTGAAGAGTGGAAAGAGATCCCATATTTCCAAAAGTTGGAGAAGCT
AGTGGGAGTTCCTGTGATAAATGTCCATATATGGTTTGACAGAAAACTGAAGAACAC
ATCTGATAATCTGCTCTTCAGCAGAAGCCCATTGCTCAGTGTGTATGCTGACATGTCT
GTTACATGTAAGGAATATTACAACCCCAATCAGTCTATGTTGGAATTGGTATTTGCAC
CTGCAGAAGAGTGGATAAATCGTAGTGACTCAGAAATTATTGATGCTACAATGAAGG
AACTAGCAAAGCTTTTCCCTGACGAAATTTCGGCAGATCAGAGCAAAGCAAAAATAT
TGAAGTATCACATTGTCAAAACTCCAAGGTCTGTTTATAAAACTGTGCCAGGTTGTGA
ACCCTGTCGGCCCTTGCAAAGATCTCCTATTGAGGGGTTTTATTTAGCTGGTGACTAC
ACAAAACAGAAATACTTGGCTTCAATGGAAGGTGCTGTCTTATCAGGAAAGCTTTGT
GCCCAAGCTATTGTACAGGATTACGAGTTACTTCTTGGCCGGAGCCAGAAGAAGTTG
GCAGAAGCAAGCGTAGTTTAG

>PDS2_Ntab0595110

ATGCCCCAAATTGGACTTGTTTCTGCCGTTAATTTGAGAGTCCAAGGTAATTCAGCTT
ATCTTTGGAGCTCGAGGTCTTCGTTGGGAACTGAAAGTCAAGATGGTCGCTTGCAAAG
GAATTTGTTATGTTTTGGTAGTAGCGACTCCATGGGGCATAAGTTAAGGATTCGTACT
CCCAGTGCCACGACCAGAAGATTGACAAAGGACTTTAATCCTTTAAAGGTTTGTTTTG
AATGCGAAAGTGTGATGATGGATTTATGGTCATGGGCATATATTCTCTAAAATAAGA
GAAGTATATCTTGCCATTCAGGTAGTCTGCATTGATTATCCAAGACCAGAGCTAGACA
ATACAGTTAACTATTTGGAGGCGGCGTTATTATCATCATCATTTCGTACTTCCTCACGC
CCAACTAAACCATTGGAGATTGTTATTGCTGGTGCAGGTGATTTTTTCCGGTCATCTAT
ATTTGTAGTCTTCATTTCTCTTTCTTCGGAAGGAAGATCATTCTATTAGTTGTATTATCA
CTAGAATATTTACCTGGGCATTCTTTTCTGATTAACTGTTTTGGACCGCAAAATTTTAG
GTTCTTACTTCTTCGCCATTTTGCAACTAATCAACAAGTCCCAGCTGATTAGATTAGGA
GCGGTTTGAAAATTCGTTTGTTTTGATCTATTTTTGCCGTCACTCCATTTATATACATAT
CTATTCTCTCTTATCATCTGAGCCATGATAAGCAGGTGAACGTGCTGTCTATTGGGTG
GCATGTCCAAAGGATCATTCTGAAATATTGGAGGCTAATGAATCAATATCTTGTGCAA
GATTGATCTCACTATACCTATAATCAGAGTACTTAGAGTTCCAAAAATTTCAAAACCA
ATTGAAAAGTCAAACGAGTTACATATAGGGGTTGCACTCTTCTAAGGCTTGCAATCTG
TGAGAAAAAGATGAGAAGGAGATCTTCATATTTCATCTTTACTAGTCTGGACCACTGA
CCGGTTAGCAGTTTTCAACGTGTTCTTCAACTTGGCTTGCAAAATACTGTGCCGGTCAT
TTCTCTTGTATTGTCATCAACTGGTTGATTATCTGAGTACCTAAAGAAAGAATGTTATA
TGCATGATTCATTCTGCTGTACTATAAAAGATATAATAAAGAATGCTAGCCGAGGTAC

TACATAGCCTTTTCCAATAAATAGAAGCTGTAACATGATTCTAATTCATTTTTTTGCA
ATATCAGGTTTGGGTGGTTTGTCTACAGCAAAATATCTGGCAGATGCTGGTCACAAAC
CGATATTGCTGGAGGCAAGAGATGTCCTAGGTGGAAAGGTGAAGAAGATCCAGTCTT
TCCTTTAATTTTATTCCTTTTTCTTTTGTGTCCTTCCCTATTGATAGTCCCTTTTCAGGAA
GGCTTCTGTTTGTTTTATTTAAAATCATTTTTCATACTCTTTAAACATTCAGTTGCTCAA
ACAATTGCAAGGGTGTTCACTATTCCTATTCGTGACTGTCGTCTTTTCTCTCAGTTCAT
ATATATATCTGTTTTTGGAGGAAATTGATATGTCCTAAAATTTTCCAGCTTTACTACTA
ATAAAGTTGGTTGTCGAAAAATTATACAGCATATTAGGTAAAAGATATGGAAAGTAT
ATTATTATCATTCTCTATTATTTTATGATTCAGTCAATTTTACCCGTCCTGTTGGGTGTA
TTTCTCGTATAAATACAGTCTTATCTGTGAGATGCTATGTGAATTAGCTGTTGTTTTCA
GTATAGAACACTATCTTAGTCTGTTTTATCTTACTGAAGCAGTCACCAAGAATCTAGC
TGTATAGGCTAAAATATTGAATTAGCATTAATCTTTATCTGTTCTGCACCTGAATACTT
ATACCTCCCTTTTAGGTAGCTGCATGGAAAGATGATGATGGAGATTGGTATGAGACTG
GGTTGCATATATTCTGTAAGTTTGACTCCTCAAGAATGCGTACTTTAATCTTCTAATAC
AGTCATAGCAATTTCTTTCAAGATCTTTTTTGTCCATTAATCAGATAGCAATCCCTGTT
TGTCTTTTGTTTTTTGCAAATAACCAATTTTTGTCAGTCGATCTGTATTCTGCCTTGCCT
CTCTTTTTTCATCTGTTAATTTCGTATGGTGACTCATACAAGTTGGTGCATCTCCTTTAA
GTTGGGGCTTACCCAAATATGCAGAACCTGTTTGGAGAACTAGGGATTAACGATCGA
TTGCAGTGGAAGGAACATTCAATGATATTTGCGATGCCTAACAAGCCAGGGGAATTC
AGCCGCTTTGATTTTCCTGAAGCTCTTCCTGCGCCATTAAATGGTAAGTACTTAATCAT
GAGTAAATTTTTTCCCTTCTGTATTGAATATTGATTATGCAAACTTCCCCAGTAAGGTA
TGAAATTGATTAGTCCATTAATACCTCTGGCGCATTGCTAACATTAAAAGAACATAAA
GGTTCATTACGTCTTGATCAGAATTTCTGCATGTAGCTAAAGTGATTGAGTGTATGTAC
AAATTTTTACACATTGCAAGCATAAGCCAGTTATTGTTATCTCTTATTTTCATTTCTCTA
GCTGTATCTCTTATTCTCATTTCTCTATCTATGTGTTATTACTTCTACAGGAATTTTGGC
CATACTAAAGAACAATGAAATGCTTACATGGCCCGAAAAAGTCAAATTTGCTATTGG
ACTCTTGCCAGCAATGCTTGGAGGGCAATCTTATGTTGAAGCTCAAGACGGTTTAAGT
GTTAAGGACTGGATGAGAAAGCAAGTGCGTGATCATTTTATCTTACTCTTTTAGCTCA
TAACCTTGAAGACATAGTTGACTTGCAAGTTGTTGATTTAACATGTTAGAAATGTCTA
CCTGCCTTTCCTTTTCTAACAACATACATCTTGCAAAACTCATCAGCAGCTATTTGCTT
AATTGCTTTTCAGGGTGTGCCTGATAGGGTGACAGATGAGGTGTTCATTGCCATGTCA
AAGGCACTTAACTTCATAAACCCTGACGAGCTTTCGATGCAGTGCATTTTGATTGCTT
TGAACAGATTTCTTCAGGTTAGAATCCTGATCCACCCTCAAAACAAAAAGAGAGAAA
GGGATATAGTCCTACCAAAGCTGTAAATCATGTTTGGGACCTGACATATTGGTGCAG
GAAACTTATGAGTGAACTTGTCCACTCAGTTTAACTTTTCTGATATATTTGAATTATCA
ATTTGCAGGAGAAACATGGTTCAAAAATGGCCTTTTTAGATGGTAACCCTCCTGAGA
GACTTTGCATGCCGATTGTTGAACATATTGAGTCAAAAGGTGGCCAAGTCAGACTAA
ACTCACGAATAAAAAAGATTGAGCTCAATGAGGATGGAAGTGTCAAATGTTTTATAC
TGAATAATGGCAGTACAATTAAAGGAGATGCTTTTGTGTTTGCCACTCCAGGTATAAT
ATCCATTATACTAGTATCGATGCTTCCAGTTTTCACATTTTTAATATGAATTTATAAATT
TTTGCTGGCTTTTGATTATCCGATTAGTGGATATCTTCAAGCTTCTTTTGCCTGAAGACT
GGAAAGAGATCCCATATTTCCAAAAGTTGGAGAAGCTAGTGGGAGTTCCTGTGATAA
ATGTCCATATATGGTTAGTGATGAAAATTTTACTTGTCAGTGTTTGTTCTTCCTCTAGC
ATATCTATGTATGTGCGTGTAAATGTCTATACGTACATAAACATGTAGTCCTCTGTTTT

TGTGTTAACTTCCATTGAATGAGGAACTTATGGATGTACGCTTTTCCAAAACTTTGATT
GGACACATTGCTTTGTGTTTTCAACTTTGATGAGCAGAACTACCATTGTTTAGCTATTA
GTGGTTGAGATTCCTATTGAAAAGATTTGTATAAATTTAATTTGCAGGTTTGACAGAA
AACTGAAGAACACATCTGATAATCTGCTCTTCAGCAGGTTCATTTTTGATCAATTTTAT
TGTTCCAGAGCAGTTTCTGCGTGTCCATGACTACATTCTCATATTAGCTCCTCCCCCTC
TCCCCCCTTCCCCGGTCTCTTATTAAATACAACATCGGGTAAATTGTGGCTCTTCCTCC
TCTCGTGAACATTTGAAAGGAATTTTTTTTGCCAATTCTGGTAGGTAGTAGTTTTCAAA
ATCTTTTATGAGCCCTTACAATGTGTTTAGTACTCTACCGTAGTGTTTTAATCAATGAG
CCAAAGGGGAAAAACTAATAAAAGTGTATAAAATTTCTTCCTGTATTAGTCCAATTCT
TTTTCAACTTATATTATTAATATTATTTATCTTTTGGATTGAAATGGATTTTGTATATCT
AATATAAACGAAGATGTTCTCTTCCGCTTACATGATTTTTCACCATAGATAAATGCTC
CTATAAGGTCAGTTATTCTGTCTAAATATCACACACTTCAACTGCTGAGATATTTTGTT
CTTTGCATCCAGGAATACATTTGGCATCATTAGACAGGAATCCAATGAAGATATATTA
TCAATTTCCTGCAAGTTTCCTTGTCACTAGAAACATTAGATCCATATCATGTAAATAG
CCTTTGTTAAATAGAAGGTCGATGAAATTTGGGAACCCCATCTCTAATCGTTTGTTCA
GTCAAGGACAGAGCTGACATGTTATGATGACCATTTCTCCAAGGCATTTATAATGGAC
TGGAGTATCCATGCCATTTTTCATCAGCTATATGTTGATTATGCTCCCCTACTTTTTAA
ATGGCACCATTGATGGTGGAGCAAGATTATAGATCTCCTTTTTTTATCTCAAGAAATT
GTGTTGTTCCTGATACTTGTATGGGTTCCCTTGCTCAATCTCTCTTTTATTTTCATGCAG
AAGTCCACTGCTCAGTGTGTATGCTGACATGTCTGTTACATGTAAGGTATTGACTCAT
CTGTAGCATTCATATTGTTCTATTCTGTTGTGTATGAGTTGCTGGTAAATTGCATAATG
CTTCTTGAATTTATGTACGAGTTGCTGCTATTGTAATCCTCATTTAGGGATGCATAATG
ATATAGGCTTTGTATGTTTGGACTATGAATAGTCAGGACACTGCTCCTGTTTGTGCAC
AAGGGTCCTACACGAAGCAACTTTCGAAGGAGAAGTAAAGGAAGTTTGATGAAACT
TAGGCGGGGAAAGTAGTTTCTTTTAGCTACTTTTTGAAAAATTTGAAGGAAGAAAATT
CATATATTAGACATTTGGATTTCTTTTGGTGGAAGGAGAAAAAGGGTAGGGCCCTCTA
AGTCTGCCCCTCGTTGCATTTCTTACTTGCCCCAAAAGAGGGGGGGGGGGGGGGGGG
GCTNNNNNNNNNNNNNNNNNNNNNNNNNNNNNNGGGGGGGGGGGGGGGGGGGG
CGCTTACTTCAGTGGTACCTGCAGTGTTTGGGGAGAGCCGATAAGCCTACGTCTCAAT
TTTATTGGTATGAAAATGCAAAATATAAAGCAGTGGTTACCAAAAAGTTAGAGAAGT
TGACATGGAATGTTTGGTTCATGATAGAACATCGGATCAATTGTCTATAGAAATGAGA
ACATCAAGAAAGGTGCAGAAGAGGGGAAAGAGTGGCTGAATGATATGCATATGGGA
GTGAGTGGGGAGTAAAATTATTTCCTCTGTTGGGTACAGTCAAGAATGGATGACAAC
TTAGCCCGCTATATCCGTTCATGTGTTCTTTAGGGCCCTCTGATATAACTGGTCTCTCT
GCAGGAATATTACAACCCCAATCAGTCTATGTTGGAATTGGTATTTGCACCTGCAGAA
GAGTGGATAAATCGCAGTGACTCAGAAATTATTGATGCTACAATGAAGGAACTAGCA
AAACTTTTCCCTGATGAAATTTCGGCAGATCAGAGCAAAGCAAAAATATTGAAGTAT
CATGTTGTCAAAACTCCAAGGTCAGTAATCATTTTGCTTCCATAGTTGTGTAATAAGC
GAGAATTACAGTCCACATGAATCTATTCCTATTCTGAATCCTGATTAATCTGCTTTTTT
CTCTCAGGTCTGTTTATAAAACTGTGCCAGGTTGTGAACTCTGTCGGCCCTTGCAAAG
ATCTCCTATTGAGGGGTTTTATTTAGCTGGTGACTACACGAAACAGAAGTACTTGGCT
TCAATGGAAGGTGCTGTCTTATCAGGAAAGCTTTGTGCCCAAGCTATTGTACAGGTTA
GCTCTCACTTTTTTTTTCTTTCCATTGATAGTGTATTTGATTATATTTTGTCATCTTTGCT
GCGGTAGAGAATTTTAGAAGCATTTCTCAGACATTAGTTGGCAGTATATGTTTCTGCA

AGCCTGTTTCGATGAATTTGGTGAGAGGACCGGGGTTGTCAAGTAGTACAGCTTTACT
TCTGGCAGACTTTCCTTCTCTCAACTTATACCTGCGAGAGAAATGGACGTCAAGTATG
CCTTTTACTTATAATTGCTTATTCTGTGTCAACTTATAGTTCTCCAACCAATATAATGTT
GAACCAAAAAACAAAATTGTGCACACAGATTGCAGAGTTACTCAGGATATCTGCACT
TTTGGAGCCTCAGTAGTAGCATGATAAAATGCAGAGGGTTGTGTTTTTTCATTCTTTAT
TAAACCTTTATCTCTATATTTTGCAGGATTACGAGTTACTTCTTGGCCGGAGCCAGAA
GAAGTTGGCAGAAGCAAGCGTAGTTTAG

>mRNA_73042_cds

ATGCCCCAAATTGGACTTGTTTCTGCCGTTAATTTGAGAGTCCAAGGTAATTCAGCTT
ATCTTTGGAGCTCGAGGTCTTCGTTGGGAACTGAAAGTCAAGATGGTCGCTTGCAAAG
GAATTTGTTATGTTTTGGTAGTAGCGACTCCATGGGGCATAAGTTAAGGATTCGTACT
CCCAGTGCCACGACCAGAAGATTGACAAAGGACTTTAATCCTTTAAAGGTAGTCTGC
ATTGATTATCCAAGACCAGAGCTAGACAATACAGTTAACTATTTGGAGGCGGCGTTA
TTATCATCATCATTTCGTACTTCCTCACGCCCAACTAAACCATTGGAGATTGTTATTGC
TGGTGCAGGTTTGGGTGGTTTGTCTACAGCAAAATATCTGGCAGATGCTGGTCACAAA
CCGATATTGCTGGAGGCAAGAGATGTCCTAGGTGGAAAGGTAGCTGCATGGAAAGAT
GATGATGGAGATTGGTATGAGACTGGGTTGCATATATTCTGGGAATTCAGCCGCTTTG
ATTTTCCTGAAGCTCTTCCTGCGCCATTAAATGGAATTTTGGCCATACTAAAGAACAA
TGAAATGCTTACATGGCCCGAAAAAGTCAAATTTGCTATTGGACTCTTGCCAGCAATG
CTTGGAGGGCAATCTTATGTTGAAGCTCAAGACGGTTTAAGTGTTAAGGACTGGATG
AGAAAGCAAGGTGTGCCTGATAGGGTGACAGATGAGGTGTTCATTGCCATGTCAAAG
GCACTTAACTTCATAAACCCTGACGAGCTTTCGATGCAGTGCATTTTGATTGCTTTGA
ACAGATTTCTTCAGGAGAAACATGGTTCAAAAATGGCCTTTTTAGATGGTAACCCTCC
TGAGAGACTTTGCATGCCGATTGTTGAACATATTGAGTCAAAAGGTGGCCAAGTCAG
ACTAAACTCACGAATAAAAAAGATTGAGCTCAATGAGGATGGAAGTGTCAAATGTTT
TATACTGAATAATGGCAGTACAATTAAAGGAGATGCTTTTGTGTTTGCCACTCCAGAC
TGGAAAGAGATCCCATATTTCCAAAAGTTGGAGAAGCTAGTGGGAGTTCCTGTGATA
AATGTCCATATATGGTTTGACAGAAAACTGAAGAACACATCTGATAATCTGCTCTTCA
GCAGAAGTCCACTGCTCAGTGTGTATGCTGACATGTCTGTTACATGTAAGGAATATTA
CAACCCCAATCAGTCTATGTTGGAATTGGTATTTGCACCTGCAGAAGAGTGGATAAAT
CGCAGTGACTCAGAAATTATTGATGCTACAATGAAGGAACTAGCAAAACTTTTCCCT
GATGAAATTTCGGCAGATCAGAGCAAAGCAAAAATATTGAAGTATCATGTTGTCAAA
ACTCCAAGGTCTGTTTATAAAACTGTGCCAGGTTGTGAACTCTGTCGGCCCTTGCAAA
GATCTCCTATTGAGGGGTTTTATTTAGCTGGTGACTACACGAAACAGAAGTACTTGGC
TTCAATGGAAGGTGCTGTCTTATCAGGAAAGCTTTGTGCCCAAGCTATTGTACAGGAT
TACGAGTTACTTCTTGGCCGGAGCCAGAAGAAGTTGGCAGAAGCAAGCGTAGTTTAG

>ZDS_Ntab0653840

ATGGCTACTTCTTCAGCTTATCTTTGTTGTCCTGCAACTTCAGCTACTGGAAAGAAACA
TATTTTTCCAAATGGGTCAGCTGGATTCTTGGTTTTCCGTGGTCCCCGTTTGTCGAACC
GGTTAGTGACTCGAAAGTCAGTTATTCGTGCTGATTTGGACTCCATGGTCTCTGATATG
AGTACAAATGGTAAAGTTTGTCCACTTCCCTATTTAATTTGAGATATTGAAACTGAAT
TATAAAAGGGTGTTAAGTAATTGTGTTTTCCTATGTGTATAGCTTGGTTGGAAGTGTCT

TTTGTCTTTGAGTTTTGTTCTCTAATTTTGGTGGAAGATAACATTTCTGTGGGTACCAAG
AAGGTTAATTTTTTTTTGTTGGTATTTTGTTGGATGCTTTGGATATCAGCTTTTTGTTTTT
TGTTTTTTTTTAAAAAAAAAGTTGTTTTGTGTATGTCTTGATTGCACATTTGTGTTTTCTC
TTTGAGCCTTGCTTTTAAACCTGGACAATTTGACTTAAGGTGGTCTTATTTATCTGTGTT
GTAAATTCAGAAGTTCTAGTGGAAGACAGGATTTTTAAGGATGTCAGCGCACTTTGA
GGTTGATGGTTCTTTGTACTTTCCTGTAATCTTTTGTATCACAGATTTCTTCATCTAAAA
GGAATAATGACTAGCAAACTAGGGCTTCTTGCATTTCATGTGAAGTTTTGCTTAGCAA
GGAAAATAGTGCCAAGGCATCACATCCAAACCATTAACCCAATAAAAGAGTGAGCA
ATCATAGGTCAGAGGGTCCACAATGGTTTCGGATTGAATTAAGCTGCAAAGATGTGT
GTGTTTGAGGTTGGAGAAGGGTTAGAGGGCGAGGCAGGAATGATTAAAAGATGAAA
TGGGTGAGAAGCATAATTCATTCCCTTTTGTATACTTGCTTTCGCAGGATCAGTCTAGG
ACTGGATATGTCAAGAAAGCGTAGGCAAGTTGGAAACTAAAGAACTTCACTATCATT
TCAATCTTCTCTCTTTAGACAGAGATATATACTTTTAACGTAACTTAATGATAAACAA
GTTGTACTAAAATTTTGAAAAAGAACTATCTGAATAAGGTAAAATGAGTTGGAAACA
GCCTCTTGCAAAAATACAGGGTAAGGCTGCGTACAATAGATCCTTGTGGTCCGGTCCT
TCCCCGGACCCCGCGCATATAGCAAGAGCTTAGTGCACCGGGCCGCCCTTTTTATAGT
GTCTGGAAACCTAGAGGGACAATCTCTGTTATATAAGTTAATGTACTGCTGCTGAAAC
AAATTTTCAACTTACAGCTCCAAAAGGGCTATTTCCACCTGAACCTGAACATTATCGA
GGGCCAAAGCTGAAAGTAGCTATTATTGGAGCTGGGCTTGCAGGCATGTCGACTGCT
GTGGAGCTCTTGGATCAAGGACATGAGGTTCAGAAGCCTAACATCCCCTTTCAAGAA
TTGCTATGATCAATTCACTTTGAAACAAAACTATTAACCAAAAAGATGAAAAAATTG
TCTTTATCTTTGTTTTTTCCCAGTGACCTGGAATTTTCATGTCTTCTACTTTCTTTCAGGT
GGATATATATGAATCGAGGCCTTTTATTGGTGGGAAAGTGGGTTCTTTTGTTGATAGA
CGTGGAAACCACATTGAAATGGGACTGCATGTGTTCTTTGGTTGCTATAATAATCTGT
TCCGTTTGTTGAAAAAGGTGCACAGAGCTCTCCATTCATAGTTCTATTGTATTTCTACT
TACTAAGTGGTTGCGCATGCAATGGAACTGCATTACTGCTCACTCTTAGTTAAGGTGC
ATTAATGCCTAAAATTAAACTTCGTTTGATAGTAGGACAATGGATATTAAAGTGCAA
GGAGTCTTGATATTTTTTTCCTGTTCAGCTTTATCAGTTAAGTAATTCTATTTTTTTTTCC
TGGTTGATGTAGGTGGGTGCTGAAAAAAATCTGCTAGTGAAGGACCATACCCACACA
TTTGTAAATAAAGGGGGTGAAATAGGGGGTATGATTTTATTTGTGTGACACAAAATAT
TTCATCTTGTGGAACTTTAAAATGCTTTCACAAATGACTCCTAGAGGAACATGTCTTC
CAATATATAAAGTTAACATGGTCTGTTACTATCTAATTTACCGTCTATACCTCAATGCA
GAGCTTGATTTCCGTTTTCCAGTTGGAGCACCCTTACATGGAATTAATGCATTTTTGAC
TACTAATCAGCTAAAGGTAACATTCTAATGCCCTCTTCGTAATTGATTTTGCAGTTCAT
GCTATCTCTTATACTAAAATAAAAAATGTCCAGTATAGCTCCTTGGTTTGATCAGTGTT
AATGACCATGCCTTTGGCATTTGACTCTTGGCTTCATTTATTTCTGCACCTATATCAGC
TCCCTTCTTAACAGTTCACTTAGACCGTAGAATGGTATAATAGTGATCTTCGGGCTGG
TTGTCTACCATTATTAATTCGTGGTTTTCTTTGAAAAAAGAAGAGAGAAGATATCAGT
TGTTGTCATTTCTGTGTTGCTGTTGTTGCAAACCCTGATTAATGCTCGTCTTAAATGTGA
TCGTCGTTAACATGGCCTTCCCTTTAAATGCAGACTTATGATAAAGCTAGAAATGCTG
TAGCTCTTGCCCTTAGTCCAGTGGTGCGGGCTTTAGTTGATCCAGATGGCGCATTGCA
GCAGATACGTGATTTGGATAGTGTAAGTGTTATTTCCTTTTTTTCCCTCCTCCTTGTTAA
CAAGTCAGATAGTCTGTTTCTCTGCAAGGATGTGTCCTCTCCAGCTACTTGTTCCTTAC
AATAGCTGTTTCAACTTTCAGGTAAGCTTTTCGGAGTGGTTTATGTCTAAAGGCGGGA

CGCGTACTAGCATCCAGAGGATGTGGGATCCTGTCGCATATGCTCTTGGATTCATTGA
CTGTGACAATATCAGTGCTCGGTGTATGCTCACTATATTTGCATTATTTGCCACTAAAA
CGGAGGCTTCCCTATTACGCATGCTTAAAGGTTCTCCTGACATTTATTTGAGTGGTCCC
ATTAAGAAGTACATCTTGGATAAGGGAGGCAGGTAAGACCAAATATGGAAACAGCT
GCTCACTGTGAATTAGCTGATTTTGGACTTGAAATTGCTATTGTGGTCCAGCAGATTTC
AGCCTAAAATTTTCTCATGAATGAACTGCTTATGCTCAGTTTATTCTGAATCTGTTCTC
TGTTAACAGGTTCCATCTGAGGTGGGGGTGCAGAGAGGTACTCTATGAGACATCCTCT
GATGGAAGTATGTATGTTAGCGGGCTTGCCATGTCAAAGGTAAAATCTTTCTTTCATC
TGTTTATCACCTTTTAAAAGAACATCTTTCAATATGTTCAAATGTTAGGCTCTTTTATG
CATATATAAGTACTTCATGCTGCTTTACTAGTGTGGATGTGCAGGCCACACTGCTTTAC
AAGTGTGGATGTGTTCATTAACTGCTATGGTGTCTATGTGCAGGCCACTCAGAAGAAA
ATTGTAAAAGCTGATGCCTATGTCGCTGGTTGGTAATCAACAATATGATTGATGTGAA
TTTTCCCTCATTTCCCTCCTTTTTAATAGCATAAAACTGAAACTGTTTTGGCTGATATCT
AATTGCAGCATGTGATGTCCCTGGAATTAAAAGATTGGTACCTCAGAAGTGGAGGGA
ATTGGAATTCTTTGACAACATTTACAAATTGGTTGGAGTGCCTGTTGTTACAGTACAA
CTACGATACAATGGCTGGGTTACAGAGTTGCAGGACTTGGAGCGTTCGAGGTGATAC
ATTTGTGGCTCAGAAGCAACTATTTGCCTCAATTTGAAAATCTTGTATGATGAACTAC
TATATCTGTGTGTTAAGATAAGAATCTCTCTGTAATCCAGGCAATTGAAGCGCGCTAC
AGGTTTGGACAATCTCCTGTATACACCAGATGCAGATTTCTCTTGCTTTGCGGACCTTG
CATTGGCATCTCCGGAAGATTATTACATTGAGGGCCAAGGCTCATTGCTTCAGTAAGG
AATTACATTCTTATCTTTACTATCCGAAAGCTGCTCAGCAACTCAATAGATATTCTGAT
CAGATTAAATCTTCTGGTTATCTTCTCTGATCTATCTTGGTGCCTTCAGATGTGTCCTTA
CGCCAGGTGACCCGTACATGCCTCTACTAAATGATGAAATCATAAAAAGAGTGTCAA
AGCAGGTAATTTAGCTTCTTATACGTGCCTTTTTTTCTTCTTCTCTAAAACCTTTAGAAC
ATGATTTTACTTAACAGGAAATGATATAGTATACATAATAACCGAGTAACCTGCAAG
GGCCTGGGATAAGGTCTAGTGGTAAGAGCGCAACACATGTTATGTGGGTTAGGCGCA
CATTACGGGTTTGAAACCTACTGCAAACAAAAGCTTGGTAATTAAGTGGAGAGAGGA
AGAGGTGCAGGCCCTATTATCCACCGAGTTTCGAACTGTGCGCCACTAGCCCTTGCAG
ACTTCTTTGGTTTTCAAAAAGAGTGTAGCATTTGGTTGGCTGCATAAGAGAAAATAAT
TTTTTCATAACTAATGTAACGTTTGGTTAGCGGTATTAAATACTATCTGCATTAAGTTA
TATGAAAACTATGTATAATTTGATGGGGAAAAGAATATTTAGTAAGAATGTGTGCGTT
AAGTTATATGAGAACTATGTATAATTTTATGGGGGAAGAATATATAGTAAGAATGCG
TGTATTAGCAGTACGAGGATTAAGCTATTAAACGACAAAAGTGTCCTTAAAGTAAGT
GTTCTGCATTATAACCCTTGGATAACTAGGATGTGAATCAAATGGCTCCTTAATGTAT
AAGGTATTTTGGTTTCAGGTTTTGGCACTATTTCCTTCTTCCCAAGGTCTTGAGGTTACC
TGGTCATCAGTTGTGAAAATTGGGCAATCCCTATATCGTGAAGGACCTGGTAAAGAC
CCATTCAGACCTGATCAGAAGACGCCAGTGGAAAATTTCTTTCTTGCTGGCTCATATA
CAAAACAGGTAGTCTGCTTTTATCCAGTACTCTTAATACTTTGATCCGTGTATTAAACA
TGTGATGCTTCTACTCAAAGAAAAAAGTTTATCTTAGTGATAGTAAGATTTAGGTCAA
GTTTAATATCTGTTTGGCCAATATTTACCAGAAAAAAGAAGTGGATGGCTGAAAATC
ATATTATGTATATAGAAGTGCATATTCTTTACGAGTTGTGCATTGATAGTATCCGAGTC
TGATTGAAATGGTCTCTTTGAGAAAGTGTTACAAGAAACTTCTTGGTCTTGATTGTTGT
AAAAAGAAGAGTTCATATGTAACTAATGACAGTTTGGAGGTTTTGAAACTTTAAACT
ATTGGATCAGGACCTCTTTATGCCAAATAGTACACATGTCTTATGTGAAATTTAACGA

TGAGCTGCAGCCAAGTATGCAATACTACCTCCGATAATTTCGTTTTGCTGAGGATACA
GCACATTGGTTAACTTCCATCTTGTGTGAAAATGCCTGTTTTCCTCAGGACTACATCGA
TAGCATGGAAGGGGCAACTCTTTCAGGTAGGCAAGCTTCTGCATACGTATGTGATGCT
GGCGAGAAGCTGGTGGCGTTTCGGAAAAAGATTGCTGCTGCTGAGTCAAACGAGATC
TCTGAAGATGTATCAGTATCTGATGAGTTGAGTCTTGTCTGA

>mRNA_101234_cds

ATGGCTACTTCTTCAGCTTATCTTTGTTGTCCTGCAACTTCAGCTACTGGAAAGAAACA
TATTTTTCCAAATGGGTCAGCTGGATTCTTGGTTTTCCGTGGTCCCCGTTTGTCGAACC
GGTTAGTGACTCGAAAGTCAGTTATTCGTGCTGATTTGGACTCCATGGTCTCTGATATG
AGTACAAATGCTCCAAAAGGGCTATTTCCACCTGAACCTGAACATTATCGAGGGCCA
AAGCTGAAAGTAGCTATTATTGGAGCTGGGCTTGCAGGCATGTCGACTGCTGTGGAG
CTCTTGGATCAAGGACATGAGGTGGATATATATGAATCGAGGCCTTTTATTGGTGGGA
AAGTGGGTTCTTTTGTTGATAGACGTGGAAACCACATTGAAATGGGACTGCATGTGTT
CTTTGGTTGCTATAATAATCTGTTCCGTTTGTTGAAAAAGGTGGGTGCTGAAAAAAAT
CTGCTAGTGAAGGACCATACCCACACATTTGTAAATAAAGGGGGTGAAATAGGGGA
GCTTGATTTCCGTTTTCCAGTTGGAGCACCCTTACATGGAATTAATGCATTTTTGACTA
CTAATCAGCTAAAGACTTATGATAAAGCTAGAAATGCTGTAGCTCTTGCCCTTAGTCC
AGTGGTGCGGGCTTTAGTTGATCCAGATGGCGCATTGCAGCAGATACGTGATTTGGAT
AGTGTAAGCTTTTCGGAGTGGTTTATGTCTAAAGGCGGGACGCGTACTAGCATCCAGA
GGATGTGGGATCCTGTCGCATATGCTCTTGGATTCATTGACTGTGACAATATCAGTGC
TCGGTGTATGCTCACTATATTTGCATTATTTGCCACTAAAACGGAGGCTTCCCTATTAC
GCATGCTTAAAGGTTCTCCTGACATTTATTTGAGTGGTCCCATTAAGAAGTACATCTTG
GATAAGGGAGGCAGGTTCCATCTGAGGTGGGGGTGCAGAGAGGTACTCTATGAGAC
ATCCTCTGATGGAAGTATGTATGTTAGCGGGCTTGCCATGTCAAAGGCCACTCAGAA
GAAAATTGTAAAAGCTGATGCCTATGTCGCTGCATGTGATGTCCCTGGAATTAAAAG
ATTGGTACCTCAGAAGTGGAGGGAATTGGAATTCTTTGACAACATTTACAAATTGGTT
GGAGTGCCTGTTGTTACAGTACAACTACGATACAATGGCTGGGTTACAGAGTTGCAG
GACTTGGAGCGTTCGAGGCAATTGAAGCGCGCTACAGGTTTGGACAATCTCCTGTAT
ACACCAGATGCAGATTTCTCTTGCTTTGCGGACCTTGCATTGGCATCTCCGGAAGATT
ATTACATTGAGGGCCAAGGCTCATTGCTTCAATGTGTCCTTACGCCAGGTGACCCGTA
CATGCCTCTACTAAATGATGAAATCATAAAAAGAGTGTCAAAGCAGGTTTTGGCACT
ATTTCCTTCTTCCCAAGGTCTTGAGGTTACCTGGTCATCAGTTGTGAAAATTGGGCAAT
CCCTATATCGTGAAGGACCTGGTAAAGACCCATTCAGACCTGATCAGAAGACGCCAG
TGGAAAATTTCTTTCTTGCTGGCTCATATACAAAACAGGACTACATCGATAGCATGGA
AGGGGCAACTCTTTCAGGTAGGCAAGCTTCTGCATACGTATGTGATGCTGGCGAGAA
GCTGGTGGCGTTTCGGAAAAAGATTGCTGCTGCTGAGTCAAACGAGATCTCTGAAGA
TGTATCAGTATCTGATGAGTTGAGTCTTGTCTGA

>CRTISO1_Ntab0634540

ATGGCGTTGAGATTTCATCCTTTTTCTCCCCTTTTCAACTTCCGCTCCACTAAACGAAG
TCGCACAGTTTTGGCGCGCAACGAAGTCTCTGCCACTTCTAACGGCTATCCTTCTTCC
ACTCTTACCTCCAAGCAAGGTTGGGCACTCTGTGTGTGTGTGTGTGTTTTTTAAGTATT
TTGTCACTGTTTTAGCTAACCGAGAGTCAAATTAACTACATATTGACTACCATTTTTTA

ACAAATGCTTTGTACTTTGGTATAACAAAAACTTATAGTAGTAAATTTAGTGTAGTTT
CTGAATATGTAAACTTCAATTTAGGGAGAATGATAGCTCCAAAAATTGGTCAAGGTA
ACAGTATTCAACAAGCCAAAAGTGCCAAACAAATTGGGATGGGGGAGAATGTACTT
GCATGCTTCTCAATTTCGGATATTCTAAAATGTATTACTAACTTTCTTTAAAAAAGATT
GACACTATTTCTTTGTTAGTCCGTTTAAAAAAGATTGACACTTTTTAATATTTCCTTGA
CAAAAAACATTTATTGACACACAAGTGATACGATAGGTTTCATACCACAAATTTCAA
AATTTTTAAAGCCATATAAATACTATAGTTTATTTAGGACCACATATCTTAAAAGTAT
TTCTCTTCTTTATTAAAGTTTGTGTCAAGTCAAAGTTAGACAATTTACATGGACAAAT
GGAGTACTTTTTCCTTATAAAAAAAAAGAACAAAATTATGCACGATATGTCTGCCTGT
TAAAAATTCCTGGTTTCTAATGTCTGTTAAAATTGTTCTCTTCATGCTTAGTATGCAAC
AGATTGGTATTCCTAGGATATGATAACTGATGATGTATCCAATTGTTAATCCATGAAA
TATGGAACTCAGCGTAAATTGACGTTCCAGCGAAGGAAAAACGCGTACTTCTGAAGT
TATACTATGAGAGGATATGCTTGTGTAGACTTCTAATGCAGATAAGTTGTCTCTCTCTA
CAGGAACATGTTTATTTGACTGCAAGAATTATGTGCAGGCAAGCCAGAAGCAGATAT
CATTGTTATTGGGAGCGGTATAGGTGGGCTATGTTGTGGTGGACTTCTTGCTAGGTATG
GCCAAGATGTTTTAGTACTCGAAAGCCATGATGTAGCTGGGGGTGCAGCTCACTCTTT
TGATATTAAAGGGTACAAATTTGACTCTGGTCCATCATTGTTCTCCGGTTTTCAATCAA
GAGGTCCTCAGGCAAATCCATTAGCACAGGTTACACTCTATCTGCTTACAATTTTGCT
TTGTTTGTTTAGAATGTAAATTGCATTCGTCTTATGTGGATAGAACACCTCTTTAGGGC
TTTATATATTAGCATTTTCTTTGTCACATCGACATTCTTAAGCCACCACTCATGAAATA
AAATGTATGGGATGTTAACAAATTAAACATCCGTGGAGAAGCTGAAGACAGTTTACT
GGCTTTTTCTCTACCTAGAAGTTCATTCTAGAGTGTTGTTGATCACCTAAGAAGCGCCT
GATCAATCGTGAATAGCAATGACCACAAAAATTGCAGCAAATCAGT

ACTGTGAAACTTCGAAGAGCTAGCTATTGACGTTTTATGCATATGTTGAAGATGGAAT
ATAATTATGGTCTTCCCCTTAATAGTTAAATATTTAGGATGTGTGCGAGTTACAAAATT
CAACAGTACGTAAAACACTGAACTCAATCACCTGGATGGCTGATTGTGAAATCCTGT
CCATTCACTATCTAACTGTGAAAATGAAGAATAGTGCTGGCGCTGACAAACGTTTTA
ATGGGATCAGTTTCACTAATATATGTATGTTGCAAACATGTATTGTATGTTAATGTGA
AAGGTTATGGTGGATGTAAACCATGAGAGGCCAAGCTGCTACAATCAACGGGCCAGT
TTTTATAATTGATTTCAAACATTCTTTATGCTGAATTCTGGCTTTATTGCCTTTGAATGA
ACTCTTTGAATTTATTTTTTATTTGTCTGTCAATGTGTTTCATCACCTTTAAGTTGGATG
GTATTAGAAACCGGCTTTGATTTACTATTTGAAGAACCTACTAGCTTCCCCTCCCTTGG
ATAGCCAGATATGCATTTATTAGTGCAACATGCTTTTATTGACCATATGCGCTGCTCTT
TATGTAAAACTACTCTATGCGTTGTATTCTGTAGATTTTGTTTCGGGGTGGGAGGGTAG
CAAGAGGGATTTGATGTTTTAGGACAATAGCTTCAATAAGTTCAGGTCACCAATTTTC
ATTTCATTCTTATTCGAAGAAAAGTTGATGTGTCCTGTTGCTCTGTAAATCTGGTAGAA
CAGATGGCCTGACGACAAATATGTTCTTGATCCTTAATGCCTTTGTTCAGTGACAAAT
AAGTGACTTGCGGCTCCATTTTTTAAAAACATTCTTTGCTTGGCCTAATTAACTAGTAA
TTCTGTACTGCAATTTATTGGACATTTAGTCTGTCTTCTTGTTAGCAAATGCAACTCAA
CTTATTGATATGTTTCTCAGGTTCTTGATGCATTAGGTGAATCAATTCCCTGTGCAAAT
TATGACTCATGGATGGTATATGTACCTGAAGGTGAATTCTTGTCACGCATTGGCCCAA
CGGAATTTTTCAAGGTCTGACCTGCTGGTTTTTTCAAGGTCTGACCTGCTGTTTGCTTTT
GGTAACATGTTTCGTGGACATCACTAAAAATTTATCCTGTGTGTTTTCATTGTTTGCAA
GCTTCACGTTGTATTTCCTTTAACATGC

TTCATGATAATCTTTCTCACCATTCAGGATCTGGAGAAGTATGCAGGACCAGATTCAG
CGAGAGAGTGGAGGAAACTTCTCGTGAGTATTGGATACTTTCCTTACATGTTTTCTTA
CATGATTTATTGCTCGAATACTCAGAGACTGAGAGACATATTTTTTTACAATTTAGCTG
AGATCTCTCGGCAGTCCCTTTCTCTCCAACTTTTGTAAGAGCTCTTTACTGTTCTCTGTT
ATCGAATCTTATCTCATTTGCATAGAGTGTGGTTTTTACAGGACGCAATACTTCCAATC
TCAGCAGCTGCAATGGCTCTACCTCCTTTATCTATCCGAGGTGATTTGGGTGTTATTTC
GACTGCTGCTGCTAGATATGCACCTTCTCTCTTAAAAACTTTTGCTCAAATGGGACCTC
AAGGAGCCCTTGGTGCTACCAAGCTTCTCAGACCCTTTTCGGAAATCATTGATTCTTT
GGGGATAAAAGACCCTTTTATACGAAAAATAAAAGACCCTTTTATACGAAATTGGCT
AGATCTCCTAGCCTTCTTGCTTGCCGGGGTCAAAACTAACGGCATACTCTCAGCAGAA
ATGGTAATTATGGAACATGCATTTACCTTATAGAGATATTTCTTGGAAGTTAATGTTGT
TTGCATCTATCAGAAAAAAATTGCTTGATCTTTGCTCAAGTGTCTCTAAATCATGCGTG
TTGCTTCCTCCATATTTGTAAAAATATGGTAACAAGCTAGCTGTTTTTTCTCTAAGGAT
TTTATAATTCATCAGTTGCACTTTAGACATTAAATACTGAATTCTACTTCTACAGGAGG
ACCTCACACACTAATATTGGAATCAGGAAATTTACCTTTTAGATTAATATGCTAATTTT
GGTTTTGCGCACACTGCAATTGTCTACTTTTGTTCTGCTTGAGATTTTGTTGGTTTTGCC
GCGGAAATTAAGAGTTTCTTAAAGTTCACTTTTATAATAGGACCTCAGTTCCATCCTA
TGATTGGAACCCAATAGGTCGTCTTGTAGATTACTGTCAAATTTGACCTGCACACATC
GCAAATTGCCTACTTATTTTCCAGTGAGTACCAGAAATTTTGAGCATATCCCAGTAAG
TTTATCCCTTTTATGGATTTCAATCCCGAGTTTGTATTTGTGGTTCACTTTATCTGAGAG
CTCAATTCGTACTAGAGGTGATTCAGTTTAAACTTAACTTGTTTGCTAAACATGAAAT
ATGTTCAAACCTAATGACAAAGTCCAAAGCATGGGAATCTTCCCCTGTTTACACTTCT
CATTTTGCATCACTATTGACAAATGTGATGGCTGCAACAACCTACTTTTGCTGAATGA
ATTTAATGGTTTTCACTTGGTTGGCATGCATTGGGATTAAGCCGATCTTGTATTAATCT
CATCTTCATGTCTCACAAATTCTTTATTTGATTTTATGTTTAACCTTCTTCAATTAATTTT
TCTTATTCATTAATGTAGATTAATCACTGTCATCCTTTATGCTTCATAGGTGTACATGTT
TTCAGAATGGTATAAGCCGGGTTGCACTCTAGAATATCCACTTCAAGGAAGTGGAGC
AATTGTTGATGCTCTTGTTCGAGGGCTACAAAAATTTGGTGGGAGGATTTCTCTCAAG
AGTCACGTAGAAAATATAGTTGTTGAAAATGGTCGAGCTGTTGGAGTCAAACTAAGA
GGTGGCCAAGTGAGTAAATAAAAACTATGATGCATAGTGAAATGATACACGTCGTTT
TGATTAAGGTTGTGCTTTTTCCTTGTGAAAAACATAAACCAAAATATTTTATGCTGCA
GTTTGTCCGTGCCAAGAAGGCTGTAGTCAGCAATGCATCTATGTGGGATACCTTGAGC
TTATTGCCTCCAGAAGTTGTCCCAAAATCATACCGAGACAACATCAAATCGACCCCA
CAGTGTGAATCGTTCATGCATCTGCATTTGGGTTTTGATGCAGAGGTAAGCTATAGCA
TAACGATGCATTGTTGAGAATTCTATTAGTAGGCTGTGGCAATGCAATATGACCTTCA
TGGATTGAAAGTAAAAGACCACATAAACTGGACTATCCAATAATATGATATAATGAT
TAGAAAGGTCATTTTCGAGGTAATTATGTTAGTACTATTCTGCTTGTTCCATTCTTTTAT
GCTCTTCCCCTTGTGGGTGGGTTGTGGAGATATTTCCTTTTTTTCTGATTGACATTGCTT
CATAGCCATTAAAACATGTCTCTGGGTGTCGCTTTTCATGGTTTCTTTTATGTCCATTG
AAATGGATGTTTAGGGTATACGTGATGACCTGGGAATCCATCATATAGTAGTAAATG
ACTGGGACAGAGGGGTTGATGCTGATCAGAATGTCATACTGATATCCGTGCCCAGTG
TGCTCAGTCCAAATCTTGCTCCACCCGGAAAGCATATTTTGCATGCCTATACCCCTGG
AACTGAGCCATTTGAAATTTGGGAAGGTCTTGATCGCCGAAGCAATGAGTACAAAAA
CCTCAAGGCTGAAAGATCTGAGGTACTCCTTGTTCTCTTATTTATTGATGATAAACAGT

GGTCCGGCTTCATTATCTACAAAATCTTGAGATGATAGTTTTGCTGATCAATTTGCATT
ATTTGTGTCTCATTAGTATGATGTTAGCTATCAAATTGTTAGATGCTGCGTCTTTCATCC
TGAATCAGAAGTTAAGCCACAATGCTCAAGTGATTGTGCATAGCTGATTTATGCTATA
GATATTTCCTGTTAAGTTTATTCTCGTTGACTTTCCTTTTCCTTCTCTGTGTTTATCTTTG
CAATTTGTGACCTTCAGTCAAGAACTTGTTGAACTTTTCACTTCAGGTAGCTGTAAATA
GGTGTGCATATTGAGGTCCCTTTTCACCTGATTTTCTGTTTACATGGCAGGTAATGTGG
AGGGCTGTGGAGAAAGCACTTGGGCCAGGGTTTAATCGCGAGAAGTGTGAGGTGAA
ATTAGTTGGAACTCCATTAACACATAAAAGATTTCTTAGAAGAAACAGAGGGACTTA
TGGGCCAGCTATATTAGCAGGTAAACATAGGTGGATCTAGGTCGGCTTCAGTGTGTTC
AGCTGAACTCATTGCTCTCGATTTGATTAACTATGTATACATATGCAAAAATAATTAA
AGTTAAATTGATAATAAAATCACACTCATTCTGAAACCACGGACCTTATCTTGGATTC
TCGTCTGCAGGTAAAGGCACATTTCCTGGACATTCCACACCAATTCCACAACTCTTGT
GCTGTGGAGACTCTACTTTTCCTGGCATTGGAGTGCCTGCAGTTGCTGCTAGTGGTGCC
ATTGTTGCGAATTCGCTGGTTTCTGTGGCAGAACATTCACAGCTTCTTGATGCTGTAGG
GATATGA

>mRNA_114973_cds

ATGGCGTTGAGATTTCATCCTTTTTCTCCCCTTTTCAACTTCCGCTCCACTAAACGAAG
TCGCACAGTTTTGGCGCGCAACGAAGTCTCTGCCACTTCTAACGGCTATCCTTCTTCC
ACTCTTACCTCCAAGCAAGGCAAGCCAGAAGCAGATATCATTGTTATTGGGAGCGGT
ATAGGTGGGCTATGTTGTGGTGGACTTCTTGCTAGGTATGGCCAAGATGTTTTAGTACT
CGAAAGCCATGATGTAGCTGGGGGTGCAGCTCACTCTTTTGATATTAAAGGGTACAA
ATTTGACTCTGGTCCATCATTGTTCTCCGGTTTTCAATCAAGAGGTCCTCAGGCAAATC
CATTAGCACAGGTTCTTGATGCATTAGGTGAATCAATTCCCTGTGCAAATTATGACTC
ATGGATGGTATATGTACCTGAAGGTGAATTCTTGTCACGCATTGGCCCAACGGATTTT
TTCAAGGATCTGGAGAAGTATGCAGGACCAGATTCAGCGAGAGAGTGGAGGAAACT
TCTCGACGCAATACTTCCAATCTCAGCAGCTGCAATGGCTCTACCTCCTTTATCTATCC
GAGGTGATTTGGGTGTTATTTCGACTGCTGCTGCTAGATATGCACCTTCTCTCTTAAAA
ACTTTTGCTCAAATGGGACCTCAAGGAGCCCCTGGTGCTACCAAGCTTCTCAGACCCT
TTTCGGAAATCATTGATTCTTTGGGAATAAAAGACCCTTTTATACGAAATTGGCTAGA
TCTCCTAGCCTTCTTGCTTGCCGGGGTCAAAACTAACGGCATACTCTCAGCAGAAATG
GTGTACATGTTTTCAGAATGGTATAAGCCGGGTTGCACTCTAGAATATCCACTTCAAG
GAAGTGGAGCAATTGTTGATGCTCTTGTTCGAGGGCTACAAAAATTTGGTGGGAGGA
TTTCTCTCAAGAGTCACGTAGAAAATATAGTTGTTGAAAATGGTCGAGCTGTTGGAGT
CAAACTAAGAGGTGGCCAATTTGTCCGTGCCAAGAAGGCTGTAGTCAGCAATGCATC
TATGTGGGATACCTTGAGCTTATTGCCTCCAGAAGTTGTCCCAAAATCATACCGAGAC
AACATCAAATCGACCCCACAGTGTGAATCGTTCATGCATCTGCATTTGGGTTTTGATG
CAGAGGGTATACGTGATGACCTGGGAATCCATCATATAGTAGTAAATGACTGGGACA
GAGGGGTTGATGCTGATCAGAATGTCATACTGATATCCGTGCCCAGTGTGCTCAGTCC
AAATCTTGCTCCACCCGGAAAGCATATTTTGCATGCCTATACCCCTGGAACTGAGCCA
TTTGAAATTTGGGAAGGTCTTGATCGCCGAAGCAATGAGTACAAAAACCTCAAGGCT
GAAAGATCTGAGGTAATGTGGAGGGCTGTGGAGAAAGCACTTGGGCCAGGGTTTAAT
CGCGAGAAGTGTGAGGTGAAATTAGTTGGAACTCCATTAACACATAAAAGATTTCTT
AGAAGAAACAGAGGGACTTATGGGCCAGCTATATTAGCAGGTAAAGGCACATTTCCT

GGACATTCCACACCAATTCCACAACTCTTGTGCTGTGGAGACTCTACTTTTCCTGGCA
TTGGAGTGCCTGCAGTTGCTGCTAGTGGTGCCATTGTTGCGAATTCGCTGGTTTCTGTG
GCAGAACATTCACAGCTTCTTGATGCTGTAGGGATATGA

>CRTISO2_Ntab0027300

ATGGGTACCTTGAATTTTATGTTTCCCAATTCACTTCTTGATGGTAACTGCAAGATTGT
AGCTTTGGGTGATAGCAAACTCAGAAACAATAAAAAGATAAGTTCTTGTTTTGACCCT
TTGAGAATTGGGAATTGTACTGATGTCCAGCAGCTTAATGGCTTGAGTTTGGGGCTGA
ATAAGACTAAGGGAAGAAAAGGGGGTATTGTTTCACAGTTGAAAGCAGTTGTAGATA
AAGGAGTGGAGAGCTATGGAGTTGGTGAGGTAGAAGTAAATGAGAGTGGCAACTGT
GATGCCATTATTATAGGGTCAGGAATAGGTGGATTAGTGGCAGCAACACAGCTAGCA
GTTAAGGGAGCTAAGGTTTTGGTTTTGGAGAAGTATGTTATTCCTGGTGGAAGCTCTG
GCTTTTACCAGAGGGATGGTTATACTTTTGATGTTGGTTCATCAGTCATGTTTGGATTC
AGTGATAAGGTTAGTTTGTCTGTATACTTTCTTTCTGTTATTTAGATGTCCAAGAAGTA
GTATTGATTAGGACCAAGATTTTGAAGCAATGATAACCTTTGATATGCGGTCAGTGCT
TTGAAATAGAAAAATCGTTCATAATAATAAAAGATGCTTTAGTTTGGCTCAAGACAG
TTGTACTTGTTTGCATCTCAAACGTACCCGCACATGGTGTTTGCACTAAACTAATGAA
TGCAAGACATGTGTCTTTTACCGCTGATGGTGCAGAATTTCTGTCATTATTAGTTTATT
ATAGCCCTTCATAGTACTATATTTCCCTCTCATGTTGTCTTCTGTTGTTTGATTCTTGAA
ATCAGTTTTTCTTGGGTGCCTTAGCTATGTATACAAGTACTCTTCTAACTATTTTTCTTT
TGGATGATATGGAACTACACCTGAAATACTATCTGCTGGTTTATAAGAAAATAATGC
AAACTTTTTGAGTTGTAAATCCATTTAACTAACACACTAGTCTAGGAGAAGTATCATA
GATATATCTGATGCTTAAGGACGGATCAGTTATAAGTTTCCTTGCATTTTTCCCAAATT
TAGGTCTTCCTAGAATTTGTTTGATAACAACTGTTAAACTATGATACAAGGTCTTCTTC
CCATATGGTTTCCTGCCCCCCAGCCTTCTTTTGTGATGACATTGCGAAAACAAGAGAA
TAGATTTTGACAATTCGCAGATACAGTGTCTTATGCCTCGTGCTTTTACATATGCCATG
CTATAGAGTAACAAATTCTGAAGAATAGCATTGTAGAAAACCTATTTACACATGTGC
ATATACTCTGTAACATTTTGGGTCAAGTTGGTGAATACATGTCAAATGTCCCAAGCTT
ACTGTATATGTAGCAACTAATTAACAGACTGACAAGATATTTCAAGTAATTGTCTGCA
GTTGGTTGGTTAATTTAACAAAGCTGGTGATGTCCCCAAAAACCATCTTTTCTCCTATG
CAGTTAGTCCTTCCAGTATTTGATGTATGTGTCGTGCATATAATGTTCTTATTCTGATGT
CTCTCTTCAAACTTCAGTTTCAAGAACAGTTGTTTCACTGCATCAGTTCACATGTTGCT
GTCGTCATCACTTGTTATCTTACCATAGTCCTTATCTGGCAATTATATTTCAGTTCTTAC
ATGAACCATCAACAAGTACACAATAACAATACCCATAGTTGGAACGTCTATCAAAAG
GTGCACCTACCTAGTCTGCACTAGTATAGCTAACAATGTGTTCATGTTTTGTATGACCT
CATTTCCTGGAAGATGTGTACAAGAATGAAAATCATAGCATCCTATTGAATATCACAT
GGGAATTCGCAAACCAACGTACTATGTTCACAACAAAGCATTGTTAGGTTGTGTTGTA
GTGAGGTAATTCCACCTTGTCGACCATCTATCTACATTAACCTGGATCAATGAGGGGC
TTTTCTACCCCAGCTGGAGCTCTAAATTTAGATTTAATAAGTGCCAGTGGGTCGAATA
TTTCCATTCCTTCGTCGAATATGTCTGATGCATGTTTTATGCTCTAGATTGGGCCGTGTT
GATGCCTCGGAAGTATCTCAATCTTTCCGAGCTTCTTAGTCTGAAAATACTGGAAGAG
ACTCTGTTTCAATTGAAGATGTCATCCCAATAACTACATAATTCTATCATTAACATAA
TTTGTACTGGCAGTGGTGTATACTTTGTTCTGTTGTATGTGGTCTGCAGTGCTTGCATGT
GATTGGATGTTTCACATCTTTTGACAGGGAAACCTCAATTTGATAACTCAAGCATTGG

CAGCAGTAGGATGTAAATTAGAAGTTATACCTGACCCAACAACTGTGCATTTCCACCT
ACCCAATGACCTTTCTGTCCGTGTACACCGAGAATATGATGACTTCATTCAAGAGCTT
GTGAGTAAATTTCCACATGAAAAAGAAGGGATTATCAAATTCTACAGTGAATGCTGG
AAGGTTTGTGGTCATTAAAATTAAGGTGACTGCAGCTTGCTTTAAATTTTGGACTTCCT
GATTTAGACTCAAAATCATTTGTTGAATATGGATGCTCAGATCTTTAATTCTCTGAATT
CTTTGGAACTGAAGTCTTTGGAGGAACCCATCTACCTTTTTGGCCAGTTTTTTAAGAAG
CCCCTCGAATGCTTGACTCTCGGTAATTTCTTTTCTCTTTTCCTTTTAACTTCAAATGTT
GTATTTCCTTAGCTGTGCCTTCAGTCCTAATGCTAGAAATGGGTAAAAGAGGTAGTCT
AGTGCACTAAGCTCCCGCAATGCTAGAAATGGATGCAGAGAATTGTTTCAAATTGAC
TTTGTTTCATGCCCTTTTATTGGTATACAAATTTACATTAATAGCAGACAAAGTCCAGA
GAAATGCTGTAACTCACACGATAACTGTAGTTGCATAATTAAACAGATGTTCAAGTG
ATAGTGTTGAAAAAGCTTGTAGAACTCAGTAGTTGCCAAAATTTAATACAGTTCCGA
ATACAGATTACTATGATTTTTTACTCTGACCTTTTTCTCAATAATCAAGAAATTCATGA
CCAAAGATTGTAACATTTCCTATCTTAGGTGGCTTTTGTCATTTTCGCTGATCAAACAC
AGACCTCGTTCCACTGTATAACGTCAATTAACTTGGTATCCTTTTGCATAAAGATAAT
GAGATCTTATGCTATCAAATCACCCTTCTTGTGTTTAACAGTTCTGATTTACATACTTT
AAATTTTTTGTAGTGCATTTAGTTAGTTTTGCAGTCTGACTGATGGCTCATAAATCATG
GAAAATTCCTTCATAGATGTGTTAATACCCAAAAGCGTTCGACTTATGATAGGTTCTT
CCTGTCATTCTTGGCATTTGCCAATATCCATCTTGCGGACTAATTTAAGAATAACTCTC
CAATTATAGGGTCATTTTTTCATGCTGACATTTGTGCAGCACAGAATGATTGAATATC
CTTCTATTGAATCTTAATTTATGCCAATGTTATTCATCACAGCCTACTATTTGCCCCAG
AATGCTGGTGACATTGCTCGGAAGTATATAAAAGATCCCGGGTTGCTGTCCTTCATAG
ATGCAGAGGTGAGGCAAATAATCTGTACAACTTACTAAAAGTTTTGATGATTTCTTTA
CCTTTTGAAGAATTCAACTATATTTTCTCTTTTGCAGTGTTTTATTGTGAGCACAGTTAA
TGCATTACAAACACCAATGATCAATGCAAGCATGGTAATTCTGCATTTAACCTTAGGC
CTTCTTGTGTTTATTGATGTCCATTTTCTCATGAGTTGCTTTCTGCAGGTTCTATGTGAC
AGACATTTTGGAGGAATCAACTATCCCGTGGGTGGAGTTGGCGAGATTGCCAAATCT
TTAGCAAAAGGCTTGGTTAATCAGGGAAGTCAGATACTTTATAGGGCAAATGTCACA
AGTATCATTTTGGACAACGGCAAAGCTGTGAGTTTTGGTCCTAGAACCATTATGAAAT
TATTGTTAAGAGTGATTATTCCTATTGTGATATGCAAAACTGTTGTTCTGGTGTTCGTA
TTGGACATGTCTTTGCCGACACTCTGTTAGACATACAGAGACACATGTCACACACACT
TTTAACCATCGTCAAATTGTCTTTCCACTGTTTGACCATCAAAAACACTTGTCCTATAT
AGGTCTTTGGTATGCCTAACTGCATGACGAGCCATTTTAAGATTTAAGGTAATATTAG
CGTGTTAGGATGTTAAAATATTTTTAAAATTCGGTAGTAGTATATAGCTAAAGAGGAA
AAATGTGAGGACAAAGAAGGAAGAGGGAAGACCAAACATCTGATGTTAAAATGTTA
TACGTTTTCTATACTTGATTGTAATTGATATATACTGTGATATTTTACTTATCATTTGGG
TAGGTCCTACATCCCCTTTAGTTGGATGTCAGTTACGCCCCTGAAAATACAGAAAGTG
TCTACATATTTATACATACATGACTAAATTATACGTAACAATATAATGAAAATTGTAT
TTTTCAATGTCCTAATCGTATCTTGTTTTGCATTTTCGAATGCTTGTTCGCCAATGTGCA
TGCTAGGCCATTTTCTCAAACTCTTCTTTTTCTTTTAGGTGGGAGTGAAACTTTCCGAT
GGAAGGAAGTTTTATGCTAAAACCATAGTATCGAATGCCACCAGATGGGATACTTTT
GGTTAGTTTTCCTAGAAGCCAGCATAATTTATATGTTTTCTTAAGATTCTTTTATCTTAG
AGCTAGTACTGTGGTTTTCTTGTAAACATTTGATACTCTTCAAATCTTGTCTTCTCATGT
CAAGTGTCTTACATTGTAGGAAAGCTTTTAAAAGCTGAGAATCTGCCAAAAGAAGAA

GAAGCTTTCCAGAAAGCTTATGTAAAAGCACCTTCTTTTCTTTCTATTCACATGGGGGT
TAAAGCAGATGTTCTCCCTCCAGACACTGATTGCCACCATTTTACCCTTGAGGTACTA
TTGGCACTTCAACGGGTTTGTTATCTATCTTTTCCTGTTTAAGGCTCACATTCATAATCT
CATGATTTTACAGGATAATTGGACAAATTTGGAGAAACCGTATGGAAGTATATTCTTG
AGCATTCCTACTGTTCTTGATTCATCATTGGCCCCAGAAGGACATCATATTCTTCACAT
TTTTACAACGGCGAGCATAGAAGATTGGGAGGTAAAATTAGTATATGCCTTAAGTGT
CTAAGGTAACCTCTAGTGTATCTATGTCATCCTTTTTCTTTTTACTGGAAACTAAAGTA
CGGATGTTTTTCCTTACTAGGGACTCTCTCTGAAAGATTATGAAGCAAAGAAAGAGCT
TGTTGCTGAAAGAATTATAGGCAGACTTGAAAAGACACTCTTCCCAGGGCTCAAGTC
ATCTATTGTTTTTAAGGAGGTATAAGTCATGATTTTATGAACTGAATAGTTGGTCATAG
TGAACAATAATGAGCTTTCTTCAGTACCAAATCGACACGCTAAAATAGTTCATGCCTT
GGGGCTTCATCCATTTATATTTATGATTGGACTCTTGTAGGTGGGAACACCAAAGACA
CATAGACGATACCTTGCTCGTGATAGTGGTACCTATGGACCAATGCCACGCGGAACT
CCAAAGGGATTACTAGGAATGCCTTTCAATACCACTGTAAGTGAATCATAACTTCAGT
ATCTAGTTGCTTTACTGTTCTACTGGTAAATAGAAGCGCACAGAATAAACTGTTCTGT
ATGGTTTCTGAGCTTACAGGCTATAGATGGTCTATATTGTGTTGGCGATAGTTGCTTTC
CTGGACAAGGTGTTATAGCTGTAGCCTTTTCAGGAGTAATGTGTGCTCACCGAGTTGC
TGCTGACTTAGGTAAACATGGTTTGACAAGATATTCAATTTTCAGAACTTGAAGGTCC
AGATTGCATTTGTCGTGATATCTTTATTACACATAGCTCTACTATATTTTACTCTGTGAC
CTTCAAAATGTTCTCGCTAGGGAGGTATTGACTTAATATGTGCCAATCAGCAATAAGT
GGTCTTATTACTTTGGTTATTGATGAAAAGTTTTGTTTGATATCCTATAATCTAATATA
ATATGCTCATCGATATGGAATTTACGTATCTTATATTTAGTTTATATCCATATTTCCATA
AAGTCTGATTTTGATATCCTCGTTTTTCTGTCACAGGATTTGAGAAAAAATCAGAGGT
GCTGGACAGTGGTCTTCTTAGACTACTCTGTTGGTTAAGGACACTAGCATGA

>mRNA_122944_cds

ATGGGTACCTTGAATTTTATGTTTCCCAATTCACTTCTTGATGGTAACTGCAAGATTGT
AGCTTTGGGTGATAGCAAACTCAGAAACAATAAAAAGATAAGTTCTTGTTTTGACCCT
TTGAGAATTGGGAATTGTACTGATGTCCAGCAGCTTAATGGCTTGAGTTTGGGGCTGA
ATAAGACTAAGGGAAGAAAAGGGGGTATTGTTTCACAGTTGAAAGCAGTTGTAGATA
AAGGAGTGGAGAGCTATGGAGTTGGTGAGGTAGAAGTAAATGAGAGTGGCAACTGT
GATGCCATTATTATAGGGTCAGGAATAGGTGGATTAGTGGCAGCAACACAGCTAGCA
GTTAAGGGAGCTAAGGTTTTGGTTTTGGAGAAGTATGTTATTCCTGGTGGAAGCTCTG
GCTTTTACCAGAGGGATGGTTATACTTTTGATGTTGGTTCATCAGTCATGTTTGGATTC
AGTGATAAGGGAAACCTCAATTTGATAACTCAAGCATTGGCAGCAGTAGGATGTAAA
TTAGAAGTTATACCTGACCCAACAACTGTGCATTTCCACCTACCCAATGACCTTTCTG
TCCGTGTACACCGAGAATATGATGACTTCATTCAAGAGCTTGTGAGTAAATTTCCACA
TGAAAAAGAAGGGATTATCAAATTCTACAGTGAATGCTGGAAGATCTTTAATTCTCTG
AATTCTTTGGAACTGAAGTCTTTGGAGGAACCCATCTACCTTTTTGGCCAGTTTTTTAA
GAAGCCCCTCGAATGCTTGACTCTCGCCTACTATTTGCCCCAGAATGCTGGTGACATT
GCTCGGAAGTATATAAAAGATCCCGGGTTGCTGGCCTTCATAGATGCAGAGTGTTTTA
TTGTGAGCACAGTTAATGCATTACAAACACCAATGATCAATGCAAGCATGGTTCTAT
GTGACAGACATTTTGGAGGAATCAACTATCCCGTGGGTGGAGTTGGCGAGATTGCCA
AATCTTTAGCAAAAGGCTTGGTTAATCAGGGAAGTCAGATACTTTATAGGGCAAATG

TCACAAGTATCATTTTGGACAACGGCAAAGCTGTGGGAGTGAAACTTTCCGATGGAA
GGAAGTTTTATGCTAAAACCATAGTATCGAATGCCACCAGATGGGATACTTTTGGAA
AGCTTTTAAAAGCTGAGAATCTGCCAAAAGAAGAAGCTTTCCAGAAAGCTTATG
TAAAAGCACCTTCTTTTCTTTCTATTCACATGGGGGTTAAAGCAGATGTTCTCCCTCCA
GACACTGATTGCCACCATTTTACCCTTGAGGATAATTGGACAAATTTGGAGAAACCGT
ATGGAAGTATATTCTTGAGCATTCCTACTGTTCTTGATTCATCATTGGCCCCAGAAGG
ACATCATATTCTTCACATTTTTACAACGGCGAGCATAGAAGATTGGGAGGGACTCTCT
CTGAAAGATTATGAAGCAAAGAAAGAGCTTGTTGCTGAAAGAATTATAGGCAGACTT
GAAAAGACACTCTTCCCAGGGCTCAAGTCATCTATTGTTTTTAAGGAGGTGGAACA
CCAAAGACACATAGACGATACCTTGCTCGTGATAGTGGTACCTATGGACCAATGCCA
CGCGGAACTCCAAAGGGATTACTAGGAATGCCTTTCAATACCACTGCTATAGATGGT
CTATATTGTGTTGGCGATAGTTGCTTTCCTGGACAAGGTGTTATAGCTGTAGCCTTTTC
AGGAGTAATGTGTGCTCACCGAGTTGCTGCTGACTTAGGATTTGAGAAAAAATCAGA
GGTGCTGGACAGTGGTCTTCTTAGACTACTCTGTTGGTTAAGGACACTAGCATGA

>CRTISO3_Ntab0736080

ATGGGTACCTTGAATTTTATGTTTCCCAATTCACTTCTTGATGGTAACTGCAAGATTGT
AGCCTTGGGTGGTAGCAAACTCAGAAACAGTAAAAAGATAAGTTCTTGTTTTGACCC
TTTGAGAATTGGGAATTGTACTGATGTCCAGCAGCTTCATGGCTTGAATTTGGGGCTG
AATAAGACTAAGGGAAGGAAATGGGGTATTGTTTCACAGTTGAAAGCAGTTGTAGAT
AAAGGAGTGGAGAGCTATGGAAGTAGTGATGTAGAAATAGATGAGAGTTGCTGCTAT
GATGCCATTGTAATAGGGTCAGGAATAGGCGGATTAGTGGCAGCAACACAGCTAGC
AGTTAAGGGAGCTAAGGTTTTAGTTTTGGAGAAGTATGTTATTCCTGGTGGAAGCTCT
GGGTTTTACCAGAGGGATGGTTATACTTTTGATGTTGGTTCTTCAGTCATGTTTGGATT
CAGTGATAAGGTTAGTTTGTCTGTATACTTTCTTTCTGTTTGGATTCAGTCATAGTCCTT
CATACTGAATAGTACCTCTATATTTCTCATGTTGTCATCTGCTGTTTGATTCTTGAAATC
AGTTTTTCTTGGGTGCCTTAGCTATATACACAAGTACTCTTCTAACTATTCCTCTTTTGG
ATGATATGGGACTACACCTGAAATATTATCTGCTGGTTTAGAAGAAAATATTGCAAA
CTTTTCTAGTTGTAAATCCGTTTGAATGACGCACTAGTCTAGGAGAAATATCTGATAC
TTAAGGACGGATCAGTTATAAGTTTCCTTGCATTTTCCCAAATTTAGGTCCTCCTAGAA
TTTGGTTGATAACAACTGTTAAACTATGATACAAGGTCTTCTTCCCATATGGTTTCCTG
CCCCCAGCCTTCTTTTGTGATGACATTGCAAAAACAAGAGAATAGATTTTGACAATTC
GCAGATACAATGCTTTATGCCTCGTGCTTTTATATATGCCATGCTATAGAGTAACAAA
ATTCTGAAGAATAGCACTGTAGAAAACCTATTTACCCATGTGCATATACTCTTTAACA
TTTTGGGTCAAGTTGGTGCATACATGTCAAATGTCCCAAGCTTGCTGTATATGTAACT
AATTAACAGACTGACAAGAGACTTCAAGTAATTGTCTGCAGTTGGTTGGTTAATTTAA
CAAAGCTGGTGATGTCCCCAAATATCATATTTTCTCCTATGTGGTTAGTCCTTCCAGTA
TTTGATGTATGTGTAGTGCATATAATGTTCTTATTCCGATCTATCTCTTCAAACTTCAGT
CCCAAGAATAGTTGTTTCATTGCATCAGTTCACATGTTGCTGTTGTCATCACTTGTTAT
CTTACCATAGTCCTTATCTGGCAATTATATTTCAGTTCTTACTTTTCATGGTACCATCAA
CAAGCACACGATACCCATAGGTGGAACGTCTATCAAAAGGTGCACGTACCAAGTCTG
CACTAGTATAGCTAACAATGTGTTCATGTTTTGTATGACCATACTATGGAGTAGTTCAT
TTCCTGGAAGACGTGTACAAGAATGAAAATAATAACATCCTATTGAATATCACATGG
GAATTCGTAAACCAACGTACAATGTTCACAACAAAGCATTGTTAGGTTGGGTTGTAGT

GAGGTAATTCCACCTTGCCGACCATCTATCTACATTAATCTGGATCTATGAGGGGCTT
TTCTACCCCAGCTGGAGCTCTAAATTTAGATTTAATAAGTGCGAGTGGTCAAATATTT
CCATCCCTGTTTCCTCGAGTATGTCTGATGCATGTTTTATGCTCTAGATTGGGTCATGTT
GATGCCTCGGAAGTATCTCAATCTTTTCCGAGCTTCTTGGTCTGAAAATGCTGGAAGA
GACTCTGTTTCAATTGAAGAATGTCATCCCAATAACTACCTATGATAACTCTGTCATT
AACATAATTTGTACTGGCAGTGGTGTATGCTTTGTTCTGTTGTATGTGGTCTGCGGTGC
TTACATGTGATTGGATGTTTCACATATTTTGACAGGGAAACCTCAATTTGATAACTCA
AGCATTGGCAGCAGTAGGATGTAAATTAGAAGTTATACCTGACCCAACAACTGTGCA
TTTCCACCTACCCAATGACCTTTCTGTCCGTGTACACCGAGAATATGATGACTTCATTC
AAGAGCTTGTGAGTAAATTTCCACATGAAAAAGAAGGGATTATCAAATTCTACAGTG
AATGCTGGAAGGTTTGTGGTCATTAACATTAAGGTGACTGCAGCTTGCTTTAAATTTT
GGACTTCCTGATTTAGACTCAAAATCATTTGTTGAATATGGATGCACAGATCTTTAGTT
CTCTGAATTCTTTGGAACTGAAGTCTTTGGAGGAACCCATCTACCTTTTTGGCCAGTTT
TTTAAGAAGCCCCTCGAATGCTTGACTCTCGGTAATTTCTTTTCTCTTTTCCTTTTAACT
TCAAATGTTATATTTCTTTAGCTGTGCCTTCAGTCCTAGTGCTAGAAATGGGTAAAAG
AGGCAGTCTAGTGCACTAAGCTCCCGCAATGCTAGAAATGGATGCAGAGAGTTGTTT
CAAATTGACTTTGTTTCATGTCCTTTTATTGGTATACAAATTTAAATTAATAGCAGGAC
AAGTCCAGAGAGATGCTGTATCTCGCACGATAACTGTAGTTGCATAATTAAACATAT
GTTCAAGTGATAGTGTTGAAAAAGCTTGTAGAACTCAGTAGTTGCCAAAATTTAATAC
AGTTCTGAATACAGATTACTCTGATTTTTACTCTGACCTTTTTCTCAATAATCAAGAA
ATTCATGACCAAAGATTGTAACATTTCCCATCTTAGGTGGCTTTTGTCATTTTCGCTGA
TCAAACACAGACTTCGTTCCACTGTATAACGCCAATTAACTTGGTATCCTTTTGCATA
AAGATAATGAGATCTTATGCTATCAAATCACCCTTCTTGTGTTTAACAGTTCTGATTTA
CATGCTTTAAATTTTTGTAGTGCATTTACTTAAATTTGCAGTCCGACTGATGGCTCATA
AATCATGGAAAATTCCTTCATAGATGTGTTAATACCCAAAAGCGTTCTACTTATGATA
GGTTCTTCCTGTCATTATTGGCATTTGCCAATATCCATCTTGCGGACTAATTTAAGAAT
AACTCTCCAATTATAGGGTCATTTTTTCATGCTGACATTTGTGCAGCACAGAATGATTG
AATATCCTTCTATTGAATCTTAATTTATGCCAATGTTATTCATCACAGCCTACTATTTG
CCCCAGAATGCTGGTGACATTGCTCGGAAGTATATAAAAGATCCCGGGTTGCTGGCC
TTCATAGATGCAGAGGTGAGGCAAATAATCTGTACAACTTACTAAAAGTTTTGATGAT
TTCTTTACCTTTTGAAGAATTCAACTATATTTTCTCTTTTGCAGTGTTTTATTGTGAGCA
CAGTTAATGCATTACAAACACCAATGATCAATGCAAGCATGGTAATTCTGCATTTAA
CCTTAGGCCTTCTTGTGTTTATTGATATCCATTTTCTCATGAGTTGCTTTCTGCAGGTTC
TATGTGACAGACATTTTGGAGGAATCAACTATCCCGTGGGTGGAGTTGGCGAGATTG
CCAAATCCTTAGCAAAAGGCTTGGTTAATCAGGGAAGTCAGATACTTTATAGGGCAA
ATGTCACAAGTATCATTTTGGACAACGGCAAAGCTGTGAGTCTTGGTCCTATAACCAT
TGTGAAATTATTGTTAAGAGTGTTTATTCCTATTGTGATATGCAAAACTGTTGTTCTGG
TGTTCGTATTGGACATGTCTTTGCCGACACTCTGTTAGACATACAGTAACACATGTCA
CACACACTTTTAACTATTGTCAAATCGTCTTTCCACTGTGAGTCTTGGTCCTAGAACCA
TTGTGAAATTATTGTTAAGAGTGTTTATTCCTATTGTGATATGCAAAACTGTTGTTCTG
GTGTTCGTATTGGACATGTCTTTGCCGACACTCTGTTAGACATACAGTAACACATGTC
ACACACACTTTTAACTATCGTCAAATCGTCTTTCCACTGTTTGACCATCAAAAACACTT
GTCCTATATAGGTCCTCGGTATGCCTAACTGCATCTAGACGAGCCGTTTTAAGATTTA
AGGTAATATTAGCATGTTAGGATGTTAAAATATTTTTAAAATTGGGTAGTAGTATATA

GTAAAAGATGAAAAGTGTGACGACAACGAAGGAAGAGGGAAGACCAAACATCTGT
ATGTTAAAATGTTATACGTTTCTATACTTGATTGTAACTGATATATATTGTGATATTTTA
CTTATCATTTGGGTAGGTCCTACATCCCCTTTAGTTGGATGTCAGTTACGCCCCTGAAA
TTATTATACAGAAAGTGTCTACGTATTTATACATACATGACCAAATTACGTAACAATA
TAATGAAATTGTATTTTTCAATGTCCTAATCATGTCTTGTTTCGCATTTGTCAATGTTGT
GTTCGCCAGTGTGCATGCCAGGCCATTTTCTCAAACTCTTCTTTTTCTTTTAGGTGGGA
GTGAAGCTTTCCAATGGAAGGAAGTTTTACGCTAAAACCATAGTATCAAATGCCACC
AGATGGGATACTTTTGGTTAGTTTTCCAGGAAGCCAGCATAATTCATGTGTTTTCTTAA
GATTCTTTTGTCTTAGAGCCAGTACTGCGGTTTTCTTGCACACATTTGATTCTCTTCAAA
TCTTGTCTTCTCATGTCAAGTGTCGTACATTGTAGGAAAGCTTTTAAAAGCCGAGAAT
CTGCCAAAAGAAGAAGAAGCTTTCCAGAAAGCTTATGTAAAAGCACCTTCCTTTCTTT
CTATTCACTTGGGCGTTAAAGCAGATGTTCTTCCGCCAGACACTGATTGCCACCATTTT
ACCCTTGAGGTACTATTGGCACTTCAATGGGTTTGTTGTTTATTTTTTCCTGTTTAAAGC
TCACATTCACAATCCCTTGATTTTACAGGATAATTGGACAAATTTGGAGAAACCATAT
GGAAGTATATTCTTGAGCATTCCTACAGTTCTTGATTCATCATTGGCCCCAGAAGGAC
ACCATATTCTTCACATTTTTACAACGTCAAGCATAGAAGATTGGGAGGTAAAATTACT
ATATGCCTTCAGTGTCGAAAGTAACCTCTTGTGTATCTTTGTCATCGTTTTTTTTTAAAA
AATGGAAATTGAAGTTGAGGTGTTTTTACTTACTAGGGACTCTCTCTGAAAGACTATG
AAGCAAAGAAAGAGCTTGTTGCTGAAAGAATTATAAGCAGACTTGAAAAGACACTC
TTCCCAGGGCTCAAGTCATCTATCGTCTTTAAAGAGGTACAAGTCATGATTTTATGAA
CTGAATAGTTGGTCATAGTGAACAATAATTAGCTTTCTTCAGTACCAAATCGACACGC
ATCTTATGCTTGCTAAAATAGTTCGTGCCTTGGGGCTTCATCCATTTATAATAATGATT
GGACTCTTGTAGGTGGGAACACCAAAGACACATAGACGATACCTTGCTCGTGATAGT
GGTACCTATGGACCAATGCCACGCGGAACTCCAAAGGGATTACTTGGAATGCCTTTC
AATACCACTGTAAGTTAATTATTCCTTCTGTATCTAGTTGCTTCACTGCTCTACTGGTA
AATAGAAGCGCACAGAATAAACTGTTCCGTATGGTTTCTGAGCTTACAGGCTATAGA
TGGTCTATATTGTGTTGGCGATAGTTGCTTTCCTGGACAAGGTGTTATAGCTGTTGCCT
TTTCAGGAGTAATGTGTGCTCACCGAGTTGCTGCTGACTTAGGTAAACATGGTTTGAC
AAGATATTCAATTTTCAGAACTTGAAAGTCCTGATTGCATTTGTCGCGATATTTTTATT
ACACATATCACTAGCTCTACTATAATTTGCTCTGTGGCCTTCAAAAGGTTCCCGCTAG
GGAGGTATCGACTTAATATATGCCAATCGGCAATAAGTGGTCTTATTACTTTGTTTATT
GATGAAAAGTTTTATTTGACATCCAATAATCTAATACAATATGCTCAACAATATGGAA
TTTATGTACCTTATGTTTAGTTTATATCCATATCACCATAAAGTAACTGTGTCTGATTTT
GATATCCTCTTTTTTCTGTCACAGGATTTGAGAAAAAATCAGAGGTGCTGGACAGTGG
TCTTCTTAGACTACTCGGTTGGTTAAGGACACTAGCATAA

>mRNA_78351_cds

ATGATCAATGCAAGCATGGTTCTATGTGACAGACATTTTGGAGGAATCAACTATCCCG
TGGGTGGAGTTGGCGAGATTGCCAAATCCTTAGCAAAAGGCTTGGTTAATCAGGGAA
GTCAGATACTTTATAGGGCAAATGTCACAAGTATCATTTTGGACAACGGCAAAGCTG
TGGGAGTGAAGCTTTCCAATGGAAGGAAGTTTTACGCTAAAACCATAGTATCAAATG
CCACCAGATGGGATACTTTTGGAAAGCTTTTAAAAGCCGAGAATCTGCCAAAAGAAG
AAGAAGCTTTCCAGAAAGCTTATGTAAAAGCACCTTCCTTTCTTTCTATTCACTTGGGC
GTTAAAGCAGATGTTCTTCCGCCAGACACTGATTGCCACCATTTTACCCTTGAGGATA

ATTGGACAAATTTGGAGAAACCATATGGAAGTATATTCTTGAGCATTCCTACAGTTCT
TGATTCATCATTGGCCCCAGAAGGACACCATATTCTTCACATTTTTACAACGTCAAGC
ATAGAAGATTGGGAGGGACTCTCTCTGAAAGACTATGAAGCAAAGAAAGAGCTTGTT
GCTGAAAGAATTATAAGCAGACTTGAAAAGACACTCTTCCCAGGGCTCAAGTCATCT
ATCGTCTTTAAAGAGGTGGGAACACCAAAGACACATAGACGATACCTTGCTCGTGAT
AGTGGTACCTATGGACCAATGCCACGCGGAACTCCAAAGGGATTACTTGGAATGCCT
TTCAATACCACTGCTATAGATGGTCTATATTGTGTTGGCGATAGTTGCTTTCCTGGACA
AGGTGTTATAGCTGTTGCCTTTTCAGGAGTAATGTGTGCTCACCGAGTTGCTGCTGACT
TAGGATTTGAGAAAAAATCAGAGGTGCTGGACAGTGGTCTTCTTAGACTACTCGGTTG
GTTAAGGACACTAGCATAA

>β-LCY1_Ntab0268950

ATGGATACATTGTTGAAAACCCCAAATAAGCTTGAGTTTCTGCACCCAGTTCATGGAT
TTTCTGTTAAAGCTAGCTCCTTTAACTCTGTAAAGCCCCATAAGTTTGGTTCTAGGAAA
ATTTGTGAAAATTGGGGTAAAGGGGTTTGTGTTAAGGCTAAGAGTAGTGCCCTTTTGG
AGCTTGTACCTGAGACCAAAAAGGAAAATCTTGATTTTGAGCTTCCTATGTATGACCC
TTCAAAAGGTCTTGTTGTAGATCTAGCTGTGGTTGGTGGTGGACCCGCTGGACTTGCA
GTTGCACAGCAGGTTTCGGAGGCTGGACTATCGGTTGTTTCAATCGATCCATCGCCGA
AATTGATATGGCCCAATAACTATGGTGTTTGGGTGGATGAATTTGAGGCCATGGATTT
GTTGGATTGCCTCGACGCCACATGGTCAGGTACTGTTGTTTATATTGATGACAATACA
ACTAAAGATCTTGATAGACCTTATGGAAGGGTTAATCGGAAACAACTTAAGTCCAAA
ATGATGCAGAAATGCATACTAAACGGTGTTAAATTCCACCACGCCAAAGTTATAAAG
GTAATTCACGAGGAAGCTAAATCTATGCTGATTTGCAATGATGGTGTAACTATTCAGG
CAACGGTGGTGCTTGATGCAACTGGCTTCTCAAGATGTCTTGTTCAGTATGATAAGCC
ATATAATCCTGGATATCAAGTAGCTTATGGCATATTGGCAGAAGTGGAGGAACATCC
CTTTGATACAAGTAAGATGGTTCTCATGGATTGGCGAGATTCGCATCTTGGTAATAAT
ATGGAGCTGAAGGAGAGAAATAGAAAAGTTCCAACTTTTTTGTATGCCATGCCATTTT
CATCAAATAAAATATTTCTTGAAGAAACCTCACTTGTTGCTCGTCCTGGATTACGTAT
GGACGATATTCAAGAAAGAATGGTGGCTCGTTTAAATCACTTGGGTATAAAAGTTAA
GAGCATTGAAGAGGACGAGCATTGTGTAATTCCGATGGGAGGCTCCCTTCCTGTAAT
ACCTCAGAGAGTTGTTGGAACTGGTGGTACAGCTGGTCTGGTTCATCCCTCAACAGGT
TATATGGTAGCAAGGACCCTAGCTGCAGCTCCGGTCGTCGCTAATGCAATAATTCACT
ACCTTGGTTCTGAGAAAGACCTTTTAGGTAATGAGTTATCTGCAGCTGTTTGGAAAGA
TTTGTGGCCCATAGAAAGGAGACGTCAACGAGAGTTCTTTTGTTTCGGTATGGATATT
CTTCTGAAGCTTGATTTACCCGCTACAAGAAGGTTTTTCGATGCCTTTTTTGATCTAGA
ACCTCGTTATTGGCATGGCTTCTTGTCATCTCGCCTGTATCTTCCTGAGCTTATATTTTT
CGGGCTGTCCCTTTTCTCTCGCGCTTCAAATACTTCTAGAATAGAGATTATGACAAAG
GGAACTCTTCCTTTGGTAAATATGATCAACAATTTGTTACAGGATACAGAATGA

>mRNA_46713_cds

ATGGATACATTGTTGAAAACCCCAAATAAGCTTGAGTTTCTGCACCCAGTTCATGGAT
TTTCTGTTAAAGCTAGCTCCTTTAACTCTGTAAAGCCCCATAAGTTTGGTTCTAGGAAA
ATTTGTGAAAATTGGGGTAAAGGGGTTTGTGTTAAGGCTAAGAGTAGTGCCCTTTTGG
AGCTTGTACCTGAGACCAAAAAGGAAAATCTTGATTTTGAGCTTCCTATGTATGACCC

TTCAAAAGGTCTTGTTGTAGATCTAGCTGTGGTTGGTGGTGGACCCGCTGGACTTGCA
GTTGCACAGCAGGTTTCGGAGGCTGGACTATCGGTTGTTTCAATCGATCCATCGCCGA
AATTGATATGGCCCAATAACTATGGTGTTTGGGTGGATGAATTTGAGGCCATGGATTT
GTTGGATTGCCTCGACGCCACATGGTCAGGTACTGTTGTTTATATTGATGACAATACA
ACTAAAGATCTTGATAGACCTTATGGAAGGGTTAATCGGAAACAACTTAAGTCCAAA
ATGATGCAGAAATGCATACTAAACGGTGTTAAATTCCACCACGCCAAAGTTATAAAG
GTAATTCACGAGGAAGCTAAATCTATGCTGATTTGCAATGATGGTGTAACTATTCAGG
CAACGGTGGTGCTTGATGCAACTGGCTTCTCAAGATGTCTTGTTCAGTATGATAAGCC
ATATAATCCTGGATATCAAGTAGCTTATGGCATATTGGCAGAAGTGGAGGAACATCC
CTTTGATACAAGTAAGATGGTTCTCATGGATTGGCGAGATTCGCATCTTGGTAATAAT
ATGGAGCTGAAGGAGAGAAATAGAAAAGTTCCAACTTTTTTGTATGCCATGCCATTTT
CATCAAATAAAATATTTCTTGAAGAAACCTCACTTGTTGCTCGTCCTGGATTACGTAT
GGACGATATTCAAGAAAGAATGGTGGCTCGTTTAAATCACTTGGGTATAAAAGTTAA
GAGCATTGAAGAGGACGAGCATTGTGTAATTCCGATGGGAGGCTCCCTTCCTGTAAT
ACCTCAGAGAGTTGTTGGAACTGGTGGTACAGCTGGTCTGGTTCATCCCTCAACAGGT
TATATGGTAGCAAGGACCCTAGCTGCAGCTCCGGTCGTCGCTAATGCAATAATTCACT
ACCTTGGTTCTGAGAAAGACCTTTTAGGTAATGAGTTATCTGCAGCTGTTTGGAAAGA
TTTGTGGCCCATAGAAAGGAGACGTCAACGAGAGTTCTTTTGTTTCGGTATGGATATT
CTTCTGAAGCTTGATTTACCCGCTACAAGAAGGTTTTTCGATGCCTTTTTTGATCTAGA
ACCTCGTTATTGGCATGGCTTCTTGTCATCTCGCCTGTATCTTCCTGAGCTTATATTTTT
CGGGCTGTCCCTTTTCTCTCGCGCTTCAAATACTTCTAGAATAGAGATTATGACAAAG
GGAACTCTTCCTTTGGTAAATATGATCAACAATTTGTTACAGGATACAGAATGA

>β-LCY2_Ntab0383390

ATGGATACATTGTTGAAAACCCCAAATAAGCTTGAGTTTTTGCACCCACTTCATGGAT
TTTCGGTTAAAGCTAGCTCCTTTAACTCTGTAAAGCCCCATAAGTTTGGTTCTAGGAA
ATTTTGTGAAGATTGGGGTAAAGGGGTTTGTGTTAAGGCTAGTAGTAGTACCCTTTTG
GAGCTTGTACCTGAGACCAAAAAGGAAAATCTTGATTTTGAGCTTCCTATGTATGACC
CTTCAAAAGGTCTTGTTGTAGATCTAGCTGTGGTTGGTGGTGGACCCGCTGGACTTGC
GGTTGCACAGCAGGTTTCAGAGGCTGGACTATCGGTTGTTTCAATCGATCCATCGCCG
AAATTGATATGGCCCAATAACTATGGTGTTTGGGTGGATGAATTTGAGGCCATGGATT
TGTTGGATTGCCTTGACGCTACATGGTCAGGTACTGTTGTTTATATCGATGACAATAC
AACTAAAGATCTTGATAGACCTTATGGAAGGGTTAATAGGAAACAGCTTAAGTCCAA
AATGATGCAGAAATGCATACTAAACGGTGTTAAATTCCACCACGCCAAAGTTATAAA
GGTAATTCACGAGGAAGCTAAATCTATGCTGATTTGCAATGATGGTGTAACTATTCAG
GCAACGGTGGTTCTTGATGCAACTGGCTTCTCAAGATGTCTTGTTCAGTATGATAAGC
CATATAATCCTGGATATCAAGTAGCTTATGGCATATTGGCGGAAGTGGAGGAACATC
CCTTTGATACAAGTAAGATGGTTCTCATGGATTGGCGAGATTCGCATCTTGGTAATAA
TATGGAGCTGAGGGAGAGAAATAGAAAAGTTCCAACTTTTTTATATGCCATGCCATTT
TCATCGAATAGTATATTTCTTGAAGAAACCTCACTTGTTGCTCGTCCTGGATTGCGTAT
GGACGATATTCAAGAAAGAATGGTGGCTCGTTTAAATCACTTGGGTATTAAAGTTAA
GAGCATTGAAGAGGACGAGTATTGTGTAATTCCGATGGGAGGCCCTCTTCCTGTATTA
CCTCAGAGAGTTGTTGGAGTTGGTGGTACAGCTGGTATGGTTCATCCCTCAACAGGTT
ATATGGTAGCAAGGACCCTAGCTGCAGCTCCGGTCGTCGCTAATGCAATAATTCAAT

ACCTTGGTTCTGAGAAAGACCTTTTTGGTAATGAGTTATCTGCAGCTGTTTGGAAAGA
TTTGTGGCCCATAGAAAGGAGACGTCAAAGAGAATTCTTTTGCTTCGGTATGGATATT
CTTCTGAAGCTTGATTTACCCGCTACGAGAAGGTTTTTCGATGCCTTTTTTGATCTAGA
ACCTCGTTATTGGCATGGCTTCTTGTCATCTCGGCTGTATCTTCCCGAGCTTATATTTTT
TGGGCTGTCCCTTTTCGCTCGCGCTTCAAATACTTCCAGAATAGAGATTATGACAAAG
GGAACTCTTCCTTTGGTAAATATGATCAACAATTTGATACAGGATACTGAATGA

>mRNA_18729_cds

ATGGATACATTGTTGAAAACCCCAAATAAGCTTGAGTTTTTGCACCCACTTCATGGAT
TTTCGGTTAAAGCTAGCTCCTTTAACTCTGTAAAGCCCCATAAGTTTGGTTCTAGGAA
ATTTTGTGAAGATTGGGGTAAAGGGGTTTGTGTTAAGGCTAGTAGTAGTACCCTTTTG
GAGCTTGTACCTGAGACCAAAAAGGAAAATCTTGATTTTGAGCTTCCTATGTATGACC
CTTCAAAAGGTCTTGTTGTAGATCTAGCTGTGGTTGGTGGTGGACCCGCTGGACTTGC
GGTTGCACAGCAGGTTTCAGAGGCTGGACTATCGGTTGTTTCAATCGATCCATCGCCG
AAATTGATATGGCCCAATAACTATGGTGTTTGGGTGGATGAATTTGAGGCCATGGATT
TGTTGGATTGCCTTGACGCTACATGGTCAGGTACTGTTGTTTATATCGATGACAATAC
AACTAAAGATCTTGATAGACCTTATGGAAGGGTTAATAGGAAACAGCTTAAGTCCAA
AATGATGCAGAAATGCATACTAAACGGTGTTAAATTCCACCACGCCAAAGTTATAAA
GGTAATTCACGAGGAAGCTAAATCTATGCTGATTTGCAATGATGGTGTAACTATTCAG
GCAACGGTGGTTCTTGATGCAACTGGCTTCTCAAGATGTCTTGTTCAGTATGATAAGC
CATATAATCCTGGATATCAAGTAGCTTATGGCATATTGGCGGAAGTGGAGGAACATC
CCTTTGATACAAGTAAGATGGTTCTCATGGATTGGCGAGATTCGCATCTTGGTAATAA
TATGGAGCTGAGGGAGAGAAATAGAAAAGTTCCAACTTTTTATATGCCATGCCATTT
TCATCGAATAGTATATTTCTTGAAGAAACCTCACTTGTTGCTCGTCCTGGATTGCGTAT
GGACGATATTCAAGAAAGAATGGTGGCTCGTTTAAATCACTTGGGTATTAAAGTTAA
GAGCATTGAAGAGGACGAGTATTGTGTAATTCCGATGGGAGGCCCTCTTCCTGTATTA
CCTCAGAGAGTTGTTGGAGTTGGTGGTACAGCTGGTATGGTTCATCCCTCAACAGGTT
ATATGGTAGCAAGGACCCTAGCTGCAGCTCCGGTCGTCGCTAATGCAATAATTCAAT
ACCTTGGTTCTGAGAAAGACCTTTTTGGTAATGAGTTATCTGCAGCTGTTTGGAAAGA
TTTGTGGCCCATAGAAAGGAGACGTCAAAGAGAATTCTTTTGCTTCGGTATGGATATT
CTTCTGAAGCTTGATTTACCCGCTACGAGAAGGTTTTTCGATGCCTTTTTTGATCTAGA
ACCTCGTTATTGGCATGGCTTCTTGTCATCTCGGCTGTATCTTCCCGAGCTTATATTTTT
TGGGCTGTCCCTTTTCGCTCGCGCTTCAAATACTTCCAGAATAGAGATTATGACAAAG
GGAACTCTTCCTTTGGTAAATATGATCAACAATTTGATACAGGATACTGAATGA

>ε-LCY1_Ntab0006110

ATGGATTGTATTGGAGCTCGAAATTTTTCTACAATGGCGGTTTTTACGTGTCCGAGGTT
CAAATCATTAGGAAGAAAGAGAATTATGCCAAGAAAAAAGCAACCATTTTGGCCTA
TACATATGAAAGTGAAGTGTAGTGGAAGTGATAGTTGTGTAGTGGTTAAAGAAGATT
TTGCTGATGAAGAAGATTATATAAAAGCTGGTGGTTCAGAACTTGTTTTTGTTCAAAT
GCAGCAGAATAAAGACATGGATCTACAGTCTAAGCTTTCTGATAAGGTGATTTTCTTG
ATCCCATATTTAAAAGGATTTAACTTGTATACATTAAGGGGTCGTTTGGTTTGAAGAC
AAGTTATGTCAGGATCAGTTATGGTGTGATTAGTTATAGTGGGATTAGTTATGTTGGG
ATTAGTTATTATTTCTTATTGACTGTTTGGTATGTTCTATTAATCCTATGATTGTCAAAT

ATAATGTTTTATTCCCAGATAACTTATCCTATGATTACTATTCCAACCTCTGGTAGGTA
TAAGTTATCCCGATACTATTTTTAATTATGGGATAACTTATTCCGAGCTTAGTAACCAA
ATAAGGGATAAGGCGCTACTAAATTTTTATCCCATGATTATTTTTGCTAATCTATCGTA
CCATGCGACCCCTAGAAGGGAAGTTCGGTACACAAAGCATCCCGCATTAATATTCAC
GCAGGGTTTGGGTAAGGGCCGGGTCCGAAGGGATGTGATGTAGATAGCCTACCTAAT
ACAAGTATTAGTGGCTGCTTCCATAGTTTGAACCTTTGGTCACACGGAGACAACTTTA
CTGTTGTTGTAAGGTTATCCTTTAACTTGTATACATTGTCAGTATAAATTTTTTAACAAT
ATCTATGTAATTTAACCTGTTATAACAGGTTAATTGCTTTATTTTTTAGGTAATCAGTTC
CACTTATTGTAAATGTAGTTACCTATAGCTATCTTTAGGTAACTTGATAGTGTAAATAT
TCTTTTACACTTTCAGCTAATTAAAGTCAAATTCTGTTTAAAAAGGAAACAAACTTAT
AGTGTACAGTGGTAGTGTGACAAAATCTTACACTATCAGTGTATTTTACCTTATATTCG
CGATTACTAAACTATTCCAAATTACCACACATTGAATTCCATGTGAGTTAGAGTTATG
CAATATGAAAGAAATGAAGAAGCTAGAAAAACTACCATACTGCAGCAACTATGTCT
GCAATTTATTTTAACTCTTATATTGTAGCAAACTATATTGCTTTGTCCATCATACTATCT
CAGCTTGCAGTTGCGACAAATATCATCAGCTGGACAAACTATACTGGATTTAGTGGTC
ATTGGCTGTGGTCCTGCTGGTCTTGCTCTTGCTGCGGAGTCTGCTAAACTCGGGTTGAA
CGTGGGGCTCGTTGGTCCTGATCTTCCTTTCACAAATAACTATGGTGTCTGGGAAGAC
GAGTTCAAAGGTCGTAATCCTTATTTATCTTTTATGAAGAGTCCTTTTCTCTACATAAC
CTTTCAAGAAACAGAGCCTCTTGTTCTCTGGAGCTTAATTTAGACATATGCTTATACTT
TCCTTTTTCAGATCTTGGGCTTCAAGCATGCATTGAACATGTTTGGAGTGATACCATTG
TATATCTTGATGATGCCGATCCAATTCTTATTGGACGTGCTTATGGAAGAGTTAGTCG
CCATTTACTGCATGAGGAGTTACTCAAAAGGTTAAATTCACTGAACTTGTTCCTTATGT
CATACGAGTTGTTGATTTATTCCTTACAATTGAGGTTGTAATTCACATTAATCTTCTTA
GGTGTGTGGAGGCAGGTGTTTTATATCTTAACTCGAAAGTGGATAGGATCGTTGAGTC
CACAAGTGGCCACAGTCTTGTAGAGTGCGAGGGCGACATTGTCATTCCTTGCAGGTAT
AGTTGGACCGCTACATCAAATTGTCACTGTTCACGTTAGAAACTTAACTGCATCCTTG
TTCTGATGCTTCTAACTTATGATAACAGGTTTGTCACTGTTGCATCTGGAGCCGCCTCA
GGGAAATTCTTGCAGTATGAGTTGGGAGGTCCTCGGGTTTCTGTTCAAACAGCTTATG
GAGTGGAAGTTGAGGTAGAGAAATATCATCAAACCTCGAGTAACTCTTGAGGGTTTT
TCATGTTATGCACCAGAAGACTTTAGACTGTTACCTGTAGTTATCATTTACGAGCTTAT
ATGAACAATTTGTTGCATTATTCCTTTTATGGCCTATCCTTTCACTGCTCGTCTTGGAGA
GTACGCATTCAATTTGGCGATTCTGTTTCTATTGTAAACTTAGAATTGGACTTCCTATA
AGGTTATGCACATTCAAGAAAACCGCTATCTGCATTTCCCAATTACTAGCTTGGCTGA
TTTTGCAATTATATGATCATTCTTCTTTTCTTTTTTCCTATATATTAGGTCGATAACAAT
CCGTATGATCCAAGCCTGATGGTTTTCATGGATTATAGAGACTATGTCAGACACGACG
CTCAATCTTTAGAAGCTAAATATCCAACATTTCTTTATGCCATGCCCATGACTAAAAC
AAGAGTCTTTTTCGAGGTTAGTTTTTGATATTATGCCTCATACTTTACTACTTGGCTTTG
TGTGACAGACAAAAGTATCTATCTGCAGGAAACTTGTTTGGCTTCAAAAGATGCAAT
GCCATTTGATCTATTAAAGAAAAAACTGATGTTACGATTGAACACATTGGGCATAAA
AATTAAAAAAATCTACGAGGAGGTAATCCACTCTACCTTTAGTTACGTGCAATTTTTG
CTAGAGAAAAAAAAGGGCAACCCGGTGCAAAGCACCCGCTATGCGCGGGTCCGGGG
AAGGGCTGGACCACAAGGGTCTATTGTACGCAACCTTACCCTGCATTTCTGCAAGAG
ACTGTTTCCACGGCTCGAATTCGTGACCTCTTGGTCGCATGGCAGCAACTTTACCAGT
TACGCCAAGGCTCCCCGTGAACACACTAGAGAATGACTGAAAAATGACTTTAACAA

AATTTTACGCAGGAATGGTCTTACATACCAGTTGGTGGATCGTTGCCAAATACAGAGC
AGAAAACACTTGCGTTTGGCGCTGCTGCTAGCATGGTTCATCCAGCTACAGGTAATA
AGAGAAGAAAATACATTACTATTACTGTGTTCTTTAAATGCAATGCTTCTGAAACTTC
CTCTACTTCATAAACAGGTTATTCAGTTGTCAGATCACTGTCCGAGGCGCCAAAATGC
GCCTCCGTACTTGCAAATATTTTAAGACAAAATCATGTCAAGAACATGATTACCAGTT
CAAGTGCCACAAGTATCTCAACTCAAGGTACTTGTTATCCAATTATACTAGTTTTTGA
CTGCATTGGTAGTGTCCTGACCTATATGGACTCAGCAAGTTGCTCATATTTTCTTCAGC
TTGGAACACCCTTTGGCCACAAGAACGAAAAAGGCAACGATCGTTTTTCCTATTTGG
ATTGGCACTCATATTGCAGCTGGATATTGAGGGGATTAGGTCATTTTTCCGCGCATTCT
TCCGTGTACCAAAATGGTATGGTTTGATCTTGCTCCATCACCCCGTTATCTTCGATTAT
GTGGCATCGTTTTCCAAATCAGTTTAACTTCTATACGCTGACAATATAAAGAATTTTTA
AACTACAAGATGACCAAAAAAAAACTACGTGTAACGGTTTGTAATAAGTGAGTCAGT
AACGCACAAAATAAGGCAGGGCAACATGATACAACAAGTTAAGATACACTAATACC
AACAACAACAACAACTATACCCTCTGTACCAAACAAGTTGGAGCGGCTATCAGAGG
CGGAGCTAGAGTGCCCCGAACGGGTTCGGCCGAACCCAGTAGCTTTGGTTCAAACCC
TGTATTTGTCTCTAAAAAATTCATTGAATATATATAAATTATTAAATTAGAATCCAATA
GCTTAAAACGATTAGAATTTCGAACCCATAAACTTGAAATCCTGACTACCTCTGGCG
ACTGTATGAATCCTCAATGACTATCCATGTAAATTCTTCTCATGTCAACATTATACGA
AACAAAAATCAAAACGAAAAGTACGTACAAGTTTTCTATGTTTCCAACTGACATAAG
AATCCCAAGAGTCTAAATTCTTTAGAGTGTCAGTGCATATAATGAGAAGTGTACTTAT
TGCAGGATGTGGCAAGGTTTTCTTGGCTCTAGTCTTTCATCAGCAGACCTCATGTTATT
TGCCTTCTACATGTTTATTATTGCACCAAATGACATGAGAAAAGGCCTAATCAGACAT
CTGTTATCTGATCCAACTGGTGCAACTATGATAAGAACTTATCTTACATTTTAG

>mRNA_60735_cds

ATGGATTGTATTGGAGCTCGAAATTTTTCTACAATGGCGGTTTTTACGTGTCCGAGATT
CAAATCATTAGGAAGAAAGAGAATTATGCCAAGAAAAAAGCAACCATTTTGGCCTA
TACATATGAAAGTGAAGTGTAGTGGAAGTGATAGTTGTGTAGTGGTTAAAGAAGATT
TTGCTGATGAAGAAGATTATATAAAAGCTGGTGGTTCAGAACTTGTTTTTGTTCAAAT
GCAGCAGAATAAAGACATGGATCTACAGTCTAAGCTTTCTGATAAGTTGCGACAAAT
ATCATCAGCTGGACAAACTATACTGGATTTAGTGGTCATTGGCTGTGGTCCTGCTGGT
CTTGCTCTTGCTGCGGAGTCTGCTAAACTCGGGTTGAACGTGGGGCTCGTTGGTCCTG
ATCTTCCTTTCACAAATAACTATGGTGTCTGGGAAGACGAGTTCAAAGATCTTGGGCT
TCAAGCATGCATTGAACATGTTTGGAGTGATACCATTGTATATCTTGATGATGCCGAT
CCAATTCTTATTGGACGTGCTTATGGAAGAGTTAGTCGCCATTTACTGCATGAGGAGT
TACTCAAAAGGTGTGTGGAGGCAGGTGTTTTATATCTTAACTCGAAAGTGGATAGGAT
CGTTGAGTCCACAAGTGGCCACAGTCTTGTAGAGTGCGAGGGCGACATTGTCATTCCT
TGCAGGTTTGTCACTGTTGCATCTGGAGCCGCCTCAGGGAAATTCTTGCAGTATGAGT
TGGGAGGTCCTCGGGTTTCTGTTCAAACAGCTTATGGAGTGGAAGTTGAGGTCGATAA
CAATCCGTATGATCCAAGCCTGATGGTTTTCATGGATTATAGAGACTATGTCAGACAC
GACGCTCAATCTTTAGAAGCTAAATATCCAACATTTCTTTATGCCATGCCCATGACTA
AAACAGGAGTCTTTTTCGAGGAAACTTGTTTGGCTTCAAAAGATGCAATGCCATTTGA
TCTATTAAAGAAAAAACTGATGTTACGATTGAACACATTGGGCATAAAAATTAAAAA
AATCTACGAGGAGGAATGGTCTTACATACCAGTTGGTGGATCGTTGCCAAATACAGA

GCAGAAAACACTTGCGTTTGGCGCTGCTGCTAGCATGGTTCATCCAGCTACAGGTTAT
TCAGTTGTCAGATCACTGTCCGAGGCGCCAAAATGCGCCTCCGTACTTGCAAATATTT
TAAGACAAAATCATGTCAAGAACATGATTACCAGTTCAAGTGCCACAAGTATCTCAA
CTCAAGCTTGGAACACCCTTTGGCCACAAGAACGAAAAAGGCAACGATCGTTTTTCC
TATTTGGATTGGCACTCATATTGCAGCTGGATATTGAGGGGATTAGGTCATTTTTCCGC
GCATTCTTCCGTGTACCAAAATGGATGTGGCAAGGTTTTCTTGGCTCTAGTCTTTCATC
AGCAGACCTCATGTTATTTGCCTTCTACATGTTTATTATTGCACCAAATGACATGAGA
AAAGGCCTAATCAGACATCTGTTATCTGATCCAACTGGTGCAACTATGATAAGAACTT
ATCTTACATTTTAG

>ε-LCY2_Ntab0455950

ATGGATTGTATTGGAGCTCGAAATTTTGCTACAATGGCGGTTTTTACGTGTCCGAGATT
CAAATCATTAGGAAGAAGGAGAATTATGCCAAGAAAAAAGCAACCAATTTGGCCTA
TACATATGCAAGTGAAGTGTAGTGGAAATGAGAGTTGTGTAGTAGTTAAAGAAGATT
TTGCCGATGAAGAGGATTATATAAAAGCTGGTGGTTCAGAACTTGTTTTTGTTCAAAT
GCAGCAGAATAAAGACATGGATCTGCAGTCTAAGCTTTCTGATAAGGTGATTTTCTTG
ATCCCATTTTTAATAAGTATAAAAGATATTTTACATTATCAGTGTAGTTTAATACAGTG
ACGGAAGCAGTATTTTCACCGAAAAGTTTCAAGAAATAGAAAAAGAATATATATAC
ACACTGATAATGTAATTCTTCGCGAAATGGGTTCGGCTGAACCCCTCCCGCCCACCTA
GCTCCGCCCCTAGTTTAAGATGTGATAGTAGGTAAACTCTTTACTAGGTTATAAATTA
ATGTTTATCATACATATTTGCATGTAATTAATTTATAAGTAACCTGATTGTGTAAGTAT
GCATTGACAAGGTAACAAATTTTACATTTTTAGTGGATTTTAAAATTGTTGTAGCACGT
TACGTGTCTTATTTTCCAGGTCGCTAATTCCACTTATTATGGATGGTTACTATAGTTATC
TCTTAGGCACGAGTTGATAGTATAGAGATTCTTTTAGCCTATCAGTGTTTATATAAGTT
AAACTCTTTAAAAAGGGATTAACATATATACTGACTGTGTAAAAAATGTTTAGATCCT
CTTGTTATTATTGTTTGTTGCTAATGCTTCCTTTCACCTTTCTTGAACCAATGATCTATC
GAAAGTAGCCTCTCTACCTTCACCAGGTAGGGGTAAGGTCTGCGTACACACTTCCCTC
CCTAGACCCTATTTGTGGGATTACACCGGGTTTGTTGTTGTAGTAAAAAATGTTTAGA
CTATTGTGTAGTTTAATAAGCTTATAGTGTAGGAATCTTTTTGTAGTTTTAGTCGGCGTT
TGGACATAAGAATTGTAAAATTCCAAAATAGGGGGAAAATTTTTTCAAGCGAAAATG
GTATTTGAAATTTAGAGTTGTATTTGGACATGAATATAATTTTGGGTTGTTTTTGAAGT
TTTGTGAATGATTTGTGTGAAAATTTTGAAAAATAACTTTTTGGAGTTTTTCAAATTTTT
GAAAATTTTCAAAATGCATCTTCGAGTGAAAATTGGAAATTTTATGAACAAACGCTC
ATTTCAAAAAAAAAGTGAAATGTTTTTGGAAAAAAGGAAAAATTTTCTTATGTCCAA
ACGGGCTCTTAATACTATTTAACCGGTTGTAATATGTTACTTACCTATATTACCATCTC
CCACATAGTATTGAAAGCAATATGTAGTTAGCTTGGGAAAATATTTACAATGTTAGTG
TAAATTCTGTAATATGGTAGCAACTACATTGCTTTGGTCTCTAATACTGTAGCAACTAT
TAGTTCTCATATCTAGCAACTATATTGCTTTGTTCATCATACTATCTCAGCTTGCAGTT
GCGACAAATATCATCAGCTGGACAAACTATACTGGATTTGGTGGTCATAGGCTGTGG
TCCTGCTGGTCTTGCTCTTGCTGCGGAGTCTGCTAAACTCGGATTGAACGTTGGGCTCG
TTGGTCCTGATCTTCCTTTCACAAATAACTATGGTGTTTGGGAGGATGAGTTCAAAGG
TCGTAATCTTTATTTGGCTTTTATCAAGAACTAGTTTGTCTCCCTATCCTTTCAAGAAA
CAGGACACTTTGTTTTACTTTCTTGAAACAATTAGCTAAAAAGCACATTGTATTTCCTT
TTATCAAATGCTAGATTCATAGGACTTGTTACATTCACCCTTTTAGCAATTTGTCAACT

TGAGATCCAAGAGATAGTTAATTTCACAGACATAAACTGATACTTGAAGTAATCACT
TTACTTTAGTTATATGCATGAGCACCGGCAACAACAACAACATCGTGTAATCTCATAA
GTGGGTTCTGGGGAAGGTAGTGAGTACGTAACCTTACTCCTACATTGTGCAGGTAAAT
AGACTGTTTCCGATAGATATATGCATGAGCACTGGATCTGTCATTATTGGTTTTGAGGT
TTTTGATACTCAACTTGGTTTTTCACTGCAAAATTGATGTTTAGGATATGATATTCCAG
GCTAAAATTACTCTCCAAACTTCATATGCTTAATACTTTCTTTTTTCAGATCTTGGGCTT
CAAGCGTGCATTGAACATGTTTGGAGGGATACCATAGTATATCTTGACGATGCCGATC
CAATTCTTATCGGTCGTGCTTATGGAAGAGTTAGTCGCCATTTACTGCACGAGGAGTT
ACTCAAAAGGTTAAATTCACTGAACTTGTTCCTTATGTAATACAAGTTGTTAATTTATT
CTTTTCAATTGTTTCATCATTTTATTTTCCTTAAAAGTTGCAATTCACATTAATCTTCTT
AGGTGTGTGGAGGCAGGTGTTTTATATCTTAACTCGAAAGTGGATAGGATCGTTGAGT
CCACAAGTGGCCACAGTCTTGTAGAGTGCGAGGGCGACATTGTCATTCCTTGCAGGT
ATAGTTGGACCGCTACTTCAAATTGTCGCTGTTCACGTTAGAAACTTAACTGCATCCTT
GTTCTGACGCTTCTAACTTATGATAACAGGTTTGTCACTGTTGCATCTGGTGCTGCCTC
AGGGAAATTCTTGCAGTATGAGTTGGGAGGTCCTCGGGTTTCTGTTCAAACAGCTTAT
GGAGTGGAAGTTGAGGTAGAGAAAGATCATCAAACATAAGTAACTCTGAAGTTTTTT
CATGTTATACACTCGAAGAACATTAAACTGTTATCTCTAGCTATCGTTTACGAGCTTAT
ATGAACAAATTGTTGCATTATTCCTTTTTTTGGCTTATCCTTACACTGCTCGTCTCGGAG
AATATGTATTCAATTTGACGATTCTGTTTCTATTGTAAACGTAGAATGGGACTTCCTAT
TCATCAGATAAAAGGAAACTACTATAATGTTATGCACATTCAAGAAACTGCTATCTG
CATTTCCCAATTATCAGTTATGGCACCTAACTTGGCTAATTCTGCAATTATATGATCAT
TCTTCTTCTTTTTTCCCTATATATTAGGTCGATAACAATCCGTATGATCCAAGCCTGAT
GGTTTTCATGGATTATAGAGACTATGTCAGACACGNNNNNNNNNNNCGACGCTCAA
TCTTTAGAAGCTAAATATCCAACATTTCTTTATGCCATGCCCATGACTAAAACAAGAG
TCTTTTTCGAGGTTAGTTTTTGATATTATGCCTCACACTTTACGACTTGGCTATATGTGA
CAGATAAAAGTATCTATCTGTAGGAAACTTGTTTGGCTTCAAAAGATGCAATGCCATT
TGATTTGTTAAAGAAAAAACTGATGTTACGATTGAACACACTGGGTGTAAGAATTAA
GCAAATCTACGAGGAGGTAATCCACTCTGCCTTTAGTTACGTGCAATTTTTGCTAGAG
AAAAAAGGGATGCCCAGTGTATTAAGCACCCGCTATGCGCGGGGTGTGGGGAAGGG
CCAGATCACAAGGGTCTATTGCACGCAACCTTACCCTGCATTTCTGCAAGAGACTATT
TCCACGGCTCGAACCCCGTGACTTCCTGGTCGCATGGCAACAACTTTACCAGTTATGC
CAAGACTCCCCGTGAACACACTAGAGAATGACTGAAAAATGACGATAACAAAATTT
TACGCAGGAATGGTCATACATACCAGTTGGTGGATCTTTACCAAATACCGAGCAAAA
AACACTTGCATTTGGTGCTGCTGCTAGCATGGTTCATCCAGCTACAGGTAATAAGAGA
AGAAAATACATTACTATTACTGTGTTCTTTAAATGCAATGCTTCTGAAACTTCCTCTAC
TTCATAAACAGGTTATTCAGCATTAACAGGTTATTCAGTTGTCAGATCACTGTCCGAG
GCACCAAAATGCGCCTCCGTACTTGCTAATATTTTACGACAAAATCATGTCAAGAAC
ATGCTAACCAGTTCAAGTACCACAAGTATCTCAACTCAAGGTACTTGTTATCCAATTA
TACTAGTTTTTGACTGCATTTGTAGTGTCCTGACCTTTATAGACTCAGCAAGTTGCTCA
TATTTTCTTCAGCTTGGAACACCCTTTGGCCACAAGAACGAAAAAGGCAACGATCGTT
TTTCCTATTTGGATTGGCACTCATATTGCAGTTGGATATTGAGGGGATTAGGTCATTTT
TCCGCGCATTCTTCCGTGTGCCAAAATGGTATGGTTTGATCTTGCTCCATCACCCCGTT
ATCTTCGATGATGTAGCATCGTATTCCAAATCAATTTAACTTCTATACACTTACAATAT
AAATAATATTTATACTACAAGATGATCAAAAAACAAATAAGTACATGTAACTGTTTA

TAATAAGTGAAATTAATAATGCACAAAATAAGGCAGTTCAACATGATACAACAGGTT
AAGAACATTAATACCAACAACAACAACAACAACTATGCCCTCAGTACCAAACAAGT
TGGAGCGGCTATCAGGGGCGGAGCTAGAGTGCCCCGAGCAGGTTCTGCCGAACCCA
ATAGTTTTGGTTCGAACCCTCTATTTGTCTTAAAAAATTCATTGAATATATATAAATTA
TTAATTTACAACCTAATACCTTAAAACGAGTAGAATTCCGAACCCATAAACTTGAAA
TCTTGGCTCCGCCTCTGGCAGCTATATGAATCCTCAATGACCATATTCCCTATTTAAAT
TCTTCTCAAGTCAACATTATACGAAACAAAAATCAAAACGAAAAGTACGTACAAGTT
TTCTATGTTTCCAACTGACATAAGAATCCCAAAAGCGTAAATTTCTTAAACTGTCAGT
GTATATAAGTTAAGTCCTTCCCGAATACAAAAAGAAACTTCTAGTTTCAACAGTACTA
ATGAGGAGTGTACTTATTGCAGGATGTGGCAAGGATTTCTTGGCTCTAGTCTTTCATC
AGCAGACCTCATGTTATTTGCCTTCTACATGTTTATTATTGCACCAAATGACATGAGA
AAAGGCCTAATCAGACATTTGTTATCTGATCCAACTGGTGCAACCATGATAAGAACTT
ATCTTACATTTTAG

>mRNA_99724_cds

ATGGATTGTATTGGAGCTCGAAATTTTGCTACAATGGCGGTTTTTACGTGTCCGAGATT
CAAATCATTAGGAAGAAGGAGAATTATGCCAAGAAAAAAGCAACCAATTTGGCCTA
TACATATGCAAGTGAAGTGTAGTGGAAATGAGAGTTGTGTAGTAGTTAAAGAAGATT
TTGCCGATGAAGAGGATTATATAAAAGCTGGTGGTTCAGAACTTGTTTTTGTTCAAAT
GCAGCAGAATAAAGACATGGATCTGCAGTCTAAGCTTTCTGATAAGTTGCGACAAAT
ATCATCAGCTGGACAAACTATACTGGATTTGGTGGTCATAGGCTGTGGTCCTGCTGGT
CTTGCTCTTGCTGCGGAGTCTGCTAAACTCGGATTGAACGTTGGGCTCGTTGGTCCTGA
TCTTCCTTTCACAAATAACTATGGTGTTTGGGAGGATGAGTTCAAAGATCTTGGGCTT
CAAGCGTGCATTGAACATGTTTGGAGGGATACCATAGTATATCTTGACGATGCCGATC
CAATTCTTATCGGTCGTGCTTATGGAAGAGTTAGTCGCCATTTACTGCACGAGGAGTT
ACTCAAAAGGTGTGTGGAGGCAGGTGTTTTATATCTTAACTCGAAAGTGGATAGGAT
CGTTGAGTCCACAAGTGGCCACAGTCTTGTAGAGTGCGAGGGCGACATTGTCATTCCT
TGCAGGTTTGTCACTGTTGCATCTGGTGCTGCCTCAGGGAAATTCTTGCAGTATGAGTT
GGGAGGTCCTCGGGTTTCTGTTCAAACAGCTTATGGAGTGGAAGTTGAGGTCGATAA
CAATCCGTATGATCCAAGCCTGATGGTTTTCATGGATTATAGAGACTATGTCAGACAC
GACGCTCAATCTTTAGAAGCTAAATATCCAACATTTCTTTATGCCATGCCCATGACTA
AAACAAGAGTCTTTTTCGAGGAAACTTGTTTGGCTTCAAAAGATGCAATGCCATTTGA
TTTGTTAAAGAAAAAACTGATGTTACGATTGAACACACTGGGTGTAAGAATTAAGCA
AATCTACGAGGAGGAATGGTCATACATACCAGTTGGTGGATCTTTACCAAATACCGA
GCAAAAAACACTTGCATTTGGTGCTGCTGCTAGCATGGTTCATCCAGCTACAGGTTAT
TCAGTTGTCAGATCACTGTCCGAGGCACCAAAATGCGCCTCCGTACTTGCTAATATTT
TACGACAAAATCATGTCAAGAACATGCTAACCAGTTCAAGTACCACAAGTATCTCAA
CTCAAGCTTGGAACACCCTTTGGCCACAAGAACGAAAAAGGCAACGATCGTTTTTCC
TATTTGGATTGGCACTCATATTGCAGTTGGATATTGAGGGGATTAGGTCATTTTTCCGC
GCATTCTTCCGTGTGCCAAAATGGATGTGGCAAGGATTTCTTGGCTCTAGTCTTTCATC
AGCAGACCTCATGTTATTTGCCTTCTACATGTTTATTATTGCACCAAATGACATGAGA
AAAGGCCTAATCAGACATTTGTTATCTGATCCAACTGGTGCAACCATGATAAGAACTT
ATCTTACATTTTAG

>β-OHase1_Ntab0677920

ATGGCCGCCACCAGAATTTCCTTTACTTCCAGTTCACAAACCATTTATTTCCGTCACAG
CCCATTTCTTGGCCCAAATCCGGTTTTCCCAATCTCTTCCTTTTCTCGTAATCTTGGCAC
AATTCTCAGGTCTAGAAGAAAACCCAGTTTCACAGTTTGCTTTGTTCTGGGGGATGAA
AAGTTGAATACTCAATTTGACAAGTTGAATGCTCAATTAGAGACTGGGACAGAGGAG
ATTGAATTGAAAATTGAGGAGCAGATCTCAGCGACGCGTTTAGCGGAAAAATTGGCT
AGGAAAAAATCGGAGAGGTTTACTTATCTTTTGGCTGCGGTTATGTCTAGTTTTGGGA
TTACTTCCTTGGCTATTATGGCCGTCTATTACAGATTTTCGTGGCAAATGGAGGTATGA
AGCACTCCATAATTAATTCCCACTTCACTCTTCTTCTCCTTTTTCTGATTTTTAATTTTTA
TTTTATTTTTTTATATTCTGTTTGGGTGTAGGGTGGAGAAATGCCTTTAACTGAAATGTT
TGGTACATTTGCTCTCTCAGTTGGTGCTGCTGTAAGTTTCTTTTTTTGAACTATGTTTTA
GCGTCTGGTAATACATTATCTCCTGTCCATTTCATATAATTGAACTACATTAAAACTTA
GACACTAGTATAGTGATTATGAAACAAAACGTACATATAGGAGTATGAATCATTTCA
ACTAGACTACTTTTAAAGAGTTGTTGCAAGTCATATCGATAAGGAAAAATGTCTAATG
TGTTTTATATAAAATTGAAAGAGGAGTAGCAATTAAGGACTAGGAGAGTCATTTATG
GTTGACGAAGTTGGATGTTGTTCGAATTTCAGGTAGGAATGGAGTTTTGGGCAAGGTG
GGCTCATAGAGCGCTTTGGCACGATTCTCTTTGGCACATGCACGAGGTTTGTGTTTTAC
TTTCTAATTTGCTTATTTGGAGCTTAAATGCTTATTAAACTATGCTAATTTTATAAAAA
AAAATCTCAACTTAATTAAATCCTTTCTCTTACCTTAAATTGTTGCAGTCACACCACAA
ACCAAGAGAAGGACCTTTCGAGATGAACGATGTGTTTGCCATAGTCAACGCCGTTCC
AGCAATAGCCCTCCTCAACTATGGTTTCTTCCATAAAGGCCTCATTCCTGGCCTCTGCT
TCGGCGCTGTATGTTTTATTTCACTCACTCTTCAGTATTTAGTGTGGTTTTAATCCTTAC
CAACAGCGGTCTGGAATATTTTATGCGGATCAGATTACTATCCATGTATAGTATAAAG
ACTTACTAGTGTAAATACATTGATCAGAAGAGAGGTATAGAGAAAGAAGCCCAACA
CATTAAGTTGATAAAAAAAAAATGCCTTGTAATGCAGGGGCTAGGGATCACAGTATT
TGGGATGGCTTACATGTTCGTTCACGATGGTTTGGTTCACAAGAGATTCCCCGTCGGA
CCTGTAGCCAATGTACCTTATTTAAGGAAGGTGGCCGCAGCTCATTCGGTAATTAAAG
ATGCTTTAAACTATAACTATGGCTTATAAAAGATGATCTTAATTAATCTAATCAGAGT
TAGCATGATTGTTAAGTAGATATTAATAGTATTAAATTGGTTGGTAAGATTTCCTTAAT
TCATTTCTTGATATTTGCAGCTCCATCACTCAGAGAAGTTCAATGGTGTCCCATATGGT
TTGTTCTTAGGACCAAAGGTGCTCTCTCTACTCTTTTCCACTCCCCACACTTCCCTTCAT
GTTCATAACCTTTTTAAAAATTACACATGACACTAGGAAATACTAACTAGTGTAATGG
TGATTGGTGCAGGAATTGGAAGAAGTAGGGGGGATTGAAGAATTGGAAAAGGAAGT
GAACCGAAGGATAAAACTTTCAAGGGGATCATGA

>mRNA_106915_cds

ATGGCCGCCACCAGAATTTCCTTTACTTCCAGTTCACAAACCATTTATTTCCGTCACAG
CCCATTTCTTGGCCCAAATCCGGTTTTCCCAATCTCTTCCTTTTCTCGTAATCTTGGCAC
AATTCTCAGGTCTAGAAGAAAACCCAGTTTCACAGTTTGCTTTGTTCTGGGGGATGAA
AAGTTGAATACTCAATTTGACAAGTTGAATGCTCAATTAGAGACTGGGACAGAGGAG
ATTGAATTGAAAATTGAGGAGCAGATCTCAGCGACGCGTTTAGCGGAAAAATTGGCT
AGGAAAAAATCGGAGAGGTTTACTTATCTTTTGGCTGCGGTTATGTCTAGTTTTGGGA
TTACTTCCTTGGCTATTATGGCCGTCTATTACAGATTTTCGTGGCAAATGGAGGGTGGA
GAAATGCCTTTAACTGAAATGTTTGGTACATTTGCTCTCTCAGTTGGTGCTGCTGTAGG
AATGGAGTTTTGGGCAAGGTGGGCTCATAGAGCGCTTTGGCACGATTCTCTTTGGCAC

ATGCACGAGTCACACCACAAACCAAGAGAAGGACCTTTCGAGATGAACGATGTGTTT
GCCATAGTCAACGCCGTTCCAGCAATAGCCCTCCTCAACTATGGTTTCTTCCATAAAG
GCCTCATTCCTGGCCTCTGCTTCGGCGCTGGGCTAGGGATCACAGTATTTGGGATGGC
TTACATGTTCGTTCACGATGGTTTGGTTCACAAGAGATTCCCCGTCGGACCTGTAGCC
AATGTACCTTATTTAAGGAAGGTGGCCGCAGCTCATTCGCTCCATCACTCAGAGAAGT
TCAATGGTGTCCCATATGGTTTGTTCTTAGGACCAAAGGAATTGGAAGAAGTAGGGG
GGATTGAAGAATTGGAAAAGGAAGTGAACCGAAGGATAAAACTTTCAAGGGGATCA
TGA

>β-OHase2_Ntab0861090

ATGGCTGCCGGATCTGCTAGCTCCCAAACCATTTGTTTCAACCACAACCCAATTCTTG
CCCCAAAACCAACCACAACTAGCCCACCAGTTTTGTTCTTCTCTCCGTTAACTCGCAA
CTTTGGCAAAACAAATCTGCAGTGTCGAAGAAAGCCAAAGATGACGGCTTGCTTTGT
GCTGGAGAATGAGAAATTGAATACGCATAGTGACATTATTGAAGCAAAGCAGATGG
GAGTAAAAAATGAGGAGCAGCGTTCAGATGCGAGGCTGGCGGAAAAACTGGCTAGG
AAAAGATCGGAGAGGTTTACTTATCTTGTCGCAGCTGTGATGTCTAGTTTAGGGATTA
CTTCTATGGCTGTTTTGGCGGTTTATTACAGATTTTCTTGGCAAATGGAGGTACCAATT
AATCACTCTCTTCTTCTTCTCCAGCTTAATCCTTTAGCTCTTTGTTGGTTACATTAACCT
TGTTCTCAAAACTTTATATATATATATGATATAGGGTGGAGAAGTGTCTTTTTCTGAAA
TGTTCGGTACTTTCGCTCTCTCGTTTGGCGCTGCTGTAAGTTACACTTTTTCATATCCAA
TATACGTATCTTATAATCAGCAGTTAAACAAACAGAAAGATTTCTGATTATTATAAGC
TGATTGCTGAATTGAATGTTGTGGTGATTTCATCAGGTAGGAATGGAGTTTTGGGCGA
TATGGGCACATAAAGCGCTGTGGCATGATTCATTATGGCACATGCACGAGGTTTGCA
AATTTCCACTAAATTATTAGATACTAATTAATTTGATTATTGTACTTGATTTGTTTGTTA
GGAAAATCAAGATTCTTGTCATGTTTTTCCCCGGCAACCAAATATTGACTACGTGATG
TTTTAAATAATATTTAATTTGCAGTCACACCATAAACCAAGAGAAGGAGCATTCGAG
CTGAACGATGTTTTTGCCATAACAAACGCTGTTCCAGCCATTGCTCTTCTTGCCTATGG
CTTTTTCAATAAAGGACTCATTCCTGGACTCTGTTTCGGCGCGGTATGTTCTCACTAAT
TAATTCTTGATTAAGAGAGACTAATTAGGTATAAAGTATTTAAAAGAAATTAGTGAA
ATGTAAAAAAAAAAAAATGTGCTCTTATAAATGCAGGGACTAGGGATCACAGTATTC
GGGATGGCTTACATGTTCGTTCACGATGGATTAGTTCACAAGAGATTCCCAGTGGGAC
CCATTGCCAATGTACCTTATTTTCGGAGAATAGCTGCAGCTCATCAGGTAAAAGTTCT
TTAACTCTTAATCTTAAATTAATCACGTTTAGCACCGTAGTTTCATTAGATTAATATTA
AATTTACTGGTCAGTTGTTTTTTTAGAGTATATCTTAAATTTACTGATATTTTCTTTGAT
TTCAGCTTCATCACTCGGAGAAATTTGATGGTGTCCCATATGGCTTGTTCCTAGGACCT
AAGGTGCCTACTCTCTCTTCCTCTTTTAACCTCCTAGTGTTAATAAATATTTCTTATTCT
TTTTTATTCGGTTAGACATTTATAACATAATTCGGAATTAAATGGTTAAAAGAGGCTT
ACGGTAGATTGATTGGTTATTCGTGCAGGAATTGGAAGAAGTAGGAGGAAATGAAGA
GTTGGAAAAGGAGGTCAACCGAAGGATTAAAAATTCCGAGGGATTATGA

>mRNA_120276_cds

ATGGCTGCCGGATCTGCTAGCTCCCAAACCATTTGTTTCAACCACAACCCAATTCTTG
CCCCAAAACCAACCACAACTAGCCCACCAGTTTTGTTCTTCTCTCCGTTAACTCGCAA
CTTTGGCAAAACAAATCTGCAGTGTCGAAGAAAGCCAAAGATGACGGCTTGCTTTGT

GCTGGAGAATGAGAAATTGAATACGCATAGTGACATTATTGAAGCAAAGCAGATGG
GAGTAAAAAATGAGGAGCAGCGTTCAGATGCGAGGCTGGCGGAAAAACTGGCTAGG
AAAAGATCGGAGAGGTTTACTTATCTTGTCGCAGCTGTGATGTCTAGTTTAGGGATTA
CTTCTATGGCTGTTTTGGCGGTTTATTACAGATTTTCTTGGCAAATGGAGGGTGGAGAA
GTGTCTTTTTCTGAAATGTTCGGTACTTTCGCTCTCTCGTTTGGCGCTGCTGTAGGAATG
GAGTTTTGGGCGATATGGGCACATAAAGCGCTGTGGCATGATTCATTATGGCACATGC
ACGAGTCACACCATAAACCAAGAGAAGGAGCATTCGAGCTGAACGATGTTTTTGCCA
TAACAAACGCTGTTCCAGCCATTGCTCTTCTTGCCTATGGCTTTTTCAATAAAGGACTC
ATTCCTGGACTCTGTTTCGGCGCGGGACTAGGGATCACAGTATTCGGGATGGCTTACA
TGTTCGTTCACGATGGATTAGTTCACAAGAGATTCCCAGTGGGACCCATTGCCAATGT
ACCTTATTTTCGGAGAATAGCTGCAGCTCATCAGCTTCATCACTCGGAGAAATTTGAT
GGTGTCCCATATGGCTTGTTCCTAGGACCTAAGGAATTGGAAGAAGTAGGAGGAAAT
GAAGAGTTGGAAAAGGAGGTCAACCGAAGGATTAAAAATTCCGAGGGATTATGA

>β-OHase3_Ntab0486180

ATGGCCGCCAGCAGAATTTCCTTTACTTCCAGCTCTCAAACCATTTATTTCCGTCACGG
CCCGTTTCTATGCCCAAATCCGGTTTTCCCAATCTCTTCCTTTTCTCGTAATCTTGGCAC
AATTCTGAGGTCTAGAAGAAAACCCAGTTTCACAGTTTGCTTTGTTCTGGAGGATGAG
AAGTTGAATACTCTATTTGACAAGTTGAATGCTCAATTAGAAACTGGGACAGAGGAG
ATTGAAATGAAAATTGAGGAGCAGATCTCAGCGACTCGTTTAGCGGAAAAATTGGCT
AGGAAAAAATCGGAGAGGTTTACTTATCTTGTGGCTGCGGTTATGTCTAGTTTTGGGA
TTACTTCCTTGGCTATTATGGCCGTCTATTACAGATTTTCGTGGCAAATGGAGGTATGA
AACACTCCATAATTAATTCCCACTTCACTCTTCTTCTCCTTTTTCTGATTTTTAAATTTTT
TAATATTCTGTTTGGGTGTAGGGTGGAGAAGTGCCTTTAACTGAAATGTTTGGTACATT
TGCTCTCTCAGTTGGTGCTGCTGTAAGTTTCTTCTTTTCTACTATCTTTTAGCGTCTGGT
AATACATGATCAGTTGAAAACAACCAGAGATATAAGTATCTCCTGTCCATTTCATATG
ATAGTGTATATAATTGAACTACATTAAAACTTAGACGCTAGTATAGTGATTATGAAAC
AAAACGTTAAGCTGCTACATCAAAGTTTTCAACTAGACTACTTTTAAAGAGTTGTTGC
AAGTCATATCGATAAAGAAAAATGTCTAATGTGTTTTATATAAAATTGAAAGAGGAG
TACCAATTAAGGACTAGGATAGTCATTTATGGTTGACGAAGTTGGATGTTGTTCGAAT
TTCAGGTAGGAATGGAGTTTTGGGCAAGGTGGGCTCATAGAGCGCTTTGGCACGATT
CTCTTTGGCACATGCACGAGGTTTGTGTTCTACTTTCTAATTTACTTATTTGGAGCTTAA
ATGCCTTATTTCATTTTTTTGTGAAGAAAACTATACTAATTTTATAAAAATCTCAACTT
AATTAAATCCTTTCTCTTACCTTAAATTGTTGCAGTCACACCATAAACCAAGAGAAGG
ACCTTTCGAGATGAACGATGTGTTTGCCATAATCAACGCCGTTCCAGCAATAGCCCTC
CTCAACTATGGTTTCTTCCATAAAGGCCTCATTCCTGGCCTCTGCTTCGGCGCTGTATG
TTTCATTTCACTTTACCTTTCACTCTTCAGTATTTAGTGGGGTTTTAATCTTTACCAACA
GCGGTCTGGAAAATTTTACTCAGATCAATTACTATCCATGTATAGTATAAAGACTTAC
TAATGTAAATACATTGATCAGAAGAGAGGTATAGAGAAAGAAGCCCAACATATTAA
ATTGATAAAAAAATGCCTTGTAATGCAGGGGCTAGGGATCACAGTATTTGGGATGGC
TTACATGTTCGTTCACGATGGTTTGGTTCACAAGAGATTCCCCGTCGGACCTGTAGCC
AATGTACCTTATTTTAGGAAGGTGGCCGCAGCTCATTTGGTAATTAAAGATGCTTTAA
ACTATAACTATGGCTTATAAAGGATAATCTTAATCTAATCAGACTTAGCATGATTGTT
AAGTAGATATTAATATTAAATTGGTTGGTAAGATTTCCTTAATTCATTTGTTGATATTT

GCAGCTCCATCACTCAGAGAAGTTCAATGGTGTCCCATATGGTTTGTTCTTGGGACCA
AAGGTGCTCTCTCTACTCTTTTCCACTCCCCACACTTCCCTTCATGTTCATAAGCTTTTT
AAAATTACACATGACATTGTTTTTCAGTAAACAAGGGAGTAGTATATAATAATAGG
AAATACTAACTAGTGGAATGGTGATTGATGCAGGAATTGGAAGAAGTAGGGGGGATT
GAAGAATTGGAAAAGGAAGTGAACCGAAGGATAAAACTTTCCAGGGGATCATGA

>mRNA_121754_cds

ATGGCCGCCAGCAGAATTTCCTTTACTTCCAGCTCTCAAACCATTTATTTCCGTCACGG
CCCGTTTCTATGCCCAAATCCGGTTTTCCCAATCTCTTCCTTTTCTCGTAATCTTGGCAC
AATTCTGAGGTCTAGAAGAAAACCCAGTTTCACAGTTTGCTTTGTTCTGGAGGATGAG
AAGTTGAATACTCTATTTGACAAGTTGAATGCTCAATTAGAAACTGGGACAGAGGAG
ATTGAAATGAAAATTGAGGAGCAGATCTCAGCGACTCGTTTAGCGGAAAAAATTGGCT
AGGAAAAAAATCGGAGAGGTTTACTTATCTTGTGGCTGCGGTTATGTCTAGTTTTGGGA
TTACTTCCTTGGCTATTATGGCCGTCTATTACAGATTTTCGTGGCAAATGGAGGGTGGA
GAAGTGCCTTTAACTGAAATGTTTGGTACATTTGCTCTCTCAGTTGGTGCTGCTGTAGG
AATGGAGTTTTGGGCAAGGTGGGCTCATAGAGCGCTTTGGCACGATTCTCTTTGGCAC
ATGCACGAGTCACACCATAAACCAAGAGAAGGACCTTTCGAGATGAACGATGTGTTT
GCCATAATCAACGCCGTTCCAGCAATAGCCCTCCTCAACTATGGTTTCTTCCATAAAG
GCCTCATTCCTGGCCTCTGCTTCGGCGCTGGGCTAGGGATCACAGTATTTGGGATGGC
TTACATGTTCGTTCACGATGGTTTGGTTCACAAGAGATTCCCCGTCGGACCTGTAGCC
AATGTACCTTATTTTAGGAAGGTGGCCGCAGCTCATTTGCTCCATCACTCAGAGAAGT
TCAATGGTGTCCCATATGGTTTGTTCTTGGGACCAAAGGAATTGGAAGAAGTAGGGG
GGATTGAAGAATTGGAAAAGGAAGTGAACCGAAGGATAAAACTTTCCAGGGGATCA
TGA

>ε-OHase1_Ntab0299130

ATGCCATTTTCACTCACTCTCTCCTCTTTCTCTCTTCTCACTCACCCCACCACCCACCAC
CACCGAACCACCCATCTGGGCCCACTTCCTTATCGTTCACTTTCCGTCAAATCCTCCAT
AGACAACAAGAAACCACCTTCTACAAAGCCCAGTTCATGGGTCAGTCCAGATTGGTT
AACAAAACTAACCAGCTCACTTACTTTGGGCCAAAATGATGATTCTAATATACCCATT
GCAAGTGCTCAGCTTGAGGATGTTTCTGAACTATTAGGTGGTGCTCTTTTTCTTCCATT
GTTTAAATGGATGAATCAGTATGGACCCATTTATCGTCTTGCTGCTGGGCCTAGGAAT
TTTGTGATTGTCAGTGATCCTGCTATTGCTAAGCATGTTTTGAAGAATTATGGAAAGTA
TGGTAAAGGACTGGTTGCTGAGGTTTCTGAGTTTTTGTTTGGTTCTGGGTTTGCCATTG
CTGAAGGTCCTCTTTGGACGGTAATTTTGCTTTCCTTTATTTTTGTTTTGTTAATTATGC
AACTTGATTGGTTTTGGAATGAATTGGGCCCAAATTGAGCAGAGTGGATAAAGTTGA
TTTATATAGCTTACTCCAACTAGTTTGGGATTGAGGCGTAGATGTTGTTGAAATATGC
AGCTTCACATGCACATTTAGTAGATATTGCTTTGACACTCACTTGGTGTTTATCAGTGT
TTTAAAAGGGTGGACGTAAGGCGGAGCTTTTAACATATGCCTCAGCGGGGCGTAAGC
GAACGTCTTACATATACCTCAGCGGGGCGTAAGCCGTAAGCCCCATAGGTATTTAATT
TTTAATATTTTATAAAATAATATAATGACAGGAAATATTTATAAACAGGTAAAATTGC
ATAAATATTTAAGAAAACTATATATATGTGTGCTCCATCCCCACAAAAAACTAATCA
AAATAATCTATTATACATTACTTACAAGCACAAGTAATTTGAGTCGAAAAGAATAAA
ATTTTGTATCATTTGAGTCTAAAAGAATAAAGTTTTCTATATGGAGGAACAAAAAGG

ATGATTAACCTGCAATTTGAACTTTGAATTTGTTGCTATGAAGAAAAATGTAGTTCTCT
TTGTATTTGTAAAAAAATTAAATATTCGTTGCTTTTGGGAGATAGTAGCAGACTAGCG
GACAAGATAAAAAATTGGGAAAACCATGAATTATGGCTTAAATCAATAAAAAAGGT
CTTTACTTTTAAATTTAATACTTTTGAGTTCCTTTTTAAACCTTTTGAGTAATTACCAAA
CTGACTTTTGAGAATTTGGGTATTATATGAAGGACTAATTCAACAAATTTTGTTTTAAT
TCGAAAAAGTCTCTGGGGTTTACTCCTTACTAAAAAAACGCGCCCCGAACGCCCGGG
CGTACGCCCCAAATTGCGGGGCGTACGCCTCTTGAGACTTTCGCCCCACACCATCGCC
CCGGGGCATTTTTGGTACGCCTCGCCCCAGGGCTCTAGAAACGCCTTTTAAAACATAG
GTGTTTATCAATGAAATTTATAATTTTTGATGGCATTTTGGTAATTCAACTTTAACTAC
ACTTTAAATATTAGTATATAGTTATATAGATTTATTGACTTATCTTAAGAAAATAAAG
GATAATGCAGCATAAAATGAAATATATTGGAATGAGTTAGGCCCAAGAAGAGCAAA
ATGGATAAAGAGGATTGGTATAGCTAATCCAACTTGTTTCGGATTGAGGCGTAGGTTG
CTCAATTTTATGCTTCCTTTGTAGTTGCATTGGAATGAAATGGGTTCAAACAATGGCG
GGTCCAAGATTTAAATTTGATAGGTTCAACATTTACATTTCTTAGTAATGAGCACATA
ATACTTTTGAAATTATGGGTTCAGAATTCAATACTATTTGTTAAAACTTTAGTGGTTTT
TCACATTTATATCTATGTTCCATGTTGCAACTACTGGGTTCAAATGAACCCAACAATT
ATGGCCTAAATCTGTCAGTAGATCCAAATAGAATGGAATGAATATAGAGGATTCATA
CAGTCAGCACAAGTAGTTAGGAATTGAGGTGTAGTAATGGATATTGTTGAATTATGC
AGCGTGCACCTCGTGGTCGAGTTTGACAGTGAGACTAGACAGTTCCGTTTTAGTTTGT
GCCAAACAAATGAGAGATCTTTAGTAGATGATTAAAATGAAAGGATATTTCTTATGA
AACATTAGTTTGGTAATTAGGGTCAATACTTTTGCATCTTCTATTTCTGTGGAGTTTGG
ACTCGGTCTGGTGAATGCTAATTTTCTTGATGAAATTTAGAATGTCCCAAGTATTCATC
TTTTCTGGCTAGTTTGAATTGTTGCATGTTGGTAGTTTAATTTGTGATATACACAGCAT
AAAACGAAGCACGCTTTAGGGTACTGAAAAGCTAATTTAATGTCAAATTCAATGGTG
ACCTCTAGTTTGGTATAAAACAATATAATATGAAGTTCCTGTAAACCATGGTTTATTA
TTGATGAATGTAATTGATGATTTTTAGTTAGTTTTTATAATAGAGGGAAAGTACTGTAC
CCTAGTGTAACAATATTATGGCCACCAATGGTGTTGTTTTGAGCTTAAACCTAATTTG
ATATAGGCAAGGCGAAGGGCTGTGGTTCCATCTCTTCACAAGAAGTACTTGTCAGTA
ATAGTTGATCGGGTCTTTTGCAGATGTGCTGAGAGAATGGTGGAGAAACTTACACCTG
ATGCAATTTCTGGCTCTGCAGTAAATATGGAGGCAAAGTTTTCTCAACTAACACTTGA
TGTTATTGGTCTTGCACTCTTCAATTACAATTTTGATTCCCTTACGACTGACAGTCCAG
TTATTGAAGCAGTTTACACTGCGTTAAAAGAAGCAGAACTCCGTTCAACTGATCTGTT
GCCGTATTGGCAGGCATGTCCTTCCTATACTTAAATGGTTTTTGCCTAACTTAATCTGT
TTGATCATTTTGACTGAAATCATCCATGTTTTTGTAGATCAAAGCCATATGTAAGGTCA
TCCCACGACAGATAAAGGCCGAAAATGCAGTTTCTTTAATCAGGCAAACTGTTGAAG
AGCTCATTGCAAAGTGCAGAGAGATTGTAGAATCTGAGGGTGAGAGGATTAATGAG
GATGAGTACGTGAATGATAGAGATCCAAGCATCCTTCGATTTTTGCTTGCTAGCCGAG
AGGAGGTTGGCCAATATTTTAACTCATTCGATATATAACTGATAGAATTTCCTTTATGT
TTATCGATTACGAAACTTCTTTGAAACTGAACTTCTTTTTGGTAAATAGCATCATCTAT
GGTATGTTAATGTCAAATACAGTTAGTACTACTGATGCAATTTTTTCTCTTTTGATAAG
TACCCCTGATGTAAGTTTTCATGAAATCATAGTCATAGCTCCCAAGGGTGTGCCATAA
CGACAATAAAGTGGTTAATTATCATAGAAGACCGGTGTTCAAATCTCAGTAGAGACA
AAAAAAATATTAGGTGATATCTTTCCATCTGCATAAGTACTGGCGGGTAGTCACTAGG
TACCTGTGCTAGTGGGAGATAGCAGGTACCCAGTGGAATAGTCGAGGTATGCTGAAG

TTGGTCCAGACACCACCAATATATAAAAAAGTACATACAACATACTTATAGGACTGT
TCTTTTTTCTTTTTCCTTGTAAAGTCTAGCAAAATAAAGATAAAGCAAAATATGTTTCT
TCATAGTTAAATGTAGTCTTTGAACATTCAAGTGAAGCCAGCTTAGTGGTAGTTTCTT
ATTCAGATTCGAGAACTCTCTTGTTGTTCTTCCATTGTTTCTTATCCTTTTGTTCAGTCTT
TTTCTATACATCATTTGTTTACTTTGCTTTAACTTTTTTTGAATTCGCTGAAGTAAAAGC
TATTAGTTTGAATGATTCTTAATATTGATGATATGATTTTGCTAATAGATCGGCCAACC
AAAGATACACATTGCATGCATATAAATATGCCACGTGGTGGCTGATTGACAACTCTCT
AGAATGAGAAATATCCACAGTGGCACCCGGGACATGACAAATATCCATTATGAGTGC
TGCCAAGTTTCAAACTAGTGTGATGGCAAGAAGCTCATCTTGAGCTTATACTTAGCTA
AACTCCATTCCGTAGTTTGGTACTACTGAGAAGGGAGTCCTCACGATGCTCGGTTGGT
GCCAATGAGCAGGGTTCTTGGAGAAAGGAAGTCTCTTCCCTCCTTATCTGCCATTATC
TATTAATGAGTGAATTAGGTGCAAGCCCGGTTGTTTATACAATGATGATAAACTTGAA
TTAGATCCTATACCCTTAGCCTATCTAATATGTATAGTTTCTTGAAGGAGATTCATACA
AATAGAACTCTTCTCTTGTACATTTCTAACAAGTTTCATAATATGACATCCATTAAAC
ATTTGAGTTAGAGACTTCGATTTGAATTTGATGTTTCCCTTGAAATAATCAGTATCCAC
AAACTTGGCCAATCAATCTGATTGTATTTTCCTCAAACCACAGCTTTCCCTCTCTGTAC
TACAACAGGCAATGCAATTGGTTTATAAGGACCATTGGCGTATAGCAGTATTATTTTA
ACAATCCAAAACTATAAGCAAAACCCAAATCTCGCCTTGTGACTTGCAATCTTTCAAT
TGACATGTTTGCTTCTTTTGGAGAAAAGGATTGGTCATAGCCTAACTTCTTAAAGACA
AAATATTGCAGATATAGCTTAATGTGCATTCTTCATACACGCTAACTCTCTGAATTTCA
GGTTTCAAGTGTACAACTTCGAGACGATCTTCTATCAATGCTAGTTGCTGGGCATGAA
ACCACAGGTTCAGTTTTGACTTGGACGTCATACCTGCTGAGTAAGGTATCTTTGATTG
ACCTACATCCTTGAATCCTTAGTTCCCTCTTAATTTATCATTCATTTTTATGTTTTTCCA
ACTTGAATGACAGCAATAAAGTGTGCAGCAGAGAAGTGCAAAGTTGCCTTTTTTTTTT
CTTAATATTTATGTGCATATTCTAGATGTATTTGCCACTTAAATTCAGCCTTTTCGACC
CTTTCACGTTTAACATGTTGCACATGGTTTCTTTTGGAGGTTAACACTCACTGCCACAA
GTACACTCAGAAAATTACCCAGCTCTTCTGTGTGTTTATGTCAAAGTCATGGTTTTAAT
AATTGAATGTAAAGTAAGGGAAAAAAAAAATGAAAAATAGGTTCTTCAATGTGTGAG
AAATTATCCTTGCAAGGCAACCCCTTTAGCTTGGTCGTACGCGGTATCTTTAGACATT
CGTTGTTCATTCATGTAAGTTGGAAACTATTTGAAGTCAACTGATTTCTTTTAACCTCT
TTACTTCTTTTGGCTTGTCAGAGGGAAAACTATTTGAAGTCAACCTAATTGTTGTGAGC
TCTGTCTCTATGACATTTACGTTAACTTAAATTCCTCCCCGTCCACAGGACAGTACATC
ATTATAAAGGTTGAATTTGAAGAAAAGTTGTCTTAACCTGTGATATATGGTGACTCAT
CTTAAAAAAAGAAAAAAAAAAACTGTGAAATATGGTGTTCCCAGCAACTAAAATAAA
TGCCATTTGAGGAGCTTTTTCTTACCCAAGGACAATACTACTTGGAAAACTCACTAAG
AATTGTTTTACTCATATGTTCTTAGCTAAAACGCATCGCTTGATGTCACTTATGGTAAT
GCATGTTAATCATGTTGTCATCACTTTCAACCATTATTCTGCTATTTAGGAATTCTTAA
CAGGTTTTGTTGCCCTTTTACCTCTATATGCACTTTTACCTGTTGTTCCCTCATATTTTAT
GTTCTTAATGCAGAACCCTTCCTCTTTGAAAAAAGCACATGAAGAAGTTGACAGAGT
TTTGGGAGGACGCAATCCGACTTATGAAGACATGAGGAATCTCAAGTTCTTGACACG
GTGCATAACTGAGTCACTCCGACTTTATCCACATCCACCTGTAAGTTCGTCAATTTCTT
CTCCTTGTGGACTATATCATTATGCATTATCTTCTGATAACCTGATTTTATGGAGAATT
GTCTTTATTAGGTTCTAATAAGAAGAGCTCTAGTAGCTGATGTGCTCCCTGGAAATTA
CAAGGTCAATGCTGGTCAAGATATAATGATTTCGGTATATAACGTTCATCATTCTTCA

GAGGTAAAATCTAACAGATTTTTCTGATCTTTTACACCCTTTCTTGTTACTGTTATTGAT
CTTTTTGGAAATTTAATTCCTAGAAATTGTTTCTGTTTACTCTTCAAGACGGTGTGAAT
CATTTCTGTCCGCTAATGGGTTATTTGGCTTTGCTAAATACCACTAATACATAAAATA
ATTGTCTTCGCTGTCTCTTTGTTTTAGGTATGGGAAAGAGCAGAAGAATTTGATCCTGA
AAGATTCGACTCAGAAGGTCCAGTTCCAAACGAAACGAATACTGACTTTAGGTATTC
ATTTCAGCCATTAGCTTTTCTCTATTCAGTTTTCTGCATTCAGTTTATACACAGCTTGTC
TATGTAGAAGATTATCATTCATGTATTTACTTGTTGCAGATTCATTCCGTTTAGTGGAG
GGCCTCGAAAATGTGTTGGCGATCAATTTGCATTGTTGGAAGCTACAGTTGCTCTTGC
AATATTTTTGCAGAACTTCTCGTTTGAGTTGATTCCAGATCAAAACATTAGCATGACT
ACTGGAGCAACCATTCATACGACAAACGTGAGATTATCACCTAAGAGAGCTATCTTC
TTGTTCTGAGCTGTTTAAACGCGTTCTCCCCGATTTAAATTCTGTTTTAAATCTGTCTGT
TTGTTCCATTGCAGGGTTTATACATGAAAGTGAAGCAAAGGCAAAAAGAATCTGTGT
TGGCTGCATGA

>mRNA_131608_cds

ATGCCATTTTCACTCACTCTCTCCTCTTTCTCTCTTCTCACTCACCCCACCACCCACCAC
CACCGAACCACCCATCTGGGCCCACTTCCTTATCGTTCACTTTCCGTCAAATCTTCCAT
AGACAACAAGAAACCACCTTCTACAAAGCCCAGTTCATGGGTCAGTCCAGATTGGTT
AACAAAACTAACCAGCTCACTTACTTTGGGCCAAAATGATGATTCTAATATACCCATT
GCAAGTGCTCAGCTTGAGGATGTTTCTGAACTATTAGGTGGTGCTCTTTTTCTTCCATT
GTTTAAATGGATGAATCAGTATGGACCCATTTATCGTCTTGCTGCTGGGCCTAGGAAT
TTTGTGATTGTCAGTGATCCTGCTATTGCTAAGCATGTTTTGAAGAATTATGGAAAGTA
TGGTAAAGGACTGGTTGCTGAGGTTTCTGAGTTTTTGTTTGGTTCTGGGTTTGCCATTG
CTGAAGGTCCTCTTTGGACGGCAAGGCGAAGGGCTGTGGTTCCATCTCTTCACAAGA
AGTACTTGTCAGTAATAGTTGATCGGGTCTTTTGCAGATGTGCTGAGAGAATGGTGGA
GAAACTTACACCTGATGCAATTTCTGGCTCTGCAGTAAATATGGAGGCAAAGTTTTCT
CAACTAACACTTGATGTTATTGGTCTTGCACTCTTCAATTACAATTTTGATTCCCTTAC
GACTGACAGTCCAGTTATTGAAGCAGTTTACACTGCGTTAAAAGAAGCAGAACTCCG
TTCAACTGATCTGTTGCCGTATTGGCAGATCAAAGCCATATGTAAGGTCATCCCACGA
CAGATAAAGGCCGAAAATGCAGTTTCTTTAATCAGGCAAACTGTTGAAGAGCTCATT
GCAAAGTGCAGAGAGATTGTAGAATCTGAGGGTGAGAGGATTAATGAGGATGAGTA
CGTGAATGATAGAGATCCAAGCATCCTTCGATTTTTGCTTGCTAGCCGAGAGGAGGTT
TCAAGTGTACAACTTCGAGACGATCTTCTATCAATGCTAGTTGCTGGGCATGAAACCA
CAGGTTCAGTTTTGACTTGGACGTCATACCTGCTGAGTAAGAACCCTTCCTCTTTGAA
AAAAGCACATGAAGAAGTTGACAGAGTTTTGGGAGGACGCAATCCGACTTATGAAG
ACATGAGGAATCTCAAGTTCTTGACACGGTGCATAACTGAGTCACTCCGACTTTATCC
ACATCCACCTGTTCTAATAAGAAGAGCTCTAGTAGCTGATGTGCTCCCTGGAAATTAC
AAGGTCAATGCTGGTCAAGATATAATGATTTCGGTATATAACGTTCATCATTCTTCAG
AGGTATGGGAAAGAGCAGAAGAATTTGATCCTGAAAGATTCGACTCAGAAGGTCCA
GTTCCAAACGAAACGAATACTGACTTTAGATTCATTCCGTTTAGTGGAGGGCCTCGAA
AATGTGTTGGCGATCAATTTGCATTGTTGGAAGCTACAGTTGCTCTTGCAATATTTTTG
CAGAACTTCTCGTTTGAGTTGATTCCAGATCAAAACATTAGCATGACTACTGGAGCAA
CCATTCATACGACAAACGGTTTATACATGAAAGTGAAGCAAAGGCAAAAAGAATCT
GTGTTGGCTGCATGA

>ε-OHase2_Ntab0895820

ATGCCATCTTCACTCGCCCTTTCTTCTTTCTCTCTTCTCACTCACCCCACCACCCACCAC
CACCGAACCACCCATCTGGGCCCACTTCCATGCCATTCACTTTCCATTAAATCTTCCAT
AGACAACAAGAAACCACCTTCTACAAAGCCCAGTTCATGGGTCAGTCCAGATTGGTT
AACAAAACTAACCAGCTCACTTACTTTGGGCCAAAATGATGATTCTAATATACCCATT
GCAAGTGCTCAGCTTGAGGATGTTTCTGAACTATTAGGTGGGGCTCTTTTTCTTCCATT
GTTTAAATGGATGAATCAGTATGGACCCATTTATCGACTTGCTGCTGGCCGAGGAAT
TTTGTAATTGTTAGTGATCCTGCTATTGCTAAGCACGTTTTGAAGAATTATGGAAAGTA
TGGTAAAGGGCTTGTAGCTGAAGTGTCTGAGTTTTTGTTTGGTTCTGGTTTTGCCATTG
CTGAAGGTCCTCTTTGGACGGTAATTTTGCTTTCCTTTATTTTGTTTTGTTAATTATGCA
ACTTGATTGGTTTTGGAATGAAATGGGCCCAAATTGAGCACAGTGGATATATTTGATT
TATATAGCGTACTCCAACTAGTTTGGCATTGAGGCGTACATGTTGTTGAAATATGCAC
TTCGCATGCACATTTTGTAGATATTGCTTTGACACTCACTTGGTGTTTATCAATGAAAT
TTATAATTTTCGATGACATTTTGGTAATTCAACTTTAACTAAGGAGCTTCCCACTTTTA
TATTAGTATATATATATATATATATATATAGATTTATTGACTTATCTTAAGAGAATAAA
GGATAATGCAGCATAAAATGAAATATATTGGAATGACTAATGAGTTAGGCCCAAGA
AGAGCAAAATGGATATAGAGGATTGGTATAGCTAATCCAACTTGTTTCGGATTGAGG
CGTAGGTTGCTGAATTTTATGCTTCCTTTGTAGTTGCATTGGAATGAAATGGGTCCAAA
CAGAAGCGGGTCCAAGATTTATCAAAAATTTGATAGGTTCAACTTTTACATTTCTTAG
TAATGAACACATAATACTTTTGAAATTATGGGTTCAGAATTCAATACTATTTGTTAAA
TTTTTTTAGTGTTTTTTCACATTTATATCTATATTCCATGTTGCAACTACTGGGTTCAGA
TGAACCCAACAATTATGGCCTAAATCTGTCAGTAGATCCAAATAGAATGGAATGAAT
ATAGAGGATTCATACAGTCAGCGCAAGTGGTTAGGAATTGAGGCGTAGTAATGGATA
TTGTTGAATTATGCAGCATGCACCTCATGGTCAAGTTTGACAGTGAGACACAGTTCCC
TTTTAGTTGGTGCCACACAAATGAGAGATCTTTAGTAGATGATTAAAATGAAAGGAT
AATTCTTATGAAACATTAGTTTGGTAATTAGGGTCAATACGTTTACATCTTCTATTTCT
GTGGAATTTGGACTCGGTCTGGTGAATGCTAATTTTCTTGATGAAATTTAGATGTCCCA
AGGATTCATCTTTTCTGGCTAGTTTGAATTGTTGCATGTTGGTAGTTTAATTGTGATAC
ACACGGCATAAGACAAAGCACACTTTAGGGTACTGAAAAGCTAATTTAATGTCAAAT
TCAATGGTGACCTCTAGTTTGGTATAAAACAATGTAATATGAAGTTCCTGTAAACCAT
GGTTTATTATTGATGAATGTAATTGATGATTTTTAGTTAGTTTTTATAATAGAGGGAAA
GTACTGTACCCTAGTGTAACAATATTATGGCCACCAATGGTGTTGTTTTGAGCTTAAA
CCTAATTTGATATAGGCAAGGCGAAGGGCTGTGGTTCCATCTCTTCACAAGAAGTACT
TGTCAGTAATAGTTGATCGGGTCTTTTGCAGATGTGCTGAGAGAATGGTGGAGAAACT
TACACCTGACGCAATTTCTGGGTCTGCAGTAAATATGGAGGCAAAGTTTTCTCAACTA
ACACTTGATGTTATTGGTCTTGCACTCTTCAATTACAACTTCGATTCCCTTACTACTGA
CAGTCCAGTTATTGAAGCAGTTTACACTGCGTTAAAAGAAGCAGAACTCCGTTCAAC
TGATCTGTTGCCATATTGGCAGGCATGTCCTTCCTATACTTGAATGGTTTTTGCCGAGG
TTAATCTATCTGATTATTTTGACTGAGCTTCCATGTTTTTGTAGATCAAAGTCTTATGTA
AGGTCATCCCACGACAAATAAAGGCTGAAAATGCAGTTTCTTTAATCAGGCAAACTG
TTGAAGAGCTCATTGCAAAGTGCAGAGAGATTGTAGAATCTGAGGGTGAGAGGATTA
ATGAGGATGAGTACGTGAATGATAGAGATCCAAGCATCCTTCGATTTTTGCTTGCTAG
CCGAGAGGAGGTTGGCCAATAGTTAAACTCATTTGATACATAACTGATAGAATTTCCT
TTCTGTTTTGGATTACAAAACTTCTTTGAAACTGAACTTCATTTAGGTAAATAGCATCA

TCTATGATATGTTAAGGTGAACTACAGTTAGTACCACTGTTGTAAGAATTCATGCAAT
CAGAGTCATAGTCATAGCTCCCAAGGGTATGGCCTAACAGTCAATTAAGTGGTGAAA
ATCATAGAGCTAGGGTTCAAATCTCAGCAGACACAAATTACTTAGGTGATTTCTTCCC
ATTTGCCCGAGTCTTGGTGGGCAAAGTTACTAGGTACCTGTGCTAGTGGGAGGTAGCA
GGTACCCTGTGGAATAGTCGAGGTGTACGCAAGTTGGCCCGAACACCACCAATATAT
AAAAGAGTCCATACAACATACTTATAGGACTATGTTCTTTTTTCTTTTTCCTTGTATAG
TCTAGCAGAATAAAGATAAAGCAAAATTTTCTTCTGGCCTTTATAACTGTTGGTTGCTT
TGTTATATTCAGAGTTAATGTAGTTGATGAACAATCAAGTGAAGCCAGCAATGGTAGT
TTCTTGTTCAGATTGTAGAACTCTATTGTTGTTCTTCCATTGTTTCTCTTTTTCTAAATAT
CATTTGTTTACTTTTCTTTGACATTTTTGAGTCAGCTGAAATAAAGAATTATTAGTTCG
AATGATTCTTAATATTGATGATATGATTTTCTAGTAGATCGGCCAACTAAAGATGCAC
ATTGCATGCGTATAAAAATATCACATGGTGGCTCATTGACTTATTGGAAAACTTCTTC
CTCAATCCTATAGGATTTGACAACTTTCTATATTGAGAAACATCCAAAATGGCCCTGG
GACATGAGAATTACCCATTTTGGGTGACGCCAAGTTTCAAACTGGTGTGATGCCAAG
AATCTCATCTTGAGCTTATACTTAGCTAAACTCCATCTTCCATAGTCTGGCACCACTGA
GAAGGGAGTCCTCAGGTTGCTCGCTTGGTGCCAATGAGCAGGGTTCTTAGAGGAAGG
ACGTCTCTTCCTTTCTTAGCTGCCATTATCAATTGATGAGTGAATTAGGTGCAAGTCCG
GTTGTTTATACAATGATGATAAACTTGAATTAGTGCCTATATCCTTAGCGTATCTAATA
TGTATAGTTTCTTGAAGGAGATTCATACAAATTTGACATGTCTTAGTGCTACGACTTTT
TAACTTTTCCCTTTTTCCTTTTTTTTTTCTCCCCGGTTTTTTCCTGCGGGGTTGGTTTGGG
ATGTGCGTGTGGGTGGGTGGGGGTAAATATGAATCTTGCACATTTCTAACTAGTTTCTT
AATTTTACATCCGTTAAACATTTGAGTTAGAGACTTCAATTAGGATTTGATGTTTTCAA
TGAAATACTCCCTCCATTTCAATTTAGATGAGGTAGTTTGACTCAGCACAGAGTTTAA
GAAAAAAAAGACTTTTAAAACTTGTGGTCTTAGAAGCTTAAGGGGTAAAAGCTTTGT
AGGCCCATGACATTTGTGTGGTTATAAAAGCTTCTCATTAAGGGTAAAATGGGTAAA
ATGAAGAGTTTAAAGTTGAATTATTTCCAATTGTAGAAATGTGTCATTCTTTTCGGAAC
GGACTAATAAAGAAAGTGTGTCATCTAAATTGAAACAGAGGAGTAATCATTATTCAC
AAACTTGGCCATTCAATCTGATTGCATTTCCCTCAAACCAGAGCTTCAGTCTCTCTAAT
TCAACACGCAATGCCCTTGGTTTATAAAGACCATTGGTGTATATCAGTATTGTTTCAA
CAAACCAAAACTATAAGCGAAATCCAAATCTTGCCATGTGACTTTCAATCATTCAATT
GACATGTTTACTTCTTTTTGGAGAAAAGGATTGATCATAACATATCTTCTTAAAGACA
AAATACTGCAGATATAGCTTAATATGAATTCTCTATGCATGCTAACTTTCTGAATTTCA
GGTTTCAAGTGTACAACTTCGAGATGATCTTCTATCCATGCTAGTTGCTGGGCATGAA
ACCACAGGTTCAGTTTTGACTTGGACGTCATACCTGCTGAGTAAGGTGTCTCCAGTTG
ACCTTCATCCTTGAATCCTTAGTTCCCTCTTAATTTATCACACATTTTATGTTTTTCCAA
CTTGAATGACAGCAATAAACTGTGCCACAGCGAAGTGCAAAGTTACATTTCTTTTAA
AATATTTTTGTGCATATTCTAGATGTATTCGCCACCTTAAATTCAGCTTTTCGACCCTTT
TACGTTTAACATGTTGCTCATGGTTTCTTATGGAGGTAAGCACTTACTGCCACAAGTA
CAGTCAGAAAATTACCCAACTCTTCTGTGTGTTGTCAGAGGAGGAACTATTTGAAGGC
AACCTAATTGTTGTGAGTTCTGTCTCCATGACATTGACGTTAACTAAAATTCCTTCCTG
TCCACAGGACAGTAAATCATTTTAAAGGTTGAATTTGAAGAAAAAATGTCTGAGCCT
GTGATGTATGGTGACTTATCTAAAAAAGAGAAAAAAGCCTGGGAAATATGGTGCTCC
CAGCAGCTAAAAGAAATGCCATTTGAAGAGCTTTTTCTTACCGAACAATAATACTGTT
TGGAGAACTTACTGAGAATTGTTTTAGTCGTATGTTCTTAGCTAAAACGCATTTGCATG

ATGTCACTTATGGTAATGCATGTTAATCATGTTGTCATCACTGTCAACCATTATTCTGC
TATTTAGGAATTCTTAACAGGTTTTGTTGCCCTTTTACCTCTATATAGGTGGAAATCCA
GTTGTTTCATTCTAGTTTTCGTTTTGGATATGTTTTGGTCTTTCTTTAAAAAAAATTGTC
GATCCTGTGAGCATATTTATTTCTATAATAATTCATAATCTTCTATTCGATAAAAACAT
GGTGCGTGACGGAGACTATGCAGACTATAAAAACCTGTTGTTCCCTCACATTTTATGT
TCTTAATGCAGAACCCTTCCTCCTTGAAAAAAGCACATGAAGAAGTTGACAGAGTTTT
GGGAGGACACGCTCCGACCTATGAAGACATGAGGAATCTGAAGTTCTTGACACGGTG
CATAACTGAGTCACTCCGACTTTATCCACATCCACCTGTAAGTTCGTCAATTTCTCTTC
TCCTTGTGGACTTTATCGTTATGCATTATCTTCTTATAACCTGATTTTATGGAGAACTCT
CTTTATTAGGTTCTAATAAGAAGAGCTCTAGAAGCTGATGTGCTCCCTGGAAATTACA
AGGTCAATGCTGGTCAAGACATAATGATTTCGGTATATAACGTTCATCATTCTTCAGA
GGTAAAATCTAAGAACTTTTTTTTATCACTCTTTCTTGTTACTGTTATTGAGCTTTTTTG
GAAATTTTCCTTGAAATTGTTTCTGCTTACTCTTCAAGTTGGTATGAATGATTCCTGTCC
GCAAATGGGTTCTTCTTCTTCTTCATGGGGCTATAGGACATCTGTTTGACTTGGTCTCT
AACAACTATTTCAATCATTTGTCTATTCCTTATTGCCTGTTTATGTGGATATATGGATCC
ACCTTTTCCACTTTGGCTTTGCTTAAATTAAGCCTACAACTGGGAGGGAGCTTTCTGGT
CTGTTTCAAGGAACAGAACTGGATCCTATATATTGTCCCACCATTTAATTTAAATTTTT
TTTTTGGTTGCGAAATACATTTCAAGGCTCATTTTTTATCCAATGAAGTGTTCTGTTCCT
GCCTTTGAAAGGCACAAATACATAAAATAATTGTCTTCGCTGTCTCTTTATTTTAGGTA
TGGGAAAGAGCAGAAGAATTTGATCCTGAAAGATTCGACTTAGAAGGTCCAGTTCCA
AACGAAACAAACACTGACTTTAGGTATTCATTTCAGCCAATAGCTTGTCTGAGTAGA
AGATTATTACTCTATGCAGTTTTCTACATTCAGTTTATGCACAGCTTGTCTGAGTATCG
TAGAAGATTGTCATTCATGCATTTACTTGTTGCAGATTCATCCCATTTAGTGGAGGGCC
TCGAAAATGTGTTGGCGATCAATTTGCATTGTTGGAAGCTACAGTTGCTCTTGCAATA
TTTTTGCAGAACTTGTCGTTTGAGTTGATTCCAGATCAAAACATTAGCATGACTACTG
GAGCAACCATTCATACGACAAACGTAAGATTCTCACCTAAGACTAAGAGAGCTATCT
TCTTGTTCTAAACTGGTTAACACCTCCTCCTATAAATTCTTATGTCTGTTTGTTCCGTTG
CAGGGTTTATACATGAAAGTGAAGCAAAGGCAAAAAGAATCTGTTTTGGCTGCATAA

>mRNA_140553_cds

ATGCCATCTTCACTCGCCCTTTCTTCTTTCTCTCTTCTCACTCACCCCACCACCCACCAC
CACCGAACCACCCATCTGGGCCCACTTCCATGCCATTCACTTTCCATTAAATCTTCCAT
AGACAACAAGAAACCACCTTCTACAAAGCCCAGTTCATGGGTCAGTCCAGATTGGTT
AACAAAACTAACCAGCTCACTTACTTTGGGCCAAAATGATGATTCTAATATACCCATT
GCAAGTGCTCAGCTTGAGGATGTTTCTGAACTATTAGGTGGGGCTCTTTTTCTTCCATT
GTTTAAATGGATGAATCAGTATGGACCCATTTATCGACTTGCTGCTGGGCCGAGGAAT
TTTGTAATTGTTAGTGATCCTGCTATTGCTAAGCACGTTTTGAAGAATTATGGAAAGTA
TGGTAAAGGGCTTGTAGCTGAAGTGTCTGAGTTTTTGTTTGGTTCTGGTTTTGCCATTG
CTGAAGGTCCTCTTTGGACGGCAAGGCGAAGGGCTGTGGTTCCATCTCTTCACAAGA
AGTACTTGTCAGTAATAGTTGATCGGGTCTTTTGCAGATGTGCTGAGAGAATGGTGGA
GAAACTTACACCTGACGCAATTTCTGGGTCTGCAGTAAATATGGAGGCAAAGTTTTCT
CAACTAACACTTGATGTTATTGGTCTTGCACTCTTCAATTACAACTTCGATTCCCTTAC
TACTGACAGTCCAGTTATTGAAGCAGTTTACACTGCGTTAAAAGAAGCAGAACTCCG
TTCAACTGATCTGTTGCCATATTGGCAGATCAAAGTCTTATGTAAGGTCATCCCACGA

CAAATAAAGGCTGAAAATGCAGTTTCTTTAATCAGGCAAACTGTTGAAGAGCTCATT
GCAAAGTGCAGAGAGATTGTAGAATCTGAGGGTGAGAGGATTAATGAGGATGAGTA
CGTGAATGATAGAGATCCAAGCATCCTTCGATTTTTGCTTGCTAGCCGAGAGGAGGTT
TCAAGTGTACAACTTCGAGATGATCTTCTATCCATGCTAGTTGCTGGGCATGAAACCA
CAGGTTCAGTTTTGACTTGGACGTCATACCTGCTGAGTAAGAACCCTTCCTCCTTGAA
AAAAGCACATGAAGAAGTTGACAGAGTTTTGGGAGGACACGCTCCGACCTATGAAG
ACATGAGGAATCTGAAGTTCTTGACACGGTGCATAACTGAGTCACTCCGACTTTATCC
ACATCCACCTGTTCTAATAAGAAGAGCTCTAGAAGCTGATGTGCTCCCTGGAAATTAC
AAGGTCAATGCTGGTCAAGACATAATGATTTCGGTATATAACGTTCATCATTCTTCAG
AGGTATGGGAAAGAGCAGAAGAATTTGATCCTGAAAGATTCGACTTAGAAGGTCCA
GTTCCAAACGAAACAAACACTGACTTTAGATTCATCCCATTTAGTGGAGGGCCTCGA
AAATGTGTTGGCGATCAATTTGCATTGTTGGAAGCTACAGTTGCTCTTGCAATATTTTT
GCAGAACTTGTCGTTTGAGTTGATTCCAGATCAAAACATTAGCATGACTACTGGAGCA
ACCATTCATACGACAAACGGTTTATACATGAAAGTGAAGCAAAGGCAAAAAGAATC
TGTTTTGGCTGCATAA

>VDE1_Ntab0858420

ATGGCTCTTGCCCCTCATTCAAACTTTCTCGCCAACCATGAAACCATCAGATATTATG
TCGGGTCAAAGTTTCCCGGTCATAAAAGGTTTAGCTGGGGATGGGAAGATTACTTTGG
TAATATATTCGTAGCGAAAATTTGTTCCAGCAGACGGATACCTAGATACTTTCGAAAA
TCTCCTAGAATATGCAGCAGTTTGGATTCAAGAAGTTTGCAACTATTGTCACACGGGA
AACACAATCTCTTTCCCGCACATAGCATTAACCAGAATGTATCTAAGGTGACTTTTGA
TAAACCTTTACTCTTGGATTTCATGCCATTGCTTATCATTAACTACTTATTTTCGAAGG
GAATGTCAAGATCTAGCTCTGTCATCTACATTCTTTTCATCCTCTTCTTTCTCTTTCCTA
ACACAAGTCCTCTTTTAGGGAAATTCAGGAAGCAAATTTCCAAAAGATGTAGCTTTG
ATGGTTTGGGAGAAATGGGGCCAATTTGCCAAAACAGCAATTGTAGCTATATTCATTT
TGTCAGTTGCTTCAAAAGCTGATGCGGTTGATGCTCTCAAGACTTGTACTTGCTTACTG
AAAGAGTGCAGGTAATTCGTGCAGATGTTACATAAGTATGCCGGAATTTTTCCTTATA
CTAAGCTATTACTAATATATTTTGCTGCACAAGTATCAAGACTAGTTTCTTCTACGAA
AAAATGAATTTGATAAGAAGATGTCAGTAAACAACTTAAAGCTTTCCTGATTGCTTTG
CTACTCCTTGTATGTTTCCTACATGAGTTTCGAACTTGATTTCTTCTATCCCATGTATAA
ATATAAGGATGTGAAATTGATTCTTGTTTGTTCTCTCAGGATTATCATCCTGGCTTAAG
AATGAAGTTGTAAAGATATAAAATTTTCAGTTTCAGCGGTGCTATCTTATCTCAACCT
GCTCACCCTGAAAGATGTCAGGTTGACCAAACTGTCTCTAGATTTTTCTGATCAACTG
TCCATAACTCCCCGACTCTAATCGCTGTTACAATCAAAGAAATCAATTTTTAAGCATG
TCCAAAGTGTATCATAATCTCAACCATAATGCTTTTCTTTCTACCTCCATTTTGATTGTG
AGACTTAGATTTTCTGTTATGTAATTAGGCCAAGAAAGTAGTAGTATGAGTTTGCCTT
GTTAACAAGTTTCGTCAAACCATTTGTTTTTTTATGGTGGTAAAATAACTGATGTGTTG
CTGTGCTAGGGATGCAGGATACCCCTTCTATAATATTTGCATTCCCAAGGATGCAATG
GTTGAAAACTAAACTAAGTTAACATCGTTGTCTAATCCTTAATCTATGGCTCTTTGATA
TTCTAGCAGCTTTTCTGTTTTTTCTTCAAATCCGTTTAGTTGTGACTTGTTTGCTTATTTT
GTTATCCACCTTCTCATGTGAGAGGAAGGAGAACTAGGTGGCAAAAAATGACAGGAA
ATCTCATAGAAGAGAAATGCTATATTAGTAATTATGAGTTTCTAACCTTCGTTTCAGG
CCTCTCTGCTCGCTCGGGGTAGGGGTAAGGTCTGCGTACTCACTACCCTCCCCAGACC

CCACTTGTGGGATTTCACTGGGTTGTTGTTGTAACCTTCATTTCAGCATGTATGTCAGC
ACTCTTATGAGACTAAGAAAAGTATTATGCTTTCAGGTTAGAGCTTGCGAAGTGCATT
GCAAACCCTGCATGTGCAGCTAATGTTGCCTGTCTCCAGACTTGCAACAATAGACCTG
ACGAAACGGAATGTCAGGTGTCTACAAATTTCTTATTAAACTCAATATTCTTGTGATC
ACATTTGTAGTGTAATAATATGCTGGAGTGATTCTGCAGATAAAATGTGGTGATTTGT
TTGAGAACAGTGTTGTAGACGAGTTCAATGAGTGTGCAGTCTCCCGAAAGAAATGTG
TACCTCGTAAATCTGATGTTGGTGACTTTCCTGTACCTGATCCAAGTGTTCTTGTCCAG
AAGTTTGACATGAAAGATTTTAGCGGGAAATGGTTCATAACACGCGGTTTAAATCCC
ACTTTTGATGCTTTTGATTGTCAATTGCATGAGTTCCATACAGAAGAAAACAAACTTG
TGGGGAATTTATCGTGGAGAATAAGGACACCTGATGGAGGATTTTTACTCGATCAGC
GGTGCAAAAATTTGTGCAAGATCCAAAGTATCCAGGGATACTCTACAATCATGATAA
TGAGTATCTTCACTACCAAGATGACTGGTATTTCCCTAACTATCTATTTAACATCGTGT
CTCCATACTGGATTAAACCTTGCTGATAGATAAATATCTGGGAGGGGGGCGGAGCTA
GCGCTTCGATTACGGGTTCGGCCTAACCCAATAGCTTTAGTCTTAACCCTATATTTGTC
TTAAGAACCCAATATGGCTTCTAAATATTAATTTAGATCCCAATAACTTGAAAAGAAT
AGAATCCTAAACGCAGAAACTTCAAAATCCTGGCTCCACATCCGTATCTAGGATTGG
CAAGAGATGATGAGTTGTTAAATCCTATCTTGAAATTAGTTCCGTATCTCGTGTGCAA
AATTGGACCTTGATGTTGTACTTGGTTTCCAATTGACATATTCTGTGTATTATTAAAAC
ATGTGAATTGATGTTTTCTGCAGGTATATTTTGTCATCCAAAGTAGAAAATAGTCCAG
AGGATTACATATTTGTGTACTATAAGGGCAGAAATGATGCATGGGATGGATATGGTG
GTTCTGTACTTTACACAAGAAGTGCAGTTTTGCCTGAAAGCATTATACCCGAGTTGCA
AACCGCCGCTCAAAAAGTTGGCCGTGATTTCAACACCTTCATAAAAACAGACAATAC
ATGTGGCCCTGAACCTCCCCTTGTTGAGAGGTTGGAGAAGAAGGTGGAAGAAGGCGA
GAGGACGATCATAAAAGAAGTCGAGGAGATAGAAGAAGAAGTAGAGAAGGTGAGA
GATAAAGAAGTCACCTTATTCAGTAGACTGTTTGAAGGTTTTAAAGAGCTCCAACGA
GATGAAGAGAACTTCTTAAGAGAGCTGAGCAAAGAAGAAATGGATGTTTTGGATGG
ACTTAAAATGGAGGCAACTGAGGTAGAAAAACTTTTTGGGCGTGCTTTACCAATAAG
GAAGTTAAGGTAA

>mRNA_114230_cds

ATGGCTCTTGCCCCTCATTCAAACTTTCTCGCCAACCATGAAACCATCAGATATTATG
TCGGGTCAAAGTTTCCCGGTCATAAAAGGTTTAGCTGGGGATGGGAAGATTACTTTGG
TAATATATTCGTAGCGAAAATTTGTTCCAGCAGACGGATACCTAGATACTTTCGAAAA
TCTCCTAGAATATGCAGCAGTTTGGATTCAAGAAGTTTGCAACTATTGTCACACGGGA
AACACAATCTCTTTCCCGCACATAGCATTAACCAGAATGTATCTAAGGGAAATTCAG
GAAGCAAATTTCCAAAAGATGTAGCTTTGATGGTTTGGGAGAAATGGGGCCAATTTG
CCAAAACAGCAATTGTAGCTATATTCATTTTGTCAGTTGCTTCAAAAGCTGATGCGGT
TGATGCTCTCAAGACTTGTACTTGCTTACTGAAAGAGTGCAGGTTAGAGCTTGCGAAG
TGCATTGCAAACCCTGCATGTGCAGCTAATGTTGCCTGTCTCCAGACTTGCAACAATA
GACCTGACGAAACGGAATGTCAGATAAAATGTGGTGATTTGTTTGAGAACAGTGTTG
TAGACGAGTTCAATGAGTGTGCAGTCTCCCGAAAGAAATGTGTACCTCGTAAATCTG
ATGTTGGTGACTTTCCTGTACCTGATCCAAGTGTTCTTGTCCAGAAGTTTGACATGAAA
GATTTTAGCGGGAAATGGTTCATAACACGCGGTTTAAATCCCACTTTTGATGCTTTTG
ATTGTCAATTGCATGAGTTCCATACAGAAGAAAACAAACTTGTGGGGAATTTATCGT

GGAGAATAAGGACACCTGATGGAGGATTTTTACTCGATCAGCGGTGCAAAAATTTG
TGCAAGATCCAAAGTATCCAGGGATACTCTACAATCATGATAATGAGTATCTTCACTA
CCAAGATGACTGGTATATTTGTCATCCAAAGTAGAAAATAGTCCAGAGGATTACAT
ATTTGTGGACTATAAGGGCAGAAATGATGCATGGGATGGATATGGTGGTTCTGTACTT
TACACAAGAAGTGCAGTTTTGCCTGAAAGCATTATACCCGAGTTGCAAACCGCCGCT
CAAAAAGTTGGCCGTGATTTCAACACCTTCATAAAAACAGACAATACATGTGGCCCT
GAACCTCCCCTTGTTGAGAGGTTGGAGAAGAAGGTGGAAGAAGGCGAGAGGACGAT
CATAAAAGAAGTCGAGGAGATAGAAGAAGAAGTAGAGAAGGTGAGAGATAAAGAA
GTCACCTTATTCAGTAGACTGTTTGAAGGTTTTAAAGAGCTCCAACGAGATGAAGAG
AACTTCTTAAGAGAGCTGAGCAAAGAAGAAATGGATGTTTTGGATGGACTTAAAATG
GAGGCAACTGAGGTAGAAAAACTTTTTGGGCGTGCTTTACCAATAAGGAAGTTAAGG
TAA

>VDE2_Ntab0230700

ATGGCTCTTGCCCCTCATTCAAATTTTCTGGCCAACCATGAAACCATCAAATATTATG
TTGGGTCAAAGCTTCCCGGTCATAAAAGGTTTAGCTGGGGTTGGGAAGATTACTTTGG
TAGTATAGTCGTAGCAAAAATTTGTTCCAGCAGACGGATACCTAGATACTTTCGAAA
ATCTCCTAGAATATGCTGTGGTTTGGATTCAAGAGGTCTGCAACTATTCTCACACGGG
AAACACAATCTCTCTCCCGCACATAGCATTAACCAGAATGTACCTAAGGTGACTTTTG
ATAAATCTTTACTCTTGGATTTCATGCTGTTGCTTATCATTAACTACTTATTTCGAAGG
GAATGTCGGGATCTAGCTCTGTCTCTGCATTCTTTTCATCCTCTTCTTTCTCTTTGCTAA
CACAAGTCCTCTTTTAGGGAAATTCAGGATGCAAATTTCCAAAAGATGTAGCTTTGAT
GGTTTGGGAGAAATGGGGCCAATTTGCCAAAACAGCAATTGTAGCTATATTCATTTTG
TCAGTTGCTTCAAAAGCTGATGCGGTTGATGCTCTCAAGACTTGTACTTGCTTACTGA
AAGAGTGCAGGTAATTCGTGCAGATGTTACTAAAGTATGCCGGAATTTTTCCTTATAC
TAAGCTATTAATATATTTTGCTGCACAAGTATCAAGACTAGTTTTTTCTACGAAACACT
GAATTTGATAAGAAGATGTCAGTAAACAACTTAAAGCTTTCCTGATTGCTTTGCTACC
CCTTGTATGTTTCGTACATGAGTTTCGAACTTGATCTCTGCTATCCCATGTATAAATAT
AAGGATGTGAAATTGATTCTTGTTTGTTCTCTCGGGATTATCATCCTGGCTTAAGAATG
AAGTGGTAAAGATGTAAAGCTTTCAGTTTTGGCGGTGCTATCTTATCTTAACCTGCTC
ACCCTGGAAGATGTCAGGTTGACCAAACTGTCTCTAGAATTTTCTGATCAACCGTCCA
TAACTCCTCGACTCTAATCGCTGTTACAATCAAAGAAATCAAGTTTTAAGCATGTTCA
AAGTGTATCATAATCTCAACCATAATGCTTTTCCTTCTACCTCCATTTTGATTGTGAGA
CTTAGATTTTCTGTTATGTAATTAGGCCAAGAAAATAGTAGTATGAGTTTGCCTTGTTA
ACAAGTTTCGTCAAACCATTTGTTCCCTTTTTTTTATTTTTTTTTATTTTATGGTTGTAAA
AATAACTGATGTGTTGCTGTGCTAGGGATACAGGAAAGTTTCGCCAATCCCTGGACCC
CAGAAGACAGCTAAATTTGTCTCGCTGAATAAGTCAATATTTAAAATGAAATTCATTT
TACTCTAAAACTTCTAACAACCCAATTGTTTTCCTTGTCTTACCTGCCCCTTATTTTTTC
TCTGCAAAACAAGGAAACCAAATATTACCTAATCAACTTTCTGGATACCCCTTCTAA
AATATTTGCATCCCAAAGGATGCAATGGTTGAAAACTAAACTAAGTTAACATCGTTG
TCTAATCCTTAATCTATGGCTCTTTGATATTCTAGCAGCTTTTCTGTTATATTTTCTTTCT
GTTTTTTCTTCAAATCCGTTTAGTTGTGACTTGTGTGCTTATTTTGTTATCCACCTTCTGA
TGTGAGATGAAGGAGAAATGCTATATTAGTTATTATGAGTTTCTAACCTTCATTTCAG
CATGTGTGTCAGCACTCTTGGGAGACTAAGAAAAGTATTATGCTTTCAGGTTAGAGCT

TGCGAAGTGCATTTCGAACCCTGCATGTGCAGCTAATGTTGCCTGTCTCCAGACTTGC
AACAATAGACCTGACGAAACGGAATGTCAGGTGTCTACAAATTTCTTATTAAGCTCA
TTATTCTTGTGATCACATTTGCAGTATACTAATATGCTGGAGTGATTCTGCAGATAAA
ATGTGGTGATTTGTTTGAAAACAGTGTCGTAGACGAGTTCAATGAGTGTGCAGTCTCC
CGAAAGAAATGTGTACCTCGTAAATCTGATGTTGGTGACTTTCCTGTACCTGATCCCA
GTGTTCTTGTCCAGAAGTTTGACATGAAAGATTTTAGCGGGAAATGGTTCATTACTCG
CGGTTTGAATCCCACTTTTGATGCTTTTGATTGCCAATTGCATGAGTTCCATACAGAAG
AAAACAAACTTGTGGGGAATTTATCTTGGAGAATACGTACACCTGATGGAGGATTTTT
TACTCGATCAGCGGTGCAAAAATTCGTGCAAGATCCAAAGTATCCGGGGATACTCTA
CAATCATGATAATGAGTATCTTCACTACCAAGATGACTGGTATTTCTCTTAATACCTAT
TTGACGTCATGTCTCCATACCGGACTAAACCATGCTGATAGATAAATATCTTGGCAGG
GGCGGAGCTTCCGTTTCGGTTACGGGTTCGGCCGAACCCAGTAGCTTTGGTCTTTACC
CTACCCTACATTTGTCTTAAGAACCCAATATGGCTGCTAAATTAGTATTTTAGAACCC
AATAACTTAAAAATAATAGAATCTCGAACCTATAAACTTCAAATCCTGACTCCGCTTT
TGTATCTAGGCTTGGCATGAGATGACGAGTTGTTAAATCCTATCTTGAAATTCGTTCTA
TATCTCGTGTGCAAAATTGGACCTTGATGTTGTACTTGGTTTGCAATTGACCTCTTCTG
TGTATTAATAAACATGTGAATTGATGTTTTCTGCAGGTATATTTTGTCATCCAAAGTAG
AAAATAGTCCAGAGGATTACATATTTGTGTACTATAAGGGCAGAAATGATGCATGGG
ATGGATATGGTGGTTCTGTACTTTACACAAGAAGTGCAGTTTTGCCTGAAAGCATTAT
ACCGGAGTTGCAAACCGCAGCTCAAAAAGTTGGGCGTGATTTCAACACATTCATAAA
AACAGACAATACATGTGGCCCTGAACCTCCCCTTGTTGAGAGGTTGGAGAAGAAAGT
GGAAGAAGGAGAAAGGACGATCATAAAAGAAGTTGAGGAGATAGAAGAAGAAGTA
GAGAAGGTGAGAGATAAAGAAGTCACCTTATTCAGTAAACTGTTTGAAGGTTTTAAA
GAGCTCCAACGAGATGAAGAGAACTTCTTAAGAGAGCTGAGCAAAGAAGAAATGGA
TGTTTTGGATGGACTTAAAATGGAAGCAACTGAGGTAGAAAAACTTTTTGGGCGTGCT
TTACCAATAAGGAAATTAAGGTAA

>mRNA_119637_cds

ATGGCTCTTGCCCCTCATTCAAATTTTCTGGCCAACCATGAAACCATCAAATATTATG
TTGGGTCAAAGCTTCCCGGTCATAAAAGGTTTAGCTGGGGTTGGGAAGATTACTTTGG
TAGTATAGTCGTAGCAAAAATTTGTTCCAGCAGACGGATACCTAGATACTTTCGAAA
ATCTCCTAGAATATGCTGTGGTTTGGATTCAAGAGGTCTGCAACTATTCTCACACGGG
AAACACAATCTCTCTCCCGCACATAGCATTAACCAGAATGTACCTAAGGGAAATTCA
GGATGCAAATTTCCAAAAGATGTAGCTTTGATGGTTTGGGAGAAATGGGGCCAATTT
GCCAAAACAGCAATTGTAGCTATATTCATTTTGTCAGTTGCTTCAAAAGCTGATGCGG
TTGATGCTCTCAAGACTTGTACTTGCTTACTGAAAGAGTGCAGGTTAGAGCTTGCGAA
GTGCATTTCGAACCCTGCATGTGCAGCTAATGTTGCCTGTCTCCAGACTTGCAACAAT
AGACCTGACGAAACGGAATGTCAGATAAAATGTGGTGATTTGTTTGAAAACAGTGTC
GTAGACGAGTTCAATGAGTGTGCAGTCTCCCGAAAGAAATGTGTACCTCGTAAATCT
GATGTTGGTGACTTTCCTGTACCTGATCCCAGTGTTCTTGTCCAGAAGTTTGACATGAA
AGATTTTAGCGGGAAATGGTTCATTACTCGCGGTTTGAATCCCACTTTTGATGCTTTTG
ATTGCCAATTGCATGAGTTCCATACAGAAGAAAACAAACTTGTGGGGAATTTATCTTG
GAGAATACGTACACCTGATGGAGGATTTTTTACTCGATCAGCGGTGCAAAAATTCGT
GCAAGATCCAAAGTATCCGGGGATACTCTACAATCATGATAATGAGTATCTTCACTA

CCAAGATGACTGGTATATTTTGTCATCCAAAGTAGAAAATAGTCCAGAGGATTACAT
ATTTGTGTACTATAAGGGCAGAAATGATGCATGGGATGGATATGGTGGTTCTGTACTT
TACACAAGAAGTGCAGTTTTGCCTGAAAGCATTATACCGGAGTTGCAAACCGCAGCT
CAAAAAGTTGGGCGTGATTTCAACACATTCATAAAAACAGACAATACATGTGGCCCT
GAACCTCCCCTTGTTGAGAGGTTGGAGAAGAAAGTGGAAGAAGGAGAAAGGACGAT
CATAAAAGAAGTTGAGGAGATAGAAGAAGAAGTAGAGAAGGTGAGAGATAAAGAA
GTCACCTTATTCAGTAAACTGTTTGAAGGTTTTAAAGAGCTCCAACGAGATGAAGAG
AACTTCTTAAGAGAGCTGAGCAAAGAAGAAATGGATGTTTTGGATGGACTTAAAATG
GAAGCAACTGAGGTAGAAAAACTTTTTGGGCGTGCTTTACCAATAAGGAAATTAAGG
TAA

>VDE3_Ntab0721110

ATGAATGTAAACGTAGCACCATCTTCCTCCATCAATTCTCCGGCTACTTTTCCGGTTAA
CCGGCGGTCCAGTGTCGCCGGACTTCTAAATCCTCTTCTTCTCCGCTGCCGGAAAACT
AAAGTGTCCGTCTTTTCTTCTTCTCTTGACCGGAAAATCAGAGCTACAAGAAGAATAC
TAGCTGCTGCTGCTCTGGAAATGAAAGAAGCAGAGAAAAAAACAAGTAGTCCAGTG
AGAATAGTGAGTATTGTTGGTGAGGATAGTGTTAGCCCTCTCAATTCTGCTCCTTGGCT
AGATGTCATGCTTCACACTGTGAGTATTTTGCAACTTCATTCACTGTCTTTGACTCCAA
ATTTATATATATACTATATATATAAAGGGTTAAAACATCATACAAATATATATATTAA
TGAGTTTAAGTTTTGTGCCAGTGTAAAAAATATGTACACAATCAGTCACTACTTTAAT
AGCACTGATCGGTAATCTTTAATAAATGACAATAACCTACTATAATAAGTTAAATTAT
ACCGATAGTGTAAAAACTTAATTAGACTGTAAGTGCACATAACTTAAATCCTGTATG
AGTACATATTGTTAGAAATTAGTGGTTACATTGATATTCCATATCGGTTAATTTAGGAT
CCGTTTGGCTGTTTTTTTGTTTTACATTGATATTCCATATCGGTTACTTTAGGATTCGTTT
GGCTGAATTTTGGCTATTTTTTTCAAAATAAATTTGAGAACATTGTTTGTTCATGAGAT
TTGATCAGTTTCTGAAAAAAAAAAATTAAAAACATTATCAAGTTCCCAAAAACTGGT
TTAGCGAAAAGATTTCTTCAACTCACAAAACTTCGACTTTTTTGTCAAATAAAATACA
TGTCCAAACACTACTTCGACTTCCAAAAATTATTTTAACACAACTTCAAAAATTTATT
TTTCAAGTTTCAACCAAATATATGTCCAAACGCTAGCTTAAAATCTTGAACTCAGTGC
TAGGGATTTTGTTTTTAAAATTCTTTATTTTTTTTCTTTTGTGTGAATTATTCTTAGTGTC
ATGTGTTTCAACTAGGAGTATTAGGTGCTTTAGCTCAAATGAAATGTGGAACCATAAA
TCAAAATTGGAAATGACAATTATTGGATGTTGGGCTGACAATTATTGGAAATGATGTA
TAATATTTAGAAAATACTATAATTTTGCTGCTGTTGACGGCTTGAAATTGAGGCAGTG
CAATTCAAATTTCTTGGGGAGTTGAAATTTTCTCAAAGTTCAGTGTTTTGTTTTGTATA
CAATGGAAAGGGTTGATGATTCTAAGGAGTTTATCCGCCTTTTTCTTTTTCAAATATCA
CATTAGTAGAGGAAGAGGGTCATTAGTTTATAAGGAGTTCTAGTTTTTTGGAGTAAAT
AAGACCAACTGTGTTTTACCAAGTTCTGGCTTTGAAGAGTTTGTAGATGTTAGAGATT
ATTTTCTCGCTTTTTTCTTCAAGGTGGGAAGGAGGATGAGTATTTTCTGATTCTGTTAG
ATCCGTTTACTTTTTGAGTAATATTGTCAATAGCACATTGTGGCAGCCTGATTAGAAC
AAGGGTGTTTTTCATTTCTACCTATTCTTTCCTCGATATTCTTATTGGAAAGTAAAGAG
GAAGCTCTCACAATTTGAAGTAGTTTTTTTCAGTAAATAAGAAACAAGAAACTGAAC
AAAGCGATAAAATATTTGACATATTACTACTTGATAGACAGCTGAAGTTCTGTTTTAC
TGTAACATCATCATGGAAAGTTGATGATTAGCAATGTTAGTTGCTTGTTGTGCTGCTCT
CAAGAGGAACTTGTGAGATTTTCAGTATAACAAGATAAACTGGTGAACAGAAACATT

GTAATTACCAGAGAAAAAGAAAACGAAAAGTATTATGTAATAGCAGGGAGCAAAAT
GGGAAGAATGTAATATGCATCTACAACTTCTACAATGTGGAAAACCAAATGACCAAT
TATTTATGAATAAGCTCTGGCCTCTTGTATTTCTGGTAGAGACTTTTCAAGTAGGAAG
GAGGTCCTGATGCTCATTGTAGCACTACCCAAACACTACTCTAAAATCTCTCTCTCCC
CCTTCTATGTATATGCACACAAGCAGATTCTTATTAGACATTAGAGTAGAAATAGCTT
GTGTATTGTGAGAACATTGCTTCCCTTCACAGTTATGAGAATATACATATTAGTTGAT
GATATTGTACTTTGTTTCCTTTTATAAATGCAAGGCAGAGAGACTGAAATGGGTTGAT
GAAGAGTTTGAAATGATTGTCTTTTCTGACAACTTTATCAATTCTCAAGATGAAACTG
TCAACCGCGTTAGAAATGAGCTGGATCGTGCTGATATTTTGGTGCTGGTTGCTGTTAG
TAAGGAAGGATCAGTCAACTGGATACAGACTAACAGCCAGAATGTCCCAAATACCA
TATGCTTTGATTCCTCTTCGGCACTGAGAAACAAGCTGGGCGGAATTTTGATTGAAAC
TGAAAAAAGAGGAGACATATTCAGCAGTTTACTTCCATTCTCTCAATCTAAGAAAAT
GAATGAATCTGTAGAGATAGTTCGAACAGTATCTGAGGCTTGGGAAAGGCATAACGC
TGATGACATACGGTTCTGCTTATTAGTTATAATTAATTCTTATATAACACCGGTTTCAA
CACTGCAGAACCTTAGAGCAAAAGGATTCTCCACCCTGAACTGCATGGTGACAAATT
GCGGACCTCAGATATTGAATTGTCTACTGGATCCTAATTGTAGGAAGGCACTTCAGTG
CTTGAACCAGTGCAGCCCTGTAGATCAAGTATGCAATTATCGGTGTATTGCTTCATAT
GAGAGTCAATACCTGGAAGAGTTTTCTCTCTGTGTACTACAGAAGAATAACTGTCTTG
AGCTCGACGCAAAGATCCCTGAAAAACCTTATGTGCCTCCAATGACCGAGTTTCGAG
GGGAAATATTGTCTTCTGAAATAGCTGAGGACCTTTTTGTGGGTTGGTTAGGGACATT
GAATTGGAGTTGGCGCGTTGTAGCAGGGCAGAATCCAGCTTATGATCAATTTCCATGC
CAGTACCAGCTATACTATCGGGGAAAAGCTAGAGGATCATTCTGGTATGAGCCAGTT
TTCCAGGTAAGAACACTTGAAGATAACTTAGTCTGGAGAAGGCGCAAGTATAGAGTG
AAAAGAGGAAAAGTTCCCGGAACATTTCAATTCAGTGTATTGGATAATGGAGTTGTTT
CAATTGAGTCGTGGACAATTGTGGATGTTTCTGATGATTTAAGCTGGGGTTTGTTTCAC
TATCATGGAGCTGCACGAGTGGCGGGGCAGTCATATACCGGGGCGGTTCTTGTGAGC
CCAGATGGCCAATATCCAGCTGAGAGGGAAAAAGAAAGGTTGGTATCTGCATTGGAT
AGATGTGGCATCAAAGAGTGGGAGCTATTCAATGTTGATAATTGTTCATGTGAAGGTC
CACCATTGGGGCTTCCGGAGGGGTCAAGTTTGCATTCTAAGATTGAAGTCCAAGAGG
GCAGGACACATGCTTCAGTATAG

>mRNA_130498_cds

ATGAATGTAAACGTAGCACCATCTTCCTCCATCAATTCTCCGGCTACTTTTCCGGTTAA
CCGGCGGTCCAGTGTCGCCGGACTTCTAAATCCTCTTCTTCTCCGCTGCCGGAAAACT
AAAGTGTCCGTCTTTTCTTCTTCTCTTGACCGGAAAATCAGAGCTACAAGAAGAATAC
TAGCTGCTGCTGCTCTGGAAATGAAAGAAGCAGAGAAAAAAACAAGTAGTCCAGTG
AGAATAGTGAGTATTGTTGGTGAGGATAGTGTTAGCCCTCTCAATTCTGCTCCTTGGCT
AGATGTCATGCTTCACACTGCAGAGAGACTGAAATGGGTTGATGAAGAGTTTGAAAT
GATTGTCTTTTCTGACAACTTTATCAATTCTCAAGATGAAACTGTCAACCGCGTTAGA
AATGAGCTGGATCGTGCTGATATTTTGGTGCTGGTTGCTGTTAGTAAGGAAGGATCAG
TCAACTGGATACAGACTAACAGCCAGAATGTCCCAAATACCATATGCTTTGATTCCTC
TTCGGCACTGAGAAACAAGCTGGGCGGAATTTTGATTGAAACTGAAAAAAGAGGAG
ACATATTCAGCAGTTTACTTCCATTCTCTCAATCTAAGAAAATGAATGAATCTGTAGA
GATAGTTCGAACAGTATCTGAGGCTTGGGAAAGGCATAACGCTGATGACATACGGTT

CTGCTTATTAGTTATAATTAATTCTTATATAACACCGGTTTCAACACTGCAGAACCTTA
GAGCAAAAGGATTCTCCACCCTGAACTGCATGGTGACAAATTGCGGACCTCAGATAT
TGAATTGTCTACTGGATCCTAATTGTAGGAAGGCACTTCAGTGCTTGAACCAGTGCAG
CCCTGTAGATCAAGTATGCAATTATCGGTGTATTGCTTCATATGAGAGTCAATACCTG
GAAGAGTTTTCTCTCTGTGTACTACAGAAGAATAACTGTCTTGAGCTCGACGCAAAGA
TCCCTGAAAAACCTCATGTGCCTCCAATGACCGAGTTTCGAGGGGAAATATTGTCTTC
TGAAATAGCTGAGGACCTTTTTGTGGGTTGGTTAGGGACATTGAATTGGAGTTGGCGC
GTTGTAGCAGGGCAGAATCCAGCTTATGATCAATTTCCATGCCAGTACCAGCTATACT
ATCGGGGAAAAGCTAGAGGATCATTCTGGTATGAGCCAGTTTTCCAGGTAAGAACAC
TTGAAGATAACTTAGTCTGGAGAAGGCGAAAGTATAGAGTGAAAAGAGGAAAAGTT
CCCGGAACATTTCAATTCAGTGTATTGGATAATGGAGTTGTTTCAATTGAGTCGTGGA
CAATTGTGGATGTTTCTGATGATTTAAGCTGGGGTTTGTTTCACTATCATGGAGCTGCA
CGAGTGGCGGGGCAGTCATATACCGGGGCAGTTCTTGTGAGCCCAGATGGCCAATAT
CCAGCTGAGAGGGAAAAAGAAAGGTTGGTATCTGCATTGGATAGATGTGGCATCAA
AGAGTGGGAGCTATTCAATGTTGATAATTGTTCATGTGAAGGTCCACCATTGGGGCTT
CCGGAGGGGTCAAGTTTGCATTCTAAGATTGAAGTCCAAGAGGGCAGGACACATGCT
TCAGTATAG

>VDE4_Ntab0189070

ATGAATGTAAACGTAGCACCATCCCCCTCCATCAATTCTCCGGCTCCTTTTCCGGTTA
ATCGGCGGTCCAGTTTCGCCGGACTTCCAAACCTTATTCTTCTCCGCTGCCGGAAAAC
TAAAGTGTCCATATTTTCTTCTTCTCTTGACCGGAAAATCAGAGCTACAAGAAGCATA
GTAGCTGCTGCTGCTGTGGAAATGAAAGAAGCAGAGAAAAAGACAAGTAATCCAGT
GAGAATAGTGAGTATTGTTGGAGAGGATAGTGTTAGCCCTCTCAGCTCTGCTCCTTGG
CTAGATGTCATGCTTCACACTGTGAGTATTTTGCAACTTCATTCACTCTCTGACTCCAA
ATTTATATTGTATATATTAATGAGTTTAAGTTCTGTGTCAGTGTAAAAAGTATGTACAC
AATCAGTCACTATTTTAATAGTATTGATTGGTAATCTTTAGTAAATGAGAAGTAATCT
GTTATAATAAGTTATGTTACACCGATGGTGTAAAAAATTAATTACATTGTAAGTGCAT
ATAACTTAAATCCTGTATTAGTACATATTGTTAAATTAGTGGCTACATTGATATTCATA
TCGGTTAATTTAAAATCTTGAACTCGGTGCTTGGGAAGTTTCATGTGTTTCAACTAGGA
GTATGAGGTGCTTTAGCTCAAACGAAATGTGGAACCATAAATCAAAATCGAGTATTT
TTTGGATGTTGGGCTGACAATTATAGGATATGATGTATAATATTTAGAAATATTATAA
TTTCGCTGATGTTGACAGCTTGAAATTGAGGCACTGCAATTCAAATTTCTTGGGAAGT
TAAAATTTCCTCAAAGTTCAGTGTTTTGTTTTGTATACAATGGAAAGGGTTGATGATTC
TAAGGAGTTTATCCACCTTTTTCTTTCCAAATATCACATTAGTAGAGGAAGAGGGTCT
TTAGTTTATAAGGAGTTCTAGTTTTTTGGTGTAAACGAGACCAACTGTGTTTTACCAAG
TTCTGGCTTTGAAGAGTTTGTAGATGTTAGAGATTATTTTCTTGCTTTTTTCTTCAAGGT
GGGGAGTATTTTCTGATTCTCTTAGATCCGTTTACTTTTTGAGTAATATTGTCAATAGC
ACATTGTGGCAGCCTGATTAGAACAACGGTGTTTTGCATTTCTACCTATTCTTTCCTCG
ATATTCTTATTGGAAAGTAAAGAGGAAGCTCTCACAATTTGATGTAGTTTTTTTCAGTA
AATAAGAAACAAGAAACTGAACAAAGGGATAAAATATTTGACATATTACTACTTGAT
AGACAGCTGAAGTTCTGTTTTACTGTAACATCATCATGAAAAGTTGATGAGTAATGTC
CGCTGCTTGTTGTGCTGCCCCCAAGAGGAACTTGTGAGATTTTCAGTATAACAAGATA
ACCTGGTGAACAGAAACATTGTAATTACCAGAGAAAAAGAAAGCTAAAAGTATTAC

TATGTAATAGCAGGGAGCAAAATGGGAAGAATGTAATATTCATCTACAACTTCTACA
ATGTGGAAAACCAAATGACCAATTATTTATGAATAAGTTCTGGGCTCTTATATTTTCT
GCTAGAGACTTTTCAAGTAGGAAGGGGGTCCTGGTGCTCATTGTAGCACTACCTAAA
CACTACTCTAAATCTCTCTCTCCCCCTTCTATGTATATGCACACAAGCAGATTCTTATT
AGACATTAGAGTAGAAATAGCTTGTGTGTTGTGAGAACATTGCTTCCCTTCACAGTTA
TGAGATACATATTAGATTGTACTTTGTTGACGATATTGTATTTTGTTTCCTTTTATAAAT
GCAAGGCAGAGAGACTGAAATGGGTTGATGAAGAGTTCGAAATGATTGTCTTTTCTG
ACAACTTTATCAATTCTCAAGATGAAACTGTCAACCGCGTTGGAAATGAGCTGGATC
GTGCTGATATTTTGGTGCTGGTTGCTGTCAGTAAGGAAGGATCAGTCAACTGGATACA
GACTAACAGCCAGAATGTCTCAAACATCATATGGTTTGATTCCTCTTCGGCACTGAGA
AACAAGCTGGGCGGAATTTTGATTGAAACTGAAAAAAGAGGAGATATATTCAGCAG
TTTACTTCCATTCTCTCAATCGAAGAAACTGAATGAATCTGTAGAGATAGCCCGAACA
GTGTCTGAGGCTTGGGAAAGGCATAACTCTGATGACATGCGGTTCTGCTTATTAGTTA
TAATTAATGCTTATATAACGCCGGTTTCAACACTGCAGAACCTTAGAGCAAAAGGAT
TCTCCACCCTGAACTGCATGGTGACAAATTGTGGACCTCAGATATTGAATTGCCTACT
GGATCCTAATTGTAGGAAGGCACTTCAGTGCTTGAACCAGTGCAGCCCTGTAGATCA
AGTATGCAATTATCGGTGTATTGCTTCATATGAGAGTCAATACCTGGAAGAGTTTTCT
CTCTGTGTACTACAGAAGAATAACTGTCTTGAGCTCGACGCAAAGATCCCTGAAAAA
CCTCATGTGCCTCCAATGACCGAGTTTCGAGGGGAAATATTGTCTTCTGAAATTGCTG
AAGACATTTTTGTGGGTTGGTTAGGGACATTGAATTGGAGTTGGCGCGTTGTAGCAGG
GCAGAATCCAGCTTATGATCAATTTCCATGCCAGTACCAGCTATACTATCGGGGAAA
AGCTAGAGGATCATTCTGGTATGAGCCAGTTTTCCAGGTAAGAACACTTGAAGATAA
CTTAGTCTGGAGAAGGCGAAAGTATAGAGTGAAAAGAGGAAAAGTTCCCGGAACAT
TTCAATTCAGTGTATTGGATAATGGAGTTGTTTCAATTGAGTCCTGGACAATTGTGGAT
GTTTCTGATGATTTAAGTTGGGGTTTGTTTCACTATCATGGAGCTGCACGAGTGGCGG
GGCAGTCATATACTGGGGCGGTTCTTGTGAGCCCAGATGGCCAATATCCAGCTGAGA
GAGAAAAAGATAGGTTGATATCTGCATTGGATAGATGTGGCATCAAAGAGTGGGAG
CTATTCAATGTTGATAATTGTTCATGTGAAGGTCCACCACTGGGGCTTCCAGAGGGGT
CAAGTTTGCATTCTAAGATTGAAGTCCAAGAGGGCAGGACACATTCTTCAGTATAG

>mRNA_86361_cds

ATGAATGTAAACGTAGCACCATCCCCCTCCATCAATTCTCCGGCTCCTTTTCCGGTTA
ATCGGCGGTCCAGTTTCGCCGGACTTCCAAACCTTATTCTTCTCCGCTGCCGGAAAAC
TAAAGTGTCCATATTTTCTTCTTCTCTTGACCGGAAAATCAGAGCTACAAGAAGCATA
GTAGCTGCTGCTGCTGTGGAAATGAAAGAAGCAGAGAAAAAGACAAGTAATCCAGT
GAGAATAGTGAGTATTGTTGGAGAGGATAGTGTTAGCCCTCTCAGCTCTGCTCCTTGG
CTAGATGTCATGCTTCACACTGCAGAGAGACTGAAATGGGTTGATGAAGAGTTCGAA
ATGATTGTCTTTTCTGACAACTTTATCAATTCTCAAGATGAAACTGTCAACCGCGTTGG
AAATGAGCTGGATCGTGCTGATATTTTGGTGCTGGTTGCTGTCAGTAAGGAAGGATCA
GTCAACTGGATACAGACTAACAGCCAGAATGTCTCAAACATCATATGGTTTGATTCCT
CTTCGGCACTGAGAAACAAGCTGGGCGGAATTTTGATTGAAACTGAAAAAAGAGGA
GATATATTCAGCAGTTTACTTCCATTCTCTCAATCGAAGAAACTGAATGAATCTGTAG
AGATAGCCCGAACAGTGTCTGAGGCTTGGGAAAGGCATAACTCTGATGACATGCGGT
TCTGCTTATTAGTTATAATTAATGCTTATATAACGCCGGTTTCAACACTGCAGAACCTT

AGAGCAAAAGGATTCTCCACCCTGAACTGCATGGTGACAAATTGTGGACCTCAGATA
TTGAATTGCCTACTGGATCCTAATTGTAGGAAGGCACTTCAGTGCTTGAACCAGTGCA
GCCCTGTAGATCAAGTATGCAATTATCGGTGTATTGCTTCATATGAGAGTCAATACCT
GGAAGAGTTTTCTCTCTGTGTACTACAGAAGAATAACTGTCTTGAGCTCGACGCAAAG
ATCCCTGAAAAACCTCATGTGCCTCCAATGACCGAGTTTCGAGGGGAAATATTGTCTT
CTGAAATTGCTGAAGACATTTTTGTGGGTTGGTTAGGGACATTGAATTGGAGTTGGCG
CGTTGTAGCAGGGCAGAATCCAGCTTATGATCAATTTCCATGCCAGTACCAGCTATAC
TATCGGGGAAAAGCTAGAGGATCATTCTGGTATGAGCCAGTTTTCCAGGTAAGAACA
CTTGAAGATAACTTAGTCTGGAGAAGGCGAAAGTATAGAGTGAAAAGAGGAAAAGT
TCCCGGAACATTTCAATTCAGTGTATTGGATAATGGAGTTGTTTCAATTGAGTCCTGG
ACAATTGTGGATGTTTCTGATGATTTAAGTTGGGGTTTGTTTCACTATCATGGAGCTGC
ACGAGTGGCGGGGCAGTCATATACTGGGGCGGTTCTTGTGAGCCCAGATGGCCAATA
TCCAGCTGAGAGAGAAAAAGATAGGTTGATATCTGCATTGGATAGATGTGGCATCAA
AGAGTGGGAGCTATTCAATGTTGATAATTGTTCATGTGAAGGTCCACCACTGGGGCTT
CCAGAGGGGTCAAGTTTGCATTCTAAGATTGAAGTCCAAGAGGGCAGGACACATTCT
TCAGTATAG

>VDE5_ Ntab0607170

ATGAATGTAAACGTAGCACCATCCTCCTCCATCAATTCTCCGGCTACTTTTTCGGTTAA
CCGGCGGTCCAGTGTCGCGGGGATTCCAGACCCTTTTCTTCTCTGCTACCGGAAAACT
AAAGTGTCCGTCTTTTCTTCTTCTCTTGACAGGAAAATCAGAGCTACAAAAGGCATAG
TTGTTGTTGCTGCTGTGGAACTGAAAGAAGCAGAGAAAAAGACAAGTAATCCAGTGA
GAATAGTGAGTCTTGTTGGAGAGGATAGTGTTAGCCCTCTCAATTCTGCTCCTTGGCT
AGATGTCATGCTTCACGCTGTGAGTATTTTGCAACTTCATTCACTCTCTTTGACTCCAA
ATTTATATTGTATGTATTAATGAGTTTGAGTTTTGTGTCAGTGTAAAAAGTATGTACAC
AATCAGTCACTACTTTAATAGTATTGATTGGTGATCTTTAATAAATGATAAGTAATCT
GTTATAATAAGTTAAATTACACCGATGGTGTAAAAAATTAATTATACTGCAAGTGCGT
ATAACTTATCATGTATTGGTACATATTGTTAAATTAGTTACATTGATATTCATATCGGT
TATTTTAAAATCTTGAACTCGGTGCTTGGGAAGTTTCATGTGTTTCAACTGGGAGTATG
AGGTGCTTTAGCTCAAACGAAATGTGGAACCATAATCGAGTATTTTTTGGATGTTGGG
CTGACAATTATTGGAAATGATGTATAATATTTAGAAAATATTAAACTTTCGCTGATGT
TGACAGCTTGAAATTGAGGCAGTGCAATTCAAATTTCTTGGAGAGTTGAAATTTTCTC
AAAGTTCAGTGTTTTGTTTTGTATACAATGGAAAGGGTTGATGATTCTAAGGAGTTTAT
CCGCCTTTTTCTTTTTCAAATATCACATTAGTAGAGGAAGAGGGTCATTAGTTTATAAG
GAGTTCTAGTTTTTTGGAGTAAATGAGACCAACTGTGTTTTACCAAGTTCTGGCTTTGA
AGAGTTTGTAGATGTTAGGGATTATTTTCCTGCTTTTCTCTTCAAGGTGGGAAGGAGG
ATGAGTATTTTCTGATTCTCTTAGATCCGTTTTCTTTTATTGAGTAATATTGTCAATAGC
GCATTGTGGCAGCCTGATTAGAACAAGGGTGTTTTGCATTTCTACCTATTCTTTCCTCG
ATATTCTTATTAGAAAGTAAAGAGGAAGCTCTCACAATTTGAAGTAGTTTTTTTGAGT
AAATAAGAAACAAGAAACTGAACAAAGGGATAAAATATTTGACATATTACTACTTG
ATAGACAGATGAAGTTCTGTTTTACTGTAACATCATCATGAAAAGTTGATGATGAGCA
ATGTCCGCTGCTTGTTGTGCTGCTCTCAAGAGGAACTTGTGAGATTTTCAGTATAACA
AGATAACCTGGTGAACAGAAACATTGTAATTACCAGAGAAAAAGAAAGCAAAAAGT
ATTATGTAATAGTAGGGAGCAAAATGGGAAGAATGTAATATGCATCTACAACTTCTA

CAATGTGGAAAACCAAATGACCAATTATTTATGAATAAGTTCCGGCCTCTTATATTTT
CTGCTAGAGACTTTTCAAGTAGGAAGGAGGTCCTGGTGCTCATTGTAGCACTACCCAA
ACACTACTCTAAAATCTCTCTCTCCCCCTTCTATGTATATGCACACAAGAATATTCTTA
TTAGACATTAGAGTAGAAATAGCTTGTGTATTGTGAGAACATTGCTTCCCTTCACAGT
TATGAGAATATACATATTAGATTGTACTTTGTTGACGATATTGTACCTTGTTTCCTTTTA
TAAATGCAAGGCAGAGAAACTGAAATGGGTTGATGAAGAGATTGAAATGATTGTCTT
TTCTGACAACTTTATTAATTCTCAAGATGAAACTGTCAACCACGTTAGAAATGAGCTG
GATCGTGCTGATATTTTGGTGCTGGTTGCTGTCTGTAAGGAAGGATCGGTCAACTGGA
TACAGACTAACAGCCAGAATGTCCCAAACATCATATGCTTTGACTCCTCTTCGGCACT
GAGAAACAAGCTCGGCGGAATTTTGATTGAAACTGAAAAAAGAGGAGACATATTCA
GCAGTTTACTTCCATTTTCTCAATCTAAGAAAATGAATGAATCTGTAGAGATAGCCCG
AATAGTGTCTGAGGCTTGGGAAAGGCATAACTCTGATGACATACGGTTCTGCTTATTA
GTTATAATTAATGCTTAATGAACGCCGGTTTCAACACTACAGAACCTTAGAGCAAAA
GGATTCTCCACCCTGAACTGCATGGTGACAAATTGCGGACCTCAGCTATTGAATTGTC
TACTGGATCCTAATTATAGGAAGGCACTTCAGTGCTTGAACCAGTGCAGCCCTGTAG
ATCAAGTATGCAATTATCGGTGTATTGCTTCATATAAGAGTCAATACCTGGAAGAGTT
TTCTCTCTGTGTACTACAGAAGAATAACTGTCTTGAGCTCGACGCAAATATCCCTGAA
AAACCTTATGTACCTCCAATGACCGAGTTTCGAGGGGAAATATTGTCTTCTGAACTTG
CTGAGGACCTTTTTGTGGGTTGGTTAGGGACATTGAATTGGAGTTGGCGCGTTGTAGC
AGGGCAGAATCCAGCTTATGATCAATTTCCATGCCAGTACCAGCTATACCATCGGGG
AAAAGCTAGAGGATCATTCTGGTATGAGCCAGTTTTCCAGGTAAGAACACTTTAAGA
TAACTTAGTCTGGAGAAGGCGCAAGTATAGAGTGAAAAGAGGAAAACTTCCTGGAA
CATTTCAATTCAGTGTATTGGATAATGGAGTTGTTTCAATTGAGTCCTGGACAATTGTG
GATGTTTCTGATGATTTAAGCTGGGGTTTGTTTCACTATCATGGAGCTGCACGAGTGG
CGGGGCAGTCATATACCGGGGCGGTTCTTGTGAGCCCAGATGGCCAATATCCAGCTG
AGAGGAAAAAAGAAAGGTTGGTATCTGCATTGGATAGATGTGGCATCAAAGAGTGG
GAGCTATTCAATGTTGATAAATGTTCATGTGAAGGTCCACCGTTGGGGCTTCCAGAGA
GGTCAAGTTTGCATTCTAAGATTGAAGTCCAAGAGGACAGGACACATTCTTCAGTAT
AG

>mRNA_95599_cds

ATGAATGTAAACGTAGCACCATCCTCCTCCATCAATTCTCCGGCTACTTTTTCGGTTAA
CCGGCGGTCCAGTGTCGCGGGGATTCCAGACCCTTTTCTTCTCTGCTACCGGAAAACT
AAAGTGTCCGTCTTTTCTTCTTCTCTTGACAGGAAAATCAGAGCTACAAAAGGCATAG
TTGTTGTTGCTGCTGTGGAACTGAAAGAAGCAGAGAAAAAGACAAGTAATCCAGTGA
GAATAGTGAGTCTTGTTGGAGAGGATAGTGTTAGCCCTCTCAATTCTGCTCCTTGGCT
AGATGTCATGCTTCACGCTGCAGAGAAACTGAAATGGGTTGATGAAGAGATTGAAAT
GATTGTCTTTTCTGACAACTTTATTAATTCTCAAGATGAAACTGTCAACCACGTTAGA
AATGAGCTGGATCGTGCTGATATTTTGGTGCTGGTTGCTGTCTGTAAGGAAGGATCGG
TCAACTGGATACAGACTAACAGCCAGAATGTCCCAAACATCATATGCTTTGACTCCTC
TTCGGCACTGAGAAACAAGCTCGGCGGAATTTTGATTGAAACTGAAAAAAGAGGAG
ACATATTCAGCAGTTTACTTCCATTTTCTCAATCTAAGAAAATGAATGAATCTGTAGA
GATAGCCCGAATAAACCTTAGAGCAAAAGGATTCTCCACCCTGAACTGCATGGTGAC
AAATTGCGGACCTCAGCTATTGAATTGTCTACTGGATCCTAATTATAGGAAGGCACTT

CAGTGCTTGAACCAGTGCAGCCCTGTAGATCAAGTATGCAATTATCGGTGTATTGCTT
CATATAAGAGTCAATACCTGGAAGAGTTTTCTCTCTGTGTACTACAGAAGAATAACTG
TCTTGAGCTCGACGCAAATATCCCTGAAAAACCTTATGTACCTCCAATGACCGAGTTT
CGAGGGGAAATATTGTCTTCTGAACTTGCTGAGGACCTTTTTGTGGGTTGGTTAGGGA
CATTGAATTGGAGTTGGCGCGTTGTAGCAGGGCAGAATCCAGCTTATGATCAATTTCC
ATGCCAGTACCAGCTATACCATCGGGGAAAAGCTAGAGGATCATTCTGTGTATTGGA
TAATGGAGTTGTTTCAATTGAGTCCTGGACAATTGTGGATGTTTCTGATGATTTAAGCT
GGGGTTTGTTTCACTATCATGGAGCTGCACGAGTGGCGGGGCAGTCATATACCGGGG
CGGTTCTTGTGAGCCCAGATGGCCAATATCCAGCTGAGAGGAAAAAAGAAAGGTTG
GTATCTGCATTGGATAGATGTGGCATCAAAGAGTGGGAGCTATTCAATGTTGATAAAT
GTTCATGTGAAGGTCCACCGTTGGGGCTTCCAGAGAGGTCAAGTTTGCATTCTAAGAT
TGAAGTCCAAGAGGACAGGACACATTCTTCAGTATAG

>ZE1_Ntab0136170

ATGTATTCAACTGTGTTTTACACTTCAGTTCATCCATCAACTTCAGCTTTTTCAAGAAA
GCAGCTGCCTTTCTTGATCTCTAAGGATTTCCCAACAGAGTTGTATCATTCTTTACCAT
GCAGCAGAAGCTTGGAAAATGGTCAAATCAAGAAAGTCAAAGGAGTAGTAAAAGCC
ACAATAGCTGAAGCTCCAGCTACTACTCCTACAACTGATTTGAAAAAGGTACCACAG
AAGAAGTTGAAAGTACTAGTAGCAGGTGGTGGGATTGGAGGGTTAGTGTTTGCATTG
GCTGCAAAGAAAAGGGGATTTGAGGTGTTGGTATTTGAGAGGGATTTAAGTGCTATT
AGAGGAGAAGGGCAATATAGAGGACCAATTCAGATACAGAGCAATGCATTGGCTGC
TTTGGAAGCAATTGACATGGATGTTGCTGAAGACATAATGAATGCTGGCTGTATCACT
GGTCAAAGGATTAATGGCTTGGTTGATGGTGTTTCTGGTAACTGGTAATTTCACATCA
CTCTAATTTCTTTGTGCTAAGTCTTTTTTTTTCCTGGCTAAGTAGCTGAATAGAACTTAA
TTTTACAAACTCCTCTTTGCTGTATGACTCTTATTCGAATTGACGAGTAATTTGATTGG
ATCATCTGTTATTTCACTCTTAATAATAACTTGAATCTCGCTTAATTCTTAGATATGAA
GTTTGTCCTTTGGCCAGTTCGAATTCACTAGTTATTGGTGCTTTAGAAATTGGAATCTT
GTCTTCTTTATTTTTCTTAGTAATAATCTTACATAAGCTTTTAAGTGAGGAAAAACCAA
GAAAATAATGAATTGTTTGAAGTACTTAGCCTTTCAGAGCTTAAACTGGAACTTAGTA
GAGGGGAAAACCTTTAGAAATTGAATTAGTCAAAAGTTTACTGAGTCATAGTTGTCA
AACTTATTTCTGGAAAAGGATCCATCTTTACCCTGCAATTTTACTGATAAATTGGTCTC
AATTATTGTATTTGTATGAGCAGCTTTATTGCCTTTTGGGTTTTGGCTGACTTTGTTACG
AGAACAGTTCCTTTAAATCTTGAGCTGCAAATTGTGACAATTGTTGGGTGTCAGGTAT
TGCAAGTTTGATACTTTCACTCCAGCAGTGGAACGTGGACTTCCTGTGACAAGAGTAA
TCAGCCGCATGACTTTGCAGCAGATCCTTGCACGTGCTGTTGGGGAGGATATAATTAT
GAATGAAAGTAATGTAGTTAACTTTGAGGATGATGGTGAAAAGGTTATATTAGATTTG
ATCTCTTTGTTTTCTTCTCTTCTCAAATATCAAGAAAGATTATAACTTTCTTAGTTTCAT
TCACGATCGTGTAGATCTTTCTTTGGAGTTTCATCAGTGTTAACTGTACCTAATTGTTCT
GTCTTATTCATCAATTTTGAATACAGCTTCTCATGTATTGTTTTGAAATCAGGTTACTGT
GACTCTTGAAGATGGACAGCAATATTCAGGTGATCTTCTGGTTGGTGCTGATGGCATA
AGGTCTAAGGTATTCAAAATCGGTCTCCTTCTATTTCCTTTTATTAATTACTACTTTGGT
TAACAAGGATAGAGTGACTTGTGTGTATTGTTACTTTAAGGTTGTGGTCCAGGTTAAA
AAATAGGCCTGAGTCTGTATTGCGCGGTGAACCTTTGTTGGTTAATTCTACTTGGTTTA
GAAGATCTCAAACATTGGCCTTTTATTGTAGGTACGGACTAATTTGTTTGGACCCAGT

GATGTTACTTACTCTGGCTACACTTGTTACACTGGAATTGCAGATTTTATATATTGAGA
CAGTTGGGTATGAAATTTTACTTAGGATTTTGTTTTCCCTCTTCATGTCCTTATCTCTGT
TGGTCCCGTTACGCTCACGCTAATGTTTTGATAGTAGAGGTTAAATTGAGTTTTTTTTTT
TTTCCTGAAGGTACCGAGTTTTTTTGGGCCACAAACAGTACTTTGTTTCTTCAGATGTG
GGTGGAGGCAAGATGCAGTGGTATGCATTTCACAATGAACCAGCTGGTGGCGTGGAT
GATCCAAACGGTAAACAATCTTAGGCCGTTTTAAACTATTTACTGGAAGACATTTTTG
AATGCTCGACTTGTTACATAACTTCAATTTCATCCAAGGGGTTTCTTAACGGATTTTTC
CTCATTAAGACCTGATTATAGTGTGGCAATAAGGTAAAGAAGAACTCATAGATTAGA
ATGAGATAAATTCGACGAATTATGCGTTGGGAAGTTTTGAACTGAAGTGCTGATTCGT
ATTTAAACTAAAATTGTGGTAAGCAGGTGCACTCTAATTAATCTACTTAATTTCCGTA
GAAGTTTCAGAGAATGATGCACGACAATTGGTTGTATCTACTCATAAATGTGAGGTTC
TTACTTCACCGCAGAAATTGTTTGTTAACTTATAGCTGAAAGTAAAAGAATATTTTCA
TCGTAGAATTTATTGTGGCAGCATCTGATTGTGTAGCGTAAAAAAACAAAGTCAAAA
CTGATAAGGTTCAAGAAACATCCATACTCTCGCAGCATTTGTGTAGACATTTATGTCA
TTGTATTGCAGAAAATGGTCATCAGGTTTTTATCTATTAGGCCTCTAATAATCCATTTT
CACTCTCTTGCCATATATATTTAAAGAATGATTCTTCTCTCTCCCCCTCTCGTTCCCCTC
CCGTGCCTGCAACCATTAAAGTTGTAATCACAAACTTTGTTTTCCGCTCTCTCTATCCC
CTTTTTTGGGGTCCCCATGAACCGCTTTTTAGTATTCGTGTTCGACTTCTTTCTTCCTTCC
CTTCCACACCGAAGGATCATGGGGTTCTCCTAACATACATATCTGGGGGTGCAACTGT
TTAGTAGGCAAGGATCGGAACGGGTTGGGAGGGGGGTTTCAGGCAATAAGTATGTTA
TCATGAATATTTAAATTGCCACTATGATGCTCAAGAGGTTGAGAAATATTTATAGTCA
TCTTGTCATTACATTGATTTTACAATATATTTGTTGTTCATGCTGCAGGTAAAAAGGCA
AGATTGCTTAAAATATTTGAGGGATGGTGTGATAATGTTATAGACCTATTAGTTGCCA
CAGATGAAGATGCAATTCTTCGACGTGACATCTATGATAGACCACCAACCTTTAATTG
GGGAAAAGGTCGTGTTACATTGCTTGGGGACTCTGTCCATGCTATGCAGCCTAATTTG
GGTCAAGGGGGATGCATGGCCATAGAGGTAAACTCTTTATATTAGCCTTTGTGGCTTC
GCTTAATTAAAATGGCATTGTTGGTCGATTGTACCCCTTAGTCCCTTCTTGCTAATAAG
CAGGGCTGAGTGAGGACTTAAAACAATCGTGCAGGACATAATTGTTAGGAGTCATAT
TTAGGGAGATTCTAGTTGAGAGAACCTAAAAGTACAAACTTAATCAATGGTTATGGG
GGTATCTCTGGAAAGTTATGGAATTTAACGACCCCTAGCAAGAAAAAGTTTTTTGTTT
TAGTTAGACCTGTCGCGGACGGTATAAATTTAGCAACCCTTTTTCCACCGGATCCATT
GCAGGAAAGGGATAATGCGCAACTTCGAATTGTCAATTGTATCCTTTATGGAAATGG
CAAACCGATTCAAGGAATTTTTGACACAAGTATTCAATTAGTTCGGACTTGTTTAGTA
TTAAATTGGAACCAAGACAAAAAAAGTTCTTCCTGGTTAACTAGTCATTGGTCTTTGG
CACCAAAGAAAGTCGAGAAGGTTGGGACGCAAGGTGGTTGTCGCTCAAGCAACTGT
GGAAGCAGACTCCGTGAAAAAGTCGTGTGCATCAATGAAATGGAAGGGCTCTCCTTC
TCTATCTTGAACCTAGCTCTGCCCAGTTTGGATGAACAAAGTGCACAATGCGTGGAGT
GAGATCACTACCATGCCTTTTTCAATTTATCTTGTGAACATAGGCTTCGAGTGCGTACC
TGGCGAGACTATGACTGTGTCAAGCGGGCGGGTACTTTATTCTTTTTTCTTTTTTTCTTT
TTAGTGGAGGTGGAAAGTGGGTATATGAGATTTTGGCTTCTGCCTTTTTTCCAAACTA
AAACCCTTTGTCATGAGAAGACATTTCTGTATTTCTCTCCATCTACCAGTTTTTTAATA
TTGTTCCTGTCGTTAAATAGGGGTTAAATATTTGGAAAAACATTGGGGTTAAATATGT
CCATGTAAGTAAGTATCATGAAGTGTAAACTGGATCACCTAATGCTCTTTGTATATCA
TCTTTGTCAAATACACGTATTATTGTAAGGTTGTATATGCCACTGAAATTTTCCTACCA

CAGGATAGCTATCAACTAGCACTGGAACTTGATAAAGCATTGAGCCGAAGTGCCGAG
TCAGGAAGCGCAGTGGATATCATCTCATCTTTAAGGAGGTAATACATTATTTATTGGC
TCAAGTGCTGTATAGTGGTTGGTTGAGTACGGGTTGCAACTTCACGATAATTAAAAAG
TCAGTTCCAATTGGATGAAGTTCTAACCCACTCTACCACCAGCCTGAAAAAGACCTG
CTTTAAATGCCATATCTGAACAAGTGCAGGAGATGAGGCATAATATTTGCATAACATT
ATATATGGAACTTCTCATCCATTAGTTCTGAAGCACCTCTCTTGTAGTCTGAGCTCAAA
ATTGTGGAGTGACTTGAACAGCTTCTATTTCTGCCAGTTATCTGATTTTTTATGATTTCC
CTTGTCTCCAGCTATGAAAGTTCTAGAAAACTTCGAGTTGGAGTTATCCATGGACTGG
CTAGAATGGCTGCAATCATGGCATCAACTTACAAGGCTTATCTTGGTGTCGGACTTGG
TCCATTATCAGTATGGAGATCTGTCAATAACTAGAAATTGGAGTAGCTAATTCGTCTG
ATTTAGCAGAGCTCTTCTTATAGATATTGTTTTCTATTATTTTTGCAGTTTTTGACCAAG
TTTCGGATACCACATCCTGGAAGAGTTGGTGGAAGATTTTTTATTGACTTGGGAATGC
CGCTTATGTTAAGCTGGGTTCTAGGAGGCAACGGGTAGGAATATGCAATCCTTATCTT
GCTTCAATATCGTTAAACATCATCTTGGTTTCCTTGCGAGATCAGCCCATAGCTAGAG
AGTAAAATCGCAATGCCACATCTATTTTTGCTTTTCGTTATAGCAGCTCTTGGGGACTT
TTATCTCAGTTCGTGGTTATTTGTTGATTCTGTTTCGTCAAAACTTATACTGGAAATTCT
TCTATTACATCTAAAGCTTTCAAATTATTATTATTATTTTTTTAAAGAAATATTAGAAG
TTTATGATTTTCAGCATCTGACATTATATCTTTGAAGATACTACTTACTGTAAAGTCAA
GTGCCAGCATGTGAATCCATGATGAATTTACTGGAATTCTTGGAAGTTGATACGTCCT
CTCGGTTGACTCGTCTGTTTTTCTTGCAGTGAAAAGCTTGAAGGCAGAATAAAACATT
GCAGGCTATCTGAGAAAGTAAGTTGTGTAAGGAACTATTCACTCAGCACACGAGCAC
AGAAAAGATTTGTGGCTTCTATTGTTACCTAATAAAGGTCGAAGTACTGAATTTTATG
GGAGTAATAATATACTGTTTTGTGGTGATGAAGGCAAATGACCAATTGAGGAATTGG
TTTGTAGATGATGATGCTTTAGAGCGTGCTACTGATGCAGAGTGAGTTTGAGAAACAT
AATATTTACAATTTTCATTTTCACGTGTCCCCTATTTTTCTAGTTTGCTTGCTAAATTTG
GTGCTCACTTGTTATCTTTCAGGTGGTTACTGCTTCCTGCCGGGAATGGCAATGCTGCT
GTAGAAGCTCTTGTTTTAAGCAGAGATGAGGATATGCCTTGCACTATTGGGTATGCTT
CTAGGTCTTAGCGCAAAGTAATCTTTGTTATTGTTTGATTTAAATGAGCAAAATGCTCT
CTATTTTTAAAGATTTACAGCATTTAAATGACACAACTTTCTTTGAGGGGATGGTTTAA
GTAATTTTACTGGATAAAATGAGCATTCAAGAGAGTATAAAGCTACAGAACTTGATA
TTTCTGTTTGTTTATTTGAATTCTTATTATTTAAAGCTCTTGAGGTATTCATAATTGTTCA
AAAGATCATGCGTTGTGGTGCATCAATTGCAAATATCAATCTCCTCTAATAAACATAT
CTGATCCATTTGCATATCAAAAGTACGAAGCAGACAGGAAAGTAATGTCATCTAACC
GAACATTGCTGTAAGAGAGGAATCTTTACTGCAATATTTGCTTCGTTAGAAGAGTCGC
TGATGATTTTCTGGTTTCAGGTCTGTCTCACATACAAACATTCCTGGAAAATCACTAGT
TATACCTTTGCCTCAGGTGGTTGCCATCTGTGATCATCTCTTATATGAGTCTATAGCTA
AGTATTGGACATTCTCCTAGTGAATGCATGCTTAGGTCATCTATTTTTCTTTTTCTAATC
TCAGGTGTCCGAAATGCACGCCCGGATATCCTACAAAGGTGGAGCTTTTTTTGTAACT
GATTTACGAAGTGAACATGGTACCTGGATTACGGAGTAAGTTCTACACTCAAGTATA
CTGTACTATTACACTGACTAGTATTTTATGAAGAGAAAATTAATGATTGTCCTGTCTTT
GTAGTAATGAAGGCAGAAGATACCGAGCGTCTCCAAACTTTCCTACTCGTTTTCATCC
ATCAGATATTATTGAATTTGGTTCTGATAAGAAGGTGAGATACAAAACCTCTTCCTCG
ATATGCTTTAGACGAAGTATTGGCACTAGCATTATTCATTTAAATTTGTGTGTATGTTA
TGCAGGCAGCATTTCGTGTAAAGGTAATGAAATTTCCTTCAAAAACTGCTGCAAAGA

AGGAAGAGCCTGAAGTTTGGCCTGATATAAACAGGGAAATTGTACAGCATTTTTATA
GCAACAGCAGAAACTGA

>mRNA_119539_cds

ATGTATTCAACTGTGTTTTACACTTCAGTTCATCCATCAACTTCAGCTTTTTCAAGAAA
GCAGCTGCCTTTCTTGATCTCTAAGGATTTCCCAACAGAGTTGTATCATTCTTTACCAT
GCAGCAGAAGCTTGGAAAATGGTCAAATCAAGAAAGTCAAAGGAGTAGTAAAAGCC
ACAATAGCTGAAGCTCCAGCTACTACTCCTACAACTGATTTGAAAAAGGTACCACAG
AAGAAGTTGAAAGTACTAGTAGCAGGTGGTGGGATTGGAGGGTTAGTGTTTGCATTG
GCTGCAAAGAAAAGGGGATTTGAGGTGTTGGTATTTGAGAGGGATTTAAGTGCTATT
AGAGGAGAAGGGCAATATAGAGGACCAATTCAGATACAGAGCAATGCATTGGCTGC
TTTGGAAGCAATTGATATGGATGTTGCTGAAGACATAATGAATGCTGGCTGTATCACT
GGTCAAAGGATTAATGGCTTGGTTGATGGTGTTTCTGGTAACTGGTATTGCAAGTTTG
ATACTTTCACTCCAGCAGTGGAACGTGGACTTCCTGTGACAAGAGTAATCAGCCGCA
TGACTTTGCAGCAGATCCTTGCACGTGCTGTTGGGGAGGATATAATTATGAATGAAAG
TAATGTAGTTAACTTTGAGGATGATGGTGAAAAGGTTACTGTGACTCTTGAAGATGGA
CAGCAATATTCAGGTGATCTTCTGGTTGGTGCTGATGGCATAAGGTCTAAGGTACGGA
CTAATTTGTTTGGACCCAGTGATGTTACTTACTCTGGCTACACTTGTTACACTGGAATT
GCAGATTTTATTCCTGCTGATATTGAGACAGTTGGGTACCGAGTTTTTTTGGGCCACAA
ACAGTACTTTGTTTCTTCAGATGTGGGTGGAGGCAAGATGCAGTGGTATGCATTTCAC
AATGAACCAGCTGGTGGCGTGGATGATCCAAACGGTAAAAAGGCAAGATTGCTTAA
AATATTTGAGGGATGGTGTGATAATGTTATAGACCTATTAGTTGCCACAGATGAAGAT
GCAATTCTTCGACGTGACATCTATGATAGACCACCAACCTTTAATTGGGGAAAAGGT
CGTGTTACATTGCTTGGGGACTCTGTCCATGCTATGCAGCCTAATTTGGGTCAAGGGG
GATGCATGGCCATAGAGGATAGCTATCAACTAGCACTGGAACTTGATAAAGCATTGA
GCCGAAGTGCCGAGTCAGGAAGCGCAGTGGATATCATCTCATCTTTAAGGAGCTATG
AAAGTTCTAGAAAACTTCGAGTTGGAGTTATCCATGGACTGGCTAGAATGGCTGCAA
TCATGGCATCAACTTACAAGGCTTATCTTGGTGTCGGACTTGGTCCATTATCATTTTTG
ACCAAGTTTCGGATACCACATCCTGGAAGAGTTGGTGGAAGATTTTTTATTGACTTGG
GAATGCCGCTTATGTTAAGCTGGGTTCTAGGAGGCAACGGTGAAAAGCTTGAAGGCA
GAATAAAACATTGCAGGCTATCTGAGAAAGCAAATGACCAATTGAGGAATTGGTTTG
TAGATGATGATGCTTTAGAGCGTGCTACTGATGCAGAGTGGTTACTGCTTCCTGCCGG
GAATGGCAATGCTGCTGTAGAAGCTCTTGTTTTAAGCAGAGATGAGGATATGCCTTGC
ACTATTGGGTCTGTCTCACATACAAACATTCCTGGAAAATCACTAGTTATACCTTTGC
CTCAGGTGTCCGAAATGCACGCCCGGATATCCTACAAAGGTGGAGCTTTTTTTGTAAC
TGATTTACGAAGTGAACATGGTACCTGGATTACGGATAATGAAGGCAGAAGATACCG
AGCGTCTCCAAACTTTCCTACTCGTTTTCATCCATCAGATATTATTGAATTTGGTTCTG
ATAAGAAGGCAGCATTTCGTGTAAAGGTAATGAAATTTCCTTCAAAAACTGCTGCAA
AGAAGGAAGAGCGTCAAGCAGTGGGGGCAGCTTGA

>ZE2_Ntab0384590

ATGTATTCAACTGTGTTTTACAATTCAGTTCATCCATCCACTTCAGTTTTTTCAAGAAA
GCAGCTGCCTTTATTGATCTCTAAGGATTTCCCTGCAGAATTGTGTCATTCTTTACCAT
GTAGTAGAAACTTGGAAAATGGTCAAATCAAGAAAGTCAAAGGAGCAGTAAAAGCC

ACAATAGCTGAAGCTCCAGCTACTATTCCTACAACTGATTTGAAAAAGGTTCCACAG
AAGAAGTTGAAAGTACTAGTAGCAGGTGGTGGGATTGGAGGGTTAGTTTTTGCATTG
GCTGCAAAGAAAAGGGGATTTGATGTGTTGGTATTTGAGAGGGATTTAAGTGCTATTA
GAGGAGAAGGGCAATATAGAGGACCAATTCAGATACAGAGCAATGCATTGGCTGCT
TTGGAAGCAATTGACATGGATGTTGCTGAAGACATAATGAATGCTGGTTGCATCACTG
GTCAAAGGATTAATGGTTTGGTTGATGGTGTCTCTGGTAACTGGTAAATTTCACATCA
CTCTAAGTGCTTGACTGAATAGAAATTCTTGGTGTTATTTAACATATGTCTTTTTTACA
CTGTAGCAATCATACACAAACTATTACTGTGGAAAATCCAAGAAAATAATGAATCCT
TTGAAATATTTAGCCTTTCAGAAATTATACTGGAATTAGTAGAGGGAAAATAATTGAC
TTAGTCAAAAGTTTATGTTCATAGTTGTCAAACTTATTACGAAAAAGGATCTATCTTTA
CCTTCAATTAGATTGATAAATTGGTCTCATTATTGTATAAGCAATTTTCTTGCGAGGAG
AGTTCCAAGAAAATAATGAATCCTTTGAAATGTTTAGCCTATCAGAAATTACACTGG
AATTAGTAGAGGGAAAATAATTGATTTGGTCCAAAGTTTATCGGTTCATAGTTGTCAA
ACTTATTTTGGAAAAGGATCTATCGTTACCTTCAATCAGATTGATAAATTGGTCTCATT
ATTGTATGAGAAGCTTTCTTGTGAGAACAGTGCCTTTGTTTATATACTTGAACTGCATA
GGGTGACAATAGTTTGGTGTCAGGTATTGCAAGTTTGATACGTTTACTCCAGCAGTGG
AACGCGGACTTCCTGTGACAAGAGTCATCAGCCGCATGACTTTGCAGCAGATCCTTG
CACGTGCTGTTGGGGAGGATATAATTATGAATGAAAGTAATGTAGTGAACTTTGAGG
ATGATGTTGAAAAGGTAATATTAGATTTGATCTCTTTGTTTTCTTCTATTCTCAAAATAT
CAAGAAAGATTATAGCTTTTGTTTGTTTCATTCACAATCGTGTAGATCTTTCTTTGGAG
TTTCATCAGTGTTGACTGTACCTAATTGTTCTGTCTTATTCATCGATTTTGAATACAGCT
TCTCATGTGCTGTTTGAAATCAGGTTACTGTGACTCTTGAGGATGGACAGCAGTATTC
AGGTGATCTTCTGGTTGGTGCTGATGGCATAAGGTCTAAGGTATTCAAAATCGGTCTC
CTTCTATTTCCTTCTATTATAATTACTACTTTGGTTAACAAGGATAGAGTGACTTGTCT
ATATTGTTACTTTGAGGTTGTGGTCCAGGTTAAAAAATAGGCCTGAGTCTGTATTGCG
CGGTGAACAATTGTTAGTTAATTCTAATTGGTTTAGAAGATCTCAAACATTGACCTTTT
ATTGTAGGTAAGGACTAATTTGTTCGGACCCAGTGATGTAACTTACTCTGGCTACACT
TGTTACACTGGAATTGCAGATTTTGCCTGCTGATATTGAGACAGTTGGGTATGAAATT
TTACTTAGGATTTTGTTTTCCCTCTTCATGTCCTTCTCTCTGTTGGTCCCGTTACACTCGC
ATTATGTTTTGATAGTAGAGGTTAAATTGAGATTTTTTTTCTGAAGGTACCGAGTCTT
TTTGGGCCACAAACAGTACTTTGTTTCTTCAGATGTGGGTGGAGGCAAGATGCAGTGG
TATGCATTTCACAATGAACCAGCTGGCGGTGTGGATGATCCAAATGGTAAATATTATT
AGTCCGCTTTAAACTATTTATTAGAAGACATTTTTGAATGCTTGACTTGTTACATAACT
TCTATTTCATCCAAGGGGTTTCTTAAGGGATTTTCCCCTATTGTGACCTGATTATAGTG
TGGCAATAAGGTAAAGAAGACCTCGTAGATTAGAATGAGATAGATTCGACGAATTAT
GCATTGAGAAGTTTTGAATTGAAGTGCCTATTCATATATAAACTAAAATTGTGGTAAG
CAGGTGCACTCTAATTTATCTACTTAATTTCTGTAAAAGTTTCAGAGAATAATGCACC
ACAGCCCCCCGGGCCTAGCTCAAGTGGCAAAGGGTTGTGGATTTGTGTCTTAGGTCAC
AGGTTCAAGCCCCCACACCATGCAAAGCAAAGCCTGTTATTTAAGTGGAGAAGAGTA
GAGGGACGGGCCCATTATTCACCGAGTTTCGAAGGCTGCGGTTGGTCCAAAGGATCG
GCCCCAGACGGATTTCTCGGTCATCAAAAAAAATAATGCACCACAATTGGTTGTATCT
ACTCATAAATGTGAGGTCCTTACTTCACCGCAGAAATTGTTTTTTAACCTATAGCTGA
AAGTTAAAGATTGTATTCAAAGTAGAATTCATTAGTGACGGCATCTGATTGTGTATCG
TAAAAAAAACAAAGTCAAAATTGATAAGGTTTCAAGAAACATCCATACTTTCACAGC

ATTTGTGTAGACATCTATGTCATTGTGTTGTAGAAAATGGTCATCAGATCTTTATCTAT
TAGGCCCTCCAATAATCCATTTTCACTCTTTTGCCATATATTTTAAAGCCTTATTCTTGC
TACTATCTGTTTATTGCTTCATTTTCACTTCGCTTGAGCCGAGGGTCTTTTGGAAACAG
ACTCTCTACCGTCATAAGGTAGGGGTAAAATCTGCATACACTTTACCTTTCCCAGACC
CCACTTGTAGGATTTCACTGGGTCTTTTGTTGTTGTTGTTGTTTGCCATATATTTTAAAG
AATGAGTCTTTTCTTTCGAAGGGGAGCCTTGGCGTAACTGGTAAAATTGTCTCCATGT
GACCAGGAGGTCATGAGTTCGAGCTGTGGAAACAGCCTCTTGAAGAACTGCAGGTAA
GGCTACGTATAATAGACCCTTGTAGTTCGGCCCTTCCCCGGACCATGTGCATAGCGGG
AGCTCCGGGCTGTCTTTAGTCTTCTCTCTTCCCCCCTCTCTCGTGCCCCTCCCTTGCCTG
GAACCATTAAATTGGGAATCACAGACATTTTTTTTTGTTGGGGGTGTGGAGGGAGCAG
CGATGCAGGCGATAAGTATGTTATCATGAATATTTAAATTGCCACTATGATGCTCAAA
AGGTTGAGAAATATATATAGTCATCTTGTCATTACATTGGTTTTACAATATTTTTGCGG
TTCATGCTGCAGGTAAAAAGGCAAGATTGCTTAAAATATTTGAGGGGTGGTGTGACA
ATGTTATAGACCTATTAGTTGCTACAGATGAAGATGCAATTCTTCGACGTGACATCTA
TGATAGACCGCCAACCTTTAATTGGGGAAAAGGTCGTGTTACATTGCTTGGGGACTCT
GTCCATGCTATGCAGCCTAATTTGGGTCAAGGGGGATGCATGGCCATAGAGGTAAAC
TCTTTATATTAGCCTTTGTGACTTCACTCAATTAAAATGCCATTGTTGATCGATTGTAC
CCCCTTAGTCCCTTCTTGCTAAGAAGCAGGTCTGAGTGAGGACTTAAAAAAATCATGC
AGGACATAATTCTTAGTCAATGGTTATGGGGGTATCGCTGAAATGTTATGGAATTTAA
CTAGACCTTTATTCTTATTTCTTTTTTGCTTTTTAGTGGAGATGGAAAGTGGGTAGATG
AGATTTTGGCTTCTGCCCTTTTTGTCAAAAGAAGACCCTTTGTTATGAGAAGACATTTC
CGTATTTCTCTCCATCTACCAGTTTTTTTACATTGTTCCTGTCATTAAATAGGGGTTAAT
ATTTGGAAAAACATTGGGGTTAAATAGGTCCATGTAAGTAAGTATCATGAAATGTAC
ACTGCATCACCTAATGCTCTTTGTCTATCATCTTTGTCAAATACACGTATTATTGTAAG
TTTGTATATGCCACTGAACTTTTCCTTGTACGACCACAGGATGGCTATCAACTAGCAC
TGGAACTTGATAAAGCATTGAGCCGAAGTGCCGAGTCAGGAACCCCTGTGGATATCA
TCTCATCTTTAAGGAGGTAACCATTATTTATTGGCTCGAGTGCTGTATAGTGGTTGGTT
GAGTACAGGTTGCAACTTCACGATAATTAAAAAGTCAGTTCCAATTGGATGAAGTTCT
AACCTCACCTATCACCTGCCTGATCTTTAAAAGCCATATCTGAACAAGTGCAGGAGA
TGAGGCATAATATTTGCATAACATTCTATATGGAACTTCTCATCCATTAGTTCTGAAG
CAATTCTCTCGTAGTCTGAACTCAAAATTGTGGATTGACTTGAAGAGCTGCTATTTCTG
CCAGTTATCTGATTTTTTATGATTTCCCTTGTTCCCAGCTATGAAAGTTCTAGAAAACT
TCGAGTTGGAGTTATCCATGGACTGGCTAGAATGGCTGCAATCATGGCATCAACTTAC
AAGGCTTATCTTGGCGTCGGACTCGGTCCATTATCAGTATGGAAATCTGTCTATAACG
TGAAATTGGAGTAGCTAATTGGTTTGATTTAGCAGAGCTCTTCTTATAGATATTGTTTT
CTATTATTTTTGCAGTTTTTGACCAAGTTTAGGATACCACATCCTGGAAGAGTTGGTGG
AAGATTTTTTATTGACTTGGGAATGCCGCTTATGTTAAGCTGGGTTCTAGGAGGCAAC
GGGTAGGAATATACAATCCTTATCTTGCTTCAATATCCTTAAACATCATCTTGGTTTCC
TTGCGAGATCAGCCAATAGCTAGAGAGTAAAATCGCAATGCCACATCTATTTTTGCTT
TTTGTTATAGCAGCTCTTGGGGTGGCCCCTGCCTTTTATCTCAGTTTGTGGTTATTTGTT
GACTCTGTTTCGTCAAAACTTGTACTGGAAATTCTTTTGTTACATCTAAAGCTTTCAAA
TATTTTAAAAAAAAAAAAAGAAATATTAGAAGTTTACGATTTTCTATTGCTAGGGCG
ATGTCTAGCAACTGATCTATTGGATTTTTTCAGCATCTGGCGTTAAATCTTTGAAGATA
CTAGTTACTGTAAAGTCAAGTGCCAACATGTGAATCCATGATGAATTTACTGGAATTC

TTGGGAGTTGATATGTTTTCTGACTCGTCTGTTTTTCTTGCAGTGAAAAGCTTGAAGGC
AGAATACAACATTGCAGGCTATCTGAGAAAGTAAGTTGTGTAACGAACTGTTCACTC
AGCACACGCGCACAGAAAAGATTTGTGGCTTCTATTGCTCTACTTAATTAAGGTCGAA
TTATTGAATTTTATGGGAGTAATAATATACTGTTTGTGGTGAAGGCAAATGACCAAT
TGAGAAATTGGTTTGAAGATGATGATGCTTTAGAGCGTGCTACTGATGCAGAGTAAG
TTTGAGAAACATAATATTTACAAATTTCATTTTCACCTGTCATATTTTTCTAGTTTGCTT
GCTAAGTTTGGTGCTTACTTTTTATCTTTTAGGTGGCTACTGCTTCCTGCTGGGAATAG
CAATGCTGATTTAGAAACTCTTGTTTTAAGCAGAGATGAGAACATGCCTTGCACTATT
GGGTATGCTTCATGTTTTATTGGATTTATGATTGTTCAAAAACTGGCTGCTAGGTCTTA
GCATAGAGTTATCTTTGTTATTGTTTGGTTTAAATGAGCAAAATGGCTCTCTCTACTTT
CAAAAATTTTGCAGCATTTAAATGATTTAGCTGCCTTTGAGGAGATGGTTTAAGTCAC
TTTACTGAAAATTGAGTGCTTACGACAAGAGTATAAAGCTACAGAACATGAGATTTC
TCTTTCTTTATTTGAACTCTTATTACTTAAAGCTATCGAAGTATCCTAATTATTCAGAA
GATCATGCATTGTGGTCCATCCATTGCAAATATCTCATCATTAATCTCCTCTAGTACAA
TATTTGCTTCATTAAAAGAGCCACTGATGATCTCTTATTTCAGGTCTGTCTCACATACA
AACATTCCTGGAAAATCAGTAGTTATACCTTTGCCTCAGGTGGTTGCCATCTGTGCTC
ATCTCTTATATGAGTCTATAGCTAAGTATCATTGGACATTGTCCTAGTTGGATGCATTC
TTAGATCATCTATTTTTCTTTTTTTTTCAAATCTCAGGTGTCCCAAATGCATGCCCGGAT
ATCCTACAAAGGTGGAGCATTTTTTGTAACTGATTTACGAAGTGAACATGGTACCTGG
ATTACGGAGTAAGTTCTACACTCTAGTATACCCTACTATTACACTGACTTATGAAGAG
AAAAGTTATGATTATCCTGTGTTTGTAGTAACGAAGGCAGAAGATACCGAGCGTCTC
CAAACTTTCCTACTCGCTTTCATCCGTCAGATATTATTGAATTTGGTTCTGATAAAAAG
GTGAAATGCAAAAACTCTTCCTCGATATGCTGTAGACGAAGTACTAAAGTTAGCATT
ATTCACTTAAATCTGTACGTGCGTATGTTGTGCAGGCAGCATTTCGCGTAAAGGTAAT
GAAATTTCCTCCAAAAACTGCTGCAAAGAAGGAAGAGCGTCAAGCAGTGGGGGCAG
CTTGA

>mRNA_42563_cds

ATGTATTCAACTGTGTTTTACAATTCAGTTCATCCATCCACTTCAGTTTTTTCAAGAAA
GCAGCTGCCTTTATTGATCTCTAAGGATTTCCCTGCAGAATTGTGTCATTCTTTACCAT
GTAGTAGAAACTTGGAAAATGGTCAAATCAAGAAAGTCAAAGGAGCAGTAAAAGCC
ACAATAGCTGAAGCTCCAGCTACTATTCCTACAACTGATTTGAAAAAGGTTCCACAG
AAGAAGTTGAAAGTACTAGTAGCAGGTGGTGGGATTGGAGGGTTAGTTTTTGCATTG
GCTGCAAAGAAAAGGGGATTTGATGTGTTGGTATTTGAGAGGGATTTAAGTGCTATTA
GAGGAGAAGGGCAATATAGAGGACCAATTCAGATACAGAGCAATGCATTGGCTGCT
TTGGAAGCAATTGACATGGATGTTGCTGAAGACATAATGAATGCTGGTTGCATCACTG
GTCAAAGGATTAATGGTTTGGTTGATGGTGTCTCTGGTAACTGGTATTGCAAGTTTGAT
ACGTTTACTCCAGCAGTGGAACGCGGACTTCCTGTGACAAGAGTCATCAGCCGCATG
ACTTTGCAGCAGATCCTTGCACGTGCTGTTGGGGAGGATATAATTATGAATGAAAGTA
ATGTAGTGAACTTTGAGGATGATGTTGAAAAGGTTACTGTGACTCTTGAGGATGGAC
AGCAGTATTCAGGTGATCTTCTGGTTGGTGCTGATGGCATAAGGTCTAAGGTAAGGAC
TAATTTGTTCGGACCCAGTGATGTAACTTACTCTGGCTACACTTGTTACACTGGAATTG
CAGATTTTGTTCCTGCTGATATTGAGACAGTTGGGTACCGAGTCTTTTTGGGCCACAA
ACAGTACTTTGTTTCTTCAGATGTGGGTGGAGGCAAGATGCAGTGGTATGCATTTCAC

AATGAACCAGCTGGCGGTGTGGATGATCCAAATGGTAAAAAGGCAAGATTGCTTAA
AATATTTGAGGGGTGGTGTGACAATGTTATAGACCTATTAGTTGCTACAGATGAAGAT
GCAATTCTTCGACGTGACATCTATGATAGACCGCCAACCTTTAATTGGGGAAAAGGT
CGTGTTACATTGCTTGGGGACTCTGTCCATGCTATGCAGCCTAATTTGGGTCAAGGGG
GATGCATGGCCATAGAGGATGGCTATCAACTAGCACTGGAACTTGATAAAGCATTGA
GCCGAAGTGCCGAGTCAGGAACCCCTGTGGATATCATCTCATCTTTAAGGAGCTATG
AAAGTTCTAGAAAACTTCGAGTTGGAGTTATCCATGGACTGGCTAGAATGGCTGCAA
TCATGGCATCAACTTACAAGGCTTATCTTGGCGTCGGACTCGGTCCATTATCATTTTTG
ACCAAGTTTAGGATACCACATCCTGGAAGAGTTGGTGGAAGATTTTTTATTGACTTGG
GAATGCCGCTTATGTTAAGCTGGGTTCTAGGAGGCAACGGTGAAAAGCTTGAAGGCA
GAATACAACATTGCAGGCTATCTGAGAAAGCAAATGACCAATTGAGAAATTGGTTTG
AAGATGATGATGCTTTAGAGCGTGCTACTGATGCAGAGTGGCTACTGCTTCCTGCTGG
GAATAGCAATGCTGATTTAGAAACTCTTGTTTTAAGCAGAGATGAGAACATGCCTTGC
ACTATTGGGTCTGTCTCACATACAAACATTCCTGGAAAATCAGTAGTTATACCTTTGC
CTCAGGTGTCCCAAATGCATGCCCGGATATCCTACAAAGGTGGAGCATTTTTTGTAAC
TGATTTACGAAGTGAACATGGTACCTGGATTACGGATAACGAAGGCAGAAGATACCG
AGCGTCTCCAAACTTTCCTACTCGCTTTCATCCGTCAGATATTATTGAATTTGGTTCTG
ATAAAAAGGCAGCATTTCGCGTAAAGGTAATGAAATTTCCTCCAAAAACTGCTGCAA
AGAAGGAAGAGCGTCAAGCAGTGGGGGCAGCTTGA
